# Observational and genetic analyses clarify the relationship between type 2 diabetes mellitus and gallstone disease

**DOI:** 10.3389/fendo.2023.1337071

**Published:** 2024-01-31

**Authors:** Peijing Yan, Li Zhang, Chao Yang, Wenqiang Zhang, Yutong Wang, Min Zhang, Huijie Cui, Mingshuang Tang, Lin Chen, Xueyao Wu, Xunying Zhao, Yanqiu Zou, Jinyu Xiao, Yunjie Liu, Chenghan Xiao, Yanfang Yang, Ling Zhang, Yuqin Yao, Jiayuan Li, Zhenmi Liu, Chunxia Yang, Xia Jiang, Ben Zhang

**Affiliations:** ^1^ Department of Epidemiology and Biostatistics, Institute of Systems Epidemiology, and West China-PUMC C. C. Chen Institute of Health, West China School of Public Health and West China Fourth Hospital, Sichuan University, Chengdu, Sichuan, China; ^2^ School of Public Health, Southwest Medical University, Luzhou, Sichuan, China; ^3^ Clinical Research Center, Women and Children’s Hospital of Chongqing Medical University, Chongqing, China; ^4^ Department of Maternal, Child and Adolescent Health, West China School of Public Health and West China Fourth Hospital, Sichuan University, Chengdu, China; ^5^ Department of Iatrical Polymer Material and Artificial Apparatus, School of Polymer Science and Engineering, Sichuan University, Chengdu, China; ^6^ Department of Occupational and Environmental Health, West China School of Public Health and West China Fourth Hospital, Sichuan University, Chengdu, China; ^7^ Department of Nutrition and Food Hygiene, West China School of Public Health and West China Fourth Hospital, Sichuan University, Chengdu, China; ^8^ Department of Clinical Neuroscience, Karolinska Institute, Stockholm, Sweden

**Keywords:** type 2 diabetes mellitus, gallstone disease, Mendelian randomization, bidirectional, causal relationship, genetic association, cohort

## Abstract

**Background:**

The relationship between type 2 diabetes mellitus (T2DM) and gallstone disease (GSD) have been incompletely understood. We aimed to investigate their phenotypic and genetic associations and evaluate the biological mechanisms underlying these associations.

**Methods:**

We first evaluated the phenotypic association between T2DM and GSD using data from the UK Biobank (n>450,000) using a prospective observational design. We then conducted genetic analyses using summary statistics from a meta-analysis of genome-wide association studies of T2DM, with and without adjusting for body mass index (BMI) (N_case_=74,124, N_control_=824,006; T2DM_adj_BMI: N_case_=50,409, N_control_=523,897) and GSD (N_case_=43,639, N_control_=506,798).

**Results:**

A unidirectional phenotypic association was observed, where individuals with T2DM exhibited a higher GSD risk (hazard ratio (HR)=1.39, *P*<0.001), but not in the reverse direction (GSD→T2DM: HR=1.00, *P*=0.912). The positive T2DM-GSD genetic correlation (*r_g_* =0.35, *P*=7.71×10^-23^) remained even after adjusting for BMI (T2DM_adj_BMI: *r_g_* =0.22, *P*=4.48×10^-10^). Mendelian randomization analyses provided evidence of a unidirectional causal relationship (T2DM→GSD: odds ratio (OR)=1.08, *P*=4.6×10^-8^; GSD→T2DM: OR=1.02, *P*=0.48), even after adjusting for important metabolic confounders (OR=1.02, *P*=0.02). This association was further corroborated through a comprehensive functional analysis reflected by 23 pleiotropic single nucleotide polymorphisms, as well as multiple neural and motor-enriched tissues.

**Conclusion:**

Through comprehensive observational and genetic analyses, our study clarified the causal relationship between T2DM and GSD, but not in the reverse direction. These findings might provide new insights into prevention and treatment strategies for T2DM and GSD.

## Introduction

Both type 2 diabetes mellitus (T2DM) and gallstone disease (GSD) are prevalent and expensive global public health issues (1, 2). The coexistence of these two conditions, known as multimorbidity, poses complex challenges for clinical management (3). For example, while laparoscopic cholecystectomy is the gold standard treatment for GSD (4), individuals with diabetes are usually high-risk candidates for any surgery. Therefore, it is imperative to comprehend the association between T2DM and GSD, as well as the underlying biological mechanisms, to ensure effective prevention and management of this multimorbidity.

The association between T2DM and GSD has been studied, but results have been inconsistent (5, 6). A Meta-analysis of prospective cohort studies found no association between diabetes and GSD risk (7), while two latest large-scale prospective cohorts reported a 31%-87% increased risk of GSD in individual with diabetes (5, 8). Conversely, three prospective cohort studies shown that GSD increased the risk of T2DM by 17%-42% (6, 9, 10). However, these observational studies are prone to bias, confounding, and reverse causality, making it difficult to establish a causal relationship. Mendelian randomization (MR) studies, which address confounding factors and reverse causation (11) have been used to evaluate the causal relationship between T2DM and GSD. Three MR studies consistently suggest that T2DM causally increases GSD risk (12–14), one study explored and refuted a reverse causal association (6). Despite the knowledge gained from exiting MR analyses advancing our understanding of causal relationships underlying T2DM and GSD, a few major gaps remain. Firstly, the only previous MR suggesting that GSD does not cause T2DM may be inaccurate due to its insufficient statistical power, using merely eight single nucleotide polymorphisms (SNPs) (15). Additionally, multivariable MR (MVMR) can be used to control for pleiotropic impact (16). However, previous MR studies either controlled for no potential confounders (6, 14) or only controlled for body mass index (BMI) (12, 13).

Despite the increasing number of studies reporting evidence between T2DM and GSD, the biological mechanisms linking these two conditions remain unclear. Genome-wide cross-trait analysis presents an opportunity to comprehensively characterize the shared genetic architectures across traits, shedding light on the underlying biological mechanisms of complex diseases (17). Moreover, both T2DM and GSD are moderately heritable, with SNP-heritability estimates of 25% for GSD (18) and 25%-69% for T2DM (19). Large-scale genome-wide association studies (GWASs) have identified a number of disease-associated variants for GSD (20) (N=62) and T2DM (21) (N=386), of which, several risk loci, including *PNPLA3*, are shared by both conditions (22). However, to the best of our knowledge, no genome-wide cross-trait analysis has been performed to systematically investigate the underlying shared genetic architectures of T2DM and GSD.

**Figure 1:**
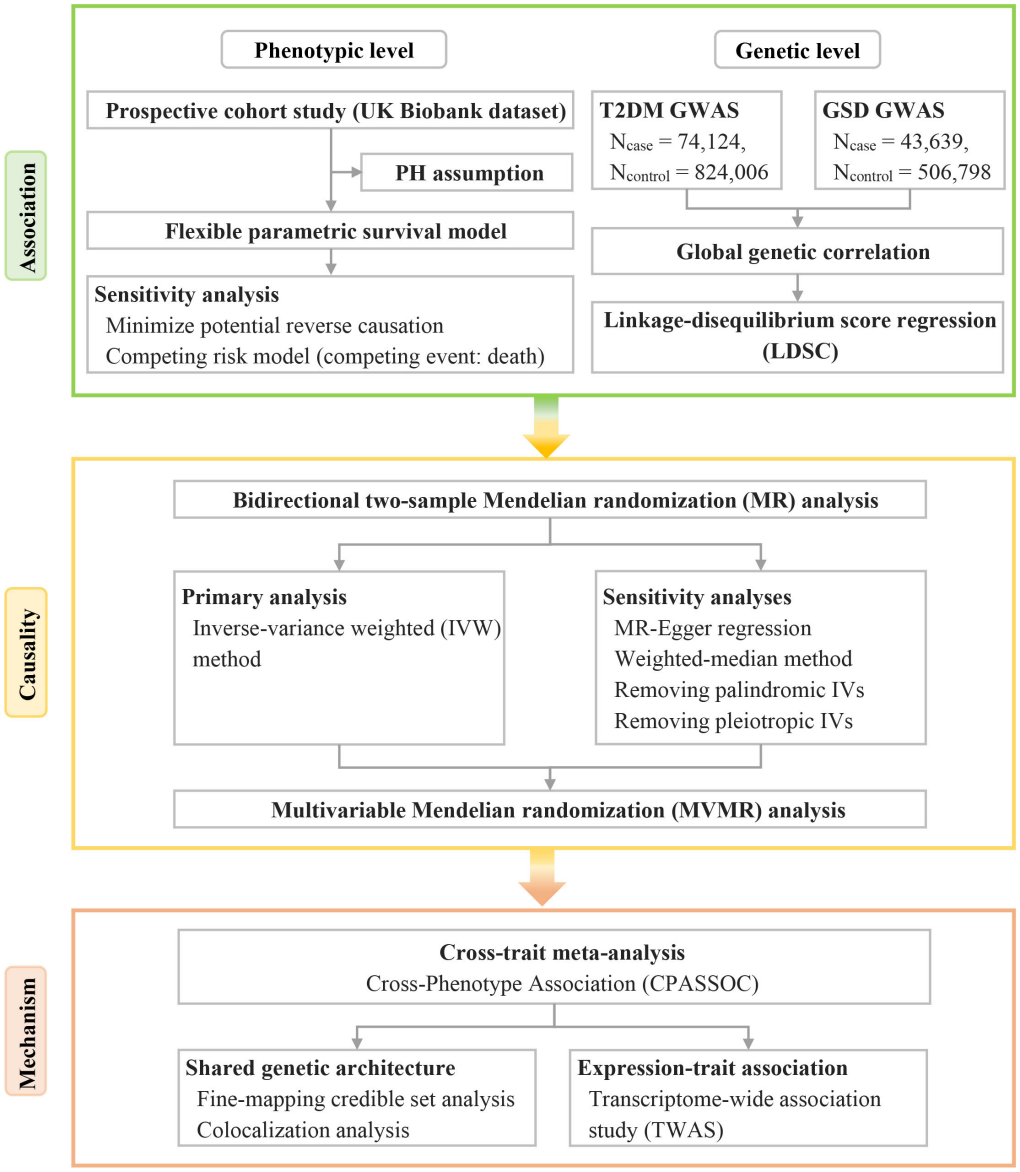
Overview of the study design, including the analysis methods in each phase. GSD, gallstone disease; GWAS, genome-wide association study; IVs, instrumental variables; PH, proportional hazards; T2DM, type 2 diabetes mellitus.

Therefore, we conducted a comprehensive bidirectional analysis to investigate the phenotypic and genetic associations between T2DM and GSD. Furthermore, we sought to identify the shared genetic architecture between T2DM and GSD in order to elucidate the underlying biological mechanisms. The study design is outlined in **Figure 1**.

## Methods

### Data sources

Our observational analyses used data from UK Biobank (UKB). UK Biobank (UKB) is a large prospective cohort study that enrolled over 500,000 participants aged 40-69 years from England, Wales, and Scotland between 2006 and 2010. The study was approved by the National Health Service North West Multi-Centre Research Ethics Committee and all participants provided written informed consent. The diagnoses of T2DM and GSD were based on the International Classification of Diseases, Tenth Revision (ICD-10) and Ninth Revision (ICD-9), as well as UK-Biobank-specific codes, which are available in **Supplementary Table S1**. We included only 472,050 white participants and excluded those with a history of events at baseline. Participants who self-reported a history of T2DM/GSD at study enrolment (self-reported non-cancer illness, Data-Field 20002), but did not have an ICD-10 or ICD-9 code for T2DM/GSD were also excluded. The participant selection flow chart is presented in **Supplementary Figure S1**.

We performed genetic analyses using meta-analysis of GWAS summary data. As instrumental variables (IVs), we selected independent genome-wide significant SNPs (P < 5×10^-8^). In the European ancestry GWAS of T2DM, which included 898,130 participants (N_case_=74,124, N_control_=824,006), we identified 344 T2DM-associated index SNPs (21). Additionally, we incorporated 144 index SNPs associated with T2DM adjusted for BMI (T2DM_adj_BMI) from a GWAS involving 574,306 participants (N_case_=50,409, N_control_=523,897). The genotype data were imputed using the Haplotype Reference Consortium (HRC) reference panel. SNPs with a low imputation quality (INFO <0.4) were excluded. Additionally, SNPs with a minor allele frequency (MAF) < 0.05 were further filtered out. For GSD, the GWAS involving 550,437 European participants (N_case_=43,639, N_control_=506,798) identified 62 GSD-associated index SNPs (20). Data were imputed by the HRC.r1-1 reference panel for UBKK and the population-specific SISu v3 imputation reference panel for FinnGen. Subsequently, data were filtered by INFO>0.30, MAF>0.001.

We also collected the largest GWAS data available for BMI (23), Waist-to-hip (WHR) (23), WHR_adj_BMI (23), fasting insulin (FI) (24), FI_adj_BMI (25), total cholesterol (TC) (26), triglycerides (TG) (26), high density lipoprotein (HDL) (26), low density lipoprotein (LDL) (26), lipoprotein A (LPA) (27), apolipoprotein A (ApoA) (27), apolipoprotein B (ApoB) (27), smoking (28), and alcohol intake (28) to conduct our MVMR. All these GWAS data were imputed using the reference panel (e.g., HRC) and filtered based on INFO>(0.3~0.5) and MAF>0.01 or 0.001. **Supplementary Table S2** provide further details on these GWAS summary data.

### Statistical analysis

#### Survival analysis

To investigate the observational association between T2DM and GSD, we first assessed the proportional hazards (PH) assumption using *stphtest* in Stata. We then employed flexible parametric survival models (FPSMs) and reported the results as hazard ratios (HRs) with 95% confidence intervals (CIs). This approach allows us to account for time-dependent effects (29). We utilized restricted cubic splines (with four degrees of freedom) in implementation of FPSMs using *stpm2* in Stata. The determination of the optimal number of knots and model was based on minimizing the Akaike’s information criterion and Bayesian information criterion (29). Covariates were selected based on existing literature and model selection.

The full model was ultimately implemented using potential confounders extracted from baseline questionnaires, including sex, age, assessment centre, the top 40 genetic principal components, BMI, Townsend deprivation index (TDI), education, smoking, alcohol intake, sleep duration, time spent watching television, physical activity (IPAQ), family history of T2DM, diastolic blood pressure (DBP), systolic blood pressure (SBP), TC, LDL and TG.

To ensure the reliability of our findings, we conducted two sensitivity analyses: 1) excluding participants who experienced events within the first two years after being diagnosed with exposure, to minimize potential reverse causation; and 2) using a flexible parametric competing risk regression model (30), with death as the competing event, to rectify overestimation of the probability for the event of interest occurring over time. All *P* values were two-sided, with statistical significance set at *P*<0.05. Survival analyses were performed using Stata version 13 (Stata Corp., College Station, TX).

#### Genome-wide genetic correlation analysis

Genetic correlation represents the average sharing of genetic effect between two traits that is not influenced by environmental confounders. We used linkage-disequilibrium score regression (LDSC) (31) to estimate the global genetic correlation (*r_g_*) between T2DM and GSD using GWAS summary data. We utilized pre-calculated HapMap3 LD scores computed from ~1.2 million common SNPs in European ancestry, commonly acknowledged as well-imputed. A Bonferroni-corrected P-value (0.025 = 0.05/2) was used to define statistical significance.

#### Mendelian randomization analysis

We conducted MR analyses to investigate the causal relationship between T2DM and GSD. Initially, we performed a bidirectional two-sample MR using the *TwoSampleMR* (https://mrcieu.github.io/TwoSampleMR/). The inverse-variance weighted (IVW) method was utilized as primary analysis (32). To ensure the robustness of our findings, we also performed sensitivity analyses by: 1) using MR-Egger regression (33); 2) using the weighted-median method (34); 3) removing palindromic IVs; and 4) removing pleiotropic IVs. Additionally, we assessed the relevance and exclusion restriction assumption by examining the R^2^ values and *F*-statistics (>10) (35) of our IVs and by detecting MR-Egger intercept. A Bonferroni-corrected P-value (0.025 = 0.05/2) was employed.

To further validate the causal relationship identified in the univariable MR analysis, we conducted MVMR and considered potential confounders such as adult BMI, WHR, FI, TC, TG, HDL, LDL, LPA, ApoA, ApoB, smoking, and alcohol intake based on a review of existing literature.

#### Cross-trait meta-analysis

To identify genetic variants with pleiotropic effects, we performed a cross-trait meta-analysis using the Cross-Phenotype Association (CPASSOC) analysis approach (17) with GWAS summary data. The pairwise S_Het_ was used to combine summary statistics as it can maintain statistical power when heterogeneity exists (17). We then obtained independent shared variants using the software PLINK (https://www.cog-genomics.org/plink/1.9/) with parameters (–clump-p1 5e-8 –clump-p2 1e-5 –clump-r2 0.2 –clump-kb 500). The significant index SNP was determined as the variant with *P*_single-trait_<1×10^-5^ (both traits) and *P*_CPASSOC_<5×10^-8^. We mapped the nearest gene of these shared SNPs identified by CPASSOC using Ensemble Variant Effect Predictor (https://grch37.ensembl.org/info/docs/tools/vep/index.html).

#### Fine-mapping credible set and colocalization analysis

To investigate the causal SNP for these index SNPs identified from CPASSOC, we performed a fine-mapping (FM) analysis using Bayesian FM method (36) to estimate credible sets of SNPs that had a 99% likelihood of containing the causal SNPs. We estimated a posterior inclusion probability (PIP) for each index SNP using the steepest descent approximation.

We further conducted colocalization analysis to determine whether shared SNPs identified by CPASSOC were shared causal variant or distinct causal variants using a Bayesian method, Coloc (37). A locus was considered colocalized if the posterior probability for H4 (PPH4) exceeded 0.7.

#### Transcriptome-wide association study analysis

We conducted a transcriptome-wide association study (TWAS) analysis using FUSION (38) to investigate specific tissue-gene pairs shared by T2DM and GSD. Firstly, we performed single-trait TWAS using the expression weights from 49 post-mortem Genotype-Tissue Expression project tissues. Subsequently, we combined the single-trait TWAS result and determined the shared gene-tissue pairs across T2DM and GSD. Bonferroni correction was used to identify expression-trait associations.

## Results

### Phenotypic association

The baseline characteristics of UKB participants included in the observational analysis are presented in **Supplementary Tables S3** and **S4**. In terms of the impact of T2DM on GSD risk, 455,405 participants were followed for 5,306,344 person-years (11.7 ± 2.7 years), during which 1,553 participants with T2DM and 14,425 T2DM-free participants developed GSD. The PH assumption test indicated that T2DM and certain covariates, such as BMI, education, and alcohol intake, exhibited a time-dependent effect on GSD risk. The FPSM analysis revealed that participants with T2DM had a higher risk of GSD (HR=1.71, 95%CI=1.58-1.84, *P*<0.001), which decreased over time when adjusting for sex, age, assessment center, and the top 40 genetic principal components. Additional adjustment for confounders had minimal impact on the hazard of GSD in the final model (HR=1.39, 95% CI=1.29-1.50, *P*<0.001). Furthermore, the association between T2DM and GSD remained consistent when considering death as a competing event (HR=1.23, 95% CI=1.14-1.32, *P*<0.001), or when excluding participants who developed GSD within the first two years after T2DM diagnosis (HR=1.17, 95% CI=1.08-1.28, *P*<0.001) (**Table 1**).

**Table 1:** Phenotypic association between type 2 diabetes mellitus and gallstone disease.

Exposures and events	No. of cases	Person-years	Primary analysis HR (95%CI)			Sensitivity analysis HR (95%CI)	
			Model 1	Model 2	Model 3	Model 4	Model 5
T2DM → GSD							
No	14,425	5,093,394.66	Reference	Reference	Reference	Reference	Reference
Yes	1,553	212,949.57	1.71 (1.58, 1.84)	1.45 (1.36, 1.56)	1.39 (1.29, 1.50)	1.23 (1.14, 1.32)	1.17 (1.08, 1.28)
P value	─	─	<0.001	<0.001	<0.001	<0.001	<0.001
* P*_interaction_	─	─	<0.001	<0.001	<0.001	<0.001	<0.001
GSD → T2DM							
No	22,952	5,146,631.69	Reference	Reference	Reference	Reference	Reference
Yes	1,601	201,712.70	1.87 (1.78, 1.97)	1.24 (1.17, 1.32)	1.16 (1.08, 1.24)	1.21 (1.14, 1.28)	1.00 (0.93, 1.08)
* P* value	─	─	<0.001	<0.001	<0.001	<0.001	0.912

CI, confidence interval; GSD, gallstone disease; HR, hazard ratio; T2DM, type 2 diabetes mellitus; Time interaction, time-varying effect of type 2 diabetes mellitus interacting with survival time; No. of, the number of; P _interaction_, the P value of interaction of T2DM and time; Model 1 was adjusted for sex, age, assessment center, and the top 40 genetic principal components; Model 2 was further adjusted for body mass index, Townsend deprivation index, education, smoking, alcohol intake, sleep duration, time spent watching TV, and physical activity; Model 3 was further adjusted for family history of Type 2 diabetes mellitus, cholesterol, low density lipoprotein and triglycerides; Model 4 was flexible parametric competing risk regression model, and death as the competing event; Model 5 was excluded the participant whose event occurring in the first two years after diagnosis of exposure.

Regarding the impact of GSD on T2DM risk, 457,608 participants were followed for 5,348,344 person-years (11.7 ± 2.7 years). Among these participants, 1,601 participants with GSD and 22,952 GSD-free participants developed T2DM. The analysis showed participants with GSD had a higher risk of T2DM (HR=1.87, 95%CI=1.78-1.97, *P*<0.001) in model 1. However, the risk decreased significantly after further adjustment for potential confounders (HR=1.16, 95% CI=1.08-1.24, *P*<0.001) or when considering death as a competing event. Moreover, the association disappeared when participants who developed T2DM within the first two years after GSD diagnosis were excluded (HR=1.00, 95% CI=0.93-1.08, *P*=0.91) (**Table 1**).

### Genetic correlation

After Bonferroni correction, a positive overall genetic correlation was observed between T2DM and GSD (*r_g_* =0.35, *P*=7.71×10^-23^). The positive overall correlation remained significant even after removing the effect of BMI on T2DM (*r_g_* =0.22, *P*=4.48×10^-10^) (**Supplementary Table S5**).

### Mendelian randomization analysis

**Figure 2:**
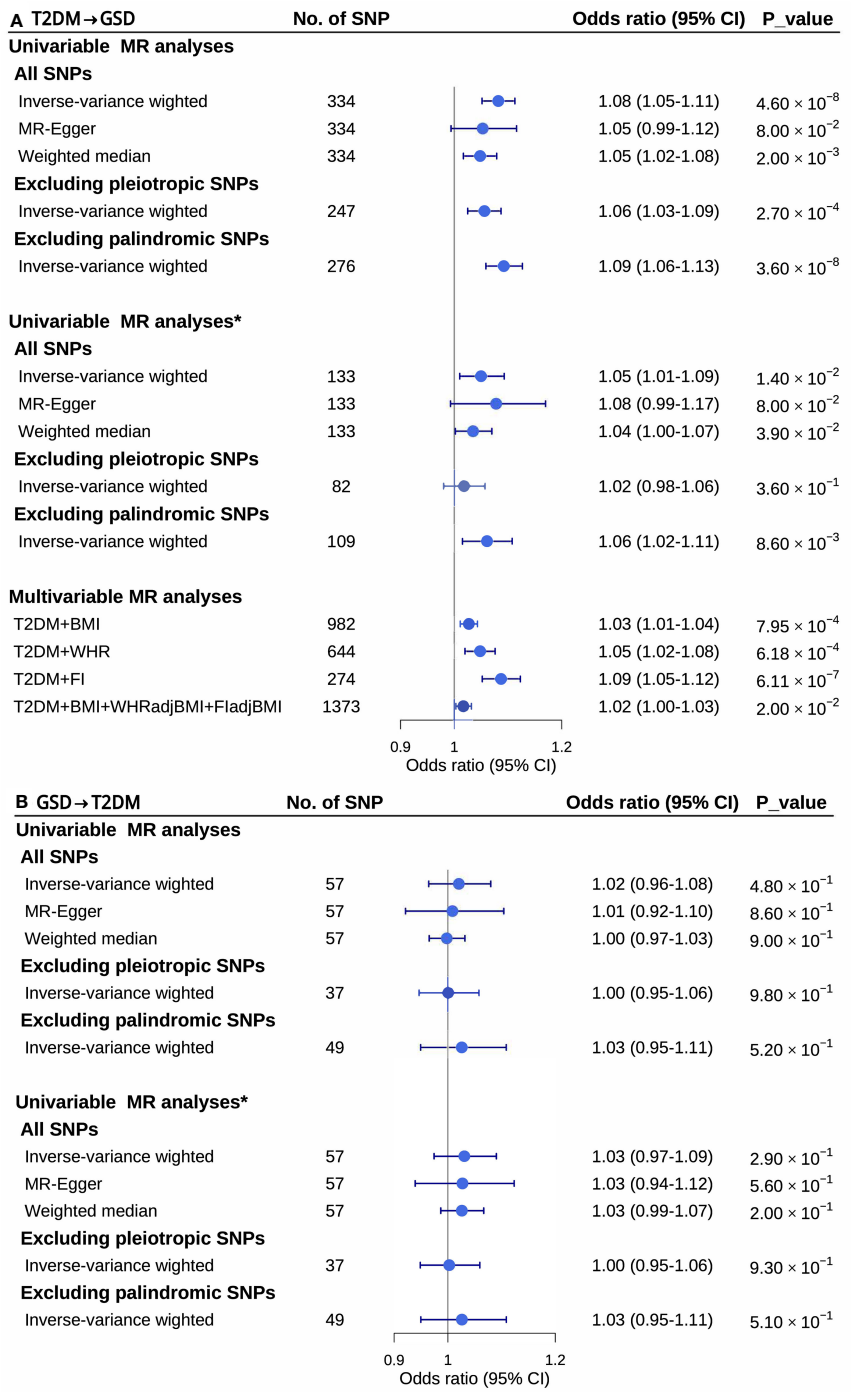
Univariable and multivariable mendelian randomization analysis between type 2 diabetes mellitus and gallstone disease. CI, confidence intervals; GSD, gallstone disease; MR, mendelian randomization; No. of, the number of; SNPs, single nucleotide polymorphisms; T2DM, type 2 diabetes mellitus; *, removing the effect of body mass index on type 2 diabetes mellitus.

The bidirectional two-sample MR analysis revealed a causal association between T2DM and GSD risk (**Figure 2**). Genetic liability to T2DM increased the risk of GSD (odds ratio (OR)=1.08, 95%CI=1.05-1.11, *P=*4.6 ×10^-8^, *P*_MR-Egger intercept=_0.29). However, no causal association was observed between genetic liability to GSD and T2DM risk (OR=1.02, 95%CI=0.96-1.08, *P*=0.48, *P*_MR-Egger intercept_= 0.74). Sensitivity analysis using the MR-Egger (T2DM→GSD: OR=1.05, 95%CI=0.99-1.12, *P*=0.08; GSD→T2DM: OR=1.01, 95%CI=0.92-1.10, *P*=0.86) and weighted median (T2DM→GSD: OR=1.05, 95%CI=1.02-1.08, *P*=0.002; GSD→T2DM: OR=1.00, 95%CI=0.97-1.03, *P*=0.9) estimator methods supported the unidirectional causal association. Removing palindromic (T2DM→GSD: OR =1.06, 95%CI=1.03-1.09, *P*=2.7×10^-4^; GSD→T2DM: OR=1.00, 95%CI=0.95-1.06, *P*=0.98) and pleiotropic (T2DM→GSD: OR=1.09, 95%CI=1.06-1.13, *P*=3.6×10^-8^; GSD→T2DM: OR=1.03, 95%CI=0.95-1.11, *P*=0.52) IVs also demonstrated similar causal effects. After removing the effect of BMI on T2DM, the causal associations remained almost unchanged (T2DM_adj_BMI→GSD: OR=1.05, 95%CI=1.01-1.09, *P*=0.014, *P*_MR-Egger intercept_=0.49; GSD→T2DM_adj_BMI: OR=1.03,95%CI=0.97-1.09, *P*=0.29, *P*_MR-Egger intercept_=0.92). Furthermore, even after adjusting for each potential confounder, T2DM still increased GSD risk (**Supplementary Table S6**). The final model of MVMR, which adjusting for BMI, WHR_adj_BMI and FI_adj_BMI, showed a slightly attenuated yet significant effect size compared to the univariable MR (OR=1.02, 95%CI=1.00-1.03, *P*=0.02) (**Figure 2**, **Supplementary Table S7**).

### Genetic architectures shared by T2DM and GSD

To identify shared genetic architectures and to elucidate the underlying biological mechanisms, we performed cross-trait meta-analysis, fine-mapping analysis and colocalization analysis (**Table 2** and **Supplementary Tables S8-S15**). Cross-trait meta-analysis identified 23 pleiotropic SNPs for T2DM and GSD. The most significant pleiotropic SNP was rs11075985 (*P*_CPASSOC_=3.35×10^-83^) located near *FTO*, followed by rs1800961 (*P*_CPASSOC_=5.69×10^-58^) and rs1260326 (*P*_CPASSOC_=1.00×10^-46^), located near *HNF4A* and *GCKR*, respectively.

**Table 2:** The cross-trait meta-analysis, fine-mapping analysis and colocalization analysis for type 2 diabetes mellitus and gallstone disease‡.

SNPs	A1	A2	BETA		*P*_value			Genomic coordinates	Mapped genes	99% credible set		Colocalization analysis
			T2DM	GSD	T2DM	GSD	CPA			No. of SNPs	CumSum	PP H_4_
T2DM and GSD												
rs10882889	G	A	-0.04	0.03	5.92×10^-8^	4.30×10^-6^	1.36×10^-11^	chr10:98950477-99290349	*ARHGAP19-SLIT1, ARHGAP19*	78	0.991	0.776
rs11075985	A	C	0.04	0.12	3.92×10^-6^	1.00×10^-74^	3.35×10^-83^	chr16:53797565-53848561	*FTO*	63	1.000	0.912
rs11244061	T	C	0.06	0.06	1.60×10^-7^	1.90×10^-8^	5.27×10^-14^	chr9:136153981-136339755	*ABO*	1	0.996	0.997
rs1169288	C	A	-0.08	-0.05	5.84×10^-23^	7.30×10^-13^	5.75×10^-30^	chr12:121189116-121485336	*HNF1A, HNF1A-AS1*	6	0.992	0.000
rs1169307	C	T	-0.06	-0.04	1.63×10^-13^	4.50×10^-10^	3.19×10^-20^	chr12:121352974-121483489	*HNF1A, C12orf43, RP11-216P16.2*	3	0.996	0.000
rs1260326	C	T	0.08	-0.07	1.22×10^-23^	1.30×10^-24^	1.00×10^-46^	chr2:27548038-28113911	*GCKR*	1	1.000	1.000
rs13029250	T	G	0.07	-0.03	9.00×10^-22^	4.50×10^-7^	2.22×10^-22^	chr2:43501802-43808065	*THADA, RNU6-958P*	1	1.000	0.000
rs1800961	T	C	0.29	0.16	4.48×10^-50^	3.20×10^-20^	5.69×10^-58^	chr20:42958768-43042364	*HNF4A*	1	1.000	1.000
rs2523504	C	T	0.04	-0.05	6.09×10^-6^	6.10×10^-14^	9.66×10^-19^	chr6:31150435-31555130	*ATP6V1G2, NFKBIL1, DDX39B, ATP6V1G2-DDX39B, DDX39B-AS1, SNORD84, SNORD83, DASS-161H22.6*	7	0.992	0.982
rs2857609	G	A	0.06	-0.07	9.15×10^-7^	9.30×10^-17^	1.36×10^-22^	chr6:31114449-32071893	*UQCRHP1, BX511262.2*	10	0.992	0.975
rs28929474	T	C	0.33	-0.11	1.11×10^-39^	5.90×10^-6^	1.32×10^-41^	chr14:94672731-94877868	*SERPINA1*	1	1.000	0.992
rs3130279	G	A	0.06	-0.07	4.81×10^-7^	1.90×10^-15^	2.84×10^-21^	chr6:32029226-32602482	*PRRT1*	14	0.991	0.002
rs35134156	G	A	0.04	-0.04	4.05×10^-6^	4.10×10^-10^	5.53×10^-15^	chr15:76953256-77324880	*PSTPIP1*	4	0.998	0.003
rs362307	T	C	0.06	0.07	6.17×10^-6^	1.10×10^-9^	3.47×10^-14^	chr4:2935618-3437308	*HTT, MSANTD1*	2	1.000	0.889
rs429358	C	T	-0.06	0.08	1.02×10^-7^	1.80×10^-18^	3.02×10^-25^	chr19:45387459-45428234	*APOE, TOMM40*	1	1.000	0.879
rs519790	G	C	0.04	-0.04	4.05×10^-6^	3.80×10-8	1.31×10^-12^	chr11:72411664-72894273	*STARD10, ARAP1*	7	0.990	0.000
rs58304657	C	G	-0.07	0.07	7.92×10^-9^	1.40×10^-11^	3.20×10^-19^	chr19:46148237-46376217	*GIPR, MIR642A*	4	0.990	0.000
rs62052815	T	C	-0.04	-0.04	5.63×10^-8^	6.50×10^-10^	3.51×10^-16^	chr16:69545116-69976089	—	19	0.999	0.902
rs72870502	T	C	0.07	-0.04	1.40×10^-10^	2.50×10^-7^	1.35×10^-14^	chr2:43920357-43933042	*PLEKHH2*	1	1.000	0.000
rs738408	T	C	-0.05	0.05	1.75×10^-7^	1.80×10^-10^	1.25×10^-16^	chr22:44324727-44395451	*PNPLA3*	4	0.999	0.990
rs7461273	G	C	-0.04	-0.04	6.92×10^-7^	9.60×10^-8^	1.20×10^-12^	chr8:11517977-11894039	*OR7E158P*	110	0.993	0.000
rs76747430	G	A	-0.06	0.04	3.40×10^-9^	2.30×10^-6^	1.19×10^-12^	chr22:40530052-41262852	*MKL1, RP5-1042K10.12*	4	0.994	0.014
rs879882	C	T	0.04	-0.05	4.14×10^-6^	4.00×10^-13^	3.88×10^-18^	chr6:31005726-31319157	*POU5F1, PSORS1C3, TCF19, CR847794.1, CR759815.2*	26	0.992	0.986
T2DM_adj_BMI and GSD												
rs1169288	C	A	-0.08	-0.06	5.84×10^-23^	6.80×10^-12^	5.32×10^-31^	chr12:121353088-121485336	*HNF1A, HNF1A-AS1*	1	0.992	0.000
rs1169307	C	T	-0.06	-0.04	1.63×10^-13^	3.90×10^-8^	1.61×10^-18^	chr12:121197124-121489657	*HNF1A, C12orf43, RP11-216P16.2*	3	0.998	0.000
rs1260326	C	T	0.08	-0.07	1.22×10^-23^	7.40×10^-19^	6.81×10^-40^	chr2:27548038-28113911	*GCKR*	1	0.998	0.998
rs149797*	T	C	-0.04	0.04	2.20×10^-7^	5.70×10^-6^	3.52×10^-11^	chr5:72071072-72406659	—	84	0.990	0.422
rs1800961	T	C	0.29	0.18	4.48×10^-50^	9.20×10^-18^	1.44×10^-59^	chr20:42958768-43042364	*HNF4A*	1	1.000	1.000
rs2239525	A	G	0.04	0.06	4.81×10^-6^	3.90×10^-12^	7.54×10^-16^	chr6:31437872-31555130	*ATP6V1G2, DDX39B, ATP6V1G2-DDX39B, DDX39B-AS1, SNORD84, SNORD83, DASS-161H22.6*	9	0.992	0.640
rs244418	A	G	-0.04	-0.04	1.04×10^-6^	1.60×10^-7^	2.94×10^-12^	chr16:69545116-69968892	*NFAT5*	42	0.990	0.834
rs2857609	G	A	0.06	-0.07	9.15×10^-7^	1.10×10^-11^	5.78×10^-17^	chr6:31114449-32071893	*UQCRHP1, BX511262.2*	12	0.990	0.557
rs3130279	G	A	0.06	-0.07	4.81×10^-7^	1.30×10^-11^	6.04×10^-16^	chr6:32080146-32602482	*PRRT1*	26	0.990	0.001
rs519790	G	C	0.04	-0.04	4.05×10^-6^	1.30×10^-7^	6.60×10^-12^	chr11:72411664-72894273	*STARD10, ARAP1*	15	0.991	0.000
rs56094641	G	A	0.04	-0.05	4.73×10-^6^	1.30×10^-12^	1.88×10^-16^	chr16:53797908-53845487	*FTO*	44	0.990	0.912
rs58304657	C	G	-0.07	0.11	7.92×10^-9^	7.50×10^-19^	2.50×10^-26^	chr19:46148237-46376217	*GIPR, MIR642A*	3	0.999	0.000
rs736820	A	G	-0.04	-0.04	4.41×10^-8^	9.70×10^-6^	1.25×10^-11^	chr20:43034016-43036649	*HNF4A, MIR3646*	2	1.000	1.000
rs738408	T	C	-0.05	0.06	1.75×10^-7^	3.00×10^-12^	1.00×10^-17^	chr22:44324727-44395451	*PNPLA3*	4	0.996	0.990
rs879882	C	T	0.04	-0.05	4.14×10^-6^	1.50×10^-11^	1.22×10^-15^	chr6:31005726-31319157	*POU5F1, PSORS1C3, TCF19, CR847794.1, CR759815.2*	9	0.990	0.954

CPA, Cross-Phenotype Association; CumSum, cumulative sum of posterior inclusion probability; GSD, gallstone disease; SNPs, single nucleotide Polymorphisms; T2DM, type 2 diabetes mellitus; T2DM_adj_BMI, removing the effect of body mass index on type 2 diabetes mellitus; ‡, PCPASSOC < 5×10-8, single trait P-value < 1×10-5, clumping r2 = 0.2; *, a novel SNP (A significant index SNP met the following criteria was identified as novel index SNP: 1) did not reach genome-wide significance (5×10^-8^ < P_single-trait_ < 1×10^-5^) in single-trait GWAS; 2) was not in LD (r^2^ < 0.5) with any previously reported genome-wide significant SNPs in single-trait GWAS, and none of their neighboring SNPs (± 500 kb) reached P < 5×10^-8^ in single-trait GWAS.).

To investigate the causal SNP for these 23 pleiotropic SNPs, we estimated the 99% credible sets of causal SNPs. A total of 368 potential causal SNPs were obtained from the 23 pleiotropic SNPs. Note that the 99% credible set of rs1800961, rs1260326 and five other pleiotropic SNPs only included themselves. To further distinguish the shared causal SNPs from the distinct ones, we assessed statistical colocalization of these 23 pleiotropic SNPs. The results revealed that 56.2% (containing rs1800961, rs1260326 and 11 other pleiotropic SNPs) of shared loci colocalized at the same candidate causal SNPs. In summary, we found a good number of loci shared by T2DM and GSD, and in particular identified rs1800961 and rs1260326 as potential shared causal variant for T2DM and GSD.

After removing the effect of BMI on T2DM, we identified five additional pleiotropic SNPs, in addition to the ten pleiotropic SNPs also shared by T2DM and GSD. Notably, rs1800961 (*P*_CPASSOC_=1.44×10^-59^) and rs1260326 (*P*_CPASSOC_=6.81×10^-40^) were also identified as the most significant pleiotropic SNPs and shared causal SNPs for T2DM and GSD even after adjusting for BMI.

### Transcriptome-wide association study

Regarding gene expressions and biological insights, we used TWAS to explore the tissue-gene pairs shared by both diseases (**Table 3**, **Supplementary Table S16**). A total of 31 tissue-gene pairs were observed for T2DM and GSD, including 16 genes (*DMWD, GPN1, GTF3C2, IFT172, KRTCAP3, LINC01126, LINC01460, NRBP1, OASL, P2RX4, PPM1G, RBKS, SPPL3, SNX17, THADA, UNC119B*), mainly expressed in multiple tissues from nervous and motor systems. After removing the effect of BMI on T2DM, 26 out of the 31 (83.9%) tissue-gene pairs and 13 out of 16 (81.3%) genes remained significant.

**Table 3:** Shared transcriptome-wide association study significant genes between type 2 diabetes mellitus and gallstone disease.

Gene	Tissue	Gene stable IDs with version	CHR	GSD		T2DM	
				BEST.GWAS.ID	*P*-value^*^	BEST.GWAS.ID	*P*-value^*^
*NRBP1*	Artery_Tibial	ENSG00000115216.13	2	rs1260326	1.33×10^-2^	rs1260326	1.61×10^-5^
*LINC01126*	Artery_Tibial	ENSG00000279873.2	2	rs13029250	9.93×10^-14^	rs17334919	3.30×10^-2^
*IFT172*	Adipose_Subcutaneous	ENSG00000235267.1	2	rs1260326	2.15×10^-2^	rs1260326	9.47×10^-7^
*GTF3C2*	Adrenal_Gland	ENSG00000234072.1	2	rs1260326	2.15×10^-2^	rs1260326	9.47×10^-7^
*UNC119B*	Brain_Anterior_cingulate_cortex_BA24	ENSG00000175970.10	12	rs2393791	1.10×10^-12^	rs1169302	3.56×10^-3^
*SNX17*	Brain_Caudate_basal_ganglia	ENSG00000115234.10	2	rs1260326	2.15×10^-2^	rs1260326	9.47×10^-7^
*THADA*	Brain_Frontal_Cortex_BA9	ENSG00000115970.18	2	rs4299376	4.56×10^-8^	rs17334919	8.40×10^-14^
*KRTCAP3*	Brain_Nucleus_accumbens_basal_ganglia	ENSG00000157992.12	2	rs1260326	2.44×10^-4^	rs1260326	2.68×10^-4^
*LINC01460*	Brain_Spinal_cord_cervical_c-1	ENSG00000205334.2	2	rs1260326	3.82×10^-7^	rs1260326	1.81×10^-6^
*NRBP1*	Cells_Cultured_fibroblasts	ENSG00000115216.13	2	rs1260326	1.73×10^-2^	rs1260326	3.56×10^-7^
*SNX17*	Cells_Cultured_fibroblasts	ENSG00000115234.10	2	rs1260326	1.17×10^-2^	rs1260326	3.86×10^-5^
*GTF3C2*	Cells_Cultured_fibroblasts	ENSG00000234072.1	2	rs1260326	2.15×10^-2^	rs1260326	9.47×10^-7^
*NRBP1*	Cells_EBV-transformed_lymphocytes	ENSG00000115216.13	2	rs1260326	4.77×10^-2^	rs1260326	8.20×10^-4^
*GTF3C2*	Colon_Sigmoid	ENSG00000234072.1	2	rs1260326	1.60×10^-2^	rs1260326	6.21×10^-7^
*NRBP1*	Esophagus_Gastroesophageal_Junction	ENSG00000115216.13	2	rs1260326	9.93×10^-3^	rs1260326	6.65×10^-4^
*NRBP1*	Heart_Atrial_Appendage	ENSG00000115216.13	2	rs1260326	4.33×10^-2^	rs1260326	2.96×10^-5^
*P2RX4*	Liver	ENSG00000135124.14	12	rs2393791	4.62×10^-2^	rs1169302	5.82×10^-5^
*P2RX4*	Minor_Salivary_Gland	ENSG00000135124.14	12	rs2393791	4.74×10^-2^	rs1169302	7.99×10^-4^
*PPM1G*	Muscle_Skeletal	ENSG00000115241.10	2	rs1260326	4.13×10^-2^	rs1260326	1.46×10^-6^
*KRTCAP3*	Muscle_Skeletal	ENSG00000157992.12	2	rs1260326	3.66×10^-2^	rs1260326	1.02×10^-2^
*GPN1*	Muscle_Skeletal	ENSG00000198522.13	2	rs1260326	5.03×10^-8^	rs1260326	1.88×10^-3^
*DMWD*	Nerve_Tibial	ENSG00000185800.11	19	rs34255979	6.03×10^-5^	rs10406431	1.85×10^-2^
*THADA*	Nerve_Tibial	ENSG00000234936.1	2	rs13029250	1.14×10^-4^	rs17334919	1.24×10^-12^
*IFT172*	Nerve_Tibial	ENSG00000235267.1	2	rs1260326	1.75×10^-2^	rs1260326	3.56×10^-7^
*THADA*	Pituitary	ENSG00000234936.1	2	rs13029250	1.13×10^-2^	rs17334919	1.54×10^-10^
*NRBP1*	Skin_Not_Sun_Exposed_Suprapubic	ENSG00000115216.13	2	rs1260326	1.64×10^-2^	rs1260326	3.50×10^-7^
*RBKS*	Skin_Not_Sun_Exposed_Suprapubic	ENSG00000171174.13	2	rs1260326	1.61×10^-7^	rs1260326	1.87×10^-4^
*NRBP1*	Skin_Sun_Exposed_Lower_leg	ENSG00000115216.13	2	rs1260326	1.75×10^-2^	rs1260326	3.56×10^-7^
*OASL*	Testis	ENSG00000135114.12	12	rs2393791	1.77×10^-5^	rs1169302	6.83×10^-4^
*P2RX4*	Testis	ENSG00000135124.14	12	rs2393791	6.11×10^-3^	rs1169302	6.33×10^-4^
*SPPL3*	Whole_Blood	ENSG00000157837.15	12	rs2393791	8.38×10^-8^	rs1169302	1.85×10^-8^

CHR, chromosome; GSD, gallstone disease; T2DM, type 2 diabetes mellitus; *, P_Bonferroni_.

## Discussion

To our knowledge, this study represents the most comprehensive research on the bidirectional relationships between T2DM and GSD, combining observational and genetic analysis. Our study identified a unidirectional causality running from T2DM to the risk of GSD. Furthermore, we identified specific genetic variants, such as rs1800961 (*HNF4A*) and rs1260326 (*GCKR*), that contribute to the biological links between T2DM and GSD. These findings advance our understanding of the complicated relationship underlying T2DM and GSD, providing important implications for preventing and treating these common diseases.

Expanding upon prior research, our study takes a comprehensive approach to investigate the association between T2DM and GSD risk through both observational and MR studies. Our findings, obtained through survival and MR analyses, consistently demonstrate a causal relationship between T2DM and GSD risk. These results align with the updated meta-analysis of prospective cohort studies (RR=1.49, 95%CI=1.09-2.03, *P*=0.012, **Supplementary Figure 2**), which pooled data from previous meta-analysis (7) and two recent large-scale studies (5, 8). While in line with the findings of three existing MR studies (12–14), our MR expands previous results in two critical aspects. First, we minimize the influence of reverse causality by applying a bidirectional design. Second, we limit the impact of confounders by adjusting for potential confounding factors using the MVMR. Consequently, we assert that a causal relationship exists between T2DM and GSD risk.

Regarding the association between GSD and T2DM risk, previous studies have reported conflicting results. Some prospective cohort studies (6, 9, 10) have shown a positive association, while one MR study fund no association (6). Our study used both survival and MR analyses and found no causal association between GSD and T2DM risk. The discrepancy between our study and previous cohort studies may be attributed to two aspects. Firstly, two of these previous cohort studies (9, 10) relied on self-reported data, which may have led to misclassification or underestimation of cases. Secondly, observational studies are prone to reverse causality, which the previous cohort study did not account for (6). However, after considering a 2-year lag time, our study found no association between GSD and T2DM. This implies that previous significant findings may have been distorted by reverse causality. MR studies are commonly employed to address reverse causality (11). Our MR analysis replicates the null findings of the previous MR study (6), yet with a much larger sample size (43,639 vs. 1,110 T2DM cases) and more IVs (62 vs. 8). Additionally, our study considered the significant contribution of BMI to T2DM development, which was not accounted for in the previous MR study.

Through cross-trait and colocalization analyses, we identified shared biological mechanisms. Specifically, the key SNPs associated with both conditions were rs1800961 mapped *HNF4A* and rs1260326 mapped *GCKR*. *HNF4A* regulates liver-specific gene expression involved in lipid transport, glucose, and bile metabolism (39). It is also essential for insulin secretion in pancreatic beta cells (40). Additionally, *HNF4A* has been linked to elevated levels of gamma-glutamyl transferase (GGT), a sensitive marker of cholestasis (41). The effects of *HNF4A* on insulin action and GGT contribute to the development of both T2DM and GSD. On the other hand, *GCKR* regulates glucose conversion to glucose-6-phosphate in the liver and pancreas (42, 43). It is associated with various metabolites involved in carbohydrate and lipid metabolism (42). The minor allele of *GCKR* has been associated with hepatospecific glucokinase activation, reduced plasma insulin levels, and protection against T2DM (42). Additionally, *GCKR* enhances hepatic cholesterol availability, leading to elevated bile cholesterol concentration and GSD development (43).

Our findings deliver important clinical and public health implications. First, our found that T2DM causally contributes to the development of GSD, but GSD does not increase T2DM risk. This suggests that preventing and treating GSD may not significantly reduce T2DM risk. However, our findings highlight the important of focusing on prevention and treatment of T2DM. Second, while new drugs for T2DM have been developed, further research is needed to explore novel treatments (44). It is noteworthy that certain medications commonly prescribed for T2DM have been associated with an increased risk of GSD in large meta-analyses of randomized controlled trials (45). Furthermore, our genetic analyses reveal shared genetic architectures between T2DM and GSD, which can enhance our understanding of biological mechanisms and potentially identify therapeutic targets for T2DM. For example, our findings suggest that *HNF4A* and *GCKR* may be promising targets for T2DM therapies (42), but their effects on GSD and other comorbidities should be considered.

Several limitations should be considered when interpreting the results and conclusions of this study. Firstly, fasting insulin may confound the association between T2DM and GSD risk, which we were unable to adjust for in our multivariable survival analysis due to a lack of information from prospective cohort. However, after adjusting for FI in our MVMR, we confirmed the causal relationship between T2DM and GSD risk. Additionally, dietary behaviors such as egg, vegetable, and fruit intake may act as other confounders for the association between T2DM and GSD. Unfortunately, due to a larger proportion (>50%) of missing data in the prospective cohort, we were unable to adjust for these factors in our multivariable survival analysis. Furthermore, it is worth noting that half of the genetic variants associated with dietary behaviors are a consequence of increased BMI (46). Considering the potential collinearity between BMI and dietary behaviors, we chose not to include the latter in the MVMR analysis. Secondly, our findings were restricted to European population to control for population stratification. However, this may limit the generalizability of our results. Additionally, despite over 90% of gallstones in Europe are cholesterol gallstones, there are also pigment gallstones with different etiologies, which were not specifically investigated in our study.

## Conclusions

Our study demonstrates an association between T2DM and GSD risk at both the phenotypic and genetic levels using large-scale prospective cohort and GWAS data. This association is independent of BMI, WHR, and FI, suggesting an intrinsic and causal relationship. However, no causal association was found between GSD and T2DM risk. Furthermore, the shared genetic architecture between GSD and T2DM enhance our understanding of the underlying biological mechanisms. These findings might offer valuable insights for the identification of potential therapeutic targets for T2DM and novel perspectives on preventing GSD, ultimately contributing to a decrease in multimorbidity incidence.

## Data availability statement

Publicly available datasets were analyzed in this study. This data can be found here: The UK Biobank analysis was conducted with the application 50538. GWAS summary statistics for type 2 diabetes mellitus are publicly available through the DIAGRAM consortium (http://diagram-consortium.org/downloads.html). GWAS summary statistics for gallstone disease were applied by the corresponding authors.

## Ethics statement

Ethical approval was not required for the study involving humans in accordance with the local legislation and institutional requirements. Written informed consent to participate in this study was not required from the participants or the participants’ legal guardians/next of kin in accordance with the national legislation and the institutional requirements.

## Author contributions

PY: Conceptualization, Data curation, Formal Analysis, Methodology, Visualization, Writing – original draft, Writing – review & editing. LZ (2^nd^ author): Conceptualization, Data curation, Formal Analysis, Methodology, Visualization, Writing – original draft, Writing – review & editing. CY: Conceptualization, Data curation, Formal Analysis, Methodology, Visualization, Writing – original draft, Writing – review & editing. WZ: Data curation, Writing – review & editing. YW: Data curation, Writing – review & editing. MZ: Data curation, Writing – review & editing. HC: Data curation, Writing – review & editing. MT: Data curation, Writing – review & editing. LC: Data curation, Writing – review & editing. XW: Data curation, Writing – review & editing. XZ: Data curation, Writing – review & editing. YZ: Data curation, Writing – review & editing. JX: Data curation, Writing – review & editing. YL: Data curation, Writing – review & editing. CX: Data curation, Writing – review & editing. YFY: Methodology, Writing – review & editing. LZ (17^th^ author): Methodology, Writing – review & editing. YQY: Methodology, Writing – review & editing. JL: Methodology, Writing – review & editing. ZL: Methodology, Writing – review & editing. CXY: Methodology, Writing – review & editing. XJ: Conceptualization, Methodology, Supervision, Writing – original draft, Writing – review & editing. BZ: Conceptualization, Funding acquisition, Methodology, Supervision, Writing – review & editing.
