# Supplementary material for: Observational and genetic analyses clarify the relationship between type 2 diabetes mellitus and gallstone disease
Source: Front Endocrinol (Lausanne). 2024 Jan 31;14:1337071. doi: 10.3389/fendo.2023.1337071 (PMC10864641; doi:10.3389/fendo.2023.1337071)

Supporting information for

**Observational and genetic analyses clarify the relationship between type 2 diabetes mellitus and gallstone disease**

*Peijing Yan, Li Zhang, Chao Yang, Wenqiang Zhang, Yutong Wang, Min Zhang, Huijie Cui, Mingshuang Tang, Lin Chen, Xueyao Wu, Xunying Zhao, Yanqiu Zou, Jinyu Xiao, Yunjie Liu, Chenghan Xiao, Yanfang Yang, Ling Zhang, Yuqin Yao, Jiayuan Li, Zhenmi Liu, Chunxia Yang, Xia Jiang, Ben Zhang*

| **Contents** | **Page** |
| --- | --- |
| **Supplementary Tables** | **3-192** |
| Table S1. Disease Codes of type 2 diabetes mellitus and gallstone disease. | 3 |
| Table S2. Characteristics of genome-wide association study summary data. | 4 |
| Table S3. Baseline characteristics of UK Biobank participants by type 2 diabetes mellitus status during follow-up for gallstone disease. | 6 |
| Table S5. Genome-wide genetic correlation between type2 diabetes mellitus and gallstone disease. | 10 |
| Table S6. Effect estimates of multivariable Mendelian randomization analyses for one of covariates. | 11 |
| Table S7. Effect estimates of multivariable Mendelian randomization analyses. | 12 |
| Table S8. Pleiotropic SNPs identified by cross-trait meta-analysis between type 2 diabetes mellitus and gallstone disease‡. | 13 |
| Table S9. Pleiotropic SNPs identified by cross-trait meta-analysis between type 2 diabetes mellitus (adjusted for BMI) and gallstone disease‡. | 16 |
| Table S10. Linear closest genes of each CPASSOC-identified SNPs for type 2 diabetes mellitus and gallstone disease. | 19 |
| Table S11. Linear closest genes of each CPASSOC-identified SNPs for type 2 diabetes mellitus (adjusted for BMI) and gallstone disease. | 103 |
| Table S12. List of SNPs in the 99% credible set identified from fine-mapping analysis for each CPASSOC-identified locus shared between type 2 diabetes mellitus and gallstone disease. | 174 |
| Table S13. List of SNPs in the 99% credible set identified from fine-mapping analysis for each CPASSOC-identified locus shared between type 2 diabetes mellitus (adjusted for BMI) and gallstone disease. | 182 |
| Table S14. Results of colocalization analysis for each pleiotropic locus identified from CPASSOC between type 2 diabetes mellitus and gallstone disease‡. | 188 |
| Table S15. Results of colocalization analysis for each pleiotropic locus identified from CPASSOC between type 2 diabetes mellitus (adjusted for BMI) and gallstone disease‡. | 190 |
| Table S16. Shared transcriptome-wide association study significant genes between type 2 diabetes mellitus and gallstone disease. | 191 |
| **Supplementary Figures** | **193-194** |
| Figure S1. The participant selection flow chart. | 193 |
| Figure S2. Undated meta-analysis of relationship between the type 2 diabetes mellitus and gallstone disease risk. | 194 |

## Table S1. Disease Codes of type 2 diabetes mellitus and gallstone disease.

| **Diagnosis** | **ICD-10** | **ICD-9** | **UKB Code**  **(data-field 20002, self-reported verified in medical notes)** |
| --- | --- | --- | --- |
| Type 2 diabetes mellitus | E11 | 25000, 25010,  25020, 25090 | 1223 |
| Gallstone disease | K80, K81, K851, K915, K9186, K563 | 574, 5750, 5751, 5760, 99741, 56031 | 1162 |

ICD-9, International Classification of Diseases, Ninth Revision; ICD-10, International Classification of Diseases, Tenth Revision; UKB, UK Biobank.

## Table S2. Characteristics of genome-wide association study summary data.

| **Traits** | **Data sources** | **PMID** | **No. of IV** | **Sample size** | **Cohorts** | **Ancestry** | **Variance (R^2^, %)** | **F statistic** |
| --- | --- | --- | --- | --- | --- | --- | --- | --- |
| GSD | Cameron J. Fairfield, 2022 | 34651315 | 62 | 550,437 | UK Biobank and FinnGen | European | 4.74 | 441.71 |
| T2DM | Mahajan, A., 2018 | 30297969 | 386 | 898,130 | 32 GWAS (https://static-content.springer.com/esm/art%3A10.1038%2Fs41588-018-0241-6/MediaObjects/41588_2018_241_MOESM3_ESM.xlsx) | European | 2.27 | 53.92 |
| T2DM_adj_BMI | Mahajan, A., 2018 | 30297969 | 143 | 574,306 | 32 GWAS (https://static-content.springer.com/esm/art%3A10.1038%2Fs41588-018-0241-6/MediaObjects/41588_2018_241_MOESM3_ESM.xlsx) | European | 1.96 | 80.46 |
| Adult BMI | Pulit, S. L., 2019 | 30239722 | 670 | 806,834 | Genetic Investigation of ANthropometric Traits (GIANT) consortium and UKB | European | 5.47 | 69.64 |
| WHR | Pulit, S. L., 2019 | 30239722 | 316 | 697,734 | Genetic Investigation of ANthropometric Traits (GIANT) consortium and UKB | European | 2.93 | 66.69 |
| WHRadjBMI | Pulit, S. L., 2019 | 30239722 | 346 | 694,649 | Genetic Investigation of ANthropometric Traits (GIANT) consortium and UKB | European | 3.76 | 78.46 |
| FI | Lagou, V., 2021 | 33402679 | 19 | 100,925 | 38 studies | European | 0.56 | 29.83 |
| FI_adj_BMI | Chen, J., 2021 | 34059833 | 43 | 151,010 | 13 European ancestry cohort | European | 1.38 | 49.16 |
| LDL | Graham, S. E., 2021 | 34887591 | 403 | 1,231,262 | 201 primary studies | European | 10.06 | 341.57 |
| HDL | Graham, S. E., 2021 | 34887591 | 380 | 1,244,544 | 201 primary studies | European | 7.87 | 279.56 |
| TG | Graham, S. E., 2021 | 34887591 | 388 | 1,253,236 | 201 primary studies | European | 7.23 | 251.47 |
| TC | Graham, S. E., 2021 | 34887591 | 429 | 1,319,982 | 201 primary studies | European | 8.62 | 290.32 |
| LPA | Sinnott-Armstrong, N., 2021 | 33462484 | 189 | 284,044 | UK Biobank | European | 59.95 | 2248.33 |
| ApoA | Sinnott-Armstrong, N., 2021 | 33462484 | 445 | 323,833 | UK Biobank | European | 16.88 | 147.55 |
| ApoB | Sinnott-Armstrong, N., 2021 | 33462484 | 593 | 354,097 | UK Biobank | European | 52.55 | 660.08 |
| Smoking Initiation | Liu, M., 2019 | 30643251 | 378 | 1,232,091 | 26 cohorts | European | 1.27 | 41.95 |
| Drinks per Week | Liu, M., 2019 | 30643251 | 99 | 941,280 | 26 cohorts | European | 0.66 | 63.16 |

ApoA, apolipoprotein A; ApoB, apolipoprotein B; BMI, body mass index, FI, fasting insulin; FI_adj_BMI, fasting insulin adjusted for BMI; GSD, gallstone disease; HDL, high density lipoprotein; IV: instrumental variable; LDL, low density lipoprotein; lpA, lipoprotein A; T2DM, type 2 diabetes mellitus; T2DM_adj_BMI, type 2 diabetes mellitus adjusted for BMI; TC, total cholesterol; TG, triglycerides; WHR, Waist-to-hip; WHR_adj_BMI, Waist-to-hip adjusted for BMI; F = (R^2^ (n-1-k))/((1-R^2^) k), n denotes the sample size, k denotes the number of IVs. R^2^ were estimated using minor allele frequency.

## Table S3. Baseline characteristics of UK Biobank participants by type 2 diabetes mellitus status during follow-up for gallstone disease.

| **Characteristics** | **Without T2DM  (n=424,060)** | **T2DM  (n=31,345)** | ***P*-value** |
| --- | --- | --- | --- |
| **Age (years), mean ± SD** | 56.41±8.06 | 60.17±6.89 | <0.001 |
| **Sex** |  |  | <0.001 |
| Female | 233,285(55.01) | 11,743(37.46) |  |
| Male | 190,775(44.99) | 19,602(62.54) |  |
| **BMI (kg/m2), mean ± SD** | 26.99±4.47 | 31.69±5.72 | <0.001 |
| **Assessment center** |  |  | <0.001 |
| England | 373,680(88.12) | 28,806(91.90) |  |
| Scotland | 32,105(7.57) | 1,438(4.59) |  |
| Wales | 18,275(4.31) | 1,101(3.51) |  |
| **Townsend deprivation index, median (IQR)** | -2.31(3.87) | -1.50(5.00) | <0.001 |
| **Education** |  |  | <0.001 |
| None | 67,517(16.18) | 9,329(30.52) |  |
| College or University degree | 139,851(33.51) | 6,093(19.93) |  |
| A levels/AS levels or equivalent | 48,441(11.61) | 2,779(9.09) |  |
| O levels/GCSEs or equivalent | 90,682(21.73) | 6,239(20.41) |  |
| CSEs or equivalent | 22,878(5.48) | 1,546(5.06) |  |
| NVQ or HND or HNC or equivalent | 26,798(6.42) | 2,804(9.17) |  |
| Other professional qualifications | 21,212(5.08) | 1,778(5.82) |  |
| **Smoking** |  |  | <0.001 |
| Never | 232,436(54.99) | 12,714(40.81) |  |
| Previous | 146,664(34.70) | 14,282(45.84) |  |
| Only occasionally | 11,434(2.71) | 819(2.63) |  |
| Most or all days | 32,134(7.60) | 3,339(10.72) |  |
| **Alcohol intake** |  |  | <0.001 |
| Daily or almost daily | 91,928(21.69) | 5,146(16.44) |  |
| Three or four times a week | 104,087(24.56) | 5,448(17.40) |  |
| Once or twice a week | 112,203(26.48) | 7,750(24.76) |  |
| One to three times a month | 46,428(10.96) | 3,867(12.35) |  |
| Special occasions only | 42,974(10.14) | 5,313(16.97) |  |
| Never | 26,157(6.17) | 3,782(12.08) |  |
| **Sleep duration (hours), mean ± SD** | 7.17±1.07 | 7.2±1.38 | <0.001 |
| **Time spent watching TV (hours/day), mean ± SD** | 2.73±1.61 | 3.56±2.00 | <0.001 |
| **IPAQ** |  |  | <0.001 |
| Low | 61,441(17.89) | 6,626(27.63) |  |
| Moderate | 140,457(40.90) | 9,448(39.40) |  |
| High | 141,555(41.22) | 7,906(32.97) |  |
| **Family history of diabetes** |  |  | <0.001 |
| None | 358,848(86.97) | 22,580(74.86) |  |
| Father/Mather | 51,399(12.46) | 6,976(23.13) |  |
| Both | 2,376(0.58) | 605(2.01) |  |
| **DBP (mmHg)** | 82.09±10.67 | 82.97±10.86 | <0.001 |
| **SBP (mmHg)** | 139.44±19.67 | 145.21±19.24 | <0.001 |
| **TC (mmol/L), mean ± SD** | 5.77±1.11 | 4.96±1.26 | <0.001 |
| **LDL (mmol/L), mean ± SD** | 3.61±0.85 | 3.05±0.94 | <0.001 |
| **Triglycerides (mmol/L), mean ± SD** | 1.7±0.99 | 2.3±1.29 | <0.001 |

BMI, Body mass index; DBP, Diastolic blood pressure, automated reading; GSD, gallstone disease; IQR, interquartile range; IPAQ, International Physical Activity Questionnaire; LDL, low density lipoprotein; SD, standard deviation; SBP, Systolic blood pressure, automated reading; T2DM, type 2 diabetes mellitus; TC, Cholesterol; TV, television.

## Table S4. Baseline Characteristics of UK Biobank participants by gallstone disease status during follow-up for type 2 diabetes mellitus.

| **Characteristics** | **Without GSD (n=432,587)** | **GSD**  **(n=25,021)** | ***P*-value** |
| --- | --- | --- | --- |
| **Age (years), mean ± SD** | 56.54±8.05 | 58.32±7.66 | <0.001 |
| **Sex** |  |  | <0.001 |
| Female | 232,689(53.79) | 17,216(68.81) |  |
| Male | 199,898(46.21) | 7,805(31.19) |  |
| **BMI (kg/m2), mean ± SD** | 27.16±4.62 | 29.37±5.26 | <0.001 |
| **Assessment center** |  |  | <0.001 |
| England | 382,064(88.32) | 21,675(86.63) |  |
| Scotland | 31,967(7.39) | 2,469(9.87) |  |
| Wales | 18,556(4.29) | 877(3.51) |  |
| **Townsend deprivation index, median (IQR)** | -2.29(3.91) | -2.02(4.29) | <0.001 |
| **Education** |  |  | <0.001 |
| None | 70,769(16.63) | 6,105(24.86) |  |
| College or University degree | 141,001(33.13) | 5,863(23.87) |  |
| A levels/AS levels or equivalent | 49,073(11.53) | 2,437(9.92) |  |
| O levels/GCSEs or equivalent | 92,044(21.63) | 5,533(22.53) |  |
| CSEs or equivalent | 23,209(5.45) | 1,379(5.61) |  |
| NVQ or HND or HNC or equivalent | 27,865(6.55) | 1,701(6.93) |  |
| Other professional qualifications | 21,655(5.09) | 1,542(6.28) |  |
| **Smoking** |  |  | <0.001 |
| Never | 234,553(54.40) | 12,854(51.63) |  |
| Previous | 151,424(35.12) | 9,355(37.57) |  |
| Only occasionally | 11,684(2.71) | 562(2.26) |  |
| Most or all days | 33,482(7.77) | 2,127(8.54) |  |
| **Alcohol intake** |  |  | <0.001 |
| Daily or almost daily | 93,740(21.68) | 3,455(13.82) |  |
| Three or four times a week | 105,556(24.42) | 4,569(18.28) |  |
| Once or twice a week | 114,096(26.39) | 6,572(26.29) |  |
| One to three times a month | 47,183(10.91) | 3,647(14.59) |  |
| Special occasions only | 44,424(10.28) | 4,116(16.47) |  |
| Never | 27,297(6.31) | 2,635(10.54) |  |
| **Sleep duration (hours), mean ± SD** | 7.17±1.08 | 7.17±1.23 | 0.608 |
| **Time spent watching TV (hours/day), mean ± SD** | 2.76±1.64 | 3.2±1.75 | <0.001 |
| **IPAQ** |  |  | <0.001 |
| Low | 63,471(18.14) | 4,512(23.56) |  |
| Moderate | 142,840(40.82) | 7,971(41.62) |  |
| High | 143,627(41.04) | 6,671(34.83) |  |
| **Family history of diabetes** |  |  | <0.001 |
| None | 363,679(86.43) | 20,560(85.05) |  |
| Father/Mather | 54,408(12.93) | 3,450(14.27) |  |
| Both | 2,686(0.64) | 165(0.68) |  |
| **DBP (mmHg)** | 82.17±10.68 | 82.64±10.55 | <0.001 |
| **SBP (mmHg)** | 139.76±19.7 | 140.54±19.48 | <0.001 |
| **TC (mmol/L), mean ± SD** | 5.74±1.13 | 5.72±1.15 | 0.009 |
| **LDL (mmol/L), mean ± SD** | 3.59±0.86 | 3.6±0.88 | 0.008 |
| **Triglycerides (mmol/L), mean ± SD** | 1.73±1.01 | 1.91±1.02 | <0.001 |

BMI, Body mass index; DBP, Diastolic blood pressure, automated reading; GSD, gallstone disease; IQR, interquartile range; IPAQ, International Physical Activity Questionnaire; LDL, low density lipoprotein; SD, standard deviation; SBP, Systolic blood pressure, automated reading; T2DM, type 2 diabetes mellitus; TC, Cholesterol; TV, television.

## Table S5. Genome-wide genetic correlation between type2 diabetes and gallstone disease.

| **Trait1** | **Trait2** | ***r_g_*** | ***SE*** | ***P*-value** |
| --- | --- | --- | --- | --- |
| T2DM | GSD | 0.35 | 0.0357 | 7.71×10^-23^ |
| T2DM_adj_BMI | GSD | 0.22 | 0.0354 | 4.48×10^-10^ |

GSD, gallstone disease; 𝑟_𝑔_, genetic correlation; SE, standard error; T2DM, type 2 diabetes mellitus; T2DM_adj_BMI, removing the effect of body mass index on type 2 diabetes mellitus.

## Table S6. Effect estimates of multivariable Mendelian randomization analyses for one of covariates.

| **Exposures** | **Covariates** | **No. of SNP** | **Exposures** | |  | **Covariates** | |
| --- | --- | --- | --- | --- | --- | --- | --- |
|  |  |  | **OR (95%CI)** | ***P*_value** |  | **OR (95%CI)** | ***P*_value** |
| T2DM | BMI | 982 | 1.027(1.011-1.043) | 7.95E-04 |  | 1.752(1.652-1.859) | 1.95E-77 |
| T2DM | Waist-to-hip | 644 | 1.048(1.020-1.076) | 6.18E-04 |  | 1.568(1.431-1.717) | 3.97E-22 |
| T2DM | Waist-to-hip adjusted for BMI | 681 | 1.069(1.043-1.096) | 1.27E-07 |  | 1.153(1.068-1.244) | 2.41E-04 |
| T2DM | Fasting insulin | 274 | 1.087(1.052-1.123) | 6.11E-07 |  | 1.711(1.359-2.154) | 5.00E-06 |
| T2DM | Fasting insulin adjusted for BMI | 366 | 1.071(1.042-1.101) | 1.10E-06 |  | 1.567(1.278-1.922) | 1.62E-05 |
| T2DM | Smoking | 679 | 1.088(1.061-1.115) | 1.93E-11 |  | 1.262(1.143-1.393) | 3.86E-06 |
| T2DM | Alcohol intake | 421 | 1.085(1.051-1.119) | 3.06E-07 |  | 1.137(0.914-1.414) | 2.49E-01 |
| T2DM | High density lipoprotein | 649 | 1.080(1.047-1.115) | 1.42E-06 |  | 0.932(0.871-0.997) | 3.92E-02 |
| T2DM | Low density lipoprotein | 667 | 1.063(1.022-1.107) | 2.62E-03 |  | 0.824(0.768-0.885) | 1.06E-07 |
| T2DM | Total cholesterol | 703 | 1.064(1.023-1.106) | 1.74E-03 |  | 0.829(0.770-0.892) | 5.91E-07 |
| T2DM | Triglycerides | 682 | 1.089(1.056-1.122) | 4.25E-08 |  | 0.999(0.934-1.068) | 9.70E-01 |
| T2DM | Lipoprotein A | 508 | 1.091(1.063-1.121) | 9.71E-11 |  | 0.982(0.962-1.002) | 8.40E-02 |
| T2DM | Apolipoprotein A | 752 | 1.103(1.073-1.134) | 3.60E-12 |  | 1.001(0.959-1.045) | 9.51E-01 |
| T2DM | Apolipoprotein B | 902 | 1.083(1.048-1.118) | 1.28E-06 |  | 0.890(0.866-0.914) | 4.62E-18 |

BMI, body mass index, CI, confidence interval; OR, odds ratio; T2DM, type 2 diabetes mellitus.

## Table S7. Effect estimates of multivariable Mendelian randomization analyses.

| **Factors** | **No. of SNP** | **OR (95%CI)** | ***P*_value** |
| --- | --- | --- | --- |
| **Full model** | | | |
| T2DM | 3,712 | 1.015(0.991-1.039) | 2.21E-01 |
| BMI | 3,712 | 1.759(1.655-1.869) | 2.40E-74 |
| Waist-to-hip adjusted for BMI | 3,712 | 1.295(1.210-1.387) | 1.32E-13 |
| Fasting insulin adjusted for BMI | 3,712 | 1.582(1.387-1.804) | 7.94E-12 |
| Smoking | 3,712 | 0.983(0.929-1.041) | 5.59E-01 |
| Alcohol intake | 3,712 | 1.119(0.988-1.266) | 7.60E-02 |
| High density lipoprotein | 3,712 | 1.089(0.910-1.305) | 3.52E-01 |
| Low density lipoprotein | 3,712 | 1.244(0.867-1.783) | 2.36E-01 |
| Total cholesterol | 3,712 | 0.695(0.461-1.049) | 8.30E-02 |
| Triglycerides | 3,712 | 1.171(0.997-1.377) | 5.50E-02 |
| Lipoprotein A | 3,712 | 1.002(0.984-1.020) | 8.65E-01 |
| Apolipoprotein A | 3,712 | 1.029(0.951-1.114) | 4.73E-01 |
| Apolipoprotein B | 3,712 | 0.976(0.926-1.029) | 3.72E-01 |
| **Final model** | | | |
| T2DM | 1,373 | 1.017(1.003-1.032) | 0.02 |
| BMI | 1,373 | 1.792(1.698-1.892) | 9.53E-99 |
| Waist-to-hip adjusted for BMI | 1,373 | 1.206(1.128-1.290) | 4.30E-08 |
| Fasting insulin adjusted for BMI | 1,373 | 1.646(1.430-1.895) | 4.00E-12 |

BMI, body mass index, CI, confidence interval; OR, odds ratio; T2DM, type 2 diabetes mellitus.

## Table S8. Pleiotropic SNPs identified by cross-trait meta-analysis between type 2 diabetes mellitus and gallstone disease‡.

| **SNPs** | **A1** | **A2** | **BETA** | |  | ***P*_value** | | | **Genomic coordinates** | **Genes within clumping area** | **Mapped genes** |
| --- | --- | --- | --- | --- | --- | --- | --- | --- | --- | --- | --- |
|  |  |  | **T2DM** | **GSD** |  | **T2DM** | **GSD** | **CPASSOC** |  |  |  |
| rs1260326 | C | T | 0.079 | -0.067 |  | 1.22E-23 | 1.30E-24 | 1.00E-46 | chr2:27548038-28113911 | *ZNF513, ZNF512, SUPT7L, SNX17, SLC4A1AP, RBKS, PPM1G, NRBP1, MRPL33, KRTCAP3, IFT172, GTF3C2, GTF3C2-AS1, GPN1, GCKR, FTH1P3, FNDC4, EIF2B4, CCDC121, C2orf16, BRE, BRE-AS1* | *GCKR* |
| rs13029250 | T | G | 0.073 | -0.032 |  | 9.00E-22 | 4.50E-07 | 2.22E-22 | chr2:43501802-43808065 | *THADA* | *THADA, RNU6-958P* |
| rs72870502 | T | C | 0.066 | -0.044 |  | 1.40E-10 | 2.50E-07 | 1.35E-14 | chr2:43920357-43933042 | *PLEKHH2* | *PLEKHH2* |
| rs362307 | T | C | 0.065 | 0.074 |  | 6.17E-06 | 1.10E-09 | 3.47E-14 | chr4:2935618-3437308 | *RGS12, NOP14-AS1, NOP14, MSANTD1, MFSD10, HTT, HTT-AS, GRK4* | *HTT, MSANTD1* |
| rs2523504 | C | T | 0.041 | -0.054 |  | 6.09E-06 | 6.10E-14 | 9.66E-19 | chr6:31150435-31555130 | *TNF, SNORD84, SNORD117, NFKBIL1, MIR6891, MICB, MICA, MCCD1, LST1, LTB, LTA, HLA-C, HLA-B, HCP5, HCG26, HCG27, DDX39B, ATP6V1G2-DDX39B, ATP6V1G2* | *ATP6V1G2, NFKBIL1, DDX39B, ATP6V1G2-DDX39B, DDX39B-AS1, SNORD84, SNORD83, DASS-161H22.6* |
| rs2857609 | G | A | 0.055 | -0.073 |  | 9.15E-07 | 9.30E-17 | 1.36E-22 | chr6:31114449-32071893 | *ZBTB12, VWA7, VARS, TNXB, TNXA, TNF, TCF19, STK19, SNORD84, SNORD52, SNORD48, SNORD117, SNORA38, SLC44A4, SKIV2L, SAPCD1, PSORS1C3, PRRC2A, POU5F1, NFKBIL1, NEU1, NELFE, NCR3, MSH5-SAPCD1, MSH5, MIR6891, MIR6832, MIR4646, MIR1236, MICB, MICA, MCCD1, LY6G6C, LY6G6E, LY6G6F, LY6G6D, LY6G5B, LY6G5C, LST1, LTB, LTA, LSM2, LOC102060414, HSPA1B, HSPA1L, HSPA1A, HLA-C, HLA-B, HCP5, HCG26, HCG27, GPANK1, EHMT2, DXO, DDX39B, DDAH2, CYP21A1P, CYP21A2, CSNK2B, CLIC1, CFB, CCHCR1, C6orf48, C6orf25, C6orf47, C4B_2, C4A, C4B, C2, BAG6, ATP6V1G2-DDX39B, ATP6V1G2, APOM, AIF1, ABHD16A* | *UQCRHP1, BX511262.2* |
| rs3130279 | G | A | 0.058 | -0.071 |  | 4.81E-07 | 1.90E-15 | 2.84E-21 | chr6:32029226-32602482 | *TNXB, RNF5, RNF5P1, PRRT1, PPT2, PPT2-EGFL8, PBX2, NOTCH4, MIR6833, MIR6721, LOC100507547, HLA-DRA, HLA-DRB5, HLA-DRB6, HLA-DRB1, HCG23, GPSM3, FKBPL, EGFL8, C6orf10, BTNL2, ATF6B, AGER, AGPAT1* | *PRRT1* |
| rs879882 | C | T | 0.037 | -0.048 |  | 4.14E-06 | 4.00E-13 | 3.88E-18 | chr6:31005726-31319157 | *TCF19, PSORS1C1, PSORS1C3, PSORS1C2, POU5F1, HLA-C, HCG27, HCG22, CDSN, CCHCR1, C6orf15* | *POU5F1, PSORS1C3, TCF19, CR847794.1, CR759815.2* |
| rs7461273 | G | C | -0.038 | -0.035 |  | 6.92E-07 | 9.60E-08 | 1.20E-12 | chr8:11517977-11894039 | *NEIL2, GATA4, FDFT1, DEFB135, DEFB136, DEFB134, CTSB, C8orf49* | *OR7E158P* |
| rs11244061 | T | C | 0.062 | 0.057 |  | 1.60E-07 | 1.90E-08 | 5.27E-14 | chr9:136153981-136339755 | *SURF4, SURF6, SURF1, SURF2, SNORD36B, SNORD36C, SNORD36A, SNORD24, SLC2A6, RPL7A, REXO4, MED22, CACFD1, C9orf96, ADAMTS13* | *ABO* |
| rs10882889 | G | A | -0.042 | 0.030 |  | 5.92E-08 | 4.30E-06 | 1.36E-11 | chr10:98950477-99290349 | *ZDHHC16, UBTD1, RRP12, PGAM1, MMS19, FRAT1, FRAT2, EXOSC1, ARHGAP19, ARHGAP19-SLIT1* | *ARHGAP19-SLIT1, ARHGAP19* |
| rs519790 | G | C | 0.038 | -0.037 |  | 4.05E-06 | 3.80E-08 | 1.31E-12 | chr11:72411664-72894273 | *STARD10, MIR4692, FCHSD2, ATG16L2, ARAP1* | *STARD10, ARAP1* |
| rs1169288 | C | A | -0.081 | -0.049 |  | 5.84E-23 | 7.30E-13 | 5.75E-30 | chr12:121189116-121485336 | *SPPL3, OASL, HNF1A-AS1, HNF1A, C12orf43* | *HNF1A, HNF1A-AS1* |
| rs1169307 | C | T | -0.057 | -0.041 |  | 1.63E-13 | 4.50E-10 | 3.19E-20 | chr12:121352974-121483489 | *OASL, HNF1A-AS1, HNF1A, C12orf43* | *HNF1A, C12orf43, RP11-216P16.2* |
| rs28929474 | T | C | 0.326 | -0.110 |  | 1.11E-39 | 5.90E-06 | 1.32E-41 | chr14:94672731-94877868 | *SERPINA1, SERPINA6, SERPINA2, SERPINA10, PPP4R4* | *SERPINA1* |
| rs35134156 | G | A | 0.035 | -0.041 |  | 4.05E-06 | 4.10E-10 | 5.53E-15 | chr15:76953256-77324880 | *SCAPER, RCN2, PSTPIP1* | *PSTPIP1* |
| rs11075985 | A | C | 0.035 | 0.120 |  | 3.92E-06 | 1.00E-74 | 3.35E-83 | chr16:53797565-53848561 | *FTO* | *FTO* |
| rs62052815 | T | C | -0.042 | -0.040 |  | 5.63E-08 | 6.50E-10 | 3.51E-16 | chr16:69545116-69976089 | *WWP2, NQO1, NOB1, NFAT5, MIR1538, MIR140* | *━* |
| rs429358 | C | T | -0.056 | 0.080 |  | 1.02E-07 | 1.80E-18 | 3.02E-25 | chr19:45387459-45428234 | *TOMM40, PVRL2, APOE, APOC1* | *APOE, TOMM40* |
| rs58304657 | C | G | -0.072 | 0.069 |  | 7.92E-09 | 1.40E-11 | 3.20E-19 | chr19:46148237-46376217 | *SYMPK, SNRPD2, SIX5, RSPH6A, QPCTL, MIR642A, MIR642B, LOC388553, GIPR, FOXA3, FBXO46, EML2, DMWD, DMPK* | *GIPR, MIR642A* |
| rs1800961 | T | C | 0.289 | 0.160 |  | 4.48E-50 | 3.20E-20 | 5.69E-58 | chr20:42958768-43042364 | *R3HDML, MIR3646, HNF4A, HNF4A-AS1* | *HNF4A* |
| rs738408 | T | C | -0.048 | 0.049 |  | 1.75E-07 | 1.80E-10 | 1.25E-16 | chr22:44324727-44395451 | *SAMM50, PNPLA3, PARVB* | *PNPLA3* |
| rs76747430 | G | A | -0.061 | 0.040 |  | 3.40E-09 | 2.30E-06 | 1.19E-12 | chr22:40530052-41262852 | *XPNPEP3, TNRC6B, ST13, SLC25A17, SGSM3, MKL1, MIR4766, MCHR1, LOC101927257, DNAJB7, ADSL* | *MKL1, RP5-1042K10.12* |

CPASSOC, Cross-Phenotype Association; GSD, gallstone disease; SNPs, single nucleotide Polymorphisms; T2DM, type 2 diabetes mellitus; ‡, P_CPASSOC_ < 5×10-8, single trait P-value < 1×10-5, clumping r2=0.2.

## Table S9. Pleiotropic SNPs identified by cross-trait meta-analysis between type 2 diabetes mellitus (adjusted for BMI) and gallstone disease‡.

| **SNPs** | **A1** | **A2** | **BETA** | |  | ***P*_value** | | | **Genomic coordinates** | **Genes within clumping area** | **Mapped genes** |
| --- | --- | --- | --- | --- | --- | --- | --- | --- | --- | --- | --- |
|  |  |  | **T2DM** | **GSD** |  | **T2DM** | **GSD** | **CPASSOC** |  |  |  |
| rs1260326 | C | T | 0.079 | -0.067 |  | 1.22E-23 | 7.40E-19 | 6.81E-40 | chr2:27548038-28113911 | *ZNF513, ZNF512, SUPT7L, SNX17, SLC4A1AP, RBKS, PPM1G, NRBP1, MRPL33, KRTCAP3, IFT172, GTF3C2, GTF3C2-AS1, GPN1, GCKR, FTH1P3, FNDC4, EIF2B4, CCDC121, C2orf16, BRE, BRE-AS1* | *GCKR* |
| rs149797* | T | C | -0.043 | 0.036 |  | 2.20E-07 | 5.70E-06 | 3.52E-11 | chr5:72071072-72406659 | *TNPO1, MIR4804, FCHO2* | *━* |
| rs2239525 | A | G | 0.041 | 0.059 |  | 4.81E-06 | 3.90E-12 | 7.54E-16 | chr6:31437872-31555130 | *TNF, SNORD84, SNORD117, NFKBIL1, MICB, MCCD1, LST1, LTB, LTA, HCG26, DDX39B, ATP6V1G2-DDX39B, ATP6V1G2* | *ATP6V1G2, DDX39B, ATP6V1G2-DDX39B, DDX39B-AS1, SNORD84, SNORD83, DASS-161H22.6* |
| rs2857609 | G | A | 0.055 | -0.070 |  | 9.15E-07 | 1.10E-11 | 5.78E-17 | chr6:31114449-32071893 | *ZBTB12, VWA7, VARS, TNXB, TNXA, TNF, TCF19, STK19, SNORD84, SNORD52, SNORD48, SNORD117, SNORA38, SLC44A4, SKIV2L, SAPCD1, PSORS1C3, PRRC2A, POU5F1, NFKBIL1, NEU1, NELFE, NCR3, MSH5-SAPCD1, MSH5, MIR6891, MIR6832, MIR4646, MIR1236, MICB, MICA, MCCD1, LY6G6C, LY6G6E, LY6G6F, LY6G6D, LY6G5B, LY6G5C, LST1, LTB, LTA, LSM2, LOC102060414, HSPA1B, HSPA1L, HSPA1A, HLA-C, HLA-B, HCP5, HCG26, HCG27, GPANK1, EHMT2, DXO, DDX39B, DDAH2, CYP21A1P, CYP21A2, CSNK2B, CLIC1, CFB, CCHCR1, C6orf48, C6orf25, C6orf47, C4B_2, C4A, C4B, C2, BAG6, ATP6V1G2-DDX39B, ATP6V1G2, APOM, AIF1, ABHD16A* | *UQCRHP1, BX511262.2* |
| rs3130279 | G | A | 0.058 | -0.072 |  | 4.81E-07 | 1.30E-11 | 6.04E-16 | chr6:32080146-32602482 | *RNF5, RNF5P1, PRRT1, PPT2, PPT2-EGFL8, PBX2, NOTCH4, MIR6833, MIR6721, LOC100507547, HLA-DRA, HLA-DRB5, HLA-DRB6, HLA-DRB1, HCG23, GPSM3, FKBPL, EGFL8, C6orf10, BTNL2, ATF6B, AGER, AGPAT1* | *PRRT1* |
| rs879882 | C | T | 0.037 | -0.053 |  | 4.14E-06 | 1.50E-11 | 1.22E-15 | chr6:31005726-31319157 | *TCF19, PSORS1C1, PSORS1C3, PSORS1C2, POU5F1, HLA-C, HCG27, HCG22, CDSN, CCHCR1, C6orf15* | *POU5F1, PSORS1C3, TCF19, CR847794.1, CR759815.2* |
| rs519790 | G | C | 0.038 | -0.042 |  | 4.05E-06 | 1.30E-07 | 6.60E-12 | chr11:72411664-72894273 | *STARD10, MIR4692, FCHSD2, ATG16L2, ARAP1* | *STARD10, ARAP1* |
| rs1169288 | C | A | -0.081 | -0.055 |  | 5.84E-23 | 6.80E-12 | 5.32E-31 | chr12:121353088-121485336 | *OASL, HNF1A-AS1, HNF1A, C12orf43* | *HNF1A, HNF1A-AS1* |
| rs1169307 | C | T | -0.057 | -0.042 |  | 1.63E-13 | 3.90E-08 | 1.61E-18 | chr12:121197124-121489657 | *SPPL3, OASL, HNF1A-AS1, HNF1A, C12orf43* | *HNF1A, C12orf43, RP11-216P16.2* |
| rs244418 | A | G | -0.038 | -0.040 |  | 1.04E-06 | 1.60E-07 | 2.94E-12 | chr16:69545116-69968892 | *WWP2, NQO1, NOB1, NFAT5, MIR1538, MIR140* | *NFAT5* |
| rs56094641 | G | A | 0.035 | -0.054 |  | 4.73E-06 | 1.30E-12 | 1.88E-16 | chr16:53797908-53845487 | *FTO* | *FTO* |
| rs58304657 | C | G | -0.072 | 0.110 |  | 7.92E-09 | 7.50E-19 | 2.50E-26 | chr19:46148237-46376217 | *SYMPK, SNRPD2, SIX5, RSPH6A, QPCTL, MIR642A, MIR642B, LOC388553, GIPR, FOXA3, FBXO46, EML2, DMWD, DMPK* | *GIPR, MIR642A* |
| rs1800961 | T | C | 0.289 | 0.180 |  | 4.48E-50 | 9.20E-18 | 1.44E-59 | chr20:42958768-43042364 | *R3HDML, MIR3646, HNF4A, HNF4A-AS1* | *HNF4A* |
| rs736820 | A | G | -0.043 | -0.035 |  | 4.41E-08 | 9.70E-06 | 1.25E-11 | chr20:43034016-43036649 | *HNF4A* | *HNF4A, MIR3646* |
| rs738408 | T | C | -0.048 | 0.062 |  | 1.75E-07 | 3.00E-12 | 1.00E-17 | chr22:44324727-44395451 | *SAMM50, PNPLA3, PARVB* | *PNPLA3* |

CPASSOC, Cross-Phenotype Association; GSD, gallstone disease; SNPs, single nucleotide Polymorphisms; T2DM, type 2 diabetes mellitus; *, a novel SNP; ‡, P_CPASSOC_ < 5×10-8, single trait P-value < 1×10-5, clumping r2=0.2.

## Table S10. Detailed annotation of each CPASSOC-identified SNPs for type 2 diabetes mellitus and gallstone disease.

| **SNPs** | **Location** | **Allele** | **Consequence** | **IMPACT** | **SYMBOL** | **Feature_type** | **BIOTYPE** |
| --- | --- | --- | --- | --- | --- | --- | --- |
| rs10882889 | 10:99045890-99045890 | C | intron_variant | MODIFIER | *ARHGAP19-SLIT1* | Transcript | protein_coding |
| rs10882889 | 10:99045890-99045890 | G | intron_variant | MODIFIER | *ARHGAP19-SLIT1* | Transcript | protein_coding |
| rs10882889 | 10:99045890-99045890 | T | intron_variant | MODIFIER | *ARHGAP19-SLIT1* | Transcript | protein_coding |
| rs10882889 | 10:99045890-99045890 | C | intron_variant | MODIFIER | *ARHGAP19-SLIT1* | Transcript | protein_coding |
| rs10882889 | 10:99045890-99045890 | G | intron_variant | MODIFIER | *ARHGAP19-SLIT1* | Transcript | protein_coding |
| rs10882889 | 10:99045890-99045890 | T | intron_variant | MODIFIER | *ARHGAP19-SLIT1* | Transcript | protein_coding |
| rs10882889 | 10:99045890-99045890 | C | intron_variant | MODIFIER | *ARHGAP19* | Transcript | protein_coding |
| rs10882889 | 10:99045890-99045890 | G | intron_variant | MODIFIER | *ARHGAP19* | Transcript | protein_coding |
| rs10882889 | 10:99045890-99045890 | T | intron_variant | MODIFIER | *ARHGAP19* | Transcript | protein_coding |
| rs10882889 | 10:99045890-99045890 | C | intron_variant | MODIFIER | *ARHGAP19-SLIT1* | Transcript | protein_coding |
| rs10882889 | 10:99045890-99045890 | G | intron_variant | MODIFIER | *ARHGAP19-SLIT1* | Transcript | protein_coding |
| rs10882889 | 10:99045890-99045890 | T | intron_variant | MODIFIER | *ARHGAP19-SLIT1* | Transcript | protein_coding |
| rs10882889 | 10:99045890-99045890 | C | intron_variant | MODIFIER | *ARHGAP19* | Transcript | protein_coding |
| rs10882889 | 10:99045890-99045890 | G | intron_variant | MODIFIER | *ARHGAP19* | Transcript | protein_coding |
| rs10882889 | 10:99045890-99045890 | T | intron_variant | MODIFIER | *ARHGAP19* | Transcript | protein_coding |
| rs10882889 | 10:99045890-99045890 | C | intron_variant,NMD_transcript_variant | MODIFIER | *ARHGAP19-SLIT1* | Transcript | nonsense_mediated_decay |
| rs10882889 | 10:99045890-99045890 | G | intron_variant,NMD_transcript_variant | MODIFIER | *ARHGAP19-SLIT1* | Transcript | nonsense_mediated_decay |
| rs10882889 | 10:99045890-99045890 | T | intron_variant,NMD_transcript_variant | MODIFIER | *ARHGAP19-SLIT1* | Transcript | nonsense_mediated_decay |
| rs10882889 | 10:99045890-99045890 | C | intron_variant,NMD_transcript_variant | MODIFIER | *ARHGAP19* | Transcript | nonsense_mediated_decay |
| rs10882889 | 10:99045890-99045890 | G | intron_variant,NMD_transcript_variant | MODIFIER | *ARHGAP19* | Transcript | nonsense_mediated_decay |
| rs10882889 | 10:99045890-99045890 | T | intron_variant,NMD_transcript_variant | MODIFIER | *ARHGAP19* | Transcript | nonsense_mediated_decay |
| rs10882889 | 10:99045890-99045890 | C | intron_variant,non_coding_transcript_variant | MODIFIER | *ARHGAP19* | Transcript | retained_intron |
| rs10882889 | 10:99045890-99045890 | G | intron_variant,non_coding_transcript_variant | MODIFIER | *ARHGAP19* | Transcript | retained_intron |
| rs10882889 | 10:99045890-99045890 | T | intron_variant,non_coding_transcript_variant | MODIFIER | *ARHGAP19* | Transcript | retained_intron |
| rs11075985 | 16:53805207-53805207 | A | intron_variant | MODIFIER | *FTO* | Transcript | protein_coding |
| rs11075985 | 16:53805207-53805207 | A | intron_variant,NMD_transcript_variant | MODIFIER | *FTO* | Transcript | nonsense_mediated_decay |
| rs11075985 | 16:53805207-53805207 | A | intron_variant | MODIFIER | *FTO* | Transcript | protein_coding |
| rs11075985 | 16:53805207-53805207 | A | intron_variant,non_coding_transcript_variant | MODIFIER | *FTO* | Transcript | processed_transcript |
| rs11075985 | 16:53805207-53805207 | A | regulatory_region_variant | MODIFIER | *-* | RegulatoryFeature | promoter_flanking_region |
| rs11244061 | 9:136153981-136153981 | G | upstream_gene_variant | MODIFIER | *ABO* | Transcript | processed_transcript |
| rs11244061 | 9:136153981-136153981 | T | upstream_gene_variant | MODIFIER | *ABO* | Transcript | processed_transcript |
| rs11244061 | 9:136153981-136153981 | G | upstream_gene_variant | MODIFIER | *ABO* | Transcript | processed_transcript |
| rs11244061 | 9:136153981-136153981 | T | upstream_gene_variant | MODIFIER | *ABO* | Transcript | processed_transcript |
| rs11244061 | HG79_PATCH:136154072-136154072 | G | upstream_gene_variant | MODIFIER | *ABO* | Transcript | protein_coding |
| rs11244061 | HG79_PATCH:136154072-136154072 | T | upstream_gene_variant | MODIFIER | *ABO* | Transcript | protein_coding |
| rs1169288 | 12:121416650-121416650 | C | missense_variant | MODERATE | *HNF1A* | Transcript | protein_coding |
| rs1169288 | 12:121416650-121416650 | T | missense_variant | MODERATE | *HNF1A* | Transcript | protein_coding |
| rs1169288 | 12:121416650-121416650 | C | missense_variant | MODERATE | *HNF1A* | Transcript | protein_coding |
| rs1169288 | 12:121416650-121416650 | T | missense_variant | MODERATE | *HNF1A* | Transcript | protein_coding |
| rs1169288 | 12:121416650-121416650 | C | missense_variant | MODERATE | *HNF1A* | Transcript | protein_coding |
| rs1169288 | 12:121416650-121416650 | T | missense_variant | MODERATE | *HNF1A* | Transcript | protein_coding |
| rs1169288 | 12:121416650-121416650 | C | intron_variant,non_coding_transcript_variant | MODIFIER | *HNF1A-AS1* | Transcript | antisense |
| rs1169288 | 12:121416650-121416650 | T | intron_variant,non_coding_transcript_variant | MODIFIER | *HNF1A-AS1* | Transcript | antisense |
| rs1169288 | 12:121416650-121416650 | C | intron_variant,non_coding_transcript_variant | MODIFIER | *HNF1A-AS1* | Transcript | antisense |
| rs1169288 | 12:121416650-121416650 | T | intron_variant,non_coding_transcript_variant | MODIFIER | *HNF1A-AS1* | Transcript | antisense |
| rs1169288 | 12:121416650-121416650 | C | intron_variant | MODIFIER | *HNF1A* | Transcript | protein_coding |
| rs1169288 | 12:121416650-121416650 | T | intron_variant | MODIFIER | *HNF1A* | Transcript | protein_coding |
| rs1169288 | 12:121416650-121416650 | C | intron_variant,non_coding_transcript_variant | MODIFIER | *HNF1A-AS1* | Transcript | antisense |
| rs1169288 | 12:121416650-121416650 | T | intron_variant,non_coding_transcript_variant | MODIFIER | *HNF1A-AS1* | Transcript | antisense |
| rs1169288 | 12:121416650-121416650 | C | splice_region_variant,intron_variant | LOW | *HNF1A* | Transcript | protein_coding |
| rs1169288 | 12:121416650-121416650 | T | splice_region_variant,intron_variant | LOW | *HNF1A* | Transcript | protein_coding |
| rs1169288 | 12:121416650-121416650 | C | missense_variant,NMD_transcript_variant | MODERATE | *HNF1A* | Transcript | nonsense_mediated_decay |
| rs1169288 | 12:121416650-121416650 | T | missense_variant,NMD_transcript_variant | MODERATE | *HNF1A* | Transcript | nonsense_mediated_decay |
| rs1169288 | 12:121416650-121416650 | C | missense_variant,NMD_transcript_variant | MODERATE | *HNF1A* | Transcript | nonsense_mediated_decay |
| rs1169288 | 12:121416650-121416650 | T | missense_variant,NMD_transcript_variant | MODERATE | *HNF1A* | Transcript | nonsense_mediated_decay |
| rs1169288 | 12:121416650-121416650 | C | missense_variant | MODERATE | *HNF1A* | Transcript | protein_coding |
| rs1169288 | 12:121416650-121416650 | T | missense_variant | MODERATE | *HNF1A* | Transcript | protein_coding |
| rs1169288 | 12:121416650-121416650 | C | missense_variant,NMD_transcript_variant | MODERATE | *HNF1A* | Transcript | nonsense_mediated_decay |
| rs1169288 | 12:121416650-121416650 | T | missense_variant,NMD_transcript_variant | MODERATE | *HNF1A* | Transcript | nonsense_mediated_decay |
| rs1169288 | 12:121416650-121416650 | C | intron_variant | MODIFIER | *HNF1A* | Transcript | protein_coding |
| rs1169288 | 12:121416650-121416650 | T | intron_variant | MODIFIER | *HNF1A* | Transcript | protein_coding |
| rs1169288 | 12:121416650-121416650 | C | missense_variant | MODERATE | *HNF1A* | Transcript | protein_coding |
| rs1169288 | 12:121416650-121416650 | T | missense_variant | MODERATE | *HNF1A* | Transcript | protein_coding |
| rs1169288 | 12:121416650-121416650 | C | splice_region_variant,intron_variant,NMD_transcript_variant | LOW | *HNF1A* | Transcript | nonsense_mediated_decay |
| rs1169288 | 12:121416650-121416650 | T | splice_region_variant,intron_variant,NMD_transcript_variant | LOW | *HNF1A* | Transcript | nonsense_mediated_decay |
| rs1169288 | 12:121416650-121416650 | C | missense_variant,NMD_transcript_variant | MODERATE | *HNF1A* | Transcript | nonsense_mediated_decay |
| rs1169288 | 12:121416650-121416650 | T | missense_variant,NMD_transcript_variant | MODERATE | *HNF1A* | Transcript | nonsense_mediated_decay |
| rs1169288 | 12:121416650-121416650 | C | regulatory_region_variant | MODIFIER | *-* | RegulatoryFeature | promoter |
| rs1169288 | 12:121416650-121416650 | T | regulatory_region_variant | MODIFIER | *-* | RegulatoryFeature | promoter |
| rs1169307 | 12:121438382-121438382 | A | intron_variant | MODIFIER | *HNF1A* | Transcript | protein_coding |
| rs1169307 | 12:121438382-121438382 | C | intron_variant | MODIFIER | *HNF1A* | Transcript | protein_coding |
| rs1169307 | 12:121438382-121438382 | G | intron_variant | MODIFIER | *HNF1A* | Transcript | protein_coding |
| rs1169307 | 12:121438382-121438382 | A | downstream_gene_variant | MODIFIER | *C12orf43* | Transcript | protein_coding |
| rs1169307 | 12:121438382-121438382 | C | downstream_gene_variant | MODIFIER | *C12orf43* | Transcript | protein_coding |
| rs1169307 | 12:121438382-121438382 | G | downstream_gene_variant | MODIFIER | *C12orf43* | Transcript | protein_coding |
| rs1169307 | 12:121438382-121438382 | A | downstream_gene_variant | MODIFIER | *C12orf43* | Transcript | protein_coding |
| rs1169307 | 12:121438382-121438382 | C | downstream_gene_variant | MODIFIER | *C12orf43* | Transcript | protein_coding |
| rs1169307 | 12:121438382-121438382 | G | downstream_gene_variant | MODIFIER | *C12orf43* | Transcript | protein_coding |
| rs1169307 | 12:121438382-121438382 | A | downstream_gene_variant | MODIFIER | *HNF1A* | Transcript | protein_coding |
| rs1169307 | 12:121438382-121438382 | C | downstream_gene_variant | MODIFIER | *HNF1A* | Transcript | protein_coding |
| rs1169307 | 12:121438382-121438382 | G | downstream_gene_variant | MODIFIER | *HNF1A* | Transcript | protein_coding |
| rs1169307 | 12:121438382-121438382 | A | downstream_gene_variant | MODIFIER | *HNF1A* | Transcript | protein_coding |
| rs1169307 | 12:121438382-121438382 | C | downstream_gene_variant | MODIFIER | *HNF1A* | Transcript | protein_coding |
| rs1169307 | 12:121438382-121438382 | G | downstream_gene_variant | MODIFIER | *HNF1A* | Transcript | protein_coding |
| rs1169307 | 12:121438382-121438382 | A | downstream_gene_variant | MODIFIER | *C12orf43* | Transcript | protein_coding |
| rs1169307 | 12:121438382-121438382 | C | downstream_gene_variant | MODIFIER | *C12orf43* | Transcript | protein_coding |
| rs1169307 | 12:121438382-121438382 | G | downstream_gene_variant | MODIFIER | *C12orf43* | Transcript | protein_coding |
| rs1169307 | 12:121438382-121438382 | A | downstream_gene_variant | MODIFIER | *C12orf43* | Transcript | retained_intron |
| rs1169307 | 12:121438382-121438382 | C | downstream_gene_variant | MODIFIER | *C12orf43* | Transcript | retained_intron |
| rs1169307 | 12:121438382-121438382 | G | downstream_gene_variant | MODIFIER | *C12orf43* | Transcript | retained_intron |
| rs1169307 | 12:121438382-121438382 | A | downstream_gene_variant | MODIFIER | *C12orf43* | Transcript | protein_coding |
| rs1169307 | 12:121438382-121438382 | C | downstream_gene_variant | MODIFIER | *C12orf43* | Transcript | protein_coding |
| rs1169307 | 12:121438382-121438382 | G | downstream_gene_variant | MODIFIER | *C12orf43* | Transcript | protein_coding |
| rs1169307 | 12:121438382-121438382 | A | downstream_gene_variant | MODIFIER | *HNF1A* | Transcript | protein_coding |
| rs1169307 | 12:121438382-121438382 | C | downstream_gene_variant | MODIFIER | *HNF1A* | Transcript | protein_coding |
| rs1169307 | 12:121438382-121438382 | G | downstream_gene_variant | MODIFIER | *HNF1A* | Transcript | protein_coding |
| rs1169307 | 12:121438382-121438382 | A | downstream_gene_variant | MODIFIER | *C12orf43* | Transcript | protein_coding |
| rs1169307 | 12:121438382-121438382 | C | downstream_gene_variant | MODIFIER | *C12orf43* | Transcript | protein_coding |
| rs1169307 | 12:121438382-121438382 | G | downstream_gene_variant | MODIFIER | *C12orf43* | Transcript | protein_coding |
| rs1169307 | 12:121438382-121438382 | A | downstream_gene_variant | MODIFIER | *C12orf43* | Transcript | protein_coding |
| rs1169307 | 12:121438382-121438382 | C | downstream_gene_variant | MODIFIER | *C12orf43* | Transcript | protein_coding |
| rs1169307 | 12:121438382-121438382 | G | downstream_gene_variant | MODIFIER | *C12orf43* | Transcript | protein_coding |
| rs1169307 | 12:121438382-121438382 | A | downstream_gene_variant | MODIFIER | *C12orf43* | Transcript | protein_coding |
| rs1169307 | 12:121438382-121438382 | C | downstream_gene_variant | MODIFIER | *C12orf43* | Transcript | protein_coding |
| rs1169307 | 12:121438382-121438382 | G | downstream_gene_variant | MODIFIER | *C12orf43* | Transcript | protein_coding |
| rs1169307 | 12:121438382-121438382 | A | downstream_gene_variant | MODIFIER | *HNF1A* | Transcript | protein_coding |
| rs1169307 | 12:121438382-121438382 | C | downstream_gene_variant | MODIFIER | *HNF1A* | Transcript | protein_coding |
| rs1169307 | 12:121438382-121438382 | G | downstream_gene_variant | MODIFIER | *HNF1A* | Transcript | protein_coding |
| rs1169307 | 12:121438382-121438382 | A | downstream_gene_variant | MODIFIER | *HNF1A* | Transcript | nonsense_mediated_decay |
| rs1169307 | 12:121438382-121438382 | C | downstream_gene_variant | MODIFIER | *HNF1A* | Transcript | nonsense_mediated_decay |
| rs1169307 | 12:121438382-121438382 | G | downstream_gene_variant | MODIFIER | *HNF1A* | Transcript | nonsense_mediated_decay |
| rs1169307 | 12:121438382-121438382 | A | downstream_gene_variant | MODIFIER | *C12orf43* | Transcript | nonsense_mediated_decay |
| rs1169307 | 12:121438382-121438382 | C | downstream_gene_variant | MODIFIER | *C12orf43* | Transcript | nonsense_mediated_decay |
| rs1169307 | 12:121438382-121438382 | G | downstream_gene_variant | MODIFIER | *C12orf43* | Transcript | nonsense_mediated_decay |
| rs1169307 | 12:121438382-121438382 | A | downstream_gene_variant | MODIFIER | *C12orf43* | Transcript | protein_coding |
| rs1169307 | 12:121438382-121438382 | C | downstream_gene_variant | MODIFIER | *C12orf43* | Transcript | protein_coding |
| rs1169307 | 12:121438382-121438382 | G | downstream_gene_variant | MODIFIER | *C12orf43* | Transcript | protein_coding |
| rs1169307 | 12:121438382-121438382 | A | intron_variant,NMD_transcript_variant | MODIFIER | *HNF1A* | Transcript | nonsense_mediated_decay |
| rs1169307 | 12:121438382-121438382 | C | intron_variant,NMD_transcript_variant | MODIFIER | *HNF1A* | Transcript | nonsense_mediated_decay |
| rs1169307 | 12:121438382-121438382 | G | intron_variant,NMD_transcript_variant | MODIFIER | *HNF1A* | Transcript | nonsense_mediated_decay |
| rs1169307 | 12:121438382-121438382 | A | intron_variant | MODIFIER | *HNF1A* | Transcript | protein_coding |
| rs1169307 | 12:121438382-121438382 | C | intron_variant | MODIFIER | *HNF1A* | Transcript | protein_coding |
| rs1169307 | 12:121438382-121438382 | G | intron_variant | MODIFIER | *HNF1A* | Transcript | protein_coding |
| rs1169307 | 12:121438382-121438382 | A | downstream_gene_variant | MODIFIER | *HNF1A* | Transcript | nonsense_mediated_decay |
| rs1169307 | 12:121438382-121438382 | C | downstream_gene_variant | MODIFIER | *HNF1A* | Transcript | nonsense_mediated_decay |
| rs1169307 | 12:121438382-121438382 | G | downstream_gene_variant | MODIFIER | *HNF1A* | Transcript | nonsense_mediated_decay |
| rs1169307 | 12:121438382-121438382 | A | downstream_gene_variant | MODIFIER | *HNF1A* | Transcript | retained_intron |
| rs1169307 | 12:121438382-121438382 | C | downstream_gene_variant | MODIFIER | *HNF1A* | Transcript | retained_intron |
| rs1169307 | 12:121438382-121438382 | G | downstream_gene_variant | MODIFIER | *HNF1A* | Transcript | retained_intron |
| rs1169307 | 12:121438382-121438382 | A | downstream_gene_variant | MODIFIER | *HNF1A* | Transcript | protein_coding |
| rs1169307 | 12:121438382-121438382 | C | downstream_gene_variant | MODIFIER | *HNF1A* | Transcript | protein_coding |
| rs1169307 | 12:121438382-121438382 | G | downstream_gene_variant | MODIFIER | *HNF1A* | Transcript | protein_coding |
| rs1169307 | 12:121438382-121438382 | A | intron_variant | MODIFIER | *HNF1A* | Transcript | protein_coding |
| rs1169307 | 12:121438382-121438382 | C | intron_variant | MODIFIER | *HNF1A* | Transcript | protein_coding |
| rs1169307 | 12:121438382-121438382 | G | intron_variant | MODIFIER | *HNF1A* | Transcript | protein_coding |
| rs1169307 | 12:121438382-121438382 | A | downstream_gene_variant | MODIFIER | *HNF1A* | Transcript | nonsense_mediated_decay |
| rs1169307 | 12:121438382-121438382 | C | downstream_gene_variant | MODIFIER | *HNF1A* | Transcript | nonsense_mediated_decay |
| rs1169307 | 12:121438382-121438382 | G | downstream_gene_variant | MODIFIER | *HNF1A* | Transcript | nonsense_mediated_decay |
| rs1169307 | 12:121438382-121438382 | A | downstream_gene_variant | MODIFIER | *C12orf43* | Transcript | protein_coding |
| rs1169307 | 12:121438382-121438382 | C | downstream_gene_variant | MODIFIER | *C12orf43* | Transcript | protein_coding |
| rs1169307 | 12:121438382-121438382 | G | downstream_gene_variant | MODIFIER | *C12orf43* | Transcript | protein_coding |
| rs1169307 | 12:121438382-121438382 | A | intron_variant,NMD_transcript_variant | MODIFIER | *HNF1A* | Transcript | nonsense_mediated_decay |
| rs1169307 | 12:121438382-121438382 | C | intron_variant,NMD_transcript_variant | MODIFIER | *HNF1A* | Transcript | nonsense_mediated_decay |
| rs1169307 | 12:121438382-121438382 | G | intron_variant,NMD_transcript_variant | MODIFIER | *HNF1A* | Transcript | nonsense_mediated_decay |
| rs1169307 | 12:121438382-121438382 | A | non_coding_transcript_exon_variant | MODIFIER | *RP11-216P16.2* | Transcript | antisense |
| rs1169307 | 12:121438382-121438382 | C | non_coding_transcript_exon_variant | MODIFIER | *RP11-216P16.2* | Transcript | antisense |
| rs1169307 | 12:121438382-121438382 | G | non_coding_transcript_exon_variant | MODIFIER | *RP11-216P16.2* | Transcript | antisense |
| rs1260326 | 2:27730940-27730940 | C | missense_variant,splice_region_variant | MODERATE | *GCKR* | Transcript | protein_coding |
| rs1260326 | 2:27730940-27730940 | G | missense_variant,splice_region_variant | MODERATE | *GCKR* | Transcript | protein_coding |
| rs1260326 | 2:27730940-27730940 | C | missense_variant,splice_region_variant | MODERATE | *GCKR* | Transcript | protein_coding |
| rs1260326 | 2:27730940-27730940 | G | missense_variant,splice_region_variant | MODERATE | *GCKR* | Transcript | protein_coding |
| rs1260326 | 2:27730940-27730940 | C | missense_variant,splice_region_variant | MODERATE | *GCKR* | Transcript | protein_coding |
| rs1260326 | 2:27730940-27730940 | G | missense_variant,splice_region_variant | MODERATE | *GCKR* | Transcript | protein_coding |
| rs1260326 | 2:27730940-27730940 | C | downstream_gene_variant | MODIFIER | *GCKR* | Transcript | retained_intron |
| rs1260326 | 2:27730940-27730940 | G | downstream_gene_variant | MODIFIER | *GCKR* | Transcript | retained_intron |
| rs1260326 | 2:27730940-27730940 | C | splice_region_variant,non_coding_transcript_exon_variant | LOW | *GCKR* | Transcript | retained_intron |
| rs1260326 | 2:27730940-27730940 | G | splice_region_variant,non_coding_transcript_exon_variant | LOW | *GCKR* | Transcript | retained_intron |
| rs13029250 | 2:43638712-43638712 | T | intron_variant | MODIFIER | *THADA* | Transcript | protein_coding |
| rs13029250 | 2:43638712-43638712 | T | intron_variant,NMD_transcript_variant | MODIFIER | *THADA* | Transcript | nonsense_mediated_decay |
| rs13029250 | 2:43638712-43638712 | T | intron_variant,NMD_transcript_variant | MODIFIER | *THADA* | Transcript | nonsense_mediated_decay |
| rs13029250 | 2:43638712-43638712 | T | intron_variant | MODIFIER | *THADA* | Transcript | protein_coding |
| rs13029250 | 2:43638712-43638712 | T | intron_variant | MODIFIER | *THADA* | Transcript | protein_coding |
| rs13029250 | 2:43638712-43638712 | T | intron_variant | MODIFIER | *THADA* | Transcript | protein_coding |
| rs13029250 | 2:43638712-43638712 | T | intron_variant,NMD_transcript_variant | MODIFIER | *THADA* | Transcript | nonsense_mediated_decay |
| rs13029250 | 2:43638712-43638712 | T | intron_variant | MODIFIER | *THADA* | Transcript | protein_coding |
| rs13029250 | 2:43638712-43638712 | T | intron_variant,non_coding_transcript_variant | MODIFIER | *THADA* | Transcript | processed_transcript |
| rs13029250 | 2:43638712-43638712 | T | downstream_gene_variant | MODIFIER | *RNU6-958P* | Transcript | snRNA |
| rs1800961 | 20:43042364-43042364 | T | missense_variant | MODERATE | *HNF4A* | Transcript | protein_coding |
| rs1800961 | 20:43042364-43042364 | T | missense_variant | MODERATE | *HNF4A* | Transcript | protein_coding |
| rs1800961 | 20:43042364-43042364 | T | 3_prime_UTR_variant,NMD_transcript_variant | MODIFIER | *HNF4A* | Transcript | nonsense_mediated_decay |
| rs1800961 | 20:43042364-43042364 | T | missense_variant | MODERATE | *HNF4A* | Transcript | protein_coding |
| rs1800961 | 20:43042364-43042364 | T | missense_variant | MODERATE | *HNF4A* | Transcript | protein_coding |
| rs1800961 | 20:43042364-43042364 | T | missense_variant | MODERATE | *HNF4A* | Transcript | protein_coding |
| rs1800961 | 20:43042364-43042364 | T | missense_variant | MODERATE | *HNF4A* | Transcript | protein_coding |
| rs2523504 | 6:31510858-31510858 | A | downstream_gene_variant | MODIFIER | *ATP6V1G2* | Transcript | protein_coding |
| rs2523504 | 6:31510858-31510858 | C | downstream_gene_variant | MODIFIER | *ATP6V1G2* | Transcript | protein_coding |
| rs2523504 | 6:31510858-31510858 | A | upstream_gene_variant | MODIFIER | *NFKBIL1* | Transcript | protein_coding |
| rs2523504 | 6:31510858-31510858 | C | upstream_gene_variant | MODIFIER | *NFKBIL1* | Transcript | protein_coding |
| rs2523504 | 6:31510858-31510858 | A | upstream_gene_variant | MODIFIER | *NFKBIL1* | Transcript | protein_coding |
| rs2523504 | 6:31510858-31510858 | C | upstream_gene_variant | MODIFIER | *NFKBIL1* | Transcript | protein_coding |
| rs2523504 | 6:31510858-31510858 | A | upstream_gene_variant | MODIFIER | *NFKBIL1* | Transcript | protein_coding |
| rs2523504 | 6:31510858-31510858 | C | upstream_gene_variant | MODIFIER | *NFKBIL1* | Transcript | protein_coding |
| rs2523504 | 6:31510858-31510858 | A | downstream_gene_variant | MODIFIER | *ATP6V1G2* | Transcript | protein_coding |
| rs2523504 | 6:31510858-31510858 | C | downstream_gene_variant | MODIFIER | *ATP6V1G2* | Transcript | protein_coding |
| rs2523504 | 6:31510858-31510858 | A | upstream_gene_variant | MODIFIER | *DDX39B* | Transcript | protein_coding |
| rs2523504 | 6:31510858-31510858 | C | upstream_gene_variant | MODIFIER | *DDX39B* | Transcript | protein_coding |
| rs2523504 | 6:31510858-31510858 | A | intron_variant,NMD_transcript_variant | MODIFIER | *ATP6V1G2-DDX39B* | Transcript | nonsense_mediated_decay |
| rs2523504 | 6:31510858-31510858 | C | intron_variant,NMD_transcript_variant | MODIFIER | *ATP6V1G2-DDX39B* | Transcript | nonsense_mediated_decay |
| rs2523504 | 6:31510858-31510858 | A | upstream_gene_variant | MODIFIER | *DDX39B* | Transcript | protein_coding |
| rs2523504 | 6:31510858-31510858 | C | upstream_gene_variant | MODIFIER | *DDX39B* | Transcript | protein_coding |
| rs2523504 | 6:31510858-31510858 | A | downstream_gene_variant | MODIFIER | *ATP6V1G2* | Transcript | protein_coding |
| rs2523504 | 6:31510858-31510858 | C | downstream_gene_variant | MODIFIER | *ATP6V1G2* | Transcript | protein_coding |
| rs2523504 | 6:31510858-31510858 | A | upstream_gene_variant | MODIFIER | *DDX39B* | Transcript | protein_coding |
| rs2523504 | 6:31510858-31510858 | C | upstream_gene_variant | MODIFIER | *DDX39B* | Transcript | protein_coding |
| rs2523504 | 6:31510858-31510858 | A | non_coding_transcript_exon_variant | MODIFIER | *DDX39B-AS1* | Transcript | antisense |
| rs2523504 | 6:31510858-31510858 | C | non_coding_transcript_exon_variant | MODIFIER | *DDX39B-AS1* | Transcript | antisense |
| rs2523504 | 6:31510858-31510858 | A | upstream_gene_variant | MODIFIER | *DDX39B* | Transcript | protein_coding |
| rs2523504 | 6:31510858-31510858 | C | upstream_gene_variant | MODIFIER | *DDX39B* | Transcript | protein_coding |
| rs2523504 | 6:31510858-31510858 | A | upstream_gene_variant | MODIFIER | *DDX39B* | Transcript | protein_coding |
| rs2523504 | 6:31510858-31510858 | C | upstream_gene_variant | MODIFIER | *DDX39B* | Transcript | protein_coding |
| rs2523504 | 6:31510858-31510858 | A | upstream_gene_variant | MODIFIER | *DDX39B* | Transcript | protein_coding |
| rs2523504 | 6:31510858-31510858 | C | upstream_gene_variant | MODIFIER | *DDX39B* | Transcript | protein_coding |
| rs2523504 | 6:31510858-31510858 | A | upstream_gene_variant | MODIFIER | *DDX39B* | Transcript | protein_coding |
| rs2523504 | 6:31510858-31510858 | C | upstream_gene_variant | MODIFIER | *DDX39B* | Transcript | protein_coding |
| rs2523504 | 6:31510858-31510858 | A | non_coding_transcript_exon_variant | MODIFIER | *DDX39B-AS1* | Transcript | antisense |
| rs2523504 | 6:31510858-31510858 | C | non_coding_transcript_exon_variant | MODIFIER | *DDX39B-AS1* | Transcript | antisense |
| rs2523504 | 6:31510858-31510858 | A | upstream_gene_variant | MODIFIER | *DDX39B* | Transcript | protein_coding |
| rs2523504 | 6:31510858-31510858 | C | upstream_gene_variant | MODIFIER | *DDX39B* | Transcript | protein_coding |
| rs2523504 | 6:31510858-31510858 | A | upstream_gene_variant | MODIFIER | *DDX39B* | Transcript | protein_coding |
| rs2523504 | 6:31510858-31510858 | C | upstream_gene_variant | MODIFIER | *DDX39B* | Transcript | protein_coding |
| rs2523504 | 6:31510858-31510858 | A | upstream_gene_variant | MODIFIER | *DDX39B* | Transcript | protein_coding |
| rs2523504 | 6:31510858-31510858 | C | upstream_gene_variant | MODIFIER | *DDX39B* | Transcript | protein_coding |
| rs2523504 | 6:31510858-31510858 | A | upstream_gene_variant | MODIFIER | *DDX39B* | Transcript | protein_coding |
| rs2523504 | 6:31510858-31510858 | C | upstream_gene_variant | MODIFIER | *DDX39B* | Transcript | protein_coding |
| rs2523504 | 6:31510858-31510858 | A | upstream_gene_variant | MODIFIER | *DDX39B* | Transcript | protein_coding |
| rs2523504 | 6:31510858-31510858 | C | upstream_gene_variant | MODIFIER | *DDX39B* | Transcript | protein_coding |
| rs2523504 | 6:31510858-31510858 | A | upstream_gene_variant | MODIFIER | *DDX39B* | Transcript | protein_coding |
| rs2523504 | 6:31510858-31510858 | C | upstream_gene_variant | MODIFIER | *DDX39B* | Transcript | protein_coding |
| rs2523504 | 6:31510858-31510858 | A | upstream_gene_variant | MODIFIER | *DDX39B* | Transcript | protein_coding |
| rs2523504 | 6:31510858-31510858 | C | upstream_gene_variant | MODIFIER | *DDX39B* | Transcript | protein_coding |
| rs2523504 | 6:31510858-31510858 | A | upstream_gene_variant | MODIFIER | *DDX39B* | Transcript | protein_coding |
| rs2523504 | 6:31510858-31510858 | C | upstream_gene_variant | MODIFIER | *DDX39B* | Transcript | protein_coding |
| rs2523504 | 6:31510858-31510858 | A | upstream_gene_variant | MODIFIER | *DDX39B* | Transcript | protein_coding |
| rs2523504 | 6:31510858-31510858 | C | upstream_gene_variant | MODIFIER | *DDX39B* | Transcript | protein_coding |
| rs2523504 | 6:31510858-31510858 | A | upstream_gene_variant | MODIFIER | *DDX39B* | Transcript | protein_coding |
| rs2523504 | 6:31510858-31510858 | C | upstream_gene_variant | MODIFIER | *DDX39B* | Transcript | protein_coding |
| rs2523504 | 6:31510858-31510858 | A | upstream_gene_variant | MODIFIER | *DDX39B* | Transcript | retained_intron |
| rs2523504 | 6:31510858-31510858 | C | upstream_gene_variant | MODIFIER | *DDX39B* | Transcript | retained_intron |
| rs2523504 | 6:31510858-31510858 | A | upstream_gene_variant | MODIFIER | *NFKBIL1* | Transcript | retained_intron |
| rs2523504 | 6:31510858-31510858 | C | upstream_gene_variant | MODIFIER | *NFKBIL1* | Transcript | retained_intron |
| rs2523504 | 6:31510858-31510858 | A | intron_variant,non_coding_transcript_variant | MODIFIER | *ATP6V1G2-DDX39B* | Transcript | processed_transcript |
| rs2523504 | 6:31510858-31510858 | C | intron_variant,non_coding_transcript_variant | MODIFIER | *ATP6V1G2-DDX39B* | Transcript | processed_transcript |
| rs2523504 | 6:31510858-31510858 | A | intron_variant,NMD_transcript_variant | MODIFIER | *ATP6V1G2-DDX39B* | Transcript | nonsense_mediated_decay |
| rs2523504 | 6:31510858-31510858 | C | intron_variant,NMD_transcript_variant | MODIFIER | *ATP6V1G2-DDX39B* | Transcript | nonsense_mediated_decay |
| rs2523504 | 6:31510858-31510858 | A | upstream_gene_variant | MODIFIER | *DDX39B* | Transcript | retained_intron |
| rs2523504 | 6:31510858-31510858 | C | upstream_gene_variant | MODIFIER | *DDX39B* | Transcript | retained_intron |
| rs2523504 | 6:31510858-31510858 | A | downstream_gene_variant | MODIFIER | *ATP6V1G2* | Transcript | retained_intron |
| rs2523504 | 6:31510858-31510858 | C | downstream_gene_variant | MODIFIER | *ATP6V1G2* | Transcript | retained_intron |
| rs2523504 | 6:31510858-31510858 | A | upstream_gene_variant | MODIFIER | *DDX39B* | Transcript | retained_intron |
| rs2523504 | 6:31510858-31510858 | C | upstream_gene_variant | MODIFIER | *DDX39B* | Transcript | retained_intron |
| rs2523504 | 6:31510858-31510858 | A | downstream_gene_variant | MODIFIER | *ATP6V1G2* | Transcript | processed_transcript |
| rs2523504 | 6:31510858-31510858 | C | downstream_gene_variant | MODIFIER | *ATP6V1G2* | Transcript | processed_transcript |
| rs2523504 | 6:31510858-31510858 | A | downstream_gene_variant | MODIFIER | *ATP6V1G2* | Transcript | protein_coding |
| rs2523504 | 6:31510858-31510858 | C | downstream_gene_variant | MODIFIER | *ATP6V1G2* | Transcript | protein_coding |
| rs2523504 | 6:31510858-31510858 | A | upstream_gene_variant | MODIFIER | *NFKBIL1* | Transcript | nonsense_mediated_decay |
| rs2523504 | 6:31510858-31510858 | C | upstream_gene_variant | MODIFIER | *NFKBIL1* | Transcript | nonsense_mediated_decay |
| rs2523504 | 6:31510858-31510858 | A | upstream_gene_variant | MODIFIER | *SNORD84* | Transcript | snoRNA |
| rs2523504 | 6:31510858-31510858 | C | upstream_gene_variant | MODIFIER | *SNORD84* | Transcript | snoRNA |
| rs2523504 | 6:31510858-31510858 | A | regulatory_region_variant | MODIFIER | *-* | RegulatoryFeature | promoter |
| rs2523504 | 6:31510858-31510858 | C | regulatory_region_variant | MODIFIER | *-* | RegulatoryFeature | promoter |
| rs2523504 | HSCHR6_MHC_COX:31498313-31498313 | T | intron_variant | MODIFIER | *DDX39B* | Transcript | protein_coding |
| rs2523504 | HSCHR6_MHC_COX:31498313-31498313 | A | intron_variant | MODIFIER | *DDX39B* | Transcript | protein_coding |
| rs2523504 | HSCHR6_MHC_COX:31498313-31498313 | T | upstream_gene_variant | MODIFIER | *NFKBIL1* | Transcript | protein_coding |
| rs2523504 | HSCHR6_MHC_COX:31498313-31498313 | A | upstream_gene_variant | MODIFIER | *NFKBIL1* | Transcript | protein_coding |
| rs2523504 | HSCHR6_MHC_COX:31498313-31498313 | T | upstream_gene_variant | MODIFIER | *DDX39B* | Transcript | protein_coding |
| rs2523504 | HSCHR6_MHC_COX:31498313-31498313 | A | upstream_gene_variant | MODIFIER | *DDX39B* | Transcript | protein_coding |
| rs2523504 | HSCHR6_MHC_COX:31498313-31498313 | T | upstream_gene_variant | MODIFIER | *DDX39B* | Transcript | protein_coding |
| rs2523504 | HSCHR6_MHC_COX:31498313-31498313 | A | upstream_gene_variant | MODIFIER | *DDX39B* | Transcript | protein_coding |
| rs2523504 | HSCHR6_MHC_COX:31498313-31498313 | T | upstream_gene_variant | MODIFIER | *DDX39B* | Transcript | protein_coding |
| rs2523504 | HSCHR6_MHC_COX:31498313-31498313 | A | upstream_gene_variant | MODIFIER | *DDX39B* | Transcript | protein_coding |
| rs2523504 | HSCHR6_MHC_COX:31498313-31498313 | T | upstream_gene_variant | MODIFIER | *NFKBIL1* | Transcript | protein_coding |
| rs2523504 | HSCHR6_MHC_COX:31498313-31498313 | A | upstream_gene_variant | MODIFIER | *NFKBIL1* | Transcript | protein_coding |
| rs2523504 | HSCHR6_MHC_COX:31498313-31498313 | T | upstream_gene_variant | MODIFIER | *DDX39B* | Transcript | protein_coding |
| rs2523504 | HSCHR6_MHC_COX:31498313-31498313 | A | upstream_gene_variant | MODIFIER | *DDX39B* | Transcript | protein_coding |
| rs2523504 | HSCHR6_MHC_COX:31498313-31498313 | T | upstream_gene_variant | MODIFIER | *DDX39B* | Transcript | protein_coding |
| rs2523504 | HSCHR6_MHC_COX:31498313-31498313 | A | upstream_gene_variant | MODIFIER | *DDX39B* | Transcript | protein_coding |
| rs2523504 | HSCHR6_MHC_COX:31498313-31498313 | T | non_coding_transcript_exon_variant | MODIFIER | *DDX39B-AS1* | Transcript | processed_transcript |
| rs2523504 | HSCHR6_MHC_COX:31498313-31498313 | A | non_coding_transcript_exon_variant | MODIFIER | *DDX39B-AS1* | Transcript | processed_transcript |
| rs2523504 | HSCHR6_MHC_COX:31498313-31498313 | T | upstream_gene_variant | MODIFIER | *DDX39B* | Transcript | protein_coding |
| rs2523504 | HSCHR6_MHC_COX:31498313-31498313 | A | upstream_gene_variant | MODIFIER | *DDX39B* | Transcript | protein_coding |
| rs2523504 | HSCHR6_MHC_COX:31498313-31498313 | T | upstream_gene_variant | MODIFIER | *DDX39B* | Transcript | protein_coding |
| rs2523504 | HSCHR6_MHC_COX:31498313-31498313 | A | upstream_gene_variant | MODIFIER | *DDX39B* | Transcript | protein_coding |
| rs2523504 | HSCHR6_MHC_COX:31498313-31498313 | T | non_coding_transcript_exon_variant | MODIFIER | *DDX39B-AS1* | Transcript | processed_transcript |
| rs2523504 | HSCHR6_MHC_COX:31498313-31498313 | A | non_coding_transcript_exon_variant | MODIFIER | *DDX39B-AS1* | Transcript | processed_transcript |
| rs2523504 | HSCHR6_MHC_COX:31498313-31498313 | T | upstream_gene_variant | MODIFIER | *NFKBIL1* | Transcript | protein_coding |
| rs2523504 | HSCHR6_MHC_COX:31498313-31498313 | A | upstream_gene_variant | MODIFIER | *NFKBIL1* | Transcript | protein_coding |
| rs2523504 | HSCHR6_MHC_COX:31498313-31498313 | T | upstream_gene_variant | MODIFIER | *DDX39B* | Transcript | protein_coding |
| rs2523504 | HSCHR6_MHC_COX:31498313-31498313 | A | upstream_gene_variant | MODIFIER | *DDX39B* | Transcript | protein_coding |
| rs2523504 | HSCHR6_MHC_COX:31498313-31498313 | T | upstream_gene_variant | MODIFIER | *DDX39B* | Transcript | protein_coding |
| rs2523504 | HSCHR6_MHC_COX:31498313-31498313 | A | upstream_gene_variant | MODIFIER | *DDX39B* | Transcript | protein_coding |
| rs2523504 | HSCHR6_MHC_COX:31498313-31498313 | T | upstream_gene_variant | MODIFIER | *DDX39B* | Transcript | protein_coding |
| rs2523504 | HSCHR6_MHC_COX:31498313-31498313 | A | upstream_gene_variant | MODIFIER | *DDX39B* | Transcript | protein_coding |
| rs2523504 | HSCHR6_MHC_COX:31498313-31498313 | T | downstream_gene_variant | MODIFIER | *ATP6V1G2* | Transcript | protein_coding |
| rs2523504 | HSCHR6_MHC_COX:31498313-31498313 | A | downstream_gene_variant | MODIFIER | *ATP6V1G2* | Transcript | protein_coding |
| rs2523504 | HSCHR6_MHC_COX:31498313-31498313 | T | upstream_gene_variant | MODIFIER | *DDX39B* | Transcript | protein_coding |
| rs2523504 | HSCHR6_MHC_COX:31498313-31498313 | A | upstream_gene_variant | MODIFIER | *DDX39B* | Transcript | protein_coding |
| rs2523504 | HSCHR6_MHC_COX:31498313-31498313 | T | downstream_gene_variant | MODIFIER | *ATP6V1G2* | Transcript | protein_coding |
| rs2523504 | HSCHR6_MHC_COX:31498313-31498313 | A | downstream_gene_variant | MODIFIER | *ATP6V1G2* | Transcript | protein_coding |
| rs2523504 | HSCHR6_MHC_COX:31498313-31498313 | T | upstream_gene_variant | MODIFIER | *DDX39B* | Transcript | protein_coding |
| rs2523504 | HSCHR6_MHC_COX:31498313-31498313 | A | upstream_gene_variant | MODIFIER | *DDX39B* | Transcript | protein_coding |
| rs2523504 | HSCHR6_MHC_COX:31498313-31498313 | T | upstream_gene_variant | MODIFIER | *DDX39B* | Transcript | protein_coding |
| rs2523504 | HSCHR6_MHC_COX:31498313-31498313 | A | upstream_gene_variant | MODIFIER | *DDX39B* | Transcript | protein_coding |
| rs2523504 | HSCHR6_MHC_COX:31498313-31498313 | T | upstream_gene_variant | MODIFIER | *DDX39B* | Transcript | protein_coding |
| rs2523504 | HSCHR6_MHC_COX:31498313-31498313 | A | upstream_gene_variant | MODIFIER | *DDX39B* | Transcript | protein_coding |
| rs2523504 | HSCHR6_MHC_COX:31498313-31498313 | T | downstream_gene_variant | MODIFIER | *ATP6V1G2* | Transcript | processed_transcript |
| rs2523504 | HSCHR6_MHC_COX:31498313-31498313 | A | downstream_gene_variant | MODIFIER | *ATP6V1G2* | Transcript | processed_transcript |
| rs2523504 | HSCHR6_MHC_COX:31498313-31498313 | T | upstream_gene_variant | MODIFIER | *DDX39B* | Transcript | protein_coding |
| rs2523504 | HSCHR6_MHC_COX:31498313-31498313 | A | upstream_gene_variant | MODIFIER | *DDX39B* | Transcript | protein_coding |
| rs2523504 | HSCHR6_MHC_COX:31498313-31498313 | T | upstream_gene_variant | MODIFIER | *NFKBIL1* | Transcript | protein_coding |
| rs2523504 | HSCHR6_MHC_COX:31498313-31498313 | A | upstream_gene_variant | MODIFIER | *NFKBIL1* | Transcript | protein_coding |
| rs2523504 | HSCHR6_MHC_COX:31498313-31498313 | T | downstream_gene_variant | MODIFIER | *ATP6V1G2* | Transcript | processed_transcript |
| rs2523504 | HSCHR6_MHC_COX:31498313-31498313 | A | downstream_gene_variant | MODIFIER | *ATP6V1G2* | Transcript | processed_transcript |
| rs2523504 | HSCHR6_MHC_COX:31498313-31498313 | T | upstream_gene_variant | MODIFIER | *DDX39B* | Transcript | retained_intron |
| rs2523504 | HSCHR6_MHC_COX:31498313-31498313 | A | upstream_gene_variant | MODIFIER | *DDX39B* | Transcript | retained_intron |
| rs2523504 | HSCHR6_MHC_COX:31498313-31498313 | T | upstream_gene_variant | MODIFIER | *DDX39B* | Transcript | retained_intron |
| rs2523504 | HSCHR6_MHC_COX:31498313-31498313 | A | upstream_gene_variant | MODIFIER | *DDX39B* | Transcript | retained_intron |
| rs2523504 | HSCHR6_MHC_COX:31498313-31498313 | T | upstream_gene_variant | MODIFIER | *NFKBIL1* | Transcript | processed_transcript |
| rs2523504 | HSCHR6_MHC_COX:31498313-31498313 | A | upstream_gene_variant | MODIFIER | *NFKBIL1* | Transcript | processed_transcript |
| rs2523504 | HSCHR6_MHC_COX:31498313-31498313 | T | upstream_gene_variant | MODIFIER | *DDX39B* | Transcript | protein_coding |
| rs2523504 | HSCHR6_MHC_COX:31498313-31498313 | A | upstream_gene_variant | MODIFIER | *DDX39B* | Transcript | protein_coding |
| rs2523504 | HSCHR6_MHC_COX:31498313-31498313 | T | upstream_gene_variant | MODIFIER | *DDX39B* | Transcript | protein_coding |
| rs2523504 | HSCHR6_MHC_COX:31498313-31498313 | A | upstream_gene_variant | MODIFIER | *DDX39B* | Transcript | protein_coding |
| rs2523504 | HSCHR6_MHC_COX:31498313-31498313 | T | upstream_gene_variant | MODIFIER | *SNORD83* | Transcript | snoRNA |
| rs2523504 | HSCHR6_MHC_COX:31498313-31498313 | A | upstream_gene_variant | MODIFIER | *SNORD83* | Transcript | snoRNA |
| rs2523504 | HSCHR6_MHC_DBB:31493039-31493039 | T | upstream_gene_variant | MODIFIER | *DDX39B* | Transcript | protein_coding |
| rs2523504 | HSCHR6_MHC_DBB:31493039-31493039 | A | upstream_gene_variant | MODIFIER | *DDX39B* | Transcript | protein_coding |
| rs2523504 | HSCHR6_MHC_DBB:31493039-31493039 | T | upstream_gene_variant | MODIFIER | *DDX39B* | Transcript | protein_coding |
| rs2523504 | HSCHR6_MHC_DBB:31493039-31493039 | A | upstream_gene_variant | MODIFIER | *DDX39B* | Transcript | protein_coding |
| rs2523504 | HSCHR6_MHC_DBB:31493039-31493039 | T | upstream_gene_variant | MODIFIER | *DDX39B* | Transcript | protein_coding |
| rs2523504 | HSCHR6_MHC_DBB:31493039-31493039 | A | upstream_gene_variant | MODIFIER | *DDX39B* | Transcript | protein_coding |
| rs2523504 | HSCHR6_MHC_DBB:31493039-31493039 | T | upstream_gene_variant | MODIFIER | *DDX39B* | Transcript | protein_coding |
| rs2523504 | HSCHR6_MHC_DBB:31493039-31493039 | A | upstream_gene_variant | MODIFIER | *DDX39B* | Transcript | protein_coding |
| rs2523504 | HSCHR6_MHC_DBB:31493039-31493039 | T | upstream_gene_variant | MODIFIER | *DDX39B* | Transcript | protein_coding |
| rs2523504 | HSCHR6_MHC_DBB:31493039-31493039 | A | upstream_gene_variant | MODIFIER | *DDX39B* | Transcript | protein_coding |
| rs2523504 | HSCHR6_MHC_DBB:31493039-31493039 | T | downstream_gene_variant | MODIFIER | *ATP6V1G2* | Transcript | protein_coding |
| rs2523504 | HSCHR6_MHC_DBB:31493039-31493039 | A | downstream_gene_variant | MODIFIER | *ATP6V1G2* | Transcript | protein_coding |
| rs2523504 | HSCHR6_MHC_DBB:31493039-31493039 | T | non_coding_transcript_exon_variant | MODIFIER | *DDX39B-AS1* | Transcript | processed_transcript |
| rs2523504 | HSCHR6_MHC_DBB:31493039-31493039 | A | non_coding_transcript_exon_variant | MODIFIER | *DDX39B-AS1* | Transcript | processed_transcript |
| rs2523504 | HSCHR6_MHC_DBB:31493039-31493039 | T | upstream_gene_variant | MODIFIER | *NFKBIL1* | Transcript | protein_coding |
| rs2523504 | HSCHR6_MHC_DBB:31493039-31493039 | A | upstream_gene_variant | MODIFIER | *NFKBIL1* | Transcript | protein_coding |
| rs2523504 | HSCHR6_MHC_DBB:31493039-31493039 | T | upstream_gene_variant | MODIFIER | *DDX39B* | Transcript | protein_coding |
| rs2523504 | HSCHR6_MHC_DBB:31493039-31493039 | A | upstream_gene_variant | MODIFIER | *DDX39B* | Transcript | protein_coding |
| rs2523504 | HSCHR6_MHC_DBB:31493039-31493039 | T | upstream_gene_variant | MODIFIER | *DDX39B* | Transcript | protein_coding |
| rs2523504 | HSCHR6_MHC_DBB:31493039-31493039 | A | upstream_gene_variant | MODIFIER | *DDX39B* | Transcript | protein_coding |
| rs2523504 | HSCHR6_MHC_DBB:31493039-31493039 | T | upstream_gene_variant | MODIFIER | *DDX39B* | Transcript | protein_coding |
| rs2523504 | HSCHR6_MHC_DBB:31493039-31493039 | A | upstream_gene_variant | MODIFIER | *DDX39B* | Transcript | protein_coding |
| rs2523504 | HSCHR6_MHC_DBB:31493039-31493039 | T | upstream_gene_variant | MODIFIER | *DDX39B* | Transcript | protein_coding |
| rs2523504 | HSCHR6_MHC_DBB:31493039-31493039 | A | upstream_gene_variant | MODIFIER | *DDX39B* | Transcript | protein_coding |
| rs2523504 | HSCHR6_MHC_DBB:31493039-31493039 | T | upstream_gene_variant | MODIFIER | *DDX39B* | Transcript | protein_coding |
| rs2523504 | HSCHR6_MHC_DBB:31493039-31493039 | A | upstream_gene_variant | MODIFIER | *DDX39B* | Transcript | protein_coding |
| rs2523504 | HSCHR6_MHC_DBB:31493039-31493039 | T | upstream_gene_variant | MODIFIER | *DDX39B* | Transcript | protein_coding |
| rs2523504 | HSCHR6_MHC_DBB:31493039-31493039 | A | upstream_gene_variant | MODIFIER | *DDX39B* | Transcript | protein_coding |
| rs2523504 | HSCHR6_MHC_DBB:31493039-31493039 | T | upstream_gene_variant | MODIFIER | *DDX39B* | Transcript | protein_coding |
| rs2523504 | HSCHR6_MHC_DBB:31493039-31493039 | A | upstream_gene_variant | MODIFIER | *DDX39B* | Transcript | protein_coding |
| rs2523504 | HSCHR6_MHC_DBB:31493039-31493039 | T | upstream_gene_variant | MODIFIER | *DDX39B* | Transcript | protein_coding |
| rs2523504 | HSCHR6_MHC_DBB:31493039-31493039 | A | upstream_gene_variant | MODIFIER | *DDX39B* | Transcript | protein_coding |
| rs2523504 | HSCHR6_MHC_DBB:31493039-31493039 | T | upstream_gene_variant | MODIFIER | *DDX39B* | Transcript | protein_coding |
| rs2523504 | HSCHR6_MHC_DBB:31493039-31493039 | A | upstream_gene_variant | MODIFIER | *DDX39B* | Transcript | protein_coding |
| rs2523504 | HSCHR6_MHC_DBB:31493039-31493039 | T | non_coding_transcript_exon_variant | MODIFIER | *DDX39B-AS1* | Transcript | processed_transcript |
| rs2523504 | HSCHR6_MHC_DBB:31493039-31493039 | A | non_coding_transcript_exon_variant | MODIFIER | *DDX39B-AS1* | Transcript | processed_transcript |
| rs2523504 | HSCHR6_MHC_DBB:31493039-31493039 | T | downstream_gene_variant | MODIFIER | *ATP6V1G2* | Transcript | protein_coding |
| rs2523504 | HSCHR6_MHC_DBB:31493039-31493039 | A | downstream_gene_variant | MODIFIER | *ATP6V1G2* | Transcript | protein_coding |
| rs2523504 | HSCHR6_MHC_DBB:31493039-31493039 | T | upstream_gene_variant | MODIFIER | *NFKBIL1* | Transcript | protein_coding |
| rs2523504 | HSCHR6_MHC_DBB:31493039-31493039 | A | upstream_gene_variant | MODIFIER | *NFKBIL1* | Transcript | protein_coding |
| rs2523504 | HSCHR6_MHC_DBB:31493039-31493039 | T | upstream_gene_variant | MODIFIER | *NFKBIL1* | Transcript | protein_coding |
| rs2523504 | HSCHR6_MHC_DBB:31493039-31493039 | A | upstream_gene_variant | MODIFIER | *NFKBIL1* | Transcript | protein_coding |
| rs2523504 | HSCHR6_MHC_DBB:31493039-31493039 | T | intron_variant | MODIFIER | *DDX39B* | Transcript | protein_coding |
| rs2523504 | HSCHR6_MHC_DBB:31493039-31493039 | A | intron_variant | MODIFIER | *DDX39B* | Transcript | protein_coding |
| rs2523504 | HSCHR6_MHC_DBB:31493039-31493039 | T | upstream_gene_variant | MODIFIER | *NFKBIL1* | Transcript | processed_transcript |
| rs2523504 | HSCHR6_MHC_DBB:31493039-31493039 | A | upstream_gene_variant | MODIFIER | *NFKBIL1* | Transcript | processed_transcript |
| rs2523504 | HSCHR6_MHC_DBB:31493039-31493039 | T | upstream_gene_variant | MODIFIER | *DDX39B* | Transcript | retained_intron |
| rs2523504 | HSCHR6_MHC_DBB:31493039-31493039 | A | upstream_gene_variant | MODIFIER | *DDX39B* | Transcript | retained_intron |
| rs2523504 | HSCHR6_MHC_DBB:31493039-31493039 | T | upstream_gene_variant | MODIFIER | *NFKBIL1* | Transcript | retained_intron |
| rs2523504 | HSCHR6_MHC_DBB:31493039-31493039 | A | upstream_gene_variant | MODIFIER | *NFKBIL1* | Transcript | retained_intron |
| rs2523504 | HSCHR6_MHC_DBB:31493039-31493039 | T | upstream_gene_variant | MODIFIER | *DDX39B* | Transcript | retained_intron |
| rs2523504 | HSCHR6_MHC_DBB:31493039-31493039 | A | upstream_gene_variant | MODIFIER | *DDX39B* | Transcript | retained_intron |
| rs2523504 | HSCHR6_MHC_DBB:31493039-31493039 | T | downstream_gene_variant | MODIFIER | *ATP6V1G2* | Transcript | processed_transcript |
| rs2523504 | HSCHR6_MHC_DBB:31493039-31493039 | A | downstream_gene_variant | MODIFIER | *ATP6V1G2* | Transcript | processed_transcript |
| rs2523504 | HSCHR6_MHC_DBB:31493039-31493039 | T | upstream_gene_variant | MODIFIER | *DDX39B* | Transcript | processed_transcript |
| rs2523504 | HSCHR6_MHC_DBB:31493039-31493039 | A | upstream_gene_variant | MODIFIER | *DDX39B* | Transcript | processed_transcript |
| rs2523504 | HSCHR6_MHC_DBB:31493039-31493039 | T | downstream_gene_variant | MODIFIER | *ATP6V1G2* | Transcript | processed_transcript |
| rs2523504 | HSCHR6_MHC_DBB:31493039-31493039 | A | downstream_gene_variant | MODIFIER | *ATP6V1G2* | Transcript | processed_transcript |
| rs2523504 | HSCHR6_MHC_DBB:31493039-31493039 | T | upstream_gene_variant | MODIFIER | *DDX39B* | Transcript | protein_coding |
| rs2523504 | HSCHR6_MHC_DBB:31493039-31493039 | A | upstream_gene_variant | MODIFIER | *DDX39B* | Transcript | protein_coding |
| rs2523504 | HSCHR6_MHC_DBB:31493039-31493039 | T | upstream_gene_variant | MODIFIER | *DDX39B* | Transcript | protein_coding |
| rs2523504 | HSCHR6_MHC_DBB:31493039-31493039 | A | upstream_gene_variant | MODIFIER | *DDX39B* | Transcript | protein_coding |
| rs2523504 | HSCHR6_MHC_DBB:31493039-31493039 | T | upstream_gene_variant | MODIFIER | *SNORD83* | Transcript | snoRNA |
| rs2523504 | HSCHR6_MHC_DBB:31493039-31493039 | A | upstream_gene_variant | MODIFIER | *SNORD83* | Transcript | snoRNA |
| rs2523504 | HSCHR6_MHC_MANN:31550357-31550357 | T | upstream_gene_variant | MODIFIER | *DDX39B* | Transcript | protein_coding |
| rs2523504 | HSCHR6_MHC_MANN:31550357-31550357 | A | upstream_gene_variant | MODIFIER | *DDX39B* | Transcript | protein_coding |
| rs2523504 | HSCHR6_MHC_MANN:31550357-31550357 | T | upstream_gene_variant | MODIFIER | *DDX39B* | Transcript | protein_coding |
| rs2523504 | HSCHR6_MHC_MANN:31550357-31550357 | A | upstream_gene_variant | MODIFIER | *DDX39B* | Transcript | protein_coding |
| rs2523504 | HSCHR6_MHC_MANN:31550357-31550357 | T | upstream_gene_variant | MODIFIER | *DDX39B* | Transcript | protein_coding |
| rs2523504 | HSCHR6_MHC_MANN:31550357-31550357 | A | upstream_gene_variant | MODIFIER | *DDX39B* | Transcript | protein_coding |
| rs2523504 | HSCHR6_MHC_MANN:31550357-31550357 | T | upstream_gene_variant | MODIFIER | *DDX39B* | Transcript | protein_coding |
| rs2523504 | HSCHR6_MHC_MANN:31550357-31550357 | A | upstream_gene_variant | MODIFIER | *DDX39B* | Transcript | protein_coding |
| rs2523504 | HSCHR6_MHC_MANN:31550357-31550357 | T | non_coding_transcript_exon_variant | MODIFIER | *DDX39B-AS1* | Transcript | processed_transcript |
| rs2523504 | HSCHR6_MHC_MANN:31550357-31550357 | A | non_coding_transcript_exon_variant | MODIFIER | *DDX39B-AS1* | Transcript | processed_transcript |
| rs2523504 | HSCHR6_MHC_MANN:31550357-31550357 | T | upstream_gene_variant | MODIFIER | *NFKBIL1* | Transcript | protein_coding |
| rs2523504 | HSCHR6_MHC_MANN:31550357-31550357 | A | upstream_gene_variant | MODIFIER | *NFKBIL1* | Transcript | protein_coding |
| rs2523504 | HSCHR6_MHC_MANN:31550357-31550357 | T | upstream_gene_variant | MODIFIER | *DDX39B* | Transcript | protein_coding |
| rs2523504 | HSCHR6_MHC_MANN:31550357-31550357 | A | upstream_gene_variant | MODIFIER | *DDX39B* | Transcript | protein_coding |
| rs2523504 | HSCHR6_MHC_MANN:31550357-31550357 | T | downstream_gene_variant | MODIFIER | *ATP6V1G2* | Transcript | protein_coding |
| rs2523504 | HSCHR6_MHC_MANN:31550357-31550357 | A | downstream_gene_variant | MODIFIER | *ATP6V1G2* | Transcript | protein_coding |
| rs2523504 | HSCHR6_MHC_MANN:31550357-31550357 | T | upstream_gene_variant | MODIFIER | *DDX39B* | Transcript | protein_coding |
| rs2523504 | HSCHR6_MHC_MANN:31550357-31550357 | A | upstream_gene_variant | MODIFIER | *DDX39B* | Transcript | protein_coding |
| rs2523504 | HSCHR6_MHC_MANN:31550357-31550357 | T | upstream_gene_variant | MODIFIER | *DDX39B* | Transcript | protein_coding |
| rs2523504 | HSCHR6_MHC_MANN:31550357-31550357 | A | upstream_gene_variant | MODIFIER | *DDX39B* | Transcript | protein_coding |
| rs2523504 | HSCHR6_MHC_MANN:31550357-31550357 | T | upstream_gene_variant | MODIFIER | *DDX39B* | Transcript | protein_coding |
| rs2523504 | HSCHR6_MHC_MANN:31550357-31550357 | A | upstream_gene_variant | MODIFIER | *DDX39B* | Transcript | protein_coding |
| rs2523504 | HSCHR6_MHC_MANN:31550357-31550357 | T | upstream_gene_variant | MODIFIER | *DDX39B* | Transcript | protein_coding |
| rs2523504 | HSCHR6_MHC_MANN:31550357-31550357 | A | upstream_gene_variant | MODIFIER | *DDX39B* | Transcript | protein_coding |
| rs2523504 | HSCHR6_MHC_MANN:31550357-31550357 | T | intron_variant | MODIFIER | *DDX39B* | Transcript | protein_coding |
| rs2523504 | HSCHR6_MHC_MANN:31550357-31550357 | A | intron_variant | MODIFIER | *DDX39B* | Transcript | protein_coding |
| rs2523504 | HSCHR6_MHC_MANN:31550357-31550357 | T | upstream_gene_variant | MODIFIER | *DDX39B* | Transcript | protein_coding |
| rs2523504 | HSCHR6_MHC_MANN:31550357-31550357 | A | upstream_gene_variant | MODIFIER | *DDX39B* | Transcript | protein_coding |
| rs2523504 | HSCHR6_MHC_MANN:31550357-31550357 | T | non_coding_transcript_exon_variant | MODIFIER | *DDX39B-AS1* | Transcript | processed_transcript |
| rs2523504 | HSCHR6_MHC_MANN:31550357-31550357 | A | non_coding_transcript_exon_variant | MODIFIER | *DDX39B-AS1* | Transcript | processed_transcript |
| rs2523504 | HSCHR6_MHC_MANN:31550357-31550357 | T | upstream_gene_variant | MODIFIER | *DDX39B* | Transcript | protein_coding |
| rs2523504 | HSCHR6_MHC_MANN:31550357-31550357 | A | upstream_gene_variant | MODIFIER | *DDX39B* | Transcript | protein_coding |
| rs2523504 | HSCHR6_MHC_MANN:31550357-31550357 | T | upstream_gene_variant | MODIFIER | *DDX39B* | Transcript | protein_coding |
| rs2523504 | HSCHR6_MHC_MANN:31550357-31550357 | A | upstream_gene_variant | MODIFIER | *DDX39B* | Transcript | protein_coding |
| rs2523504 | HSCHR6_MHC_MANN:31550357-31550357 | T | upstream_gene_variant | MODIFIER | *DDX39B* | Transcript | protein_coding |
| rs2523504 | HSCHR6_MHC_MANN:31550357-31550357 | A | upstream_gene_variant | MODIFIER | *DDX39B* | Transcript | protein_coding |
| rs2523504 | HSCHR6_MHC_MANN:31550357-31550357 | T | downstream_gene_variant | MODIFIER | *ATP6V1G2* | Transcript | protein_coding |
| rs2523504 | HSCHR6_MHC_MANN:31550357-31550357 | A | downstream_gene_variant | MODIFIER | *ATP6V1G2* | Transcript | protein_coding |
| rs2523504 | HSCHR6_MHC_MANN:31550357-31550357 | T | upstream_gene_variant | MODIFIER | *NFKBIL1* | Transcript | protein_coding |
| rs2523504 | HSCHR6_MHC_MANN:31550357-31550357 | A | upstream_gene_variant | MODIFIER | *NFKBIL1* | Transcript | protein_coding |
| rs2523504 | HSCHR6_MHC_MANN:31550357-31550357 | T | upstream_gene_variant | MODIFIER | *NFKBIL1* | Transcript | protein_coding |
| rs2523504 | HSCHR6_MHC_MANN:31550357-31550357 | A | upstream_gene_variant | MODIFIER | *NFKBIL1* | Transcript | protein_coding |
| rs2523504 | HSCHR6_MHC_MANN:31550357-31550357 | T | upstream_gene_variant | MODIFIER | *DDX39B* | Transcript | protein_coding |
| rs2523504 | HSCHR6_MHC_MANN:31550357-31550357 | A | upstream_gene_variant | MODIFIER | *DDX39B* | Transcript | protein_coding |
| rs2523504 | HSCHR6_MHC_MANN:31550357-31550357 | T | downstream_gene_variant | MODIFIER | *ATP6V1G2* | Transcript | processed_transcript |
| rs2523504 | HSCHR6_MHC_MANN:31550357-31550357 | A | downstream_gene_variant | MODIFIER | *ATP6V1G2* | Transcript | processed_transcript |
| rs2523504 | HSCHR6_MHC_MANN:31550357-31550357 | T | upstream_gene_variant | MODIFIER | *NFKBIL1* | Transcript | retained_intron |
| rs2523504 | HSCHR6_MHC_MANN:31550357-31550357 | A | upstream_gene_variant | MODIFIER | *NFKBIL1* | Transcript | retained_intron |
| rs2523504 | HSCHR6_MHC_MANN:31550357-31550357 | T | downstream_gene_variant | MODIFIER | *ATP6V1G2* | Transcript | processed_transcript |
| rs2523504 | HSCHR6_MHC_MANN:31550357-31550357 | A | downstream_gene_variant | MODIFIER | *ATP6V1G2* | Transcript | processed_transcript |
| rs2523504 | HSCHR6_MHC_MANN:31550357-31550357 | T | upstream_gene_variant | MODIFIER | *DDX39B* | Transcript | retained_intron |
| rs2523504 | HSCHR6_MHC_MANN:31550357-31550357 | A | upstream_gene_variant | MODIFIER | *DDX39B* | Transcript | retained_intron |
| rs2523504 | HSCHR6_MHC_MANN:31550357-31550357 | T | upstream_gene_variant | MODIFIER | *DDX39B* | Transcript | retained_intron |
| rs2523504 | HSCHR6_MHC_MANN:31550357-31550357 | A | upstream_gene_variant | MODIFIER | *DDX39B* | Transcript | retained_intron |
| rs2523504 | HSCHR6_MHC_MANN:31550357-31550357 | T | upstream_gene_variant | MODIFIER | *NFKBIL1* | Transcript | processed_transcript |
| rs2523504 | HSCHR6_MHC_MANN:31550357-31550357 | A | upstream_gene_variant | MODIFIER | *NFKBIL1* | Transcript | processed_transcript |
| rs2523504 | HSCHR6_MHC_MANN:31550357-31550357 | T | upstream_gene_variant | MODIFIER | *DDX39B* | Transcript | processed_transcript |
| rs2523504 | HSCHR6_MHC_MANN:31550357-31550357 | A | upstream_gene_variant | MODIFIER | *DDX39B* | Transcript | processed_transcript |
| rs2523504 | HSCHR6_MHC_MANN:31550357-31550357 | T | upstream_gene_variant | MODIFIER | *DDX39B* | Transcript | protein_coding |
| rs2523504 | HSCHR6_MHC_MANN:31550357-31550357 | A | upstream_gene_variant | MODIFIER | *DDX39B* | Transcript | protein_coding |
| rs2523504 | HSCHR6_MHC_MANN:31550357-31550357 | T | upstream_gene_variant | MODIFIER | *DDX39B* | Transcript | protein_coding |
| rs2523504 | HSCHR6_MHC_MANN:31550357-31550357 | A | upstream_gene_variant | MODIFIER | *DDX39B* | Transcript | protein_coding |
| rs2523504 | HSCHR6_MHC_MANN:31550357-31550357 | T | upstream_gene_variant | MODIFIER | *SNORD83* | Transcript | snoRNA |
| rs2523504 | HSCHR6_MHC_MANN:31550357-31550357 | A | upstream_gene_variant | MODIFIER | *SNORD83* | Transcript | snoRNA |
| rs2523504 | HSCHR6_MHC_MCF:31587208-31587208 | T | upstream_gene_variant | MODIFIER | *DDX39B* | Transcript | protein_coding |
| rs2523504 | HSCHR6_MHC_MCF:31587208-31587208 | A | upstream_gene_variant | MODIFIER | *DDX39B* | Transcript | protein_coding |
| rs2523504 | HSCHR6_MHC_MCF:31587208-31587208 | T | upstream_gene_variant | MODIFIER | *DDX39B* | Transcript | protein_coding |
| rs2523504 | HSCHR6_MHC_MCF:31587208-31587208 | A | upstream_gene_variant | MODIFIER | *DDX39B* | Transcript | protein_coding |
| rs2523504 | HSCHR6_MHC_MCF:31587208-31587208 | T | intron_variant | MODIFIER | *DDX39B* | Transcript | protein_coding |
| rs2523504 | HSCHR6_MHC_MCF:31587208-31587208 | A | intron_variant | MODIFIER | *DDX39B* | Transcript | protein_coding |
| rs2523504 | HSCHR6_MHC_MCF:31587208-31587208 | T | upstream_gene_variant | MODIFIER | *DDX39B* | Transcript | protein_coding |
| rs2523504 | HSCHR6_MHC_MCF:31587208-31587208 | A | upstream_gene_variant | MODIFIER | *DDX39B* | Transcript | protein_coding |
| rs2523504 | HSCHR6_MHC_MCF:31587208-31587208 | T | upstream_gene_variant | MODIFIER | *DDX39B* | Transcript | protein_coding |
| rs2523504 | HSCHR6_MHC_MCF:31587208-31587208 | A | upstream_gene_variant | MODIFIER | *DDX39B* | Transcript | protein_coding |
| rs2523504 | HSCHR6_MHC_MCF:31587208-31587208 | T | upstream_gene_variant | MODIFIER | *DDX39B* | Transcript | protein_coding |
| rs2523504 | HSCHR6_MHC_MCF:31587208-31587208 | A | upstream_gene_variant | MODIFIER | *DDX39B* | Transcript | protein_coding |
| rs2523504 | HSCHR6_MHC_MCF:31587208-31587208 | T | upstream_gene_variant | MODIFIER | *DDX39B* | Transcript | protein_coding |
| rs2523504 | HSCHR6_MHC_MCF:31587208-31587208 | A | upstream_gene_variant | MODIFIER | *DDX39B* | Transcript | protein_coding |
| rs2523504 | HSCHR6_MHC_MCF:31587208-31587208 | T | upstream_gene_variant | MODIFIER | *NFKBIL1* | Transcript | protein_coding |
| rs2523504 | HSCHR6_MHC_MCF:31587208-31587208 | A | upstream_gene_variant | MODIFIER | *NFKBIL1* | Transcript | protein_coding |
| rs2523504 | HSCHR6_MHC_MCF:31587208-31587208 | T | upstream_gene_variant | MODIFIER | *DDX39B* | Transcript | protein_coding |
| rs2523504 | HSCHR6_MHC_MCF:31587208-31587208 | A | upstream_gene_variant | MODIFIER | *DDX39B* | Transcript | protein_coding |
| rs2523504 | HSCHR6_MHC_MCF:31587208-31587208 | T | upstream_gene_variant | MODIFIER | *NFKBIL1* | Transcript | protein_coding |
| rs2523504 | HSCHR6_MHC_MCF:31587208-31587208 | A | upstream_gene_variant | MODIFIER | *NFKBIL1* | Transcript | protein_coding |
| rs2523504 | HSCHR6_MHC_MCF:31587208-31587208 | T | upstream_gene_variant | MODIFIER | *DDX39B* | Transcript | protein_coding |
| rs2523504 | HSCHR6_MHC_MCF:31587208-31587208 | A | upstream_gene_variant | MODIFIER | *DDX39B* | Transcript | protein_coding |
| rs2523504 | HSCHR6_MHC_MCF:31587208-31587208 | T | upstream_gene_variant | MODIFIER | *DDX39B* | Transcript | protein_coding |
| rs2523504 | HSCHR6_MHC_MCF:31587208-31587208 | A | upstream_gene_variant | MODIFIER | *DDX39B* | Transcript | protein_coding |
| rs2523504 | HSCHR6_MHC_MCF:31587208-31587208 | T | upstream_gene_variant | MODIFIER | *NFKBIL1* | Transcript | protein_coding |
| rs2523504 | HSCHR6_MHC_MCF:31587208-31587208 | A | upstream_gene_variant | MODIFIER | *NFKBIL1* | Transcript | protein_coding |
| rs2523504 | HSCHR6_MHC_MCF:31587208-31587208 | T | non_coding_transcript_exon_variant | MODIFIER | *DDX39B-AS1* | Transcript | processed_transcript |
| rs2523504 | HSCHR6_MHC_MCF:31587208-31587208 | A | non_coding_transcript_exon_variant | MODIFIER | *DDX39B-AS1* | Transcript | processed_transcript |
| rs2523504 | HSCHR6_MHC_MCF:31587208-31587208 | T | upstream_gene_variant | MODIFIER | *DDX39B* | Transcript | protein_coding |
| rs2523504 | HSCHR6_MHC_MCF:31587208-31587208 | A | upstream_gene_variant | MODIFIER | *DDX39B* | Transcript | protein_coding |
| rs2523504 | HSCHR6_MHC_MCF:31587208-31587208 | T | downstream_gene_variant | MODIFIER | *ATP6V1G2* | Transcript | protein_coding |
| rs2523504 | HSCHR6_MHC_MCF:31587208-31587208 | A | downstream_gene_variant | MODIFIER | *ATP6V1G2* | Transcript | protein_coding |
| rs2523504 | HSCHR6_MHC_MCF:31587208-31587208 | T | non_coding_transcript_exon_variant | MODIFIER | *DDX39B-AS1* | Transcript | processed_transcript |
| rs2523504 | HSCHR6_MHC_MCF:31587208-31587208 | A | non_coding_transcript_exon_variant | MODIFIER | *DDX39B-AS1* | Transcript | processed_transcript |
| rs2523504 | HSCHR6_MHC_MCF:31587208-31587208 | T | upstream_gene_variant | MODIFIER | *DDX39B* | Transcript | protein_coding |
| rs2523504 | HSCHR6_MHC_MCF:31587208-31587208 | A | upstream_gene_variant | MODIFIER | *DDX39B* | Transcript | protein_coding |
| rs2523504 | HSCHR6_MHC_MCF:31587208-31587208 | T | upstream_gene_variant | MODIFIER | *DDX39B* | Transcript | protein_coding |
| rs2523504 | HSCHR6_MHC_MCF:31587208-31587208 | A | upstream_gene_variant | MODIFIER | *DDX39B* | Transcript | protein_coding |
| rs2523504 | HSCHR6_MHC_MCF:31587208-31587208 | T | upstream_gene_variant | MODIFIER | *DDX39B* | Transcript | protein_coding |
| rs2523504 | HSCHR6_MHC_MCF:31587208-31587208 | A | upstream_gene_variant | MODIFIER | *DDX39B* | Transcript | protein_coding |
| rs2523504 | HSCHR6_MHC_MCF:31587208-31587208 | T | downstream_gene_variant | MODIFIER | *ATP6V1G2* | Transcript | protein_coding |
| rs2523504 | HSCHR6_MHC_MCF:31587208-31587208 | A | downstream_gene_variant | MODIFIER | *ATP6V1G2* | Transcript | protein_coding |
| rs2523504 | HSCHR6_MHC_MCF:31587208-31587208 | T | upstream_gene_variant | MODIFIER | *DDX39B* | Transcript | protein_coding |
| rs2523504 | HSCHR6_MHC_MCF:31587208-31587208 | A | upstream_gene_variant | MODIFIER | *DDX39B* | Transcript | protein_coding |
| rs2523504 | HSCHR6_MHC_MCF:31587208-31587208 | T | upstream_gene_variant | MODIFIER | *NFKBIL1* | Transcript | processed_transcript |
| rs2523504 | HSCHR6_MHC_MCF:31587208-31587208 | A | upstream_gene_variant | MODIFIER | *NFKBIL1* | Transcript | processed_transcript |
| rs2523504 | HSCHR6_MHC_MCF:31587208-31587208 | T | upstream_gene_variant | MODIFIER | *DDX39B* | Transcript | processed_transcript |
| rs2523504 | HSCHR6_MHC_MCF:31587208-31587208 | A | upstream_gene_variant | MODIFIER | *DDX39B* | Transcript | processed_transcript |
| rs2523504 | HSCHR6_MHC_MCF:31587208-31587208 | T | downstream_gene_variant | MODIFIER | *ATP6V1G2* | Transcript | processed_transcript |
| rs2523504 | HSCHR6_MHC_MCF:31587208-31587208 | A | downstream_gene_variant | MODIFIER | *ATP6V1G2* | Transcript | processed_transcript |
| rs2523504 | HSCHR6_MHC_MCF:31587208-31587208 | T | upstream_gene_variant | MODIFIER | *NFKBIL1* | Transcript | retained_intron |
| rs2523504 | HSCHR6_MHC_MCF:31587208-31587208 | A | upstream_gene_variant | MODIFIER | *NFKBIL1* | Transcript | retained_intron |
| rs2523504 | HSCHR6_MHC_MCF:31587208-31587208 | T | downstream_gene_variant | MODIFIER | *ATP6V1G2* | Transcript | processed_transcript |
| rs2523504 | HSCHR6_MHC_MCF:31587208-31587208 | A | downstream_gene_variant | MODIFIER | *ATP6V1G2* | Transcript | processed_transcript |
| rs2523504 | HSCHR6_MHC_MCF:31587208-31587208 | T | upstream_gene_variant | MODIFIER | *DDX39B* | Transcript | retained_intron |
| rs2523504 | HSCHR6_MHC_MCF:31587208-31587208 | A | upstream_gene_variant | MODIFIER | *DDX39B* | Transcript | retained_intron |
| rs2523504 | HSCHR6_MHC_MCF:31587208-31587208 | T | upstream_gene_variant | MODIFIER | *DDX39B* | Transcript | retained_intron |
| rs2523504 | HSCHR6_MHC_MCF:31587208-31587208 | A | upstream_gene_variant | MODIFIER | *DDX39B* | Transcript | retained_intron |
| rs2523504 | HSCHR6_MHC_MCF:31587208-31587208 | T | upstream_gene_variant | MODIFIER | *DDX39B* | Transcript | protein_coding |
| rs2523504 | HSCHR6_MHC_MCF:31587208-31587208 | A | upstream_gene_variant | MODIFIER | *DDX39B* | Transcript | protein_coding |
| rs2523504 | HSCHR6_MHC_MCF:31587208-31587208 | T | upstream_gene_variant | MODIFIER | *DDX39B* | Transcript | protein_coding |
| rs2523504 | HSCHR6_MHC_MCF:31587208-31587208 | A | upstream_gene_variant | MODIFIER | *DDX39B* | Transcript | protein_coding |
| rs2523504 | HSCHR6_MHC_MCF:31587208-31587208 | T | downstream_gene_variant | MODIFIER | *ATP6V1G2* | Transcript | protein_coding |
| rs2523504 | HSCHR6_MHC_MCF:31587208-31587208 | A | downstream_gene_variant | MODIFIER | *ATP6V1G2* | Transcript | protein_coding |
| rs2523504 | HSCHR6_MHC_MCF:31587208-31587208 | T | upstream_gene_variant | MODIFIER | *SNORD83* | Transcript | snoRNA |
| rs2523504 | HSCHR6_MHC_MCF:31587208-31587208 | A | upstream_gene_variant | MODIFIER | *SNORD83* | Transcript | snoRNA |
| rs2523504 | HSCHR6_MHC_QBL:31501136-31501136 | T | upstream_gene_variant | MODIFIER | *NFKBIL1* | Transcript | protein_coding |
| rs2523504 | HSCHR6_MHC_QBL:31501136-31501136 | A | upstream_gene_variant | MODIFIER | *NFKBIL1* | Transcript | protein_coding |
| rs2523504 | HSCHR6_MHC_QBL:31501136-31501136 | T | downstream_gene_variant | MODIFIER | *ATP6V1G2* | Transcript | protein_coding |
| rs2523504 | HSCHR6_MHC_QBL:31501136-31501136 | A | downstream_gene_variant | MODIFIER | *ATP6V1G2* | Transcript | protein_coding |
| rs2523504 | HSCHR6_MHC_QBL:31501136-31501136 | T | intron_variant | MODIFIER | *DDX39B* | Transcript | protein_coding |
| rs2523504 | HSCHR6_MHC_QBL:31501136-31501136 | A | intron_variant | MODIFIER | *DDX39B* | Transcript | protein_coding |
| rs2523504 | HSCHR6_MHC_QBL:31501136-31501136 | T | upstream_gene_variant | MODIFIER | *NFKBIL1* | Transcript | protein_coding |
| rs2523504 | HSCHR6_MHC_QBL:31501136-31501136 | A | upstream_gene_variant | MODIFIER | *NFKBIL1* | Transcript | protein_coding |
| rs2523504 | HSCHR6_MHC_QBL:31501136-31501136 | T | upstream_gene_variant | MODIFIER | *NFKBIL1* | Transcript | protein_coding |
| rs2523504 | HSCHR6_MHC_QBL:31501136-31501136 | A | upstream_gene_variant | MODIFIER | *NFKBIL1* | Transcript | protein_coding |
| rs2523504 | HSCHR6_MHC_QBL:31501136-31501136 | T | upstream_gene_variant | MODIFIER | *DDX39B* | Transcript | protein_coding |
| rs2523504 | HSCHR6_MHC_QBL:31501136-31501136 | A | upstream_gene_variant | MODIFIER | *DDX39B* | Transcript | protein_coding |
| rs2523504 | HSCHR6_MHC_QBL:31501136-31501136 | T | upstream_gene_variant | MODIFIER | *DDX39B* | Transcript | protein_coding |
| rs2523504 | HSCHR6_MHC_QBL:31501136-31501136 | A | upstream_gene_variant | MODIFIER | *DDX39B* | Transcript | protein_coding |
| rs2523504 | HSCHR6_MHC_QBL:31501136-31501136 | T | upstream_gene_variant | MODIFIER | *DDX39B* | Transcript | protein_coding |
| rs2523504 | HSCHR6_MHC_QBL:31501136-31501136 | A | upstream_gene_variant | MODIFIER | *DDX39B* | Transcript | protein_coding |
| rs2523504 | HSCHR6_MHC_QBL:31501136-31501136 | T | non_coding_transcript_exon_variant | MODIFIER | *DDX39B-AS1* | Transcript | processed_transcript |
| rs2523504 | HSCHR6_MHC_QBL:31501136-31501136 | A | non_coding_transcript_exon_variant | MODIFIER | *DDX39B-AS1* | Transcript | processed_transcript |
| rs2523504 | HSCHR6_MHC_QBL:31501136-31501136 | T | non_coding_transcript_exon_variant | MODIFIER | *DDX39B-AS1* | Transcript | processed_transcript |
| rs2523504 | HSCHR6_MHC_QBL:31501136-31501136 | A | non_coding_transcript_exon_variant | MODIFIER | *DDX39B-AS1* | Transcript | processed_transcript |
| rs2523504 | HSCHR6_MHC_QBL:31501136-31501136 | T | upstream_gene_variant | MODIFIER | *DDX39B* | Transcript | protein_coding |
| rs2523504 | HSCHR6_MHC_QBL:31501136-31501136 | A | upstream_gene_variant | MODIFIER | *DDX39B* | Transcript | protein_coding |
| rs2523504 | HSCHR6_MHC_QBL:31501136-31501136 | T | upstream_gene_variant | MODIFIER | *DDX39B* | Transcript | protein_coding |
| rs2523504 | HSCHR6_MHC_QBL:31501136-31501136 | A | upstream_gene_variant | MODIFIER | *DDX39B* | Transcript | protein_coding |
| rs2523504 | HSCHR6_MHC_QBL:31501136-31501136 | T | upstream_gene_variant | MODIFIER | *DDX39B* | Transcript | protein_coding |
| rs2523504 | HSCHR6_MHC_QBL:31501136-31501136 | A | upstream_gene_variant | MODIFIER | *DDX39B* | Transcript | protein_coding |
| rs2523504 | HSCHR6_MHC_QBL:31501136-31501136 | T | upstream_gene_variant | MODIFIER | *DDX39B* | Transcript | protein_coding |
| rs2523504 | HSCHR6_MHC_QBL:31501136-31501136 | A | upstream_gene_variant | MODIFIER | *DDX39B* | Transcript | protein_coding |
| rs2523504 | HSCHR6_MHC_QBL:31501136-31501136 | T | upstream_gene_variant | MODIFIER | *DDX39B* | Transcript | protein_coding |
| rs2523504 | HSCHR6_MHC_QBL:31501136-31501136 | A | upstream_gene_variant | MODIFIER | *DDX39B* | Transcript | protein_coding |
| rs2523504 | HSCHR6_MHC_QBL:31501136-31501136 | T | upstream_gene_variant | MODIFIER | *DDX39B* | Transcript | protein_coding |
| rs2523504 | HSCHR6_MHC_QBL:31501136-31501136 | A | upstream_gene_variant | MODIFIER | *DDX39B* | Transcript | protein_coding |
| rs2523504 | HSCHR6_MHC_QBL:31501136-31501136 | T | upstream_gene_variant | MODIFIER | *DDX39B* | Transcript | protein_coding |
| rs2523504 | HSCHR6_MHC_QBL:31501136-31501136 | A | upstream_gene_variant | MODIFIER | *DDX39B* | Transcript | protein_coding |
| rs2523504 | HSCHR6_MHC_QBL:31501136-31501136 | T | upstream_gene_variant | MODIFIER | *DDX39B* | Transcript | protein_coding |
| rs2523504 | HSCHR6_MHC_QBL:31501136-31501136 | A | upstream_gene_variant | MODIFIER | *DDX39B* | Transcript | protein_coding |
| rs2523504 | HSCHR6_MHC_QBL:31501136-31501136 | T | upstream_gene_variant | MODIFIER | *DDX39B* | Transcript | protein_coding |
| rs2523504 | HSCHR6_MHC_QBL:31501136-31501136 | A | upstream_gene_variant | MODIFIER | *DDX39B* | Transcript | protein_coding |
| rs2523504 | HSCHR6_MHC_QBL:31501136-31501136 | T | upstream_gene_variant | MODIFIER | *DDX39B* | Transcript | protein_coding |
| rs2523504 | HSCHR6_MHC_QBL:31501136-31501136 | A | upstream_gene_variant | MODIFIER | *DDX39B* | Transcript | protein_coding |
| rs2523504 | HSCHR6_MHC_QBL:31501136-31501136 | T | upstream_gene_variant | MODIFIER | *DDX39B* | Transcript | protein_coding |
| rs2523504 | HSCHR6_MHC_QBL:31501136-31501136 | A | upstream_gene_variant | MODIFIER | *DDX39B* | Transcript | protein_coding |
| rs2523504 | HSCHR6_MHC_QBL:31501136-31501136 | T | downstream_gene_variant | MODIFIER | *ATP6V1G2* | Transcript | protein_coding |
| rs2523504 | HSCHR6_MHC_QBL:31501136-31501136 | A | downstream_gene_variant | MODIFIER | *ATP6V1G2* | Transcript | protein_coding |
| rs2523504 | HSCHR6_MHC_QBL:31501136-31501136 | T | upstream_gene_variant | MODIFIER | *DDX39B* | Transcript | retained_intron |
| rs2523504 | HSCHR6_MHC_QBL:31501136-31501136 | A | upstream_gene_variant | MODIFIER | *DDX39B* | Transcript | retained_intron |
| rs2523504 | HSCHR6_MHC_QBL:31501136-31501136 | T | upstream_gene_variant | MODIFIER | *NFKBIL1* | Transcript | processed_transcript |
| rs2523504 | HSCHR6_MHC_QBL:31501136-31501136 | A | upstream_gene_variant | MODIFIER | *NFKBIL1* | Transcript | processed_transcript |
| rs2523504 | HSCHR6_MHC_QBL:31501136-31501136 | T | downstream_gene_variant | MODIFIER | *ATP6V1G2* | Transcript | processed_transcript |
| rs2523504 | HSCHR6_MHC_QBL:31501136-31501136 | A | downstream_gene_variant | MODIFIER | *ATP6V1G2* | Transcript | processed_transcript |
| rs2523504 | HSCHR6_MHC_QBL:31501136-31501136 | T | downstream_gene_variant | MODIFIER | *ATP6V1G2* | Transcript | processed_transcript |
| rs2523504 | HSCHR6_MHC_QBL:31501136-31501136 | A | downstream_gene_variant | MODIFIER | *ATP6V1G2* | Transcript | processed_transcript |
| rs2523504 | HSCHR6_MHC_QBL:31501136-31501136 | T | upstream_gene_variant | MODIFIER | *DDX39B* | Transcript | retained_intron |
| rs2523504 | HSCHR6_MHC_QBL:31501136-31501136 | A | upstream_gene_variant | MODIFIER | *DDX39B* | Transcript | retained_intron |
| rs2523504 | HSCHR6_MHC_QBL:31501136-31501136 | T | upstream_gene_variant | MODIFIER | *NFKBIL1* | Transcript | retained_intron |
| rs2523504 | HSCHR6_MHC_QBL:31501136-31501136 | A | upstream_gene_variant | MODIFIER | *NFKBIL1* | Transcript | retained_intron |
| rs2523504 | HSCHR6_MHC_QBL:31501136-31501136 | T | upstream_gene_variant | MODIFIER | *DDX39B* | Transcript | processed_transcript |
| rs2523504 | HSCHR6_MHC_QBL:31501136-31501136 | A | upstream_gene_variant | MODIFIER | *DDX39B* | Transcript | processed_transcript |
| rs2523504 | HSCHR6_MHC_QBL:31501136-31501136 | T | upstream_gene_variant | MODIFIER | *DDX39B* | Transcript | protein_coding |
| rs2523504 | HSCHR6_MHC_QBL:31501136-31501136 | A | upstream_gene_variant | MODIFIER | *DDX39B* | Transcript | protein_coding |
| rs2523504 | HSCHR6_MHC_QBL:31501136-31501136 | T | upstream_gene_variant | MODIFIER | *DDX39B* | Transcript | protein_coding |
| rs2523504 | HSCHR6_MHC_QBL:31501136-31501136 | A | upstream_gene_variant | MODIFIER | *DDX39B* | Transcript | protein_coding |
| rs2523504 | HSCHR6_MHC_QBL:31501136-31501136 | T | upstream_gene_variant | MODIFIER | *SNORD83* | Transcript | snoRNA |
| rs2523504 | HSCHR6_MHC_QBL:31501136-31501136 | A | upstream_gene_variant | MODIFIER | *SNORD83* | Transcript | snoRNA |
| rs2523504 | HSCHR6_MHC_SSTO:31500805-31500805 | A | upstream_gene_variant | MODIFIER | *DDX39B* | Transcript | protein_coding |
| rs2523504 | HSCHR6_MHC_SSTO:31500805-31500805 | C | upstream_gene_variant | MODIFIER | *DDX39B* | Transcript | protein_coding |
| rs2523504 | HSCHR6_MHC_SSTO:31500805-31500805 | A | upstream_gene_variant | MODIFIER | *NFKBIL1* | Transcript | protein_coding |
| rs2523504 | HSCHR6_MHC_SSTO:31500805-31500805 | C | upstream_gene_variant | MODIFIER | *NFKBIL1* | Transcript | protein_coding |
| rs2523504 | HSCHR6_MHC_SSTO:31500805-31500805 | A | upstream_gene_variant | MODIFIER | *DDX39B* | Transcript | protein_coding |
| rs2523504 | HSCHR6_MHC_SSTO:31500805-31500805 | C | upstream_gene_variant | MODIFIER | *DDX39B* | Transcript | protein_coding |
| rs2523504 | HSCHR6_MHC_SSTO:31500805-31500805 | A | upstream_gene_variant | MODIFIER | *NFKBIL1* | Transcript | protein_coding |
| rs2523504 | HSCHR6_MHC_SSTO:31500805-31500805 | C | upstream_gene_variant | MODIFIER | *NFKBIL1* | Transcript | protein_coding |
| rs2523504 | HSCHR6_MHC_SSTO:31500805-31500805 | A | non_coding_transcript_exon_variant | MODIFIER | *DDX39B-AS1* | Transcript | processed_transcript |
| rs2523504 | HSCHR6_MHC_SSTO:31500805-31500805 | C | non_coding_transcript_exon_variant | MODIFIER | *DDX39B-AS1* | Transcript | processed_transcript |
| rs2523504 | HSCHR6_MHC_SSTO:31500805-31500805 | A | upstream_gene_variant | MODIFIER | *DDX39B* | Transcript | protein_coding |
| rs2523504 | HSCHR6_MHC_SSTO:31500805-31500805 | C | upstream_gene_variant | MODIFIER | *DDX39B* | Transcript | protein_coding |
| rs2523504 | HSCHR6_MHC_SSTO:31500805-31500805 | A | upstream_gene_variant | MODIFIER | *DDX39B* | Transcript | protein_coding |
| rs2523504 | HSCHR6_MHC_SSTO:31500805-31500805 | C | upstream_gene_variant | MODIFIER | *DDX39B* | Transcript | protein_coding |
| rs2523504 | HSCHR6_MHC_SSTO:31500805-31500805 | A | upstream_gene_variant | MODIFIER | *NFKBIL1* | Transcript | protein_coding |
| rs2523504 | HSCHR6_MHC_SSTO:31500805-31500805 | C | upstream_gene_variant | MODIFIER | *NFKBIL1* | Transcript | protein_coding |
| rs2523504 | HSCHR6_MHC_SSTO:31500805-31500805 | A | upstream_gene_variant | MODIFIER | *DDX39B* | Transcript | protein_coding |
| rs2523504 | HSCHR6_MHC_SSTO:31500805-31500805 | C | upstream_gene_variant | MODIFIER | *DDX39B* | Transcript | protein_coding |
| rs2523504 | HSCHR6_MHC_SSTO:31500805-31500805 | A | upstream_gene_variant | MODIFIER | *DDX39B* | Transcript | protein_coding |
| rs2523504 | HSCHR6_MHC_SSTO:31500805-31500805 | C | upstream_gene_variant | MODIFIER | *DDX39B* | Transcript | protein_coding |
| rs2523504 | HSCHR6_MHC_SSTO:31500805-31500805 | A | upstream_gene_variant | MODIFIER | *DDX39B* | Transcript | protein_coding |
| rs2523504 | HSCHR6_MHC_SSTO:31500805-31500805 | C | upstream_gene_variant | MODIFIER | *DDX39B* | Transcript | protein_coding |
| rs2523504 | HSCHR6_MHC_SSTO:31500805-31500805 | A | upstream_gene_variant | MODIFIER | *DDX39B* | Transcript | protein_coding |
| rs2523504 | HSCHR6_MHC_SSTO:31500805-31500805 | C | upstream_gene_variant | MODIFIER | *DDX39B* | Transcript | protein_coding |
| rs2523504 | HSCHR6_MHC_SSTO:31500805-31500805 | A | downstream_gene_variant | MODIFIER | *ATP6V1G2* | Transcript | protein_coding |
| rs2523504 | HSCHR6_MHC_SSTO:31500805-31500805 | C | downstream_gene_variant | MODIFIER | *ATP6V1G2* | Transcript | protein_coding |
| rs2523504 | HSCHR6_MHC_SSTO:31500805-31500805 | A | upstream_gene_variant | MODIFIER | *DDX39B* | Transcript | protein_coding |
| rs2523504 | HSCHR6_MHC_SSTO:31500805-31500805 | C | upstream_gene_variant | MODIFIER | *DDX39B* | Transcript | protein_coding |
| rs2523504 | HSCHR6_MHC_SSTO:31500805-31500805 | A | upstream_gene_variant | MODIFIER | *DDX39B* | Transcript | protein_coding |
| rs2523504 | HSCHR6_MHC_SSTO:31500805-31500805 | C | upstream_gene_variant | MODIFIER | *DDX39B* | Transcript | protein_coding |
| rs2523504 | HSCHR6_MHC_SSTO:31500805-31500805 | A | downstream_gene_variant | MODIFIER | *ATP6V1G2* | Transcript | protein_coding |
| rs2523504 | HSCHR6_MHC_SSTO:31500805-31500805 | C | downstream_gene_variant | MODIFIER | *ATP6V1G2* | Transcript | protein_coding |
| rs2523504 | HSCHR6_MHC_SSTO:31500805-31500805 | A | upstream_gene_variant | MODIFIER | *DDX39B* | Transcript | protein_coding |
| rs2523504 | HSCHR6_MHC_SSTO:31500805-31500805 | C | upstream_gene_variant | MODIFIER | *DDX39B* | Transcript | protein_coding |
| rs2523504 | HSCHR6_MHC_SSTO:31500805-31500805 | A | upstream_gene_variant | MODIFIER | *DDX39B* | Transcript | protein_coding |
| rs2523504 | HSCHR6_MHC_SSTO:31500805-31500805 | C | upstream_gene_variant | MODIFIER | *DDX39B* | Transcript | protein_coding |
| rs2523504 | HSCHR6_MHC_SSTO:31500805-31500805 | A | intron_variant,NMD_transcript_variant | MODIFIER | *DASS-161H22.6* | Transcript | nonsense_mediated_decay |
| rs2523504 | HSCHR6_MHC_SSTO:31500805-31500805 | C | intron_variant,NMD_transcript_variant | MODIFIER | *DASS-161H22.6* | Transcript | nonsense_mediated_decay |
| rs2523504 | HSCHR6_MHC_SSTO:31500805-31500805 | A | upstream_gene_variant | MODIFIER | *DDX39B* | Transcript | protein_coding |
| rs2523504 | HSCHR6_MHC_SSTO:31500805-31500805 | C | upstream_gene_variant | MODIFIER | *DDX39B* | Transcript | protein_coding |
| rs2523504 | HSCHR6_MHC_SSTO:31500805-31500805 | A | non_coding_transcript_exon_variant | MODIFIER | *DDX39B-AS1* | Transcript | processed_transcript |
| rs2523504 | HSCHR6_MHC_SSTO:31500805-31500805 | C | non_coding_transcript_exon_variant | MODIFIER | *DDX39B-AS1* | Transcript | processed_transcript |
| rs2523504 | HSCHR6_MHC_SSTO:31500805-31500805 | A | upstream_gene_variant | MODIFIER | *DDX39B* | Transcript | protein_coding |
| rs2523504 | HSCHR6_MHC_SSTO:31500805-31500805 | C | upstream_gene_variant | MODIFIER | *DDX39B* | Transcript | protein_coding |
| rs2523504 | HSCHR6_MHC_SSTO:31500805-31500805 | A | upstream_gene_variant | MODIFIER | *NFKBIL1* | Transcript | nonsense_mediated_decay |
| rs2523504 | HSCHR6_MHC_SSTO:31500805-31500805 | C | upstream_gene_variant | MODIFIER | *NFKBIL1* | Transcript | nonsense_mediated_decay |
| rs2523504 | HSCHR6_MHC_SSTO:31500805-31500805 | A | downstream_gene_variant | MODIFIER | *ATP6V1G2* | Transcript | processed_transcript |
| rs2523504 | HSCHR6_MHC_SSTO:31500805-31500805 | C | downstream_gene_variant | MODIFIER | *ATP6V1G2* | Transcript | processed_transcript |
| rs2523504 | HSCHR6_MHC_SSTO:31500805-31500805 | A | downstream_gene_variant | MODIFIER | *ATP6V1G2* | Transcript | processed_transcript |
| rs2523504 | HSCHR6_MHC_SSTO:31500805-31500805 | C | downstream_gene_variant | MODIFIER | *ATP6V1G2* | Transcript | processed_transcript |
| rs2523504 | HSCHR6_MHC_SSTO:31500805-31500805 | A | upstream_gene_variant | MODIFIER | *NFKBIL1* | Transcript | retained_intron |
| rs2523504 | HSCHR6_MHC_SSTO:31500805-31500805 | C | upstream_gene_variant | MODIFIER | *NFKBIL1* | Transcript | retained_intron |
| rs2523504 | HSCHR6_MHC_SSTO:31500805-31500805 | A | upstream_gene_variant | MODIFIER | *DDX39B* | Transcript | retained_intron |
| rs2523504 | HSCHR6_MHC_SSTO:31500805-31500805 | C | upstream_gene_variant | MODIFIER | *DDX39B* | Transcript | retained_intron |
| rs2523504 | HSCHR6_MHC_SSTO:31500805-31500805 | A | upstream_gene_variant | MODIFIER | *DDX39B* | Transcript | retained_intron |
| rs2523504 | HSCHR6_MHC_SSTO:31500805-31500805 | C | upstream_gene_variant | MODIFIER | *DDX39B* | Transcript | retained_intron |
| rs2523504 | HSCHR6_MHC_SSTO:31500805-31500805 | A | upstream_gene_variant | MODIFIER | *DDX39B* | Transcript | retained_intron |
| rs2523504 | HSCHR6_MHC_SSTO:31500805-31500805 | C | upstream_gene_variant | MODIFIER | *DDX39B* | Transcript | retained_intron |
| rs2523504 | HSCHR6_MHC_SSTO:31500805-31500805 | A | upstream_gene_variant | MODIFIER | *DDX39B* | Transcript | protein_coding |
| rs2523504 | HSCHR6_MHC_SSTO:31500805-31500805 | C | upstream_gene_variant | MODIFIER | *DDX39B* | Transcript | protein_coding |
| rs2523504 | HSCHR6_MHC_SSTO:31500805-31500805 | A | upstream_gene_variant | MODIFIER | *DDX39B* | Transcript | protein_coding |
| rs2523504 | HSCHR6_MHC_SSTO:31500805-31500805 | C | upstream_gene_variant | MODIFIER | *DDX39B* | Transcript | protein_coding |
| rs2523504 | HSCHR6_MHC_SSTO:31500805-31500805 | A | upstream_gene_variant | MODIFIER | *SNORD83* | Transcript | snoRNA |
| rs2523504 | HSCHR6_MHC_SSTO:31500805-31500805 | C | upstream_gene_variant | MODIFIER | *SNORD83* | Transcript | snoRNA |
| rs2857609 | 6:31577825-31577825 | G | downstream_gene_variant | MODIFIER | *UQCRHP1* | Transcript | processed_pseudogene |
| rs2857609 | 6:31577825-31577825 | T | downstream_gene_variant | MODIFIER | *UQCRHP1* | Transcript | processed_pseudogene |
| rs2857609 | 6:31577825-31577825 | G | regulatory_region_variant | MODIFIER | *-* | RegulatoryFeature | CTCF_binding_site |
| rs2857609 | 6:31577825-31577825 | T | regulatory_region_variant | MODIFIER | *-* | RegulatoryFeature | CTCF_binding_site |
| rs2857609 | 6:31577825-31577825 | G | regulatory_region_variant | MODIFIER | *-* | RegulatoryFeature | enhancer |
| rs2857609 | 6:31577825-31577825 | T | regulatory_region_variant | MODIFIER | *-* | RegulatoryFeature | enhancer |
| rs2857609 | HSCHR6_MHC_APD:31589176-31589176 | A | downstream_gene_variant | MODIFIER | *UQCRHP1* | Transcript | processed_pseudogene |
| rs2857609 | HSCHR6_MHC_APD:31589176-31589176 | T | downstream_gene_variant | MODIFIER | *UQCRHP1* | Transcript | processed_pseudogene |
| rs2857609 | HSCHR6_MHC_COX:31565239-31565239 | A | downstream_gene_variant | MODIFIER | *UQCRHP1* | Transcript | processed_pseudogene |
| rs2857609 | HSCHR6_MHC_COX:31565239-31565239 | T | downstream_gene_variant | MODIFIER | *UQCRHP1* | Transcript | processed_pseudogene |
| rs2857609 | HSCHR6_MHC_DBB:31560007-31560007 | A | downstream_gene_variant | MODIFIER | *UQCRHP1* | Transcript | processed_pseudogene |
| rs2857609 | HSCHR6_MHC_DBB:31560007-31560007 | T | downstream_gene_variant | MODIFIER | *UQCRHP1* | Transcript | processed_pseudogene |
| rs2857609 | HSCHR6_MHC_MANN:31617313-31617313 | A | intergenic_variant | MODIFIER | *-* | - | - |
| rs2857609 | HSCHR6_MHC_MANN:31617313-31617313 | T | intergenic_variant | MODIFIER | *-* | - | - |
| rs2857609 | HSCHR6_MHC_QBL:31568070-31568070 | A | downstream_gene_variant | MODIFIER | *UQCRHP1* | Transcript | processed_pseudogene |
| rs2857609 | HSCHR6_MHC_QBL:31568070-31568070 | T | downstream_gene_variant | MODIFIER | *UQCRHP1* | Transcript | processed_pseudogene |
| rs2857609 | HSCHR6_MHC_SSTO:31567775-31567775 | A | downstream_gene_variant | MODIFIER | *UQCRHP1* | Transcript | processed_pseudogene |
| rs2857609 | HSCHR6_MHC_SSTO:31567775-31567775 | T | downstream_gene_variant | MODIFIER | *UQCRHP1* | Transcript | processed_pseudogene |
| rs2857609 | HSCHR6_MHC_SSTO:31567775-31567775 | A | intron_variant | MODIFIER | *BX511262.2* | Transcript | protein_coding |
| rs2857609 | HSCHR6_MHC_SSTO:31567775-31567775 | T | intron_variant | MODIFIER | *BX511262.2* | Transcript | protein_coding |
| rs28929474 | 14:94844947-94844947 | G | missense_variant | MODERATE | *SERPINA1* | Transcript | protein_coding |
| rs28929474 | 14:94844947-94844947 | T | missense_variant | MODERATE | *SERPINA1* | Transcript | protein_coding |
| rs28929474 | 14:94844947-94844947 | G | missense_variant | MODERATE | *SERPINA1* | Transcript | protein_coding |
| rs28929474 | 14:94844947-94844947 | T | missense_variant | MODERATE | *SERPINA1* | Transcript | protein_coding |
| rs28929474 | 14:94844947-94844947 | G | missense_variant | MODERATE | *SERPINA1* | Transcript | protein_coding |
| rs28929474 | 14:94844947-94844947 | T | missense_variant | MODERATE | *SERPINA1* | Transcript | protein_coding |
| rs28929474 | 14:94844947-94844947 | G | downstream_gene_variant | MODIFIER | *SERPINA1* | Transcript | protein_coding |
| rs28929474 | 14:94844947-94844947 | T | downstream_gene_variant | MODIFIER | *SERPINA1* | Transcript | protein_coding |
| rs28929474 | 14:94844947-94844947 | G | missense_variant | MODERATE | *SERPINA1* | Transcript | protein_coding |
| rs28929474 | 14:94844947-94844947 | T | missense_variant | MODERATE | *SERPINA1* | Transcript | protein_coding |
| rs28929474 | 14:94844947-94844947 | G | missense_variant | MODERATE | *SERPINA1* | Transcript | protein_coding |
| rs28929474 | 14:94844947-94844947 | T | missense_variant | MODERATE | *SERPINA1* | Transcript | protein_coding |
| rs28929474 | 14:94844947-94844947 | G | missense_variant | MODERATE | *SERPINA1* | Transcript | protein_coding |
| rs28929474 | 14:94844947-94844947 | T | missense_variant | MODERATE | *SERPINA1* | Transcript | protein_coding |
| rs28929474 | 14:94844947-94844947 | G | missense_variant | MODERATE | *SERPINA1* | Transcript | protein_coding |
| rs28929474 | 14:94844947-94844947 | T | missense_variant | MODERATE | *SERPINA1* | Transcript | protein_coding |
| rs28929474 | 14:94844947-94844947 | G | missense_variant | MODERATE | *SERPINA1* | Transcript | protein_coding |
| rs28929474 | 14:94844947-94844947 | T | missense_variant | MODERATE | *SERPINA1* | Transcript | protein_coding |
| rs28929474 | 14:94844947-94844947 | G | 3_prime_UTR_variant,NMD_transcript_variant | MODIFIER | *SERPINA1* | Transcript | nonsense_mediated_decay |
| rs28929474 | 14:94844947-94844947 | T | 3_prime_UTR_variant,NMD_transcript_variant | MODIFIER | *SERPINA1* | Transcript | nonsense_mediated_decay |
| rs28929474 | 14:94844947-94844947 | G | downstream_gene_variant | MODIFIER | *SERPINA1* | Transcript | protein_coding |
| rs28929474 | 14:94844947-94844947 | T | downstream_gene_variant | MODIFIER | *SERPINA1* | Transcript | protein_coding |
| rs28929474 | 14:94844947-94844947 | G | downstream_gene_variant | MODIFIER | *SERPINA1* | Transcript | protein_coding |
| rs28929474 | 14:94844947-94844947 | T | downstream_gene_variant | MODIFIER | *SERPINA1* | Transcript | protein_coding |
| rs28929474 | 14:94844947-94844947 | G | downstream_gene_variant | MODIFIER | *SERPINA1* | Transcript | protein_coding |
| rs28929474 | 14:94844947-94844947 | T | downstream_gene_variant | MODIFIER | *SERPINA1* | Transcript | protein_coding |
| rs28929474 | 14:94844947-94844947 | G | downstream_gene_variant | MODIFIER | *SERPINA1* | Transcript | protein_coding |
| rs28929474 | 14:94844947-94844947 | T | downstream_gene_variant | MODIFIER | *SERPINA1* | Transcript | protein_coding |
| rs28929474 | 14:94844947-94844947 | G | downstream_gene_variant | MODIFIER | *SERPINA1* | Transcript | protein_coding |
| rs28929474 | 14:94844947-94844947 | T | downstream_gene_variant | MODIFIER | *SERPINA1* | Transcript | protein_coding |
| rs28929474 | 14:94844947-94844947 | G | downstream_gene_variant | MODIFIER | *SERPINA1* | Transcript | protein_coding |
| rs28929474 | 14:94844947-94844947 | T | downstream_gene_variant | MODIFIER | *SERPINA1* | Transcript | protein_coding |
| rs3130279 | 6:32112626-32112626 | C | downstream_gene_variant | MODIFIER | *PRRT1* | Transcript | protein_coding |
| rs3130279 | 6:32112626-32112626 | G | downstream_gene_variant | MODIFIER | *PRRT1* | Transcript | protein_coding |
| rs3130279 | 6:32112626-32112626 | C | downstream_gene_variant | MODIFIER | *PRRT1* | Transcript | protein_coding |
| rs3130279 | 6:32112626-32112626 | G | downstream_gene_variant | MODIFIER | *PRRT1* | Transcript | protein_coding |
| rs3130279 | 6:32112626-32112626 | C | downstream_gene_variant | MODIFIER | *PRRT1* | Transcript | protein_coding |
| rs3130279 | 6:32112626-32112626 | G | downstream_gene_variant | MODIFIER | *PRRT1* | Transcript | protein_coding |
| rs3130279 | 6:32112626-32112626 | C | downstream_gene_variant | MODIFIER | *PRRT1* | Transcript | processed_transcript |
| rs3130279 | 6:32112626-32112626 | G | downstream_gene_variant | MODIFIER | *PRRT1* | Transcript | processed_transcript |
| rs3130279 | 6:32112626-32112626 | C | downstream_gene_variant | MODIFIER | *PRRT1* | Transcript | processed_transcript |
| rs3130279 | 6:32112626-32112626 | G | downstream_gene_variant | MODIFIER | *PRRT1* | Transcript | processed_transcript |
| rs3130279 | 6:32112626-32112626 | C | downstream_gene_variant | MODIFIER | *PRRT1* | Transcript | retained_intron |
| rs3130279 | 6:32112626-32112626 | G | downstream_gene_variant | MODIFIER | *PRRT1* | Transcript | retained_intron |
| rs3130279 | HSCHR6_MHC_APD:32123966-32123966 | A | downstream_gene_variant | MODIFIER | *PRRT1* | Transcript | protein_coding |
| rs3130279 | HSCHR6_MHC_APD:32123966-32123966 | C | downstream_gene_variant | MODIFIER | *PRRT1* | Transcript | protein_coding |
| rs3130279 | HSCHR6_MHC_APD:32123966-32123966 | A | downstream_gene_variant | MODIFIER | *PRRT1* | Transcript | protein_coding |
| rs3130279 | HSCHR6_MHC_APD:32123966-32123966 | C | downstream_gene_variant | MODIFIER | *PRRT1* | Transcript | protein_coding |
| rs3130279 | HSCHR6_MHC_APD:32123966-32123966 | A | downstream_gene_variant | MODIFIER | *PRRT1* | Transcript | retained_intron |
| rs3130279 | HSCHR6_MHC_APD:32123966-32123966 | C | downstream_gene_variant | MODIFIER | *PRRT1* | Transcript | retained_intron |
| rs3130279 | HSCHR6_MHC_APD:32123966-32123966 | A | downstream_gene_variant | MODIFIER | *PRRT1* | Transcript | processed_transcript |
| rs3130279 | HSCHR6_MHC_APD:32123966-32123966 | C | downstream_gene_variant | MODIFIER | *PRRT1* | Transcript | processed_transcript |
| rs3130279 | HSCHR6_MHC_APD:32123966-32123966 | A | downstream_gene_variant | MODIFIER | *PRRT1* | Transcript | processed_transcript |
| rs3130279 | HSCHR6_MHC_APD:32123966-32123966 | C | downstream_gene_variant | MODIFIER | *PRRT1* | Transcript | processed_transcript |
| rs3130279 | HSCHR6_MHC_APD:32123966-32123966 | A | downstream_gene_variant | MODIFIER | *PRRT1* | Transcript | protein_coding |
| rs3130279 | HSCHR6_MHC_APD:32123966-32123966 | C | downstream_gene_variant | MODIFIER | *PRRT1* | Transcript | protein_coding |
| rs3130279 | HSCHR6_MHC_COX:32061085-32061085 | A | downstream_gene_variant | MODIFIER | *PRRT1* | Transcript | protein_coding |
| rs3130279 | HSCHR6_MHC_COX:32061085-32061085 | C | downstream_gene_variant | MODIFIER | *PRRT1* | Transcript | protein_coding |
| rs3130279 | HSCHR6_MHC_COX:32061085-32061085 | A | downstream_gene_variant | MODIFIER | *PRRT1* | Transcript | protein_coding |
| rs3130279 | HSCHR6_MHC_COX:32061085-32061085 | C | downstream_gene_variant | MODIFIER | *PRRT1* | Transcript | protein_coding |
| rs3130279 | HSCHR6_MHC_COX:32061085-32061085 | A | downstream_gene_variant | MODIFIER | *PRRT1* | Transcript | retained_intron |
| rs3130279 | HSCHR6_MHC_COX:32061085-32061085 | C | downstream_gene_variant | MODIFIER | *PRRT1* | Transcript | retained_intron |
| rs3130279 | HSCHR6_MHC_COX:32061085-32061085 | A | downstream_gene_variant | MODIFIER | *PRRT1* | Transcript | processed_transcript |
| rs3130279 | HSCHR6_MHC_COX:32061085-32061085 | C | downstream_gene_variant | MODIFIER | *PRRT1* | Transcript | processed_transcript |
| rs3130279 | HSCHR6_MHC_COX:32061085-32061085 | A | downstream_gene_variant | MODIFIER | *PRRT1* | Transcript | processed_transcript |
| rs3130279 | HSCHR6_MHC_COX:32061085-32061085 | C | downstream_gene_variant | MODIFIER | *PRRT1* | Transcript | processed_transcript |
| rs3130279 | HSCHR6_MHC_COX:32061085-32061085 | A | downstream_gene_variant | MODIFIER | *PRRT1* | Transcript | protein_coding |
| rs3130279 | HSCHR6_MHC_COX:32061085-32061085 | C | downstream_gene_variant | MODIFIER | *PRRT1* | Transcript | protein_coding |
| rs3130279 | HSCHR6_MHC_DBB:32088397-32088397 | A | downstream_gene_variant | MODIFIER | *PRRT1* | Transcript | protein_coding |
| rs3130279 | HSCHR6_MHC_DBB:32088397-32088397 | C | downstream_gene_variant | MODIFIER | *PRRT1* | Transcript | protein_coding |
| rs3130279 | HSCHR6_MHC_DBB:32088397-32088397 | A | downstream_gene_variant | MODIFIER | *PRRT1* | Transcript | protein_coding |
| rs3130279 | HSCHR6_MHC_DBB:32088397-32088397 | C | downstream_gene_variant | MODIFIER | *PRRT1* | Transcript | protein_coding |
| rs3130279 | HSCHR6_MHC_DBB:32088397-32088397 | A | downstream_gene_variant | MODIFIER | *PRRT1* | Transcript | processed_transcript |
| rs3130279 | HSCHR6_MHC_DBB:32088397-32088397 | C | downstream_gene_variant | MODIFIER | *PRRT1* | Transcript | processed_transcript |
| rs3130279 | HSCHR6_MHC_DBB:32088397-32088397 | A | downstream_gene_variant | MODIFIER | *PRRT1* | Transcript | retained_intron |
| rs3130279 | HSCHR6_MHC_DBB:32088397-32088397 | C | downstream_gene_variant | MODIFIER | *PRRT1* | Transcript | retained_intron |
| rs3130279 | HSCHR6_MHC_DBB:32088397-32088397 | A | downstream_gene_variant | MODIFIER | *PRRT1* | Transcript | processed_transcript |
| rs3130279 | HSCHR6_MHC_DBB:32088397-32088397 | C | downstream_gene_variant | MODIFIER | *PRRT1* | Transcript | processed_transcript |
| rs3130279 | HSCHR6_MHC_DBB:32088397-32088397 | A | downstream_gene_variant | MODIFIER | *PRRT1* | Transcript | protein_coding |
| rs3130279 | HSCHR6_MHC_DBB:32088397-32088397 | C | downstream_gene_variant | MODIFIER | *PRRT1* | Transcript | protein_coding |
| rs3130279 | HSCHR6_MHC_MANN:32152072-32152072 | A | downstream_gene_variant | MODIFIER | *PRRT1* | Transcript | protein_coding |
| rs3130279 | HSCHR6_MHC_MANN:32152072-32152072 | C | downstream_gene_variant | MODIFIER | *PRRT1* | Transcript | protein_coding |
| rs3130279 | HSCHR6_MHC_MANN:32152072-32152072 | A | downstream_gene_variant | MODIFIER | *PRRT1* | Transcript | protein_coding |
| rs3130279 | HSCHR6_MHC_MANN:32152072-32152072 | C | downstream_gene_variant | MODIFIER | *PRRT1* | Transcript | protein_coding |
| rs3130279 | HSCHR6_MHC_MANN:32152072-32152072 | A | downstream_gene_variant | MODIFIER | *PRRT1* | Transcript | processed_transcript |
| rs3130279 | HSCHR6_MHC_MANN:32152072-32152072 | C | downstream_gene_variant | MODIFIER | *PRRT1* | Transcript | processed_transcript |
| rs3130279 | HSCHR6_MHC_MANN:32152072-32152072 | A | downstream_gene_variant | MODIFIER | *PRRT1* | Transcript | retained_intron |
| rs3130279 | HSCHR6_MHC_MANN:32152072-32152072 | C | downstream_gene_variant | MODIFIER | *PRRT1* | Transcript | retained_intron |
| rs3130279 | HSCHR6_MHC_MANN:32152072-32152072 | A | downstream_gene_variant | MODIFIER | *PRRT1* | Transcript | processed_transcript |
| rs3130279 | HSCHR6_MHC_MANN:32152072-32152072 | C | downstream_gene_variant | MODIFIER | *PRRT1* | Transcript | processed_transcript |
| rs3130279 | HSCHR6_MHC_MANN:32152072-32152072 | A | downstream_gene_variant | MODIFIER | *PRRT1* | Transcript | protein_coding |
| rs3130279 | HSCHR6_MHC_MANN:32152072-32152072 | C | downstream_gene_variant | MODIFIER | *PRRT1* | Transcript | protein_coding |
| rs3130279 | HSCHR6_MHC_MCF:32189052-32189052 | A | downstream_gene_variant | MODIFIER | *PRRT1* | Transcript | protein_coding |
| rs3130279 | HSCHR6_MHC_MCF:32189052-32189052 | C | downstream_gene_variant | MODIFIER | *PRRT1* | Transcript | protein_coding |
| rs3130279 | HSCHR6_MHC_MCF:32189052-32189052 | A | downstream_gene_variant | MODIFIER | *PRRT1* | Transcript | protein_coding |
| rs3130279 | HSCHR6_MHC_MCF:32189052-32189052 | C | downstream_gene_variant | MODIFIER | *PRRT1* | Transcript | protein_coding |
| rs3130279 | HSCHR6_MHC_MCF:32189052-32189052 | A | downstream_gene_variant | MODIFIER | *PRRT1* | Transcript | processed_transcript |
| rs3130279 | HSCHR6_MHC_MCF:32189052-32189052 | C | downstream_gene_variant | MODIFIER | *PRRT1* | Transcript | processed_transcript |
| rs3130279 | HSCHR6_MHC_MCF:32189052-32189052 | A | downstream_gene_variant | MODIFIER | *PRRT1* | Transcript | retained_intron |
| rs3130279 | HSCHR6_MHC_MCF:32189052-32189052 | C | downstream_gene_variant | MODIFIER | *PRRT1* | Transcript | retained_intron |
| rs3130279 | HSCHR6_MHC_MCF:32189052-32189052 | A | downstream_gene_variant | MODIFIER | *PRRT1* | Transcript | processed_transcript |
| rs3130279 | HSCHR6_MHC_MCF:32189052-32189052 | C | downstream_gene_variant | MODIFIER | *PRRT1* | Transcript | processed_transcript |
| rs3130279 | HSCHR6_MHC_MCF:32189052-32189052 | A | downstream_gene_variant | MODIFIER | *PRRT1* | Transcript | protein_coding |
| rs3130279 | HSCHR6_MHC_MCF:32189052-32189052 | C | downstream_gene_variant | MODIFIER | *PRRT1* | Transcript | protein_coding |
| rs3130279 | HSCHR6_MHC_QBL:32070244-32070244 | A | downstream_gene_variant | MODIFIER | *PRRT1* | Transcript | protein_coding |
| rs3130279 | HSCHR6_MHC_QBL:32070244-32070244 | C | downstream_gene_variant | MODIFIER | *PRRT1* | Transcript | protein_coding |
| rs3130279 | HSCHR6_MHC_QBL:32070244-32070244 | A | downstream_gene_variant | MODIFIER | *PRRT1* | Transcript | protein_coding |
| rs3130279 | HSCHR6_MHC_QBL:32070244-32070244 | C | downstream_gene_variant | MODIFIER | *PRRT1* | Transcript | protein_coding |
| rs3130279 | HSCHR6_MHC_QBL:32070244-32070244 | A | downstream_gene_variant | MODIFIER | *PRRT1* | Transcript | retained_intron |
| rs3130279 | HSCHR6_MHC_QBL:32070244-32070244 | C | downstream_gene_variant | MODIFIER | *PRRT1* | Transcript | retained_intron |
| rs3130279 | HSCHR6_MHC_QBL:32070244-32070244 | A | downstream_gene_variant | MODIFIER | *PRRT1* | Transcript | processed_transcript |
| rs3130279 | HSCHR6_MHC_QBL:32070244-32070244 | C | downstream_gene_variant | MODIFIER | *PRRT1* | Transcript | processed_transcript |
| rs3130279 | HSCHR6_MHC_QBL:32070244-32070244 | A | downstream_gene_variant | MODIFIER | *PRRT1* | Transcript | processed_transcript |
| rs3130279 | HSCHR6_MHC_QBL:32070244-32070244 | C | downstream_gene_variant | MODIFIER | *PRRT1* | Transcript | processed_transcript |
| rs3130279 | HSCHR6_MHC_QBL:32070244-32070244 | A | downstream_gene_variant | MODIFIER | *PRRT1* | Transcript | protein_coding |
| rs3130279 | HSCHR6_MHC_QBL:32070244-32070244 | C | downstream_gene_variant | MODIFIER | *PRRT1* | Transcript | protein_coding |
| rs3130279 | HSCHR6_MHC_SSTO:32119493-32119493 | A | downstream_gene_variant | MODIFIER | *PRRT1* | Transcript | protein_coding |
| rs3130279 | HSCHR6_MHC_SSTO:32119493-32119493 | C | downstream_gene_variant | MODIFIER | *PRRT1* | Transcript | protein_coding |
| rs3130279 | HSCHR6_MHC_SSTO:32119493-32119493 | A | downstream_gene_variant | MODIFIER | *PRRT1* | Transcript | protein_coding |
| rs3130279 | HSCHR6_MHC_SSTO:32119493-32119493 | C | downstream_gene_variant | MODIFIER | *PRRT1* | Transcript | protein_coding |
| rs3130279 | HSCHR6_MHC_SSTO:32119493-32119493 | A | downstream_gene_variant | MODIFIER | *PRRT1* | Transcript | processed_transcript |
| rs3130279 | HSCHR6_MHC_SSTO:32119493-32119493 | C | downstream_gene_variant | MODIFIER | *PRRT1* | Transcript | processed_transcript |
| rs3130279 | HSCHR6_MHC_SSTO:32119493-32119493 | A | downstream_gene_variant | MODIFIER | *PRRT1* | Transcript | processed_transcript |
| rs3130279 | HSCHR6_MHC_SSTO:32119493-32119493 | C | downstream_gene_variant | MODIFIER | *PRRT1* | Transcript | processed_transcript |
| rs3130279 | HSCHR6_MHC_SSTO:32119493-32119493 | A | downstream_gene_variant | MODIFIER | *PRRT1* | Transcript | retained_intron |
| rs3130279 | HSCHR6_MHC_SSTO:32119493-32119493 | C | downstream_gene_variant | MODIFIER | *PRRT1* | Transcript | retained_intron |
| rs3130279 | HSCHR6_MHC_SSTO:32119493-32119493 | A | downstream_gene_variant | MODIFIER | *PRRT1* | Transcript | protein_coding |
| rs3130279 | HSCHR6_MHC_SSTO:32119493-32119493 | C | downstream_gene_variant | MODIFIER | *PRRT1* | Transcript | protein_coding |
| rs35134156 | 15:77315432-77315432 | G | intron_variant | MODIFIER | *PSTPIP1* | Transcript | protein_coding |
| rs35134156 | 15:77315432-77315432 | T | intron_variant | MODIFIER | *PSTPIP1* | Transcript | protein_coding |
| rs35134156 | 15:77315432-77315432 | G | intron_variant | MODIFIER | *PSTPIP1* | Transcript | protein_coding |
| rs35134156 | 15:77315432-77315432 | T | intron_variant | MODIFIER | *PSTPIP1* | Transcript | protein_coding |
| rs35134156 | 15:77315432-77315432 | G | intron_variant | MODIFIER | *PSTPIP1* | Transcript | protein_coding |
| rs35134156 | 15:77315432-77315432 | T | intron_variant | MODIFIER | *PSTPIP1* | Transcript | protein_coding |
| rs35134156 | 15:77315432-77315432 | G | intron_variant | MODIFIER | *PSTPIP1* | Transcript | protein_coding |
| rs35134156 | 15:77315432-77315432 | T | intron_variant | MODIFIER | *PSTPIP1* | Transcript | protein_coding |
| rs35134156 | 15:77315432-77315432 | G | intron_variant | MODIFIER | *PSTPIP1* | Transcript | protein_coding |
| rs35134156 | 15:77315432-77315432 | T | intron_variant | MODIFIER | *PSTPIP1* | Transcript | protein_coding |
| rs35134156 | 15:77315432-77315432 | G | intron_variant | MODIFIER | *PSTPIP1* | Transcript | protein_coding |
| rs35134156 | 15:77315432-77315432 | T | intron_variant | MODIFIER | *PSTPIP1* | Transcript | protein_coding |
| rs35134156 | 15:77315432-77315432 | G | intron_variant,NMD_transcript_variant | MODIFIER | *PSTPIP1* | Transcript | nonsense_mediated_decay |
| rs35134156 | 15:77315432-77315432 | T | intron_variant,NMD_transcript_variant | MODIFIER | *PSTPIP1* | Transcript | nonsense_mediated_decay |
| rs35134156 | 15:77315432-77315432 | G | intron_variant,NMD_transcript_variant | MODIFIER | *PSTPIP1* | Transcript | nonsense_mediated_decay |
| rs35134156 | 15:77315432-77315432 | T | intron_variant,NMD_transcript_variant | MODIFIER | *PSTPIP1* | Transcript | nonsense_mediated_decay |
| rs35134156 | 15:77315432-77315432 | G | intron_variant,non_coding_transcript_variant | MODIFIER | *PSTPIP1* | Transcript | retained_intron |
| rs35134156 | 15:77315432-77315432 | T | intron_variant,non_coding_transcript_variant | MODIFIER | *PSTPIP1* | Transcript | retained_intron |
| rs35134156 | 15:77315432-77315432 | G | intron_variant | MODIFIER | *PSTPIP1* | Transcript | protein_coding |
| rs35134156 | 15:77315432-77315432 | T | intron_variant | MODIFIER | *PSTPIP1* | Transcript | protein_coding |
| rs35134156 | 15:77315432-77315432 | G | intron_variant | MODIFIER | *PSTPIP1* | Transcript | protein_coding |
| rs35134156 | 15:77315432-77315432 | T | intron_variant | MODIFIER | *PSTPIP1* | Transcript | protein_coding |
| rs35134156 | 15:77315432-77315432 | G | intron_variant,NMD_transcript_variant | MODIFIER | *PSTPIP1* | Transcript | nonsense_mediated_decay |
| rs35134156 | 15:77315432-77315432 | T | intron_variant,NMD_transcript_variant | MODIFIER | *PSTPIP1* | Transcript | nonsense_mediated_decay |
| rs35134156 | 15:77315432-77315432 | G | intron_variant,non_coding_transcript_variant | MODIFIER | *PSTPIP1* | Transcript | retained_intron |
| rs35134156 | 15:77315432-77315432 | T | intron_variant,non_coding_transcript_variant | MODIFIER | *PSTPIP1* | Transcript | retained_intron |
| rs35134156 | 15:77315432-77315432 | G | intron_variant,non_coding_transcript_variant | MODIFIER | *PSTPIP1* | Transcript | processed_transcript |
| rs35134156 | 15:77315432-77315432 | T | intron_variant,non_coding_transcript_variant | MODIFIER | *PSTPIP1* | Transcript | processed_transcript |
| rs35134156 | 15:77315432-77315432 | G | intron_variant,NMD_transcript_variant | MODIFIER | *PSTPIP1* | Transcript | nonsense_mediated_decay |
| rs35134156 | 15:77315432-77315432 | T | intron_variant,NMD_transcript_variant | MODIFIER | *PSTPIP1* | Transcript | nonsense_mediated_decay |
| rs35134156 | 15:77315432-77315432 | G | upstream_gene_variant | MODIFIER | *PSTPIP1* | Transcript | retained_intron |
| rs35134156 | 15:77315432-77315432 | T | upstream_gene_variant | MODIFIER | *PSTPIP1* | Transcript | retained_intron |
| rs362307 | 4:3241845-3241845 | T | 3_prime_UTR_variant | MODIFIER | *HTT* | Transcript | protein_coding |
| rs362307 | 4:3241845-3241845 | T | upstream_gene_variant | MODIFIER | *MSANTD1* | Transcript | protein_coding |
| rs362307 | 4:3241845-3241845 | T | downstream_gene_variant | MODIFIER | *HTT* | Transcript | retained_intron |
| rs362307 | 4:3241845-3241845 | T | non_coding_transcript_exon_variant | MODIFIER | *HTT* | Transcript | retained_intron |
| rs362307 | 4:3241845-3241845 | T | downstream_gene_variant | MODIFIER | *HTT* | Transcript | processed_transcript |
| rs362307 | 4:3241845-3241845 | T | regulatory_region_variant | MODIFIER | *-* | RegulatoryFeature | promoter_flanking_region |
| rs429358 | 19:45411941-45411941 | C | missense_variant | MODERATE | *APOE* | Transcript | protein_coding |
| rs429358 | 19:45411941-45411941 | C | downstream_gene_variant | MODIFIER | *TOMM40* | Transcript | protein_coding |
| rs429358 | 19:45411941-45411941 | C | missense_variant | MODERATE | *APOE* | Transcript | protein_coding |
| rs429358 | 19:45411941-45411941 | C | missense_variant | MODERATE | *APOE* | Transcript | protein_coding |
| rs429358 | 19:45411941-45411941 | C | missense_variant | MODERATE | *APOE* | Transcript | protein_coding |
| rs429358 | 19:45411941-45411941 | C | downstream_gene_variant | MODIFIER | *APOE* | Transcript | retained_intron |
| rs429358 | 19:45411941-45411941 | C | downstream_gene_variant | MODIFIER | *TOMM40* | Transcript | protein_coding |
| rs519790 | 11:72504141-72504141 | G | 5_prime_UTR_variant | MODIFIER | *STARD10* | Transcript | protein_coding |
| rs519790 | 11:72504141-72504141 | T | 5_prime_UTR_variant | MODIFIER | *STARD10* | Transcript | protein_coding |
| rs519790 | 11:72504141-72504141 | G | 5_prime_UTR_variant | MODIFIER | *ARAP1* | Transcript | protein_coding |
| rs519790 | 11:72504141-72504141 | T | 5_prime_UTR_variant | MODIFIER | *ARAP1* | Transcript | protein_coding |
| rs519790 | 11:72504141-72504141 | G | intron_variant | MODIFIER | *STARD10* | Transcript | protein_coding |
| rs519790 | 11:72504141-72504141 | T | intron_variant | MODIFIER | *STARD10* | Transcript | protein_coding |
| rs519790 | 11:72504141-72504141 | G | intron_variant,non_coding_transcript_variant | MODIFIER | *STARD10* | Transcript | retained_intron |
| rs519790 | 11:72504141-72504141 | T | intron_variant,non_coding_transcript_variant | MODIFIER | *STARD10* | Transcript | retained_intron |
| rs519790 | 11:72504141-72504141 | G | 5_prime_UTR_variant | MODIFIER | *STARD10* | Transcript | protein_coding |
| rs519790 | 11:72504141-72504141 | T | 5_prime_UTR_variant | MODIFIER | *STARD10* | Transcript | protein_coding |
| rs519790 | 11:72504141-72504141 | G | intron_variant | MODIFIER | *STARD10* | Transcript | protein_coding |
| rs519790 | 11:72504141-72504141 | T | intron_variant | MODIFIER | *STARD10* | Transcript | protein_coding |
| rs519790 | 11:72504141-72504141 | G | intron_variant | MODIFIER | *STARD10* | Transcript | protein_coding |
| rs519790 | 11:72504141-72504141 | T | intron_variant | MODIFIER | *STARD10* | Transcript | protein_coding |
| rs519790 | 11:72504141-72504141 | G | regulatory_region_variant | MODIFIER | *-* | RegulatoryFeature | promoter |
| rs519790 | 11:72504141-72504141 | T | regulatory_region_variant | MODIFIER | *-* | RegulatoryFeature | promoter |
| rs58304657 | 19:46176405-46176405 | C | intron_variant | MODIFIER | *GIPR* | Transcript | protein_coding |
| rs58304657 | 19:46176405-46176405 | C | intron_variant | MODIFIER | *GIPR* | Transcript | protein_coding |
| rs58304657 | 19:46176405-46176405 | C | upstream_gene_variant | MODIFIER | *MIR642A* | Transcript | miRNA |
| rs58304657 | 19:46176405-46176405 | C | intron_variant,NMD_transcript_variant | MODIFIER | *GIPR* | Transcript | nonsense_mediated_decay |
| rs58304657 | 19:46176405-46176405 | C | intron_variant,non_coding_transcript_variant | MODIFIER | *GIPR* | Transcript | retained_intron |
| rs58304657 | 19:46176405-46176405 | C | intron_variant | MODIFIER | *GIPR* | Transcript | protein_coding |
| rs58304657 | 19:46176405-46176405 | C | upstream_gene_variant | MODIFIER | *GIPR* | Transcript | retained_intron |
| rs58304657 | 19:46176405-46176405 | C | downstream_gene_variant | MODIFIER | *GIPR* | Transcript | protein_coding |
| rs58304657 | 19:46176405-46176405 | C | upstream_gene_variant | MODIFIER | *GIPR* | Transcript | processed_transcript |
| rs62052815 | 16:69561826-69561826 | T | intergenic_variant | MODIFIER | *-* | - | - |
| rs72870502 | 2:43920357-43920357 | T | intron_variant | MODIFIER | *PLEKHH2* | Transcript | protein_coding |
| rs72870502 | 2:43920357-43920357 | T | intron_variant,non_coding_transcript_variant | MODIFIER | *PLEKHH2* | Transcript | retained_intron |
| rs72870502 | 2:43920357-43920357 | T | intron_variant,non_coding_transcript_variant | MODIFIER | *PLEKHH2* | Transcript | retained_intron |
| rs72870502 | 2:43920357-43920357 | T | intron_variant,non_coding_transcript_variant | MODIFIER | *PLEKHH2* | Transcript | retained_intron |
| rs72870502 | 2:43920357-43920357 | T | intron_variant,NMD_transcript_variant | MODIFIER | *PLEKHH2* | Transcript | nonsense_mediated_decay |
| rs738408 | 22:44324730-44324730 | T | synonymous_variant | LOW | *PNPLA3* | Transcript | protein_coding |
| rs738408 | 22:44324730-44324730 | T | 3_prime_UTR_variant,NMD_transcript_variant | MODIFIER | *PNPLA3* | Transcript | nonsense_mediated_decay |
| rs738408 | 22:44324730-44324730 | T | synonymous_variant | LOW | *PNPLA3* | Transcript | protein_coding |
| rs738408 | 22:44324730-44324730 | T | non_coding_transcript_exon_variant | MODIFIER | *PNPLA3* | Transcript | processed_transcript |
| rs738408 | 22:44324730-44324730 | T | upstream_gene_variant | MODIFIER | *PNPLA3* | Transcript | processed_transcript |
| rs7461273 | 8:11777977-11777977 | A | non_coding_transcript_exon_variant | MODIFIER | *OR7E158P* | Transcript | unprocessed_pseudogene |
| rs7461273 | 8:11777977-11777977 | G | non_coding_transcript_exon_variant | MODIFIER | *OR7E158P* | Transcript | unprocessed_pseudogene |
| rs7461273 | 8:11777977-11777977 | T | non_coding_transcript_exon_variant | MODIFIER | *OR7E158P* | Transcript | unprocessed_pseudogene |
| rs76747430 | 22:40833842-40833842 | G | intron_variant | MODIFIER | *MKL1* | Transcript | protein_coding |
| rs76747430 | 22:40833842-40833842 | G | intron_variant | MODIFIER | *MKL1* | Transcript | protein_coding |
| rs76747430 | 22:40833842-40833842 | G | intron_variant | MODIFIER | *MKL1* | Transcript | protein_coding |
| rs76747430 | 22:40833842-40833842 | G | intron_variant | MODIFIER | *MKL1* | Transcript | protein_coding |
| rs76747430 | 22:40833842-40833842 | G | intron_variant | MODIFIER | *MKL1* | Transcript | protein_coding |
| rs76747430 | 22:40833842-40833842 | G | intron_variant | MODIFIER | *MKL1* | Transcript | protein_coding |
| rs76747430 | 22:40833842-40833842 | G | downstream_gene_variant | MODIFIER | *RP5-1042K10.12* | Transcript | processed_pseudogene |
| rs879882 | 6:31139452-31139452 | C | upstream_gene_variant | MODIFIER | *POU5F1* | Transcript | protein_coding |
| rs879882 | 6:31139452-31139452 | G | upstream_gene_variant | MODIFIER | *POU5F1* | Transcript | protein_coding |
| rs879882 | 6:31139452-31139452 | C | downstream_gene_variant | MODIFIER | *PSORS1C3* | Transcript | sense_intronic |
| rs879882 | 6:31139452-31139452 | G | downstream_gene_variant | MODIFIER | *PSORS1C3* | Transcript | sense_intronic |
| rs879882 | 6:31139452-31139452 | C | intron_variant | MODIFIER | *POU5F1* | Transcript | protein_coding |
| rs879882 | 6:31139452-31139452 | G | intron_variant | MODIFIER | *POU5F1* | Transcript | protein_coding |
| rs879882 | 6:31139452-31139452 | C | upstream_gene_variant | MODIFIER | *POU5F1* | Transcript | retained_intron |
| rs879882 | 6:31139452-31139452 | G | upstream_gene_variant | MODIFIER | *POU5F1* | Transcript | retained_intron |
| rs879882 | 6:31139452-31139452 | C | upstream_gene_variant | MODIFIER | *POU5F1* | Transcript | protein_coding |
| rs879882 | 6:31139452-31139452 | G | upstream_gene_variant | MODIFIER | *POU5F1* | Transcript | protein_coding |
| rs879882 | 6:31139452-31139452 | C | upstream_gene_variant | MODIFIER | *POU5F1* | Transcript | protein_coding |
| rs879882 | 6:31139452-31139452 | G | upstream_gene_variant | MODIFIER | *POU5F1* | Transcript | protein_coding |
| rs879882 | 6:31139452-31139452 | C | upstream_gene_variant | MODIFIER | *POU5F1* | Transcript | protein_coding |
| rs879882 | 6:31139452-31139452 | G | upstream_gene_variant | MODIFIER | *POU5F1* | Transcript | protein_coding |
| rs879882 | 6:31139452-31139452 | C | downstream_gene_variant | MODIFIER | *TCF19* | Transcript | protein_coding |
| rs879882 | 6:31139452-31139452 | G | downstream_gene_variant | MODIFIER | *TCF19* | Transcript | protein_coding |
| rs879882 | 6:31139452-31139452 | C | upstream_gene_variant | MODIFIER | *POU5F1* | Transcript | protein_coding |
| rs879882 | 6:31139452-31139452 | G | upstream_gene_variant | MODIFIER | *POU5F1* | Transcript | protein_coding |
| rs879882 | HSCHR6_MHC_COX:31132018-31132018 | T | upstream_gene_variant | MODIFIER | *POU5F1* | Transcript | protein_coding |
| rs879882 | HSCHR6_MHC_COX:31132018-31132018 | G | upstream_gene_variant | MODIFIER | *POU5F1* | Transcript | protein_coding |
| rs879882 | HSCHR6_MHC_COX:31132018-31132018 | T | downstream_gene_variant | MODIFIER | *PSORS1C3* | Transcript | processed_transcript |
| rs879882 | HSCHR6_MHC_COX:31132018-31132018 | G | downstream_gene_variant | MODIFIER | *PSORS1C3* | Transcript | processed_transcript |
| rs879882 | HSCHR6_MHC_COX:31132018-31132018 | T | upstream_gene_variant | MODIFIER | *POU5F1* | Transcript | retained_intron |
| rs879882 | HSCHR6_MHC_COX:31132018-31132018 | G | upstream_gene_variant | MODIFIER | *POU5F1* | Transcript | retained_intron |
| rs879882 | HSCHR6_MHC_COX:31132018-31132018 | T | upstream_gene_variant | MODIFIER | *POU5F1* | Transcript | protein_coding |
| rs879882 | HSCHR6_MHC_COX:31132018-31132018 | G | upstream_gene_variant | MODIFIER | *POU5F1* | Transcript | protein_coding |
| rs879882 | HSCHR6_MHC_COX:31132018-31132018 | T | upstream_gene_variant | MODIFIER | *POU5F1* | Transcript | protein_coding |
| rs879882 | HSCHR6_MHC_COX:31132018-31132018 | G | upstream_gene_variant | MODIFIER | *POU5F1* | Transcript | protein_coding |
| rs879882 | HSCHR6_MHC_DBB:31133196-31133196 | T | upstream_gene_variant | MODIFIER | *POU5F1* | Transcript | protein_coding |
| rs879882 | HSCHR6_MHC_DBB:31133196-31133196 | G | upstream_gene_variant | MODIFIER | *POU5F1* | Transcript | protein_coding |
| rs879882 | HSCHR6_MHC_DBB:31133196-31133196 | T | downstream_gene_variant | MODIFIER | *PSORS1C3* | Transcript | processed_transcript |
| rs879882 | HSCHR6_MHC_DBB:31133196-31133196 | G | downstream_gene_variant | MODIFIER | *PSORS1C3* | Transcript | processed_transcript |
| rs879882 | HSCHR6_MHC_DBB:31133196-31133196 | T | upstream_gene_variant | MODIFIER | *POU5F1* | Transcript | retained_intron |
| rs879882 | HSCHR6_MHC_DBB:31133196-31133196 | G | upstream_gene_variant | MODIFIER | *POU5F1* | Transcript | retained_intron |
| rs879882 | HSCHR6_MHC_DBB:31133196-31133196 | T | upstream_gene_variant | MODIFIER | *POU5F1* | Transcript | protein_coding |
| rs879882 | HSCHR6_MHC_DBB:31133196-31133196 | G | upstream_gene_variant | MODIFIER | *POU5F1* | Transcript | protein_coding |
| rs879882 | HSCHR6_MHC_DBB:31133196-31133196 | T | upstream_gene_variant | MODIFIER | *POU5F1* | Transcript | protein_coding |
| rs879882 | HSCHR6_MHC_DBB:31133196-31133196 | G | upstream_gene_variant | MODIFIER | *POU5F1* | Transcript | protein_coding |
| rs879882 | HSCHR6_MHC_MANN:31184417-31184417 | T | downstream_gene_variant | MODIFIER | *PSORS1C3* | Transcript | processed_transcript |
| rs879882 | HSCHR6_MHC_MANN:31184417-31184417 | G | downstream_gene_variant | MODIFIER | *PSORS1C3* | Transcript | processed_transcript |
| rs879882 | HSCHR6_MHC_MANN:31184417-31184417 | T | upstream_gene_variant | MODIFIER | *POU5F1* | Transcript | protein_coding |
| rs879882 | HSCHR6_MHC_MANN:31184417-31184417 | G | upstream_gene_variant | MODIFIER | *POU5F1* | Transcript | protein_coding |
| rs879882 | HSCHR6_MHC_MANN:31184417-31184417 | T | upstream_gene_variant | MODIFIER | *POU5F1* | Transcript | retained_intron |
| rs879882 | HSCHR6_MHC_MANN:31184417-31184417 | G | upstream_gene_variant | MODIFIER | *POU5F1* | Transcript | retained_intron |
| rs879882 | HSCHR6_MHC_MANN:31184417-31184417 | T | upstream_gene_variant | MODIFIER | *POU5F1* | Transcript | protein_coding |
| rs879882 | HSCHR6_MHC_MANN:31184417-31184417 | G | upstream_gene_variant | MODIFIER | *POU5F1* | Transcript | protein_coding |
| rs879882 | HSCHR6_MHC_MANN:31184417-31184417 | T | upstream_gene_variant | MODIFIER | *POU5F1* | Transcript | protein_coding |
| rs879882 | HSCHR6_MHC_MANN:31184417-31184417 | G | upstream_gene_variant | MODIFIER | *POU5F1* | Transcript | protein_coding |
| rs879882 | HSCHR6_MHC_MANN:31184417-31184417 | T | intron_variant | MODIFIER | *CR847794.1* | Transcript | protein_coding |
| rs879882 | HSCHR6_MHC_MANN:31184417-31184417 | G | intron_variant | MODIFIER | *CR847794.1* | Transcript | protein_coding |
| rs879882 | HSCHR6_MHC_MCF:31217996-31217996 | T | downstream_gene_variant | MODIFIER | *PSORS1C3* | Transcript | processed_transcript |
| rs879882 | HSCHR6_MHC_MCF:31217996-31217996 | G | downstream_gene_variant | MODIFIER | *PSORS1C3* | Transcript | processed_transcript |
| rs879882 | HSCHR6_MHC_MCF:31217996-31217996 | T | upstream_gene_variant | MODIFIER | *POU5F1* | Transcript | protein_coding |
| rs879882 | HSCHR6_MHC_MCF:31217996-31217996 | G | upstream_gene_variant | MODIFIER | *POU5F1* | Transcript | protein_coding |
| rs879882 | HSCHR6_MHC_MCF:31217996-31217996 | T | upstream_gene_variant | MODIFIER | *POU5F1* | Transcript | retained_intron |
| rs879882 | HSCHR6_MHC_MCF:31217996-31217996 | G | upstream_gene_variant | MODIFIER | *POU5F1* | Transcript | retained_intron |
| rs879882 | HSCHR6_MHC_MCF:31217996-31217996 | T | intron_variant | MODIFIER | *CR759815.2* | Transcript | protein_coding |
| rs879882 | HSCHR6_MHC_MCF:31217996-31217996 | G | intron_variant | MODIFIER | *CR759815.2* | Transcript | protein_coding |
| rs879882 | HSCHR6_MHC_MCF:31217996-31217996 | T | upstream_gene_variant | MODIFIER | *POU5F1* | Transcript | protein_coding |
| rs879882 | HSCHR6_MHC_MCF:31217996-31217996 | G | upstream_gene_variant | MODIFIER | *POU5F1* | Transcript | protein_coding |
| rs879882 | HSCHR6_MHC_MCF:31217996-31217996 | T | upstream_gene_variant | MODIFIER | *POU5F1* | Transcript | protein_coding |
| rs879882 | HSCHR6_MHC_MCF:31217996-31217996 | G | upstream_gene_variant | MODIFIER | *POU5F1* | Transcript | protein_coding |
| rs879882 | HSCHR6_MHC_QBL:31131924-31131924 | C | upstream_gene_variant | MODIFIER | *POU5F1* | Transcript | protein_coding |
| rs879882 | HSCHR6_MHC_QBL:31131924-31131924 | G | upstream_gene_variant | MODIFIER | *POU5F1* | Transcript | protein_coding |
| rs879882 | HSCHR6_MHC_QBL:31131924-31131924 | C | downstream_gene_variant | MODIFIER | *PSORS1C3* | Transcript | processed_transcript |
| rs879882 | HSCHR6_MHC_QBL:31131924-31131924 | G | downstream_gene_variant | MODIFIER | *PSORS1C3* | Transcript | processed_transcript |
| rs879882 | HSCHR6_MHC_QBL:31131924-31131924 | C | upstream_gene_variant | MODIFIER | *POU5F1* | Transcript | retained_intron |
| rs879882 | HSCHR6_MHC_QBL:31131924-31131924 | G | upstream_gene_variant | MODIFIER | *POU5F1* | Transcript | retained_intron |
| rs879882 | HSCHR6_MHC_QBL:31131924-31131924 | C | upstream_gene_variant | MODIFIER | *POU5F1* | Transcript | protein_coding |
| rs879882 | HSCHR6_MHC_QBL:31131924-31131924 | G | upstream_gene_variant | MODIFIER | *POU5F1* | Transcript | protein_coding |
| rs879882 | HSCHR6_MHC_QBL:31131924-31131924 | C | upstream_gene_variant | MODIFIER | *POU5F1* | Transcript | protein_coding |
| rs879882 | HSCHR6_MHC_QBL:31131924-31131924 | G | upstream_gene_variant | MODIFIER | *POU5F1* | Transcript | protein_coding |
| rs879882 | HSCHR6_MHC_SSTO:31133546-31133546 | T | downstream_gene_variant | MODIFIER | *PSORS1C3* | Transcript | processed_transcript |
| rs879882 | HSCHR6_MHC_SSTO:31133546-31133546 | G | downstream_gene_variant | MODIFIER | *PSORS1C3* | Transcript | processed_transcript |
| rs879882 | HSCHR6_MHC_SSTO:31133546-31133546 | T | upstream_gene_variant | MODIFIER | *POU5F1* | Transcript | protein_coding |
| rs879882 | HSCHR6_MHC_SSTO:31133546-31133546 | G | upstream_gene_variant | MODIFIER | *POU5F1* | Transcript | protein_coding |
| rs879882 | HSCHR6_MHC_SSTO:31133546-31133546 | T | upstream_gene_variant | MODIFIER | *POU5F1* | Transcript | retained_intron |
| rs879882 | HSCHR6_MHC_SSTO:31133546-31133546 | G | upstream_gene_variant | MODIFIER | *POU5F1* | Transcript | retained_intron |
| rs879882 | HSCHR6_MHC_SSTO:31133546-31133546 | T | upstream_gene_variant | MODIFIER | *POU5F1* | Transcript | protein_coding |
| rs879882 | HSCHR6_MHC_SSTO:31133546-31133546 | G | upstream_gene_variant | MODIFIER | *POU5F1* | Transcript | protein_coding |
| rs879882 | HSCHR6_MHC_SSTO:31133546-31133546 | T | upstream_gene_variant | MODIFIER | *POU5F1* | Transcript | protein_coding |
| rs879882 | HSCHR6_MHC_SSTO:31133546-31133546 | G | upstream_gene_variant | MODIFIER | *POU5F1* | Transcript | protein_coding |

CPASSOC, Cross-Phenotype Association; SNPs, single nucleotide Polymorphisms.

## Table S11. Detailed annotation of each CPASSOC-identified SNPs for type 2 diabetes mellitus (adjusted for BMI) and gallstone disease.

| **SNPs** | **Location** | **Allele** | **Consequence** | **IMPACT** | **SYMBOL** | **Feature_type** | **BIOTYPE** |
| --- | --- | --- | --- | --- | --- | --- | --- |
| rs1169288 | 12:121416650-121416650 | C | missense_variant | MODERATE | *HNF1A* | Transcript | protein_coding |
| rs1169288 | 12:121416650-121416650 | T | missense_variant | MODERATE | *HNF1A* | Transcript | protein_coding |
| rs1169288 | 12:121416650-121416650 | C | missense_variant | MODERATE | *HNF1A* | Transcript | protein_coding |
| rs1169288 | 12:121416650-121416650 | T | missense_variant | MODERATE | *HNF1A* | Transcript | protein_coding |
| rs1169288 | 12:121416650-121416650 | C | missense_variant | MODERATE | *HNF1A* | Transcript | protein_coding |
| rs1169288 | 12:121416650-121416650 | T | missense_variant | MODERATE | *HNF1A* | Transcript | protein_coding |
| rs1169288 | 12:121416650-121416650 | C | intron_variant,non_coding_transcript_variant | MODIFIER | *HNF1A-AS1* | Transcript | antisense |
| rs1169288 | 12:121416650-121416650 | T | intron_variant,non_coding_transcript_variant | MODIFIER | *HNF1A-AS1* | Transcript | antisense |
| rs1169288 | 12:121416650-121416650 | C | intron_variant,non_coding_transcript_variant | MODIFIER | *HNF1A-AS1* | Transcript | antisense |
| rs1169288 | 12:121416650-121416650 | T | intron_variant,non_coding_transcript_variant | MODIFIER | *HNF1A-AS1* | Transcript | antisense |
| rs1169288 | 12:121416650-121416650 | C | intron_variant | MODIFIER | *HNF1A* | Transcript | protein_coding |
| rs1169288 | 12:121416650-121416650 | T | intron_variant | MODIFIER | *HNF1A* | Transcript | protein_coding |
| rs1169288 | 12:121416650-121416650 | C | intron_variant,non_coding_transcript_variant | MODIFIER | *HNF1A-AS1* | Transcript | antisense |
| rs1169288 | 12:121416650-121416650 | T | intron_variant,non_coding_transcript_variant | MODIFIER | *HNF1A-AS1* | Transcript | antisense |
| rs1169288 | 12:121416650-121416650 | C | splice_region_variant,intron_variant | LOW | *HNF1A* | Transcript | protein_coding |
| rs1169288 | 12:121416650-121416650 | T | splice_region_variant,intron_variant | LOW | *HNF1A* | Transcript | protein_coding |
| rs1169288 | 12:121416650-121416650 | C | missense_variant,NMD_transcript_variant | MODERATE | *HNF1A* | Transcript | nonsense_mediated_decay |
| rs1169288 | 12:121416650-121416650 | T | missense_variant,NMD_transcript_variant | MODERATE | *HNF1A* | Transcript | nonsense_mediated_decay |
| rs1169288 | 12:121416650-121416650 | C | missense_variant,NMD_transcript_variant | MODERATE | *HNF1A* | Transcript | nonsense_mediated_decay |
| rs1169288 | 12:121416650-121416650 | T | missense_variant,NMD_transcript_variant | MODERATE | *HNF1A* | Transcript | nonsense_mediated_decay |
| rs1169288 | 12:121416650-121416650 | C | missense_variant | MODERATE | *HNF1A* | Transcript | protein_coding |
| rs1169288 | 12:121416650-121416650 | T | missense_variant | MODERATE | *HNF1A* | Transcript | protein_coding |
| rs1169288 | 12:121416650-121416650 | C | missense_variant,NMD_transcript_variant | MODERATE | *HNF1A* | Transcript | nonsense_mediated_decay |
| rs1169288 | 12:121416650-121416650 | T | missense_variant,NMD_transcript_variant | MODERATE | *HNF1A* | Transcript | nonsense_mediated_decay |
| rs1169288 | 12:121416650-121416650 | C | intron_variant | MODIFIER | *HNF1A* | Transcript | protein_coding |
| rs1169288 | 12:121416650-121416650 | T | intron_variant | MODIFIER | *HNF1A* | Transcript | protein_coding |
| rs1169288 | 12:121416650-121416650 | C | missense_variant | MODERATE | *HNF1A* | Transcript | protein_coding |
| rs1169288 | 12:121416650-121416650 | T | missense_variant | MODERATE | *HNF1A* | Transcript | protein_coding |
| rs1169288 | 12:121416650-121416650 | C | splice_region_variant,intron_variant,NMD_transcript_variant | LOW | *HNF1A* | Transcript | nonsense_mediated_decay |
| rs1169288 | 12:121416650-121416650 | T | splice_region_variant,intron_variant,NMD_transcript_variant | LOW | *HNF1A* | Transcript | nonsense_mediated_decay |
| rs1169288 | 12:121416650-121416650 | C | missense_variant,NMD_transcript_variant | MODERATE | *HNF1A* | Transcript | nonsense_mediated_decay |
| rs1169288 | 12:121416650-121416650 | T | missense_variant,NMD_transcript_variant | MODERATE | *HNF1A* | Transcript | nonsense_mediated_decay |
| rs1169288 | 12:121416650-121416650 | C | regulatory_region_variant | MODIFIER | *-* | RegulatoryFeature | promoter |
| rs1169288 | 12:121416650-121416650 | T | regulatory_region_variant | MODIFIER | *-* | RegulatoryFeature | promoter |
| rs1169307 | 12:121438382-121438382 | A | intron_variant | MODIFIER | *HNF1A* | Transcript | protein_coding |
| rs1169307 | 12:121438382-121438382 | C | intron_variant | MODIFIER | *HNF1A* | Transcript | protein_coding |
| rs1169307 | 12:121438382-121438382 | G | intron_variant | MODIFIER | *HNF1A* | Transcript | protein_coding |
| rs1169307 | 12:121438382-121438382 | A | downstream_gene_variant | MODIFIER | *C12orf43* | Transcript | protein_coding |
| rs1169307 | 12:121438382-121438382 | C | downstream_gene_variant | MODIFIER | *C12orf43* | Transcript | protein_coding |
| rs1169307 | 12:121438382-121438382 | G | downstream_gene_variant | MODIFIER | *C12orf43* | Transcript | protein_coding |
| rs1169307 | 12:121438382-121438382 | A | downstream_gene_variant | MODIFIER | *C12orf43* | Transcript | protein_coding |
| rs1169307 | 12:121438382-121438382 | C | downstream_gene_variant | MODIFIER | *C12orf43* | Transcript | protein_coding |
| rs1169307 | 12:121438382-121438382 | G | downstream_gene_variant | MODIFIER | *C12orf43* | Transcript | protein_coding |
| rs1169307 | 12:121438382-121438382 | A | downstream_gene_variant | MODIFIER | *HNF1A* | Transcript | protein_coding |
| rs1169307 | 12:121438382-121438382 | C | downstream_gene_variant | MODIFIER | *HNF1A* | Transcript | protein_coding |
| rs1169307 | 12:121438382-121438382 | G | downstream_gene_variant | MODIFIER | *HNF1A* | Transcript | protein_coding |
| rs1169307 | 12:121438382-121438382 | A | downstream_gene_variant | MODIFIER | *HNF1A* | Transcript | protein_coding |
| rs1169307 | 12:121438382-121438382 | C | downstream_gene_variant | MODIFIER | *HNF1A* | Transcript | protein_coding |
| rs1169307 | 12:121438382-121438382 | G | downstream_gene_variant | MODIFIER | *HNF1A* | Transcript | protein_coding |
| rs1169307 | 12:121438382-121438382 | A | downstream_gene_variant | MODIFIER | *C12orf43* | Transcript | protein_coding |
| rs1169307 | 12:121438382-121438382 | C | downstream_gene_variant | MODIFIER | *C12orf43* | Transcript | protein_coding |
| rs1169307 | 12:121438382-121438382 | G | downstream_gene_variant | MODIFIER | *C12orf43* | Transcript | protein_coding |
| rs1169307 | 12:121438382-121438382 | A | downstream_gene_variant | MODIFIER | *C12orf43* | Transcript | retained_intron |
| rs1169307 | 12:121438382-121438382 | C | downstream_gene_variant | MODIFIER | *C12orf43* | Transcript | retained_intron |
| rs1169307 | 12:121438382-121438382 | G | downstream_gene_variant | MODIFIER | *C12orf43* | Transcript | retained_intron |
| rs1169307 | 12:121438382-121438382 | A | downstream_gene_variant | MODIFIER | *C12orf43* | Transcript | protein_coding |
| rs1169307 | 12:121438382-121438382 | C | downstream_gene_variant | MODIFIER | *C12orf43* | Transcript | protein_coding |
| rs1169307 | 12:121438382-121438382 | G | downstream_gene_variant | MODIFIER | *C12orf43* | Transcript | protein_coding |
| rs1169307 | 12:121438382-121438382 | A | downstream_gene_variant | MODIFIER | *HNF1A* | Transcript | protein_coding |
| rs1169307 | 12:121438382-121438382 | C | downstream_gene_variant | MODIFIER | *HNF1A* | Transcript | protein_coding |
| rs1169307 | 12:121438382-121438382 | G | downstream_gene_variant | MODIFIER | *HNF1A* | Transcript | protein_coding |
| rs1169307 | 12:121438382-121438382 | A | downstream_gene_variant | MODIFIER | *C12orf43* | Transcript | protein_coding |
| rs1169307 | 12:121438382-121438382 | C | downstream_gene_variant | MODIFIER | *C12orf43* | Transcript | protein_coding |
| rs1169307 | 12:121438382-121438382 | G | downstream_gene_variant | MODIFIER | *C12orf43* | Transcript | protein_coding |
| rs1169307 | 12:121438382-121438382 | A | downstream_gene_variant | MODIFIER | *C12orf43* | Transcript | protein_coding |
| rs1169307 | 12:121438382-121438382 | C | downstream_gene_variant | MODIFIER | *C12orf43* | Transcript | protein_coding |
| rs1169307 | 12:121438382-121438382 | G | downstream_gene_variant | MODIFIER | *C12orf43* | Transcript | protein_coding |
| rs1169307 | 12:121438382-121438382 | A | downstream_gene_variant | MODIFIER | *C12orf43* | Transcript | protein_coding |
| rs1169307 | 12:121438382-121438382 | C | downstream_gene_variant | MODIFIER | *C12orf43* | Transcript | protein_coding |
| rs1169307 | 12:121438382-121438382 | G | downstream_gene_variant | MODIFIER | *C12orf43* | Transcript | protein_coding |
| rs1169307 | 12:121438382-121438382 | A | downstream_gene_variant | MODIFIER | *HNF1A* | Transcript | protein_coding |
| rs1169307 | 12:121438382-121438382 | C | downstream_gene_variant | MODIFIER | *HNF1A* | Transcript | protein_coding |
| rs1169307 | 12:121438382-121438382 | G | downstream_gene_variant | MODIFIER | *HNF1A* | Transcript | protein_coding |
| rs1169307 | 12:121438382-121438382 | A | downstream_gene_variant | MODIFIER | *HNF1A* | Transcript | nonsense_mediated_decay |
| rs1169307 | 12:121438382-121438382 | C | downstream_gene_variant | MODIFIER | *HNF1A* | Transcript | nonsense_mediated_decay |
| rs1169307 | 12:121438382-121438382 | G | downstream_gene_variant | MODIFIER | *HNF1A* | Transcript | nonsense_mediated_decay |
| rs1169307 | 12:121438382-121438382 | A | downstream_gene_variant | MODIFIER | *C12orf43* | Transcript | nonsense_mediated_decay |
| rs1169307 | 12:121438382-121438382 | C | downstream_gene_variant | MODIFIER | *C12orf43* | Transcript | nonsense_mediated_decay |
| rs1169307 | 12:121438382-121438382 | G | downstream_gene_variant | MODIFIER | *C12orf43* | Transcript | nonsense_mediated_decay |
| rs1169307 | 12:121438382-121438382 | A | downstream_gene_variant | MODIFIER | *C12orf43* | Transcript | protein_coding |
| rs1169307 | 12:121438382-121438382 | C | downstream_gene_variant | MODIFIER | *C12orf43* | Transcript | protein_coding |
| rs1169307 | 12:121438382-121438382 | G | downstream_gene_variant | MODIFIER | *C12orf43* | Transcript | protein_coding |
| rs1169307 | 12:121438382-121438382 | A | intron_variant,NMD_transcript_variant | MODIFIER | *HNF1A* | Transcript | nonsense_mediated_decay |
| rs1169307 | 12:121438382-121438382 | C | intron_variant,NMD_transcript_variant | MODIFIER | *HNF1A* | Transcript | nonsense_mediated_decay |
| rs1169307 | 12:121438382-121438382 | G | intron_variant,NMD_transcript_variant | MODIFIER | *HNF1A* | Transcript | nonsense_mediated_decay |
| rs1169307 | 12:121438382-121438382 | A | intron_variant | MODIFIER | *HNF1A* | Transcript | protein_coding |
| rs1169307 | 12:121438382-121438382 | C | intron_variant | MODIFIER | *HNF1A* | Transcript | protein_coding |
| rs1169307 | 12:121438382-121438382 | G | intron_variant | MODIFIER | *HNF1A* | Transcript | protein_coding |
| rs1169307 | 12:121438382-121438382 | A | downstream_gene_variant | MODIFIER | *HNF1A* | Transcript | nonsense_mediated_decay |
| rs1169307 | 12:121438382-121438382 | C | downstream_gene_variant | MODIFIER | *HNF1A* | Transcript | nonsense_mediated_decay |
| rs1169307 | 12:121438382-121438382 | G | downstream_gene_variant | MODIFIER | *HNF1A* | Transcript | nonsense_mediated_decay |
| rs1169307 | 12:121438382-121438382 | A | downstream_gene_variant | MODIFIER | *HNF1A* | Transcript | retained_intron |
| rs1169307 | 12:121438382-121438382 | C | downstream_gene_variant | MODIFIER | *HNF1A* | Transcript | retained_intron |
| rs1169307 | 12:121438382-121438382 | G | downstream_gene_variant | MODIFIER | *HNF1A* | Transcript | retained_intron |
| rs1169307 | 12:121438382-121438382 | A | downstream_gene_variant | MODIFIER | *HNF1A* | Transcript | protein_coding |
| rs1169307 | 12:121438382-121438382 | C | downstream_gene_variant | MODIFIER | *HNF1A* | Transcript | protein_coding |
| rs1169307 | 12:121438382-121438382 | G | downstream_gene_variant | MODIFIER | *HNF1A* | Transcript | protein_coding |
| rs1169307 | 12:121438382-121438382 | A | intron_variant | MODIFIER | *HNF1A* | Transcript | protein_coding |
| rs1169307 | 12:121438382-121438382 | C | intron_variant | MODIFIER | *HNF1A* | Transcript | protein_coding |
| rs1169307 | 12:121438382-121438382 | G | intron_variant | MODIFIER | *HNF1A* | Transcript | protein_coding |
| rs1169307 | 12:121438382-121438382 | A | downstream_gene_variant | MODIFIER | *HNF1A* | Transcript | nonsense_mediated_decay |
| rs1169307 | 12:121438382-121438382 | C | downstream_gene_variant | MODIFIER | *HNF1A* | Transcript | nonsense_mediated_decay |
| rs1169307 | 12:121438382-121438382 | G | downstream_gene_variant | MODIFIER | *HNF1A* | Transcript | nonsense_mediated_decay |
| rs1169307 | 12:121438382-121438382 | A | downstream_gene_variant | MODIFIER | *C12orf43* | Transcript | protein_coding |
| rs1169307 | 12:121438382-121438382 | C | downstream_gene_variant | MODIFIER | *C12orf43* | Transcript | protein_coding |
| rs1169307 | 12:121438382-121438382 | G | downstream_gene_variant | MODIFIER | *C12orf43* | Transcript | protein_coding |
| rs1169307 | 12:121438382-121438382 | A | intron_variant,NMD_transcript_variant | MODIFIER | *HNF1A* | Transcript | nonsense_mediated_decay |
| rs1169307 | 12:121438382-121438382 | C | intron_variant,NMD_transcript_variant | MODIFIER | *HNF1A* | Transcript | nonsense_mediated_decay |
| rs1169307 | 12:121438382-121438382 | G | intron_variant,NMD_transcript_variant | MODIFIER | *HNF1A* | Transcript | nonsense_mediated_decay |
| rs1169307 | 12:121438382-121438382 | A | non_coding_transcript_exon_variant | MODIFIER | *RP11-216P16.2* | Transcript | antisense |
| rs1169307 | 12:121438382-121438382 | C | non_coding_transcript_exon_variant | MODIFIER | *RP11-216P16.2* | Transcript | antisense |
| rs1169307 | 12:121438382-121438382 | G | non_coding_transcript_exon_variant | MODIFIER | *RP11-216P16.2* | Transcript | antisense |
| rs1260326 | 2:27730940-27730940 | C | missense_variant,splice_region_variant | MODERATE | *GCKR* | Transcript | protein_coding |
| rs1260326 | 2:27730940-27730940 | G | missense_variant,splice_region_variant | MODERATE | *GCKR* | Transcript | protein_coding |
| rs1260326 | 2:27730940-27730940 | C | missense_variant,splice_region_variant | MODERATE | *GCKR* | Transcript | protein_coding |
| rs1260326 | 2:27730940-27730940 | G | missense_variant,splice_region_variant | MODERATE | *GCKR* | Transcript | protein_coding |
| rs1260326 | 2:27730940-27730940 | C | missense_variant,splice_region_variant | MODERATE | *GCKR* | Transcript | protein_coding |
| rs1260326 | 2:27730940-27730940 | G | missense_variant,splice_region_variant | MODERATE | *GCKR* | Transcript | protein_coding |
| rs1260326 | 2:27730940-27730940 | C | downstream_gene_variant | MODIFIER | *GCKR* | Transcript | retained_intron |
| rs1260326 | 2:27730940-27730940 | G | downstream_gene_variant | MODIFIER | *GCKR* | Transcript | retained_intron |
| rs1260326 | 2:27730940-27730940 | C | splice_region_variant,non_coding_transcript_exon_variant | LOW | *GCKR* | Transcript | retained_intron |
| rs1260326 | 2:27730940-27730940 | G | splice_region_variant,non_coding_transcript_exon_variant | LOW | *GCKR* | Transcript | retained_intron |
| rs149797 | 5:72227620-72227620 | T | intergenic_variant | MODIFIER | *-* | - | - |
| rs1800961 | 20:43042364-43042364 | T | missense_variant | MODERATE | *HNF4A* | Transcript | protein_coding |
| rs1800961 | 20:43042364-43042364 | T | missense_variant | MODERATE | *HNF4A* | Transcript | protein_coding |
| rs1800961 | 20:43042364-43042364 | T | 3_prime_UTR_variant,NMD_transcript_variant | MODIFIER | *HNF4A* | Transcript | nonsense_mediated_decay |
| rs1800961 | 20:43042364-43042364 | T | missense_variant | MODERATE | *HNF4A* | Transcript | protein_coding |
| rs1800961 | 20:43042364-43042364 | T | missense_variant | MODERATE | *HNF4A* | Transcript | protein_coding |
| rs1800961 | 20:43042364-43042364 | T | missense_variant | MODERATE | *HNF4A* | Transcript | protein_coding |
| rs1800961 | 20:43042364-43042364 | T | missense_variant | MODERATE | *HNF4A* | Transcript | protein_coding |
| rs2239525 | 6:31509372-31509372 | A | downstream_gene_variant | MODIFIER | *ATP6V1G2* | Transcript | protein_coding |
| rs2239525 | 6:31509372-31509372 | C | downstream_gene_variant | MODIFIER | *ATP6V1G2* | Transcript | protein_coding |
| rs2239525 | 6:31509372-31509372 | A | downstream_gene_variant | MODIFIER | *ATP6V1G2* | Transcript | protein_coding |
| rs2239525 | 6:31509372-31509372 | C | downstream_gene_variant | MODIFIER | *ATP6V1G2* | Transcript | protein_coding |
| rs2239525 | 6:31509372-31509372 | A | upstream_gene_variant | MODIFIER | *DDX39B* | Transcript | protein_coding |
| rs2239525 | 6:31509372-31509372 | C | upstream_gene_variant | MODIFIER | *DDX39B* | Transcript | protein_coding |
| rs2239525 | 6:31509372-31509372 | A | intron_variant,NMD_transcript_variant | MODIFIER | *ATP6V1G2-DDX39B* | Transcript | nonsense_mediated_decay |
| rs2239525 | 6:31509372-31509372 | C | intron_variant,NMD_transcript_variant | MODIFIER | *ATP6V1G2-DDX39B* | Transcript | nonsense_mediated_decay |
| rs2239525 | 6:31509372-31509372 | A | intron_variant | MODIFIER | *DDX39B* | Transcript | protein_coding |
| rs2239525 | 6:31509372-31509372 | C | intron_variant | MODIFIER | *DDX39B* | Transcript | protein_coding |
| rs2239525 | 6:31509372-31509372 | A | downstream_gene_variant | MODIFIER | *ATP6V1G2* | Transcript | protein_coding |
| rs2239525 | 6:31509372-31509372 | C | downstream_gene_variant | MODIFIER | *ATP6V1G2* | Transcript | protein_coding |
| rs2239525 | 6:31509372-31509372 | A | intron_variant | MODIFIER | *DDX39B* | Transcript | protein_coding |
| rs2239525 | 6:31509372-31509372 | C | intron_variant | MODIFIER | *DDX39B* | Transcript | protein_coding |
| rs2239525 | 6:31509372-31509372 | A | upstream_gene_variant | MODIFIER | *DDX39B-AS1* | Transcript | antisense |
| rs2239525 | 6:31509372-31509372 | C | upstream_gene_variant | MODIFIER | *DDX39B-AS1* | Transcript | antisense |
| rs2239525 | 6:31509372-31509372 | A | intron_variant | MODIFIER | *DDX39B* | Transcript | protein_coding |
| rs2239525 | 6:31509372-31509372 | C | intron_variant | MODIFIER | *DDX39B* | Transcript | protein_coding |
| rs2239525 | 6:31509372-31509372 | A | intron_variant | MODIFIER | *DDX39B* | Transcript | protein_coding |
| rs2239525 | 6:31509372-31509372 | C | intron_variant | MODIFIER | *DDX39B* | Transcript | protein_coding |
| rs2239525 | 6:31509372-31509372 | A | intron_variant | MODIFIER | *DDX39B* | Transcript | protein_coding |
| rs2239525 | 6:31509372-31509372 | C | intron_variant | MODIFIER | *DDX39B* | Transcript | protein_coding |
| rs2239525 | 6:31509372-31509372 | A | upstream_gene_variant | MODIFIER | *DDX39B* | Transcript | protein_coding |
| rs2239525 | 6:31509372-31509372 | C | upstream_gene_variant | MODIFIER | *DDX39B* | Transcript | protein_coding |
| rs2239525 | 6:31509372-31509372 | A | upstream_gene_variant | MODIFIER | *DDX39B-AS1* | Transcript | antisense |
| rs2239525 | 6:31509372-31509372 | C | upstream_gene_variant | MODIFIER | *DDX39B-AS1* | Transcript | antisense |
| rs2239525 | 6:31509372-31509372 | A | upstream_gene_variant | MODIFIER | *DDX39B* | Transcript | protein_coding |
| rs2239525 | 6:31509372-31509372 | C | upstream_gene_variant | MODIFIER | *DDX39B* | Transcript | protein_coding |
| rs2239525 | 6:31509372-31509372 | A | intron_variant | MODIFIER | *DDX39B* | Transcript | protein_coding |
| rs2239525 | 6:31509372-31509372 | C | intron_variant | MODIFIER | *DDX39B* | Transcript | protein_coding |
| rs2239525 | 6:31509372-31509372 | A | intron_variant | MODIFIER | *DDX39B* | Transcript | protein_coding |
| rs2239525 | 6:31509372-31509372 | C | intron_variant | MODIFIER | *DDX39B* | Transcript | protein_coding |
| rs2239525 | 6:31509372-31509372 | A | intron_variant | MODIFIER | *DDX39B* | Transcript | protein_coding |
| rs2239525 | 6:31509372-31509372 | C | intron_variant | MODIFIER | *DDX39B* | Transcript | protein_coding |
| rs2239525 | 6:31509372-31509372 | A | intron_variant | MODIFIER | *DDX39B* | Transcript | protein_coding |
| rs2239525 | 6:31509372-31509372 | C | intron_variant | MODIFIER | *DDX39B* | Transcript | protein_coding |
| rs2239525 | 6:31509372-31509372 | A | intron_variant | MODIFIER | *DDX39B* | Transcript | protein_coding |
| rs2239525 | 6:31509372-31509372 | C | intron_variant | MODIFIER | *DDX39B* | Transcript | protein_coding |
| rs2239525 | 6:31509372-31509372 | A | intron_variant | MODIFIER | *DDX39B* | Transcript | protein_coding |
| rs2239525 | 6:31509372-31509372 | C | intron_variant | MODIFIER | *DDX39B* | Transcript | protein_coding |
| rs2239525 | 6:31509372-31509372 | A | intron_variant | MODIFIER | *DDX39B* | Transcript | protein_coding |
| rs2239525 | 6:31509372-31509372 | C | intron_variant | MODIFIER | *DDX39B* | Transcript | protein_coding |
| rs2239525 | 6:31509372-31509372 | A | intron_variant | MODIFIER | *DDX39B* | Transcript | protein_coding |
| rs2239525 | 6:31509372-31509372 | C | intron_variant | MODIFIER | *DDX39B* | Transcript | protein_coding |
| rs2239525 | 6:31509372-31509372 | A | intron_variant | MODIFIER | *DDX39B* | Transcript | protein_coding |
| rs2239525 | 6:31509372-31509372 | C | intron_variant | MODIFIER | *DDX39B* | Transcript | protein_coding |
| rs2239525 | 6:31509372-31509372 | A | intron_variant,non_coding_transcript_variant | MODIFIER | *DDX39B* | Transcript | retained_intron |
| rs2239525 | 6:31509372-31509372 | C | intron_variant,non_coding_transcript_variant | MODIFIER | *DDX39B* | Transcript | retained_intron |
| rs2239525 | 6:31509372-31509372 | A | downstream_gene_variant | MODIFIER | *ATP6V1G2-DDX39B* | Transcript | processed_transcript |
| rs2239525 | 6:31509372-31509372 | C | downstream_gene_variant | MODIFIER | *ATP6V1G2-DDX39B* | Transcript | processed_transcript |
| rs2239525 | 6:31509372-31509372 | A | intron_variant,NMD_transcript_variant | MODIFIER | *ATP6V1G2-DDX39B* | Transcript | nonsense_mediated_decay |
| rs2239525 | 6:31509372-31509372 | C | intron_variant,NMD_transcript_variant | MODIFIER | *ATP6V1G2-DDX39B* | Transcript | nonsense_mediated_decay |
| rs2239525 | 6:31509372-31509372 | A | non_coding_transcript_exon_variant | MODIFIER | *DDX39B* | Transcript | retained_intron |
| rs2239525 | 6:31509372-31509372 | C | non_coding_transcript_exon_variant | MODIFIER | *DDX39B* | Transcript | retained_intron |
| rs2239525 | 6:31509372-31509372 | A | downstream_gene_variant | MODIFIER | *ATP6V1G2* | Transcript | retained_intron |
| rs2239525 | 6:31509372-31509372 | C | downstream_gene_variant | MODIFIER | *ATP6V1G2* | Transcript | retained_intron |
| rs2239525 | 6:31509372-31509372 | A | intron_variant,non_coding_transcript_variant | MODIFIER | *DDX39B* | Transcript | retained_intron |
| rs2239525 | 6:31509372-31509372 | C | intron_variant,non_coding_transcript_variant | MODIFIER | *DDX39B* | Transcript | retained_intron |
| rs2239525 | 6:31509372-31509372 | A | downstream_gene_variant | MODIFIER | *ATP6V1G2* | Transcript | processed_transcript |
| rs2239525 | 6:31509372-31509372 | C | downstream_gene_variant | MODIFIER | *ATP6V1G2* | Transcript | processed_transcript |
| rs2239525 | 6:31509372-31509372 | A | downstream_gene_variant | MODIFIER | *ATP6V1G2* | Transcript | protein_coding |
| rs2239525 | 6:31509372-31509372 | C | downstream_gene_variant | MODIFIER | *ATP6V1G2* | Transcript | protein_coding |
| rs2239525 | 6:31509372-31509372 | A | upstream_gene_variant | MODIFIER | *SNORD84* | Transcript | snoRNA |
| rs2239525 | 6:31509372-31509372 | C | upstream_gene_variant | MODIFIER | *SNORD84* | Transcript | snoRNA |
| rs2239525 | 6:31509372-31509372 | A | regulatory_region_variant | MODIFIER | *-* | RegulatoryFeature | promoter |
| rs2239525 | 6:31509372-31509372 | C | regulatory_region_variant | MODIFIER | *-* | RegulatoryFeature | promoter |
| rs2239525 | HSCHR6_MHC_COX:31496827-31496827 | G | intron_variant | MODIFIER | *DDX39B* | Transcript | protein_coding |
| rs2239525 | HSCHR6_MHC_COX:31496827-31496827 | C | intron_variant | MODIFIER | *DDX39B* | Transcript | protein_coding |
| rs2239525 | HSCHR6_MHC_COX:31496827-31496827 | G | intron_variant | MODIFIER | *DDX39B* | Transcript | protein_coding |
| rs2239525 | HSCHR6_MHC_COX:31496827-31496827 | C | intron_variant | MODIFIER | *DDX39B* | Transcript | protein_coding |
| rs2239525 | HSCHR6_MHC_COX:31496827-31496827 | G | intron_variant | MODIFIER | *DDX39B* | Transcript | protein_coding |
| rs2239525 | HSCHR6_MHC_COX:31496827-31496827 | C | intron_variant | MODIFIER | *DDX39B* | Transcript | protein_coding |
| rs2239525 | HSCHR6_MHC_COX:31496827-31496827 | G | intron_variant | MODIFIER | *DDX39B* | Transcript | protein_coding |
| rs2239525 | HSCHR6_MHC_COX:31496827-31496827 | C | intron_variant | MODIFIER | *DDX39B* | Transcript | protein_coding |
| rs2239525 | HSCHR6_MHC_COX:31496827-31496827 | G | upstream_gene_variant | MODIFIER | *DDX39B* | Transcript | protein_coding |
| rs2239525 | HSCHR6_MHC_COX:31496827-31496827 | C | upstream_gene_variant | MODIFIER | *DDX39B* | Transcript | protein_coding |
| rs2239525 | HSCHR6_MHC_COX:31496827-31496827 | G | intron_variant | MODIFIER | *DDX39B* | Transcript | protein_coding |
| rs2239525 | HSCHR6_MHC_COX:31496827-31496827 | C | intron_variant | MODIFIER | *DDX39B* | Transcript | protein_coding |
| rs2239525 | HSCHR6_MHC_COX:31496827-31496827 | G | upstream_gene_variant | MODIFIER | *DDX39B-AS1* | Transcript | processed_transcript |
| rs2239525 | HSCHR6_MHC_COX:31496827-31496827 | C | upstream_gene_variant | MODIFIER | *DDX39B-AS1* | Transcript | processed_transcript |
| rs2239525 | HSCHR6_MHC_COX:31496827-31496827 | G | intron_variant | MODIFIER | *DDX39B* | Transcript | protein_coding |
| rs2239525 | HSCHR6_MHC_COX:31496827-31496827 | C | intron_variant | MODIFIER | *DDX39B* | Transcript | protein_coding |
| rs2239525 | HSCHR6_MHC_COX:31496827-31496827 | G | intron_variant | MODIFIER | *DDX39B* | Transcript | protein_coding |
| rs2239525 | HSCHR6_MHC_COX:31496827-31496827 | C | intron_variant | MODIFIER | *DDX39B* | Transcript | protein_coding |
| rs2239525 | HSCHR6_MHC_COX:31496827-31496827 | G | upstream_gene_variant | MODIFIER | *DDX39B-AS1* | Transcript | processed_transcript |
| rs2239525 | HSCHR6_MHC_COX:31496827-31496827 | C | upstream_gene_variant | MODIFIER | *DDX39B-AS1* | Transcript | processed_transcript |
| rs2239525 | HSCHR6_MHC_COX:31496827-31496827 | G | upstream_gene_variant | MODIFIER | *DDX39B* | Transcript | protein_coding |
| rs2239525 | HSCHR6_MHC_COX:31496827-31496827 | C | upstream_gene_variant | MODIFIER | *DDX39B* | Transcript | protein_coding |
| rs2239525 | HSCHR6_MHC_COX:31496827-31496827 | G | upstream_gene_variant | MODIFIER | *DDX39B* | Transcript | protein_coding |
| rs2239525 | HSCHR6_MHC_COX:31496827-31496827 | C | upstream_gene_variant | MODIFIER | *DDX39B* | Transcript | protein_coding |
| rs2239525 | HSCHR6_MHC_COX:31496827-31496827 | G | intron_variant | MODIFIER | *DDX39B* | Transcript | protein_coding |
| rs2239525 | HSCHR6_MHC_COX:31496827-31496827 | C | intron_variant | MODIFIER | *DDX39B* | Transcript | protein_coding |
| rs2239525 | HSCHR6_MHC_COX:31496827-31496827 | G | downstream_gene_variant | MODIFIER | *ATP6V1G2* | Transcript | protein_coding |
| rs2239525 | HSCHR6_MHC_COX:31496827-31496827 | C | downstream_gene_variant | MODIFIER | *ATP6V1G2* | Transcript | protein_coding |
| rs2239525 | HSCHR6_MHC_COX:31496827-31496827 | G | intron_variant | MODIFIER | *DDX39B* | Transcript | protein_coding |
| rs2239525 | HSCHR6_MHC_COX:31496827-31496827 | C | intron_variant | MODIFIER | *DDX39B* | Transcript | protein_coding |
| rs2239525 | HSCHR6_MHC_COX:31496827-31496827 | G | downstream_gene_variant | MODIFIER | *ATP6V1G2* | Transcript | protein_coding |
| rs2239525 | HSCHR6_MHC_COX:31496827-31496827 | C | downstream_gene_variant | MODIFIER | *ATP6V1G2* | Transcript | protein_coding |
| rs2239525 | HSCHR6_MHC_COX:31496827-31496827 | G | intron_variant | MODIFIER | *DDX39B* | Transcript | protein_coding |
| rs2239525 | HSCHR6_MHC_COX:31496827-31496827 | C | intron_variant | MODIFIER | *DDX39B* | Transcript | protein_coding |
| rs2239525 | HSCHR6_MHC_COX:31496827-31496827 | G | intron_variant | MODIFIER | *DDX39B* | Transcript | protein_coding |
| rs2239525 | HSCHR6_MHC_COX:31496827-31496827 | C | intron_variant | MODIFIER | *DDX39B* | Transcript | protein_coding |
| rs2239525 | HSCHR6_MHC_COX:31496827-31496827 | G | intron_variant | MODIFIER | *DDX39B* | Transcript | protein_coding |
| rs2239525 | HSCHR6_MHC_COX:31496827-31496827 | C | intron_variant | MODIFIER | *DDX39B* | Transcript | protein_coding |
| rs2239525 | HSCHR6_MHC_COX:31496827-31496827 | G | downstream_gene_variant | MODIFIER | *ATP6V1G2* | Transcript | processed_transcript |
| rs2239525 | HSCHR6_MHC_COX:31496827-31496827 | C | downstream_gene_variant | MODIFIER | *ATP6V1G2* | Transcript | processed_transcript |
| rs2239525 | HSCHR6_MHC_COX:31496827-31496827 | G | intron_variant | MODIFIER | *DDX39B* | Transcript | protein_coding |
| rs2239525 | HSCHR6_MHC_COX:31496827-31496827 | C | intron_variant | MODIFIER | *DDX39B* | Transcript | protein_coding |
| rs2239525 | HSCHR6_MHC_COX:31496827-31496827 | G | downstream_gene_variant | MODIFIER | *ATP6V1G2* | Transcript | processed_transcript |
| rs2239525 | HSCHR6_MHC_COX:31496827-31496827 | C | downstream_gene_variant | MODIFIER | *ATP6V1G2* | Transcript | processed_transcript |
| rs2239525 | HSCHR6_MHC_COX:31496827-31496827 | G | non_coding_transcript_exon_variant | MODIFIER | *DDX39B* | Transcript | retained_intron |
| rs2239525 | HSCHR6_MHC_COX:31496827-31496827 | C | non_coding_transcript_exon_variant | MODIFIER | *DDX39B* | Transcript | retained_intron |
| rs2239525 | HSCHR6_MHC_COX:31496827-31496827 | G | intron_variant,non_coding_transcript_variant | MODIFIER | *DDX39B* | Transcript | retained_intron |
| rs2239525 | HSCHR6_MHC_COX:31496827-31496827 | C | intron_variant,non_coding_transcript_variant | MODIFIER | *DDX39B* | Transcript | retained_intron |
| rs2239525 | HSCHR6_MHC_COX:31496827-31496827 | G | intron_variant | MODIFIER | *DDX39B* | Transcript | protein_coding |
| rs2239525 | HSCHR6_MHC_COX:31496827-31496827 | C | intron_variant | MODIFIER | *DDX39B* | Transcript | protein_coding |
| rs2239525 | HSCHR6_MHC_COX:31496827-31496827 | G | intron_variant | MODIFIER | *DDX39B* | Transcript | protein_coding |
| rs2239525 | HSCHR6_MHC_COX:31496827-31496827 | C | intron_variant | MODIFIER | *DDX39B* | Transcript | protein_coding |
| rs2239525 | HSCHR6_MHC_COX:31496827-31496827 | G | upstream_gene_variant | MODIFIER | *SNORD83* | Transcript | snoRNA |
| rs2239525 | HSCHR6_MHC_COX:31496827-31496827 | C | upstream_gene_variant | MODIFIER | *SNORD83* | Transcript | snoRNA |
| rs2239525 | HSCHR6_MHC_DBB:31491553-31491553 | G | intron_variant | MODIFIER | *DDX39B* | Transcript | protein_coding |
| rs2239525 | HSCHR6_MHC_DBB:31491553-31491553 | C | intron_variant | MODIFIER | *DDX39B* | Transcript | protein_coding |
| rs2239525 | HSCHR6_MHC_DBB:31491553-31491553 | G | intron_variant | MODIFIER | *DDX39B* | Transcript | protein_coding |
| rs2239525 | HSCHR6_MHC_DBB:31491553-31491553 | C | intron_variant | MODIFIER | *DDX39B* | Transcript | protein_coding |
| rs2239525 | HSCHR6_MHC_DBB:31491553-31491553 | G | intron_variant | MODIFIER | *DDX39B* | Transcript | protein_coding |
| rs2239525 | HSCHR6_MHC_DBB:31491553-31491553 | C | intron_variant | MODIFIER | *DDX39B* | Transcript | protein_coding |
| rs2239525 | HSCHR6_MHC_DBB:31491553-31491553 | G | intron_variant | MODIFIER | *DDX39B* | Transcript | protein_coding |
| rs2239525 | HSCHR6_MHC_DBB:31491553-31491553 | C | intron_variant | MODIFIER | *DDX39B* | Transcript | protein_coding |
| rs2239525 | HSCHR6_MHC_DBB:31491553-31491553 | G | upstream_gene_variant | MODIFIER | *DDX39B* | Transcript | protein_coding |
| rs2239525 | HSCHR6_MHC_DBB:31491553-31491553 | C | upstream_gene_variant | MODIFIER | *DDX39B* | Transcript | protein_coding |
| rs2239525 | HSCHR6_MHC_DBB:31491553-31491553 | G | downstream_gene_variant | MODIFIER | *ATP6V1G2* | Transcript | protein_coding |
| rs2239525 | HSCHR6_MHC_DBB:31491553-31491553 | C | downstream_gene_variant | MODIFIER | *ATP6V1G2* | Transcript | protein_coding |
| rs2239525 | HSCHR6_MHC_DBB:31491553-31491553 | G | upstream_gene_variant | MODIFIER | *DDX39B-AS1* | Transcript | processed_transcript |
| rs2239525 | HSCHR6_MHC_DBB:31491553-31491553 | C | upstream_gene_variant | MODIFIER | *DDX39B-AS1* | Transcript | processed_transcript |
| rs2239525 | HSCHR6_MHC_DBB:31491553-31491553 | G | intron_variant | MODIFIER | *DDX39B* | Transcript | protein_coding |
| rs2239525 | HSCHR6_MHC_DBB:31491553-31491553 | C | intron_variant | MODIFIER | *DDX39B* | Transcript | protein_coding |
| rs2239525 | HSCHR6_MHC_DBB:31491553-31491553 | G | intron_variant | MODIFIER | *DDX39B* | Transcript | protein_coding |
| rs2239525 | HSCHR6_MHC_DBB:31491553-31491553 | C | intron_variant | MODIFIER | *DDX39B* | Transcript | protein_coding |
| rs2239525 | HSCHR6_MHC_DBB:31491553-31491553 | G | upstream_gene_variant | MODIFIER | *DDX39B* | Transcript | protein_coding |
| rs2239525 | HSCHR6_MHC_DBB:31491553-31491553 | C | upstream_gene_variant | MODIFIER | *DDX39B* | Transcript | protein_coding |
| rs2239525 | HSCHR6_MHC_DBB:31491553-31491553 | G | intron_variant | MODIFIER | *DDX39B* | Transcript | protein_coding |
| rs2239525 | HSCHR6_MHC_DBB:31491553-31491553 | C | intron_variant | MODIFIER | *DDX39B* | Transcript | protein_coding |
| rs2239525 | HSCHR6_MHC_DBB:31491553-31491553 | G | intron_variant | MODIFIER | *DDX39B* | Transcript | protein_coding |
| rs2239525 | HSCHR6_MHC_DBB:31491553-31491553 | C | intron_variant | MODIFIER | *DDX39B* | Transcript | protein_coding |
| rs2239525 | HSCHR6_MHC_DBB:31491553-31491553 | G | intron_variant | MODIFIER | *DDX39B* | Transcript | protein_coding |
| rs2239525 | HSCHR6_MHC_DBB:31491553-31491553 | C | intron_variant | MODIFIER | *DDX39B* | Transcript | protein_coding |
| rs2239525 | HSCHR6_MHC_DBB:31491553-31491553 | G | intron_variant | MODIFIER | *DDX39B* | Transcript | protein_coding |
| rs2239525 | HSCHR6_MHC_DBB:31491553-31491553 | C | intron_variant | MODIFIER | *DDX39B* | Transcript | protein_coding |
| rs2239525 | HSCHR6_MHC_DBB:31491553-31491553 | G | intron_variant | MODIFIER | *DDX39B* | Transcript | protein_coding |
| rs2239525 | HSCHR6_MHC_DBB:31491553-31491553 | C | intron_variant | MODIFIER | *DDX39B* | Transcript | protein_coding |
| rs2239525 | HSCHR6_MHC_DBB:31491553-31491553 | G | upstream_gene_variant | MODIFIER | *DDX39B* | Transcript | protein_coding |
| rs2239525 | HSCHR6_MHC_DBB:31491553-31491553 | C | upstream_gene_variant | MODIFIER | *DDX39B* | Transcript | protein_coding |
| rs2239525 | HSCHR6_MHC_DBB:31491553-31491553 | G | upstream_gene_variant | MODIFIER | *DDX39B-AS1* | Transcript | processed_transcript |
| rs2239525 | HSCHR6_MHC_DBB:31491553-31491553 | C | upstream_gene_variant | MODIFIER | *DDX39B-AS1* | Transcript | processed_transcript |
| rs2239525 | HSCHR6_MHC_DBB:31491553-31491553 | G | downstream_gene_variant | MODIFIER | *ATP6V1G2* | Transcript | protein_coding |
| rs2239525 | HSCHR6_MHC_DBB:31491553-31491553 | C | downstream_gene_variant | MODIFIER | *ATP6V1G2* | Transcript | protein_coding |
| rs2239525 | HSCHR6_MHC_DBB:31491553-31491553 | G | intron_variant | MODIFIER | *DDX39B* | Transcript | protein_coding |
| rs2239525 | HSCHR6_MHC_DBB:31491553-31491553 | C | intron_variant | MODIFIER | *DDX39B* | Transcript | protein_coding |
| rs2239525 | HSCHR6_MHC_DBB:31491553-31491553 | G | intron_variant,non_coding_transcript_variant | MODIFIER | *DDX39B* | Transcript | retained_intron |
| rs2239525 | HSCHR6_MHC_DBB:31491553-31491553 | C | intron_variant,non_coding_transcript_variant | MODIFIER | *DDX39B* | Transcript | retained_intron |
| rs2239525 | HSCHR6_MHC_DBB:31491553-31491553 | G | non_coding_transcript_exon_variant | MODIFIER | *DDX39B* | Transcript | retained_intron |
| rs2239525 | HSCHR6_MHC_DBB:31491553-31491553 | C | non_coding_transcript_exon_variant | MODIFIER | *DDX39B* | Transcript | retained_intron |
| rs2239525 | HSCHR6_MHC_DBB:31491553-31491553 | G | downstream_gene_variant | MODIFIER | *ATP6V1G2* | Transcript | processed_transcript |
| rs2239525 | HSCHR6_MHC_DBB:31491553-31491553 | C | downstream_gene_variant | MODIFIER | *ATP6V1G2* | Transcript | processed_transcript |
| rs2239525 | HSCHR6_MHC_DBB:31491553-31491553 | G | intron_variant,non_coding_transcript_variant | MODIFIER | *DDX39B* | Transcript | processed_transcript |
| rs2239525 | HSCHR6_MHC_DBB:31491553-31491553 | C | intron_variant,non_coding_transcript_variant | MODIFIER | *DDX39B* | Transcript | processed_transcript |
| rs2239525 | HSCHR6_MHC_DBB:31491553-31491553 | G | downstream_gene_variant | MODIFIER | *ATP6V1G2* | Transcript | processed_transcript |
| rs2239525 | HSCHR6_MHC_DBB:31491553-31491553 | C | downstream_gene_variant | MODIFIER | *ATP6V1G2* | Transcript | processed_transcript |
| rs2239525 | HSCHR6_MHC_DBB:31491553-31491553 | G | intron_variant | MODIFIER | *DDX39B* | Transcript | protein_coding |
| rs2239525 | HSCHR6_MHC_DBB:31491553-31491553 | C | intron_variant | MODIFIER | *DDX39B* | Transcript | protein_coding |
| rs2239525 | HSCHR6_MHC_DBB:31491553-31491553 | G | intron_variant | MODIFIER | *DDX39B* | Transcript | protein_coding |
| rs2239525 | HSCHR6_MHC_DBB:31491553-31491553 | C | intron_variant | MODIFIER | *DDX39B* | Transcript | protein_coding |
| rs2239525 | HSCHR6_MHC_DBB:31491553-31491553 | G | upstream_gene_variant | MODIFIER | *SNORD83* | Transcript | snoRNA |
| rs2239525 | HSCHR6_MHC_DBB:31491553-31491553 | C | upstream_gene_variant | MODIFIER | *SNORD83* | Transcript | snoRNA |
| rs2239525 | HSCHR6_MHC_MANN:31548871-31548871 | G | intron_variant | MODIFIER | *DDX39B* | Transcript | protein_coding |
| rs2239525 | HSCHR6_MHC_MANN:31548871-31548871 | C | intron_variant | MODIFIER | *DDX39B* | Transcript | protein_coding |
| rs2239525 | HSCHR6_MHC_MANN:31548871-31548871 | G | intron_variant | MODIFIER | *DDX39B* | Transcript | protein_coding |
| rs2239525 | HSCHR6_MHC_MANN:31548871-31548871 | C | intron_variant | MODIFIER | *DDX39B* | Transcript | protein_coding |
| rs2239525 | HSCHR6_MHC_MANN:31548871-31548871 | G | intron_variant | MODIFIER | *DDX39B* | Transcript | protein_coding |
| rs2239525 | HSCHR6_MHC_MANN:31548871-31548871 | C | intron_variant | MODIFIER | *DDX39B* | Transcript | protein_coding |
| rs2239525 | HSCHR6_MHC_MANN:31548871-31548871 | G | upstream_gene_variant | MODIFIER | *DDX39B* | Transcript | protein_coding |
| rs2239525 | HSCHR6_MHC_MANN:31548871-31548871 | C | upstream_gene_variant | MODIFIER | *DDX39B* | Transcript | protein_coding |
| rs2239525 | HSCHR6_MHC_MANN:31548871-31548871 | G | upstream_gene_variant | MODIFIER | *DDX39B-AS1* | Transcript | processed_transcript |
| rs2239525 | HSCHR6_MHC_MANN:31548871-31548871 | C | upstream_gene_variant | MODIFIER | *DDX39B-AS1* | Transcript | processed_transcript |
| rs2239525 | HSCHR6_MHC_MANN:31548871-31548871 | G | intron_variant | MODIFIER | *DDX39B* | Transcript | protein_coding |
| rs2239525 | HSCHR6_MHC_MANN:31548871-31548871 | C | intron_variant | MODIFIER | *DDX39B* | Transcript | protein_coding |
| rs2239525 | HSCHR6_MHC_MANN:31548871-31548871 | G | downstream_gene_variant | MODIFIER | *ATP6V1G2* | Transcript | protein_coding |
| rs2239525 | HSCHR6_MHC_MANN:31548871-31548871 | C | downstream_gene_variant | MODIFIER | *ATP6V1G2* | Transcript | protein_coding |
| rs2239525 | HSCHR6_MHC_MANN:31548871-31548871 | G | intron_variant | MODIFIER | *DDX39B* | Transcript | protein_coding |
| rs2239525 | HSCHR6_MHC_MANN:31548871-31548871 | C | intron_variant | MODIFIER | *DDX39B* | Transcript | protein_coding |
| rs2239525 | HSCHR6_MHC_MANN:31548871-31548871 | G | intron_variant | MODIFIER | *DDX39B* | Transcript | protein_coding |
| rs2239525 | HSCHR6_MHC_MANN:31548871-31548871 | C | intron_variant | MODIFIER | *DDX39B* | Transcript | protein_coding |
| rs2239525 | HSCHR6_MHC_MANN:31548871-31548871 | G | intron_variant | MODIFIER | *DDX39B* | Transcript | protein_coding |
| rs2239525 | HSCHR6_MHC_MANN:31548871-31548871 | C | intron_variant | MODIFIER | *DDX39B* | Transcript | protein_coding |
| rs2239525 | HSCHR6_MHC_MANN:31548871-31548871 | G | intron_variant | MODIFIER | *DDX39B* | Transcript | protein_coding |
| rs2239525 | HSCHR6_MHC_MANN:31548871-31548871 | C | intron_variant | MODIFIER | *DDX39B* | Transcript | protein_coding |
| rs2239525 | HSCHR6_MHC_MANN:31548871-31548871 | G | intron_variant | MODIFIER | *DDX39B* | Transcript | protein_coding |
| rs2239525 | HSCHR6_MHC_MANN:31548871-31548871 | C | intron_variant | MODIFIER | *DDX39B* | Transcript | protein_coding |
| rs2239525 | HSCHR6_MHC_MANN:31548871-31548871 | G | upstream_gene_variant | MODIFIER | *DDX39B* | Transcript | protein_coding |
| rs2239525 | HSCHR6_MHC_MANN:31548871-31548871 | C | upstream_gene_variant | MODIFIER | *DDX39B* | Transcript | protein_coding |
| rs2239525 | HSCHR6_MHC_MANN:31548871-31548871 | G | upstream_gene_variant | MODIFIER | *DDX39B-AS1* | Transcript | processed_transcript |
| rs2239525 | HSCHR6_MHC_MANN:31548871-31548871 | C | upstream_gene_variant | MODIFIER | *DDX39B-AS1* | Transcript | processed_transcript |
| rs2239525 | HSCHR6_MHC_MANN:31548871-31548871 | G | intron_variant | MODIFIER | *DDX39B* | Transcript | protein_coding |
| rs2239525 | HSCHR6_MHC_MANN:31548871-31548871 | C | intron_variant | MODIFIER | *DDX39B* | Transcript | protein_coding |
| rs2239525 | HSCHR6_MHC_MANN:31548871-31548871 | G | intron_variant | MODIFIER | *DDX39B* | Transcript | protein_coding |
| rs2239525 | HSCHR6_MHC_MANN:31548871-31548871 | C | intron_variant | MODIFIER | *DDX39B* | Transcript | protein_coding |
| rs2239525 | HSCHR6_MHC_MANN:31548871-31548871 | G | intron_variant | MODIFIER | *DDX39B* | Transcript | protein_coding |
| rs2239525 | HSCHR6_MHC_MANN:31548871-31548871 | C | intron_variant | MODIFIER | *DDX39B* | Transcript | protein_coding |
| rs2239525 | HSCHR6_MHC_MANN:31548871-31548871 | G | downstream_gene_variant | MODIFIER | *ATP6V1G2* | Transcript | protein_coding |
| rs2239525 | HSCHR6_MHC_MANN:31548871-31548871 | C | downstream_gene_variant | MODIFIER | *ATP6V1G2* | Transcript | protein_coding |
| rs2239525 | HSCHR6_MHC_MANN:31548871-31548871 | G | upstream_gene_variant | MODIFIER | *DDX39B* | Transcript | protein_coding |
| rs2239525 | HSCHR6_MHC_MANN:31548871-31548871 | C | upstream_gene_variant | MODIFIER | *DDX39B* | Transcript | protein_coding |
| rs2239525 | HSCHR6_MHC_MANN:31548871-31548871 | G | downstream_gene_variant | MODIFIER | *ATP6V1G2* | Transcript | processed_transcript |
| rs2239525 | HSCHR6_MHC_MANN:31548871-31548871 | C | downstream_gene_variant | MODIFIER | *ATP6V1G2* | Transcript | processed_transcript |
| rs2239525 | HSCHR6_MHC_MANN:31548871-31548871 | G | downstream_gene_variant | MODIFIER | *ATP6V1G2* | Transcript | processed_transcript |
| rs2239525 | HSCHR6_MHC_MANN:31548871-31548871 | C | downstream_gene_variant | MODIFIER | *ATP6V1G2* | Transcript | processed_transcript |
| rs2239525 | HSCHR6_MHC_MANN:31548871-31548871 | G | non_coding_transcript_exon_variant | MODIFIER | *DDX39B* | Transcript | retained_intron |
| rs2239525 | HSCHR6_MHC_MANN:31548871-31548871 | C | non_coding_transcript_exon_variant | MODIFIER | *DDX39B* | Transcript | retained_intron |
| rs2239525 | HSCHR6_MHC_MANN:31548871-31548871 | G | intron_variant,non_coding_transcript_variant | MODIFIER | *DDX39B* | Transcript | retained_intron |
| rs2239525 | HSCHR6_MHC_MANN:31548871-31548871 | C | intron_variant,non_coding_transcript_variant | MODIFIER | *DDX39B* | Transcript | retained_intron |
| rs2239525 | HSCHR6_MHC_MANN:31548871-31548871 | G | intron_variant,non_coding_transcript_variant | MODIFIER | *DDX39B* | Transcript | processed_transcript |
| rs2239525 | HSCHR6_MHC_MANN:31548871-31548871 | C | intron_variant,non_coding_transcript_variant | MODIFIER | *DDX39B* | Transcript | processed_transcript |
| rs2239525 | HSCHR6_MHC_MANN:31548871-31548871 | G | intron_variant | MODIFIER | *DDX39B* | Transcript | protein_coding |
| rs2239525 | HSCHR6_MHC_MANN:31548871-31548871 | C | intron_variant | MODIFIER | *DDX39B* | Transcript | protein_coding |
| rs2239525 | HSCHR6_MHC_MANN:31548871-31548871 | G | intron_variant | MODIFIER | *DDX39B* | Transcript | protein_coding |
| rs2239525 | HSCHR6_MHC_MANN:31548871-31548871 | C | intron_variant | MODIFIER | *DDX39B* | Transcript | protein_coding |
| rs2239525 | HSCHR6_MHC_MANN:31548871-31548871 | G | upstream_gene_variant | MODIFIER | *SNORD83* | Transcript | snoRNA |
| rs2239525 | HSCHR6_MHC_MANN:31548871-31548871 | C | upstream_gene_variant | MODIFIER | *SNORD83* | Transcript | snoRNA |
| rs2239525 | HSCHR6_MHC_MCF:31585722-31585722 | G | intron_variant | MODIFIER | *DDX39B* | Transcript | protein_coding |
| rs2239525 | HSCHR6_MHC_MCF:31585722-31585722 | C | intron_variant | MODIFIER | *DDX39B* | Transcript | protein_coding |
| rs2239525 | HSCHR6_MHC_MCF:31585722-31585722 | G | upstream_gene_variant | MODIFIER | *DDX39B* | Transcript | protein_coding |
| rs2239525 | HSCHR6_MHC_MCF:31585722-31585722 | C | upstream_gene_variant | MODIFIER | *DDX39B* | Transcript | protein_coding |
| rs2239525 | HSCHR6_MHC_MCF:31585722-31585722 | G | intron_variant | MODIFIER | *DDX39B* | Transcript | protein_coding |
| rs2239525 | HSCHR6_MHC_MCF:31585722-31585722 | C | intron_variant | MODIFIER | *DDX39B* | Transcript | protein_coding |
| rs2239525 | HSCHR6_MHC_MCF:31585722-31585722 | G | intron_variant | MODIFIER | *DDX39B* | Transcript | protein_coding |
| rs2239525 | HSCHR6_MHC_MCF:31585722-31585722 | C | intron_variant | MODIFIER | *DDX39B* | Transcript | protein_coding |
| rs2239525 | HSCHR6_MHC_MCF:31585722-31585722 | G | intron_variant | MODIFIER | *DDX39B* | Transcript | protein_coding |
| rs2239525 | HSCHR6_MHC_MCF:31585722-31585722 | C | intron_variant | MODIFIER | *DDX39B* | Transcript | protein_coding |
| rs2239525 | HSCHR6_MHC_MCF:31585722-31585722 | G | intron_variant | MODIFIER | *DDX39B* | Transcript | protein_coding |
| rs2239525 | HSCHR6_MHC_MCF:31585722-31585722 | C | intron_variant | MODIFIER | *DDX39B* | Transcript | protein_coding |
| rs2239525 | HSCHR6_MHC_MCF:31585722-31585722 | G | intron_variant | MODIFIER | *DDX39B* | Transcript | protein_coding |
| rs2239525 | HSCHR6_MHC_MCF:31585722-31585722 | C | intron_variant | MODIFIER | *DDX39B* | Transcript | protein_coding |
| rs2239525 | HSCHR6_MHC_MCF:31585722-31585722 | G | upstream_gene_variant | MODIFIER | *DDX39B* | Transcript | protein_coding |
| rs2239525 | HSCHR6_MHC_MCF:31585722-31585722 | C | upstream_gene_variant | MODIFIER | *DDX39B* | Transcript | protein_coding |
| rs2239525 | HSCHR6_MHC_MCF:31585722-31585722 | G | intron_variant | MODIFIER | *DDX39B* | Transcript | protein_coding |
| rs2239525 | HSCHR6_MHC_MCF:31585722-31585722 | C | intron_variant | MODIFIER | *DDX39B* | Transcript | protein_coding |
| rs2239525 | HSCHR6_MHC_MCF:31585722-31585722 | G | intron_variant | MODIFIER | *DDX39B* | Transcript | protein_coding |
| rs2239525 | HSCHR6_MHC_MCF:31585722-31585722 | C | intron_variant | MODIFIER | *DDX39B* | Transcript | protein_coding |
| rs2239525 | HSCHR6_MHC_MCF:31585722-31585722 | G | upstream_gene_variant | MODIFIER | *DDX39B-AS1* | Transcript | processed_transcript |
| rs2239525 | HSCHR6_MHC_MCF:31585722-31585722 | C | upstream_gene_variant | MODIFIER | *DDX39B-AS1* | Transcript | processed_transcript |
| rs2239525 | HSCHR6_MHC_MCF:31585722-31585722 | G | upstream_gene_variant | MODIFIER | *DDX39B* | Transcript | protein_coding |
| rs2239525 | HSCHR6_MHC_MCF:31585722-31585722 | C | upstream_gene_variant | MODIFIER | *DDX39B* | Transcript | protein_coding |
| rs2239525 | HSCHR6_MHC_MCF:31585722-31585722 | G | downstream_gene_variant | MODIFIER | *ATP6V1G2* | Transcript | protein_coding |
| rs2239525 | HSCHR6_MHC_MCF:31585722-31585722 | C | downstream_gene_variant | MODIFIER | *ATP6V1G2* | Transcript | protein_coding |
| rs2239525 | HSCHR6_MHC_MCF:31585722-31585722 | G | upstream_gene_variant | MODIFIER | *DDX39B-AS1* | Transcript | processed_transcript |
| rs2239525 | HSCHR6_MHC_MCF:31585722-31585722 | C | upstream_gene_variant | MODIFIER | *DDX39B-AS1* | Transcript | processed_transcript |
| rs2239525 | HSCHR6_MHC_MCF:31585722-31585722 | G | intron_variant | MODIFIER | *DDX39B* | Transcript | protein_coding |
| rs2239525 | HSCHR6_MHC_MCF:31585722-31585722 | C | intron_variant | MODIFIER | *DDX39B* | Transcript | protein_coding |
| rs2239525 | HSCHR6_MHC_MCF:31585722-31585722 | G | intron_variant | MODIFIER | *DDX39B* | Transcript | protein_coding |
| rs2239525 | HSCHR6_MHC_MCF:31585722-31585722 | C | intron_variant | MODIFIER | *DDX39B* | Transcript | protein_coding |
| rs2239525 | HSCHR6_MHC_MCF:31585722-31585722 | G | intron_variant | MODIFIER | *DDX39B* | Transcript | protein_coding |
| rs2239525 | HSCHR6_MHC_MCF:31585722-31585722 | C | intron_variant | MODIFIER | *DDX39B* | Transcript | protein_coding |
| rs2239525 | HSCHR6_MHC_MCF:31585722-31585722 | G | downstream_gene_variant | MODIFIER | *ATP6V1G2* | Transcript | protein_coding |
| rs2239525 | HSCHR6_MHC_MCF:31585722-31585722 | C | downstream_gene_variant | MODIFIER | *ATP6V1G2* | Transcript | protein_coding |
| rs2239525 | HSCHR6_MHC_MCF:31585722-31585722 | G | intron_variant | MODIFIER | *DDX39B* | Transcript | protein_coding |
| rs2239525 | HSCHR6_MHC_MCF:31585722-31585722 | C | intron_variant | MODIFIER | *DDX39B* | Transcript | protein_coding |
| rs2239525 | HSCHR6_MHC_MCF:31585722-31585722 | G | intron_variant,non_coding_transcript_variant | MODIFIER | *DDX39B* | Transcript | processed_transcript |
| rs2239525 | HSCHR6_MHC_MCF:31585722-31585722 | C | intron_variant,non_coding_transcript_variant | MODIFIER | *DDX39B* | Transcript | processed_transcript |
| rs2239525 | HSCHR6_MHC_MCF:31585722-31585722 | G | downstream_gene_variant | MODIFIER | *ATP6V1G2* | Transcript | processed_transcript |
| rs2239525 | HSCHR6_MHC_MCF:31585722-31585722 | C | downstream_gene_variant | MODIFIER | *ATP6V1G2* | Transcript | processed_transcript |
| rs2239525 | HSCHR6_MHC_MCF:31585722-31585722 | G | downstream_gene_variant | MODIFIER | *ATP6V1G2* | Transcript | processed_transcript |
| rs2239525 | HSCHR6_MHC_MCF:31585722-31585722 | C | downstream_gene_variant | MODIFIER | *ATP6V1G2* | Transcript | processed_transcript |
| rs2239525 | HSCHR6_MHC_MCF:31585722-31585722 | G | intron_variant,non_coding_transcript_variant | MODIFIER | *DDX39B* | Transcript | retained_intron |
| rs2239525 | HSCHR6_MHC_MCF:31585722-31585722 | C | intron_variant,non_coding_transcript_variant | MODIFIER | *DDX39B* | Transcript | retained_intron |
| rs2239525 | HSCHR6_MHC_MCF:31585722-31585722 | G | non_coding_transcript_exon_variant | MODIFIER | *DDX39B* | Transcript | retained_intron |
| rs2239525 | HSCHR6_MHC_MCF:31585722-31585722 | C | non_coding_transcript_exon_variant | MODIFIER | *DDX39B* | Transcript | retained_intron |
| rs2239525 | HSCHR6_MHC_MCF:31585722-31585722 | G | intron_variant | MODIFIER | *DDX39B* | Transcript | protein_coding |
| rs2239525 | HSCHR6_MHC_MCF:31585722-31585722 | C | intron_variant | MODIFIER | *DDX39B* | Transcript | protein_coding |
| rs2239525 | HSCHR6_MHC_MCF:31585722-31585722 | G | intron_variant | MODIFIER | *DDX39B* | Transcript | protein_coding |
| rs2239525 | HSCHR6_MHC_MCF:31585722-31585722 | C | intron_variant | MODIFIER | *DDX39B* | Transcript | protein_coding |
| rs2239525 | HSCHR6_MHC_MCF:31585722-31585722 | G | downstream_gene_variant | MODIFIER | *ATP6V1G2* | Transcript | protein_coding |
| rs2239525 | HSCHR6_MHC_MCF:31585722-31585722 | C | downstream_gene_variant | MODIFIER | *ATP6V1G2* | Transcript | protein_coding |
| rs2239525 | HSCHR6_MHC_MCF:31585722-31585722 | G | upstream_gene_variant | MODIFIER | *SNORD83* | Transcript | snoRNA |
| rs2239525 | HSCHR6_MHC_MCF:31585722-31585722 | C | upstream_gene_variant | MODIFIER | *SNORD83* | Transcript | snoRNA |
| rs2239525 | HSCHR6_MHC_QBL:31499650-31499650 | G | downstream_gene_variant | MODIFIER | *ATP6V1G2* | Transcript | protein_coding |
| rs2239525 | HSCHR6_MHC_QBL:31499650-31499650 | C | downstream_gene_variant | MODIFIER | *ATP6V1G2* | Transcript | protein_coding |
| rs2239525 | HSCHR6_MHC_QBL:31499650-31499650 | G | intron_variant | MODIFIER | *DDX39B* | Transcript | protein_coding |
| rs2239525 | HSCHR6_MHC_QBL:31499650-31499650 | C | intron_variant | MODIFIER | *DDX39B* | Transcript | protein_coding |
| rs2239525 | HSCHR6_MHC_QBL:31499650-31499650 | G | upstream_gene_variant | MODIFIER | *DDX39B* | Transcript | protein_coding |
| rs2239525 | HSCHR6_MHC_QBL:31499650-31499650 | C | upstream_gene_variant | MODIFIER | *DDX39B* | Transcript | protein_coding |
| rs2239525 | HSCHR6_MHC_QBL:31499650-31499650 | G | intron_variant | MODIFIER | *DDX39B* | Transcript | protein_coding |
| rs2239525 | HSCHR6_MHC_QBL:31499650-31499650 | C | intron_variant | MODIFIER | *DDX39B* | Transcript | protein_coding |
| rs2239525 | HSCHR6_MHC_QBL:31499650-31499650 | G | intron_variant | MODIFIER | *DDX39B* | Transcript | protein_coding |
| rs2239525 | HSCHR6_MHC_QBL:31499650-31499650 | C | intron_variant | MODIFIER | *DDX39B* | Transcript | protein_coding |
| rs2239525 | HSCHR6_MHC_QBL:31499650-31499650 | G | upstream_gene_variant | MODIFIER | *DDX39B-AS1* | Transcript | processed_transcript |
| rs2239525 | HSCHR6_MHC_QBL:31499650-31499650 | C | upstream_gene_variant | MODIFIER | *DDX39B-AS1* | Transcript | processed_transcript |
| rs2239525 | HSCHR6_MHC_QBL:31499650-31499650 | G | upstream_gene_variant | MODIFIER | *DDX39B-AS1* | Transcript | processed_transcript |
| rs2239525 | HSCHR6_MHC_QBL:31499650-31499650 | C | upstream_gene_variant | MODIFIER | *DDX39B-AS1* | Transcript | processed_transcript |
| rs2239525 | HSCHR6_MHC_QBL:31499650-31499650 | G | intron_variant | MODIFIER | *DDX39B* | Transcript | protein_coding |
| rs2239525 | HSCHR6_MHC_QBL:31499650-31499650 | C | intron_variant | MODIFIER | *DDX39B* | Transcript | protein_coding |
| rs2239525 | HSCHR6_MHC_QBL:31499650-31499650 | G | intron_variant | MODIFIER | *DDX39B* | Transcript | protein_coding |
| rs2239525 | HSCHR6_MHC_QBL:31499650-31499650 | C | intron_variant | MODIFIER | *DDX39B* | Transcript | protein_coding |
| rs2239525 | HSCHR6_MHC_QBL:31499650-31499650 | G | intron_variant | MODIFIER | *DDX39B* | Transcript | protein_coding |
| rs2239525 | HSCHR6_MHC_QBL:31499650-31499650 | C | intron_variant | MODIFIER | *DDX39B* | Transcript | protein_coding |
| rs2239525 | HSCHR6_MHC_QBL:31499650-31499650 | G | intron_variant | MODIFIER | *DDX39B* | Transcript | protein_coding |
| rs2239525 | HSCHR6_MHC_QBL:31499650-31499650 | C | intron_variant | MODIFIER | *DDX39B* | Transcript | protein_coding |
| rs2239525 | HSCHR6_MHC_QBL:31499650-31499650 | G | upstream_gene_variant | MODIFIER | *DDX39B* | Transcript | protein_coding |
| rs2239525 | HSCHR6_MHC_QBL:31499650-31499650 | C | upstream_gene_variant | MODIFIER | *DDX39B* | Transcript | protein_coding |
| rs2239525 | HSCHR6_MHC_QBL:31499650-31499650 | G | intron_variant | MODIFIER | *DDX39B* | Transcript | protein_coding |
| rs2239525 | HSCHR6_MHC_QBL:31499650-31499650 | C | intron_variant | MODIFIER | *DDX39B* | Transcript | protein_coding |
| rs2239525 | HSCHR6_MHC_QBL:31499650-31499650 | G | intron_variant | MODIFIER | *DDX39B* | Transcript | protein_coding |
| rs2239525 | HSCHR6_MHC_QBL:31499650-31499650 | C | intron_variant | MODIFIER | *DDX39B* | Transcript | protein_coding |
| rs2239525 | HSCHR6_MHC_QBL:31499650-31499650 | G | intron_variant | MODIFIER | *DDX39B* | Transcript | protein_coding |
| rs2239525 | HSCHR6_MHC_QBL:31499650-31499650 | C | intron_variant | MODIFIER | *DDX39B* | Transcript | protein_coding |
| rs2239525 | HSCHR6_MHC_QBL:31499650-31499650 | G | intron_variant | MODIFIER | *DDX39B* | Transcript | protein_coding |
| rs2239525 | HSCHR6_MHC_QBL:31499650-31499650 | C | intron_variant | MODIFIER | *DDX39B* | Transcript | protein_coding |
| rs2239525 | HSCHR6_MHC_QBL:31499650-31499650 | G | upstream_gene_variant | MODIFIER | *DDX39B* | Transcript | protein_coding |
| rs2239525 | HSCHR6_MHC_QBL:31499650-31499650 | C | upstream_gene_variant | MODIFIER | *DDX39B* | Transcript | protein_coding |
| rs2239525 | HSCHR6_MHC_QBL:31499650-31499650 | G | intron_variant | MODIFIER | *DDX39B* | Transcript | protein_coding |
| rs2239525 | HSCHR6_MHC_QBL:31499650-31499650 | C | intron_variant | MODIFIER | *DDX39B* | Transcript | protein_coding |
| rs2239525 | HSCHR6_MHC_QBL:31499650-31499650 | G | downstream_gene_variant | MODIFIER | *ATP6V1G2* | Transcript | protein_coding |
| rs2239525 | HSCHR6_MHC_QBL:31499650-31499650 | C | downstream_gene_variant | MODIFIER | *ATP6V1G2* | Transcript | protein_coding |
| rs2239525 | HSCHR6_MHC_QBL:31499650-31499650 | G | non_coding_transcript_exon_variant | MODIFIER | *DDX39B* | Transcript | retained_intron |
| rs2239525 | HSCHR6_MHC_QBL:31499650-31499650 | C | non_coding_transcript_exon_variant | MODIFIER | *DDX39B* | Transcript | retained_intron |
| rs2239525 | HSCHR6_MHC_QBL:31499650-31499650 | G | downstream_gene_variant | MODIFIER | *ATP6V1G2* | Transcript | processed_transcript |
| rs2239525 | HSCHR6_MHC_QBL:31499650-31499650 | C | downstream_gene_variant | MODIFIER | *ATP6V1G2* | Transcript | processed_transcript |
| rs2239525 | HSCHR6_MHC_QBL:31499650-31499650 | G | downstream_gene_variant | MODIFIER | *ATP6V1G2* | Transcript | processed_transcript |
| rs2239525 | HSCHR6_MHC_QBL:31499650-31499650 | C | downstream_gene_variant | MODIFIER | *ATP6V1G2* | Transcript | processed_transcript |
| rs2239525 | HSCHR6_MHC_QBL:31499650-31499650 | G | intron_variant,non_coding_transcript_variant | MODIFIER | *DDX39B* | Transcript | retained_intron |
| rs2239525 | HSCHR6_MHC_QBL:31499650-31499650 | C | intron_variant,non_coding_transcript_variant | MODIFIER | *DDX39B* | Transcript | retained_intron |
| rs2239525 | HSCHR6_MHC_QBL:31499650-31499650 | G | intron_variant,non_coding_transcript_variant | MODIFIER | *DDX39B* | Transcript | processed_transcript |
| rs2239525 | HSCHR6_MHC_QBL:31499650-31499650 | C | intron_variant,non_coding_transcript_variant | MODIFIER | *DDX39B* | Transcript | processed_transcript |
| rs2239525 | HSCHR6_MHC_QBL:31499650-31499650 | G | intron_variant | MODIFIER | *DDX39B* | Transcript | protein_coding |
| rs2239525 | HSCHR6_MHC_QBL:31499650-31499650 | C | intron_variant | MODIFIER | *DDX39B* | Transcript | protein_coding |
| rs2239525 | HSCHR6_MHC_QBL:31499650-31499650 | G | intron_variant | MODIFIER | *DDX39B* | Transcript | protein_coding |
| rs2239525 | HSCHR6_MHC_QBL:31499650-31499650 | C | intron_variant | MODIFIER | *DDX39B* | Transcript | protein_coding |
| rs2239525 | HSCHR6_MHC_QBL:31499650-31499650 | G | upstream_gene_variant | MODIFIER | *SNORD83* | Transcript | snoRNA |
| rs2239525 | HSCHR6_MHC_QBL:31499650-31499650 | C | upstream_gene_variant | MODIFIER | *SNORD83* | Transcript | snoRNA |
| rs2239525 | HSCHR6_MHC_SSTO:31499319-31499319 | A | intron_variant | MODIFIER | *DDX39B* | Transcript | protein_coding |
| rs2239525 | HSCHR6_MHC_SSTO:31499319-31499319 | C | intron_variant | MODIFIER | *DDX39B* | Transcript | protein_coding |
| rs2239525 | HSCHR6_MHC_SSTO:31499319-31499319 | A | intron_variant | MODIFIER | *DDX39B* | Transcript | protein_coding |
| rs2239525 | HSCHR6_MHC_SSTO:31499319-31499319 | C | intron_variant | MODIFIER | *DDX39B* | Transcript | protein_coding |
| rs2239525 | HSCHR6_MHC_SSTO:31499319-31499319 | A | upstream_gene_variant | MODIFIER | *DDX39B-AS1* | Transcript | processed_transcript |
| rs2239525 | HSCHR6_MHC_SSTO:31499319-31499319 | C | upstream_gene_variant | MODIFIER | *DDX39B-AS1* | Transcript | processed_transcript |
| rs2239525 | HSCHR6_MHC_SSTO:31499319-31499319 | A | intron_variant | MODIFIER | *DDX39B* | Transcript | protein_coding |
| rs2239525 | HSCHR6_MHC_SSTO:31499319-31499319 | C | intron_variant | MODIFIER | *DDX39B* | Transcript | protein_coding |
| rs2239525 | HSCHR6_MHC_SSTO:31499319-31499319 | A | intron_variant | MODIFIER | *DDX39B* | Transcript | protein_coding |
| rs2239525 | HSCHR6_MHC_SSTO:31499319-31499319 | C | intron_variant | MODIFIER | *DDX39B* | Transcript | protein_coding |
| rs2239525 | HSCHR6_MHC_SSTO:31499319-31499319 | A | intron_variant | MODIFIER | *DDX39B* | Transcript | protein_coding |
| rs2239525 | HSCHR6_MHC_SSTO:31499319-31499319 | C | intron_variant | MODIFIER | *DDX39B* | Transcript | protein_coding |
| rs2239525 | HSCHR6_MHC_SSTO:31499319-31499319 | A | intron_variant | MODIFIER | *DDX39B* | Transcript | protein_coding |
| rs2239525 | HSCHR6_MHC_SSTO:31499319-31499319 | C | intron_variant | MODIFIER | *DDX39B* | Transcript | protein_coding |
| rs2239525 | HSCHR6_MHC_SSTO:31499319-31499319 | A | upstream_gene_variant | MODIFIER | *DDX39B* | Transcript | protein_coding |
| rs2239525 | HSCHR6_MHC_SSTO:31499319-31499319 | C | upstream_gene_variant | MODIFIER | *DDX39B* | Transcript | protein_coding |
| rs2239525 | HSCHR6_MHC_SSTO:31499319-31499319 | A | upstream_gene_variant | MODIFIER | *DDX39B* | Transcript | protein_coding |
| rs2239525 | HSCHR6_MHC_SSTO:31499319-31499319 | C | upstream_gene_variant | MODIFIER | *DDX39B* | Transcript | protein_coding |
| rs2239525 | HSCHR6_MHC_SSTO:31499319-31499319 | A | downstream_gene_variant | MODIFIER | *ATP6V1G2* | Transcript | protein_coding |
| rs2239525 | HSCHR6_MHC_SSTO:31499319-31499319 | C | downstream_gene_variant | MODIFIER | *ATP6V1G2* | Transcript | protein_coding |
| rs2239525 | HSCHR6_MHC_SSTO:31499319-31499319 | A | intron_variant | MODIFIER | *DDX39B* | Transcript | protein_coding |
| rs2239525 | HSCHR6_MHC_SSTO:31499319-31499319 | C | intron_variant | MODIFIER | *DDX39B* | Transcript | protein_coding |
| rs2239525 | HSCHR6_MHC_SSTO:31499319-31499319 | A | intron_variant | MODIFIER | *DDX39B* | Transcript | protein_coding |
| rs2239525 | HSCHR6_MHC_SSTO:31499319-31499319 | C | intron_variant | MODIFIER | *DDX39B* | Transcript | protein_coding |
| rs2239525 | HSCHR6_MHC_SSTO:31499319-31499319 | A | downstream_gene_variant | MODIFIER | *ATP6V1G2* | Transcript | protein_coding |
| rs2239525 | HSCHR6_MHC_SSTO:31499319-31499319 | C | downstream_gene_variant | MODIFIER | *ATP6V1G2* | Transcript | protein_coding |
| rs2239525 | HSCHR6_MHC_SSTO:31499319-31499319 | A | intron_variant | MODIFIER | *DDX39B* | Transcript | protein_coding |
| rs2239525 | HSCHR6_MHC_SSTO:31499319-31499319 | C | intron_variant | MODIFIER | *DDX39B* | Transcript | protein_coding |
| rs2239525 | HSCHR6_MHC_SSTO:31499319-31499319 | A | upstream_gene_variant | MODIFIER | *DDX39B* | Transcript | protein_coding |
| rs2239525 | HSCHR6_MHC_SSTO:31499319-31499319 | C | upstream_gene_variant | MODIFIER | *DDX39B* | Transcript | protein_coding |
| rs2239525 | HSCHR6_MHC_SSTO:31499319-31499319 | A | intron_variant,NMD_transcript_variant | MODIFIER | *DASS-161H22.6* | Transcript | nonsense_mediated_decay |
| rs2239525 | HSCHR6_MHC_SSTO:31499319-31499319 | C | intron_variant,NMD_transcript_variant | MODIFIER | *DASS-161H22.6* | Transcript | nonsense_mediated_decay |
| rs2239525 | HSCHR6_MHC_SSTO:31499319-31499319 | A | intron_variant | MODIFIER | *DDX39B* | Transcript | protein_coding |
| rs2239525 | HSCHR6_MHC_SSTO:31499319-31499319 | C | intron_variant | MODIFIER | *DDX39B* | Transcript | protein_coding |
| rs2239525 | HSCHR6_MHC_SSTO:31499319-31499319 | A | upstream_gene_variant | MODIFIER | *DDX39B-AS1* | Transcript | processed_transcript |
| rs2239525 | HSCHR6_MHC_SSTO:31499319-31499319 | C | upstream_gene_variant | MODIFIER | *DDX39B-AS1* | Transcript | processed_transcript |
| rs2239525 | HSCHR6_MHC_SSTO:31499319-31499319 | A | intron_variant | MODIFIER | *DDX39B* | Transcript | protein_coding |
| rs2239525 | HSCHR6_MHC_SSTO:31499319-31499319 | C | intron_variant | MODIFIER | *DDX39B* | Transcript | protein_coding |
| rs2239525 | HSCHR6_MHC_SSTO:31499319-31499319 | A | downstream_gene_variant | MODIFIER | *ATP6V1G2* | Transcript | processed_transcript |
| rs2239525 | HSCHR6_MHC_SSTO:31499319-31499319 | C | downstream_gene_variant | MODIFIER | *ATP6V1G2* | Transcript | processed_transcript |
| rs2239525 | HSCHR6_MHC_SSTO:31499319-31499319 | A | downstream_gene_variant | MODIFIER | *ATP6V1G2* | Transcript | processed_transcript |
| rs2239525 | HSCHR6_MHC_SSTO:31499319-31499319 | C | downstream_gene_variant | MODIFIER | *ATP6V1G2* | Transcript | processed_transcript |
| rs2239525 | HSCHR6_MHC_SSTO:31499319-31499319 | A | intron_variant,non_coding_transcript_variant | MODIFIER | *DDX39B* | Transcript | retained_intron |
| rs2239525 | HSCHR6_MHC_SSTO:31499319-31499319 | C | intron_variant,non_coding_transcript_variant | MODIFIER | *DDX39B* | Transcript | retained_intron |
| rs2239525 | HSCHR6_MHC_SSTO:31499319-31499319 | A | intron_variant,non_coding_transcript_variant | MODIFIER | *DDX39B* | Transcript | retained_intron |
| rs2239525 | HSCHR6_MHC_SSTO:31499319-31499319 | C | intron_variant,non_coding_transcript_variant | MODIFIER | *DDX39B* | Transcript | retained_intron |
| rs2239525 | HSCHR6_MHC_SSTO:31499319-31499319 | A | non_coding_transcript_exon_variant | MODIFIER | *DDX39B* | Transcript | retained_intron |
| rs2239525 | HSCHR6_MHC_SSTO:31499319-31499319 | C | non_coding_transcript_exon_variant | MODIFIER | *DDX39B* | Transcript | retained_intron |
| rs2239525 | HSCHR6_MHC_SSTO:31499319-31499319 | A | intron_variant | MODIFIER | *DDX39B* | Transcript | protein_coding |
| rs2239525 | HSCHR6_MHC_SSTO:31499319-31499319 | C | intron_variant | MODIFIER | *DDX39B* | Transcript | protein_coding |
| rs2239525 | HSCHR6_MHC_SSTO:31499319-31499319 | A | intron_variant | MODIFIER | *DDX39B* | Transcript | protein_coding |
| rs2239525 | HSCHR6_MHC_SSTO:31499319-31499319 | C | intron_variant | MODIFIER | *DDX39B* | Transcript | protein_coding |
| rs2239525 | HSCHR6_MHC_SSTO:31499319-31499319 | A | upstream_gene_variant | MODIFIER | *SNORD83* | Transcript | snoRNA |
| rs2239525 | HSCHR6_MHC_SSTO:31499319-31499319 | C | upstream_gene_variant | MODIFIER | *SNORD83* | Transcript | snoRNA |
| rs244418 | 16:69622762-69622762 | A | intron_variant | MODIFIER | *NFAT5* | Transcript | protein_coding |
| rs244418 | 16:69622762-69622762 | A | intron_variant | MODIFIER | *NFAT5* | Transcript | protein_coding |
| rs244418 | 16:69622762-69622762 | A | intron_variant | MODIFIER | *NFAT5* | Transcript | protein_coding |
| rs244418 | 16:69622762-69622762 | A | intron_variant,NMD_transcript_variant | MODIFIER | *NFAT5* | Transcript | nonsense_mediated_decay |
| rs244418 | 16:69622762-69622762 | A | intron_variant | MODIFIER | *NFAT5* | Transcript | protein_coding |
| rs244418 | 16:69622762-69622762 | A | intron_variant | MODIFIER | *NFAT5* | Transcript | protein_coding |
| rs244418 | 16:69622762-69622762 | A | intron_variant | MODIFIER | *NFAT5* | Transcript | protein_coding |
| rs244418 | 16:69622762-69622762 | A | intron_variant,NMD_transcript_variant | MODIFIER | *NFAT5* | Transcript | nonsense_mediated_decay |
| rs244418 | 16:69622762-69622762 | A | regulatory_region_variant | MODIFIER | *-* | RegulatoryFeature | promoter_flanking_region |
| rs2857609 | 6:31577825-31577825 | G | downstream_gene_variant | MODIFIER | *UQCRHP1* | Transcript | processed_pseudogene |
| rs2857609 | 6:31577825-31577825 | T | downstream_gene_variant | MODIFIER | *UQCRHP1* | Transcript | processed_pseudogene |
| rs2857609 | 6:31577825-31577825 | G | regulatory_region_variant | MODIFIER | *-* | RegulatoryFeature | CTCF_binding_site |
| rs2857609 | 6:31577825-31577825 | T | regulatory_region_variant | MODIFIER | *-* | RegulatoryFeature | CTCF_binding_site |
| rs2857609 | 6:31577825-31577825 | G | regulatory_region_variant | MODIFIER | *-* | RegulatoryFeature | enhancer |
| rs2857609 | 6:31577825-31577825 | T | regulatory_region_variant | MODIFIER | *-* | RegulatoryFeature | enhancer |
| rs2857609 | HSCHR6_MHC_APD:31589176-31589176 | A | downstream_gene_variant | MODIFIER | *UQCRHP1* | Transcript | processed_pseudogene |
| rs2857609 | HSCHR6_MHC_APD:31589176-31589176 | T | downstream_gene_variant | MODIFIER | *UQCRHP1* | Transcript | processed_pseudogene |
| rs2857609 | HSCHR6_MHC_COX:31565239-31565239 | A | downstream_gene_variant | MODIFIER | *UQCRHP1* | Transcript | processed_pseudogene |
| rs2857609 | HSCHR6_MHC_COX:31565239-31565239 | T | downstream_gene_variant | MODIFIER | *UQCRHP1* | Transcript | processed_pseudogene |
| rs2857609 | HSCHR6_MHC_DBB:31560007-31560007 | A | downstream_gene_variant | MODIFIER | *UQCRHP1* | Transcript | processed_pseudogene |
| rs2857609 | HSCHR6_MHC_DBB:31560007-31560007 | T | downstream_gene_variant | MODIFIER | *UQCRHP1* | Transcript | processed_pseudogene |
| rs2857609 | HSCHR6_MHC_MANN:31617313-31617313 | A | intergenic_variant | MODIFIER | *-* | - | - |
| rs2857609 | HSCHR6_MHC_MANN:31617313-31617313 | T | intergenic_variant | MODIFIER | *-* | - | - |
| rs2857609 | HSCHR6_MHC_QBL:31568070-31568070 | A | downstream_gene_variant | MODIFIER | *UQCRHP1* | Transcript | processed_pseudogene |
| rs2857609 | HSCHR6_MHC_QBL:31568070-31568070 | T | downstream_gene_variant | MODIFIER | *UQCRHP1* | Transcript | processed_pseudogene |
| rs2857609 | HSCHR6_MHC_SSTO:31567775-31567775 | A | downstream_gene_variant | MODIFIER | *UQCRHP1* | Transcript | processed_pseudogene |
| rs2857609 | HSCHR6_MHC_SSTO:31567775-31567775 | T | downstream_gene_variant | MODIFIER | *UQCRHP1* | Transcript | processed_pseudogene |
| rs2857609 | HSCHR6_MHC_SSTO:31567775-31567775 | A | intron_variant | MODIFIER | *BX511262.2* | Transcript | protein_coding |
| rs2857609 | HSCHR6_MHC_SSTO:31567775-31567775 | T | intron_variant | MODIFIER | *BX511262.2* | Transcript | protein_coding |
| rs3130279 | 6:32112626-32112626 | C | downstream_gene_variant | MODIFIER | *PRRT1* | Transcript | protein_coding |
| rs3130279 | 6:32112626-32112626 | G | downstream_gene_variant | MODIFIER | *PRRT1* | Transcript | protein_coding |
| rs3130279 | 6:32112626-32112626 | C | downstream_gene_variant | MODIFIER | *PRRT1* | Transcript | protein_coding |
| rs3130279 | 6:32112626-32112626 | G | downstream_gene_variant | MODIFIER | *PRRT1* | Transcript | protein_coding |
| rs3130279 | 6:32112626-32112626 | C | downstream_gene_variant | MODIFIER | *PRRT1* | Transcript | protein_coding |
| rs3130279 | 6:32112626-32112626 | G | downstream_gene_variant | MODIFIER | *PRRT1* | Transcript | protein_coding |
| rs3130279 | 6:32112626-32112626 | C | downstream_gene_variant | MODIFIER | *PRRT1* | Transcript | processed_transcript |
| rs3130279 | 6:32112626-32112626 | G | downstream_gene_variant | MODIFIER | *PRRT1* | Transcript | processed_transcript |
| rs3130279 | 6:32112626-32112626 | C | downstream_gene_variant | MODIFIER | *PRRT1* | Transcript | processed_transcript |
| rs3130279 | 6:32112626-32112626 | G | downstream_gene_variant | MODIFIER | *PRRT1* | Transcript | processed_transcript |
| rs3130279 | 6:32112626-32112626 | C | downstream_gene_variant | MODIFIER | *PRRT1* | Transcript | retained_intron |
| rs3130279 | 6:32112626-32112626 | G | downstream_gene_variant | MODIFIER | *PRRT1* | Transcript | retained_intron |
| rs3130279 | HSCHR6_MHC_APD:32123966-32123966 | A | downstream_gene_variant | MODIFIER | *PRRT1* | Transcript | protein_coding |
| rs3130279 | HSCHR6_MHC_APD:32123966-32123966 | C | downstream_gene_variant | MODIFIER | *PRRT1* | Transcript | protein_coding |
| rs3130279 | HSCHR6_MHC_APD:32123966-32123966 | A | downstream_gene_variant | MODIFIER | *PRRT1* | Transcript | protein_coding |
| rs3130279 | HSCHR6_MHC_APD:32123966-32123966 | C | downstream_gene_variant | MODIFIER | *PRRT1* | Transcript | protein_coding |
| rs3130279 | HSCHR6_MHC_APD:32123966-32123966 | A | downstream_gene_variant | MODIFIER | *PRRT1* | Transcript | retained_intron |
| rs3130279 | HSCHR6_MHC_APD:32123966-32123966 | C | downstream_gene_variant | MODIFIER | *PRRT1* | Transcript | retained_intron |
| rs3130279 | HSCHR6_MHC_APD:32123966-32123966 | A | downstream_gene_variant | MODIFIER | *PRRT1* | Transcript | processed_transcript |
| rs3130279 | HSCHR6_MHC_APD:32123966-32123966 | C | downstream_gene_variant | MODIFIER | *PRRT1* | Transcript | processed_transcript |
| rs3130279 | HSCHR6_MHC_APD:32123966-32123966 | A | downstream_gene_variant | MODIFIER | *PRRT1* | Transcript | processed_transcript |
| rs3130279 | HSCHR6_MHC_APD:32123966-32123966 | C | downstream_gene_variant | MODIFIER | *PRRT1* | Transcript | processed_transcript |
| rs3130279 | HSCHR6_MHC_APD:32123966-32123966 | A | downstream_gene_variant | MODIFIER | *PRRT1* | Transcript | protein_coding |
| rs3130279 | HSCHR6_MHC_APD:32123966-32123966 | C | downstream_gene_variant | MODIFIER | *PRRT1* | Transcript | protein_coding |
| rs3130279 | HSCHR6_MHC_COX:32061085-32061085 | A | downstream_gene_variant | MODIFIER | *PRRT1* | Transcript | protein_coding |
| rs3130279 | HSCHR6_MHC_COX:32061085-32061085 | C | downstream_gene_variant | MODIFIER | *PRRT1* | Transcript | protein_coding |
| rs3130279 | HSCHR6_MHC_COX:32061085-32061085 | A | downstream_gene_variant | MODIFIER | *PRRT1* | Transcript | protein_coding |
| rs3130279 | HSCHR6_MHC_COX:32061085-32061085 | C | downstream_gene_variant | MODIFIER | *PRRT1* | Transcript | protein_coding |
| rs3130279 | HSCHR6_MHC_COX:32061085-32061085 | A | downstream_gene_variant | MODIFIER | *PRRT1* | Transcript | retained_intron |
| rs3130279 | HSCHR6_MHC_COX:32061085-32061085 | C | downstream_gene_variant | MODIFIER | *PRRT1* | Transcript | retained_intron |
| rs3130279 | HSCHR6_MHC_COX:32061085-32061085 | A | downstream_gene_variant | MODIFIER | *PRRT1* | Transcript | processed_transcript |
| rs3130279 | HSCHR6_MHC_COX:32061085-32061085 | C | downstream_gene_variant | MODIFIER | *PRRT1* | Transcript | processed_transcript |
| rs3130279 | HSCHR6_MHC_COX:32061085-32061085 | A | downstream_gene_variant | MODIFIER | *PRRT1* | Transcript | processed_transcript |
| rs3130279 | HSCHR6_MHC_COX:32061085-32061085 | C | downstream_gene_variant | MODIFIER | *PRRT1* | Transcript | processed_transcript |
| rs3130279 | HSCHR6_MHC_COX:32061085-32061085 | A | downstream_gene_variant | MODIFIER | *PRRT1* | Transcript | protein_coding |
| rs3130279 | HSCHR6_MHC_COX:32061085-32061085 | C | downstream_gene_variant | MODIFIER | *PRRT1* | Transcript | protein_coding |
| rs3130279 | HSCHR6_MHC_DBB:32088397-32088397 | A | downstream_gene_variant | MODIFIER | *PRRT1* | Transcript | protein_coding |
| rs3130279 | HSCHR6_MHC_DBB:32088397-32088397 | C | downstream_gene_variant | MODIFIER | *PRRT1* | Transcript | protein_coding |
| rs3130279 | HSCHR6_MHC_DBB:32088397-32088397 | A | downstream_gene_variant | MODIFIER | *PRRT1* | Transcript | protein_coding |
| rs3130279 | HSCHR6_MHC_DBB:32088397-32088397 | C | downstream_gene_variant | MODIFIER | *PRRT1* | Transcript | protein_coding |
| rs3130279 | HSCHR6_MHC_DBB:32088397-32088397 | A | downstream_gene_variant | MODIFIER | *PRRT1* | Transcript | processed_transcript |
| rs3130279 | HSCHR6_MHC_DBB:32088397-32088397 | C | downstream_gene_variant | MODIFIER | *PRRT1* | Transcript | processed_transcript |
| rs3130279 | HSCHR6_MHC_DBB:32088397-32088397 | A | downstream_gene_variant | MODIFIER | *PRRT1* | Transcript | retained_intron |
| rs3130279 | HSCHR6_MHC_DBB:32088397-32088397 | C | downstream_gene_variant | MODIFIER | *PRRT1* | Transcript | retained_intron |
| rs3130279 | HSCHR6_MHC_DBB:32088397-32088397 | A | downstream_gene_variant | MODIFIER | *PRRT1* | Transcript | processed_transcript |
| rs3130279 | HSCHR6_MHC_DBB:32088397-32088397 | C | downstream_gene_variant | MODIFIER | *PRRT1* | Transcript | processed_transcript |
| rs3130279 | HSCHR6_MHC_DBB:32088397-32088397 | A | downstream_gene_variant | MODIFIER | *PRRT1* | Transcript | protein_coding |
| rs3130279 | HSCHR6_MHC_DBB:32088397-32088397 | C | downstream_gene_variant | MODIFIER | *PRRT1* | Transcript | protein_coding |
| rs3130279 | HSCHR6_MHC_MANN:32152072-32152072 | A | downstream_gene_variant | MODIFIER | *PRRT1* | Transcript | protein_coding |
| rs3130279 | HSCHR6_MHC_MANN:32152072-32152072 | C | downstream_gene_variant | MODIFIER | *PRRT1* | Transcript | protein_coding |
| rs3130279 | HSCHR6_MHC_MANN:32152072-32152072 | A | downstream_gene_variant | MODIFIER | *PRRT1* | Transcript | protein_coding |
| rs3130279 | HSCHR6_MHC_MANN:32152072-32152072 | C | downstream_gene_variant | MODIFIER | *PRRT1* | Transcript | protein_coding |
| rs3130279 | HSCHR6_MHC_MANN:32152072-32152072 | A | downstream_gene_variant | MODIFIER | *PRRT1* | Transcript | processed_transcript |
| rs3130279 | HSCHR6_MHC_MANN:32152072-32152072 | C | downstream_gene_variant | MODIFIER | *PRRT1* | Transcript | processed_transcript |
| rs3130279 | HSCHR6_MHC_MANN:32152072-32152072 | A | downstream_gene_variant | MODIFIER | *PRRT1* | Transcript | retained_intron |
| rs3130279 | HSCHR6_MHC_MANN:32152072-32152072 | C | downstream_gene_variant | MODIFIER | *PRRT1* | Transcript | retained_intron |
| rs3130279 | HSCHR6_MHC_MANN:32152072-32152072 | A | downstream_gene_variant | MODIFIER | *PRRT1* | Transcript | processed_transcript |
| rs3130279 | HSCHR6_MHC_MANN:32152072-32152072 | C | downstream_gene_variant | MODIFIER | *PRRT1* | Transcript | processed_transcript |
| rs3130279 | HSCHR6_MHC_MANN:32152072-32152072 | A | downstream_gene_variant | MODIFIER | *PRRT1* | Transcript | protein_coding |
| rs3130279 | HSCHR6_MHC_MANN:32152072-32152072 | C | downstream_gene_variant | MODIFIER | *PRRT1* | Transcript | protein_coding |
| rs3130279 | HSCHR6_MHC_MCF:32189052-32189052 | A | downstream_gene_variant | MODIFIER | *PRRT1* | Transcript | protein_coding |
| rs3130279 | HSCHR6_MHC_MCF:32189052-32189052 | C | downstream_gene_variant | MODIFIER | *PRRT1* | Transcript | protein_coding |
| rs3130279 | HSCHR6_MHC_MCF:32189052-32189052 | A | downstream_gene_variant | MODIFIER | *PRRT1* | Transcript | protein_coding |
| rs3130279 | HSCHR6_MHC_MCF:32189052-32189052 | C | downstream_gene_variant | MODIFIER | *PRRT1* | Transcript | protein_coding |
| rs3130279 | HSCHR6_MHC_MCF:32189052-32189052 | A | downstream_gene_variant | MODIFIER | *PRRT1* | Transcript | processed_transcript |
| rs3130279 | HSCHR6_MHC_MCF:32189052-32189052 | C | downstream_gene_variant | MODIFIER | *PRRT1* | Transcript | processed_transcript |
| rs3130279 | HSCHR6_MHC_MCF:32189052-32189052 | A | downstream_gene_variant | MODIFIER | *PRRT1* | Transcript | retained_intron |
| rs3130279 | HSCHR6_MHC_MCF:32189052-32189052 | C | downstream_gene_variant | MODIFIER | *PRRT1* | Transcript | retained_intron |
| rs3130279 | HSCHR6_MHC_MCF:32189052-32189052 | A | downstream_gene_variant | MODIFIER | *PRRT1* | Transcript | processed_transcript |
| rs3130279 | HSCHR6_MHC_MCF:32189052-32189052 | C | downstream_gene_variant | MODIFIER | *PRRT1* | Transcript | processed_transcript |
| rs3130279 | HSCHR6_MHC_MCF:32189052-32189052 | A | downstream_gene_variant | MODIFIER | *PRRT1* | Transcript | protein_coding |
| rs3130279 | HSCHR6_MHC_MCF:32189052-32189052 | C | downstream_gene_variant | MODIFIER | *PRRT1* | Transcript | protein_coding |
| rs3130279 | HSCHR6_MHC_QBL:32070244-32070244 | A | downstream_gene_variant | MODIFIER | *PRRT1* | Transcript | protein_coding |
| rs3130279 | HSCHR6_MHC_QBL:32070244-32070244 | C | downstream_gene_variant | MODIFIER | *PRRT1* | Transcript | protein_coding |
| rs3130279 | HSCHR6_MHC_QBL:32070244-32070244 | A | downstream_gene_variant | MODIFIER | *PRRT1* | Transcript | protein_coding |
| rs3130279 | HSCHR6_MHC_QBL:32070244-32070244 | C | downstream_gene_variant | MODIFIER | *PRRT1* | Transcript | protein_coding |
| rs3130279 | HSCHR6_MHC_QBL:32070244-32070244 | A | downstream_gene_variant | MODIFIER | *PRRT1* | Transcript | retained_intron |
| rs3130279 | HSCHR6_MHC_QBL:32070244-32070244 | C | downstream_gene_variant | MODIFIER | *PRRT1* | Transcript | retained_intron |
| rs3130279 | HSCHR6_MHC_QBL:32070244-32070244 | A | downstream_gene_variant | MODIFIER | *PRRT1* | Transcript | processed_transcript |
| rs3130279 | HSCHR6_MHC_QBL:32070244-32070244 | C | downstream_gene_variant | MODIFIER | *PRRT1* | Transcript | processed_transcript |
| rs3130279 | HSCHR6_MHC_QBL:32070244-32070244 | A | downstream_gene_variant | MODIFIER | *PRRT1* | Transcript | processed_transcript |
| rs3130279 | HSCHR6_MHC_QBL:32070244-32070244 | C | downstream_gene_variant | MODIFIER | *PRRT1* | Transcript | processed_transcript |
| rs3130279 | HSCHR6_MHC_QBL:32070244-32070244 | A | downstream_gene_variant | MODIFIER | *PRRT1* | Transcript | protein_coding |
| rs3130279 | HSCHR6_MHC_QBL:32070244-32070244 | C | downstream_gene_variant | MODIFIER | *PRRT1* | Transcript | protein_coding |
| rs3130279 | HSCHR6_MHC_SSTO:32119493-32119493 | A | downstream_gene_variant | MODIFIER | *PRRT1* | Transcript | protein_coding |
| rs3130279 | HSCHR6_MHC_SSTO:32119493-32119493 | C | downstream_gene_variant | MODIFIER | *PRRT1* | Transcript | protein_coding |
| rs3130279 | HSCHR6_MHC_SSTO:32119493-32119493 | A | downstream_gene_variant | MODIFIER | *PRRT1* | Transcript | protein_coding |
| rs3130279 | HSCHR6_MHC_SSTO:32119493-32119493 | C | downstream_gene_variant | MODIFIER | *PRRT1* | Transcript | protein_coding |
| rs3130279 | HSCHR6_MHC_SSTO:32119493-32119493 | A | downstream_gene_variant | MODIFIER | *PRRT1* | Transcript | processed_transcript |
| rs3130279 | HSCHR6_MHC_SSTO:32119493-32119493 | C | downstream_gene_variant | MODIFIER | *PRRT1* | Transcript | processed_transcript |
| rs3130279 | HSCHR6_MHC_SSTO:32119493-32119493 | A | downstream_gene_variant | MODIFIER | *PRRT1* | Transcript | processed_transcript |
| rs3130279 | HSCHR6_MHC_SSTO:32119493-32119493 | C | downstream_gene_variant | MODIFIER | *PRRT1* | Transcript | processed_transcript |
| rs3130279 | HSCHR6_MHC_SSTO:32119493-32119493 | A | downstream_gene_variant | MODIFIER | *PRRT1* | Transcript | retained_intron |
| rs3130279 | HSCHR6_MHC_SSTO:32119493-32119493 | C | downstream_gene_variant | MODIFIER | *PRRT1* | Transcript | retained_intron |
| rs3130279 | HSCHR6_MHC_SSTO:32119493-32119493 | A | downstream_gene_variant | MODIFIER | *PRRT1* | Transcript | protein_coding |
| rs3130279 | HSCHR6_MHC_SSTO:32119493-32119493 | C | downstream_gene_variant | MODIFIER | *PRRT1* | Transcript | protein_coding |
| rs519790 | 11:72504141-72504141 | G | 5_prime_UTR_variant | MODIFIER | *STARD10* | Transcript | protein_coding |
| rs519790 | 11:72504141-72504141 | T | 5_prime_UTR_variant | MODIFIER | *STARD10* | Transcript | protein_coding |
| rs519790 | 11:72504141-72504141 | G | 5_prime_UTR_variant | MODIFIER | *ARAP1* | Transcript | protein_coding |
| rs519790 | 11:72504141-72504141 | T | 5_prime_UTR_variant | MODIFIER | *ARAP1* | Transcript | protein_coding |
| rs519790 | 11:72504141-72504141 | G | intron_variant | MODIFIER | *STARD10* | Transcript | protein_coding |
| rs519790 | 11:72504141-72504141 | T | intron_variant | MODIFIER | *STARD10* | Transcript | protein_coding |
| rs519790 | 11:72504141-72504141 | G | intron_variant,non_coding_transcript_variant | MODIFIER | *STARD10* | Transcript | retained_intron |
| rs519790 | 11:72504141-72504141 | T | intron_variant,non_coding_transcript_variant | MODIFIER | *STARD10* | Transcript | retained_intron |
| rs519790 | 11:72504141-72504141 | G | 5_prime_UTR_variant | MODIFIER | *STARD10* | Transcript | protein_coding |
| rs519790 | 11:72504141-72504141 | T | 5_prime_UTR_variant | MODIFIER | *STARD10* | Transcript | protein_coding |
| rs519790 | 11:72504141-72504141 | G | intron_variant | MODIFIER | *STARD10* | Transcript | protein_coding |
| rs519790 | 11:72504141-72504141 | T | intron_variant | MODIFIER | *STARD10* | Transcript | protein_coding |
| rs519790 | 11:72504141-72504141 | G | intron_variant | MODIFIER | *STARD10* | Transcript | protein_coding |
| rs519790 | 11:72504141-72504141 | T | intron_variant | MODIFIER | *STARD10* | Transcript | protein_coding |
| rs519790 | 11:72504141-72504141 | G | regulatory_region_variant | MODIFIER | *-* | RegulatoryFeature | promoter |
| rs519790 | 11:72504141-72504141 | T | regulatory_region_variant | MODIFIER | *-* | RegulatoryFeature | promoter |
| rs56094641 | 16:53806453-53806453 | G | intron_variant | MODIFIER | *FTO* | Transcript | protein_coding |
| rs56094641 | 16:53806453-53806453 | T | intron_variant | MODIFIER | *FTO* | Transcript | protein_coding |
| rs56094641 | 16:53806453-53806453 | G | intron_variant,NMD_transcript_variant | MODIFIER | *FTO* | Transcript | nonsense_mediated_decay |
| rs56094641 | 16:53806453-53806453 | T | intron_variant,NMD_transcript_variant | MODIFIER | *FTO* | Transcript | nonsense_mediated_decay |
| rs56094641 | 16:53806453-53806453 | G | intron_variant | MODIFIER | *FTO* | Transcript | protein_coding |
| rs56094641 | 16:53806453-53806453 | T | intron_variant | MODIFIER | *FTO* | Transcript | protein_coding |
| rs56094641 | 16:53806453-53806453 | G | intron_variant,non_coding_transcript_variant | MODIFIER | *FTO* | Transcript | processed_transcript |
| rs56094641 | 16:53806453-53806453 | T | intron_variant,non_coding_transcript_variant | MODIFIER | *FTO* | Transcript | processed_transcript |
| rs56094641 | 16:53806453-53806453 | G | regulatory_region_variant | MODIFIER | *-* | RegulatoryFeature | promoter_flanking_region |
| rs56094641 | 16:53806453-53806453 | T | regulatory_region_variant | MODIFIER | *-* | RegulatoryFeature | promoter_flanking_region |
| rs58304657 | 19:46176405-46176405 | C | intron_variant | MODIFIER | *GIPR* | Transcript | protein_coding |
| rs58304657 | 19:46176405-46176405 | C | intron_variant | MODIFIER | *GIPR* | Transcript | protein_coding |
| rs58304657 | 19:46176405-46176405 | C | upstream_gene_variant | MODIFIER | *MIR642A* | Transcript | miRNA |
| rs58304657 | 19:46176405-46176405 | C | intron_variant,NMD_transcript_variant | MODIFIER | *GIPR* | Transcript | nonsense_mediated_decay |
| rs58304657 | 19:46176405-46176405 | C | intron_variant,non_coding_transcript_variant | MODIFIER | *GIPR* | Transcript | retained_intron |
| rs58304657 | 19:46176405-46176405 | C | intron_variant | MODIFIER | *GIPR* | Transcript | protein_coding |
| rs58304657 | 19:46176405-46176405 | C | upstream_gene_variant | MODIFIER | *GIPR* | Transcript | retained_intron |
| rs58304657 | 19:46176405-46176405 | C | downstream_gene_variant | MODIFIER | *GIPR* | Transcript | protein_coding |
| rs58304657 | 19:46176405-46176405 | C | upstream_gene_variant | MODIFIER | *GIPR* | Transcript | processed_transcript |
| rs736820 | 20:43034016-43034016 | A | intron_variant | MODIFIER | *HNF4A* | Transcript | protein_coding |
| rs736820 | 20:43034016-43034016 | C | intron_variant | MODIFIER | *HNF4A* | Transcript | protein_coding |
| rs736820 | 20:43034016-43034016 | A | intron_variant | MODIFIER | *HNF4A* | Transcript | protein_coding |
| rs736820 | 20:43034016-43034016 | C | intron_variant | MODIFIER | *HNF4A* | Transcript | protein_coding |
| rs736820 | 20:43034016-43034016 | A | intron_variant,NMD_transcript_variant | MODIFIER | *HNF4A* | Transcript | nonsense_mediated_decay |
| rs736820 | 20:43034016-43034016 | C | intron_variant,NMD_transcript_variant | MODIFIER | *HNF4A* | Transcript | nonsense_mediated_decay |
| rs736820 | 20:43034016-43034016 | A | intron_variant | MODIFIER | *HNF4A* | Transcript | protein_coding |
| rs736820 | 20:43034016-43034016 | C | intron_variant | MODIFIER | *HNF4A* | Transcript | protein_coding |
| rs736820 | 20:43034016-43034016 | A | intron_variant | MODIFIER | *HNF4A* | Transcript | protein_coding |
| rs736820 | 20:43034016-43034016 | C | intron_variant | MODIFIER | *HNF4A* | Transcript | protein_coding |
| rs736820 | 20:43034016-43034016 | A | intron_variant | MODIFIER | *HNF4A* | Transcript | protein_coding |
| rs736820 | 20:43034016-43034016 | C | intron_variant | MODIFIER | *HNF4A* | Transcript | protein_coding |
| rs736820 | 20:43034016-43034016 | A | upstream_gene_variant | MODIFIER | *MIR3646* | Transcript | miRNA |
| rs736820 | 20:43034016-43034016 | C | upstream_gene_variant | MODIFIER | *MIR3646* | Transcript | miRNA |
| rs736820 | 20:43034016-43034016 | A | intron_variant | MODIFIER | *HNF4A* | Transcript | protein_coding |
| rs736820 | 20:43034016-43034016 | C | intron_variant | MODIFIER | *HNF4A* | Transcript | protein_coding |
| rs736820 | 20:43034016-43034016 | A | intron_variant | MODIFIER | *HNF4A* | Transcript | protein_coding |
| rs736820 | 20:43034016-43034016 | C | intron_variant | MODIFIER | *HNF4A* | Transcript | protein_coding |
| rs738408 | 22:44324730-44324730 | T | synonymous_variant | LOW | *PNPLA3* | Transcript | protein_coding |
| rs738408 | 22:44324730-44324730 | T | 3_prime_UTR_variant,NMD_transcript_variant | MODIFIER | *PNPLA3* | Transcript | nonsense_mediated_decay |
| rs738408 | 22:44324730-44324730 | T | synonymous_variant | LOW | *PNPLA3* | Transcript | protein_coding |
| rs738408 | 22:44324730-44324730 | T | non_coding_transcript_exon_variant | MODIFIER | *PNPLA3* | Transcript | processed_transcript |
| rs738408 | 22:44324730-44324730 | T | upstream_gene_variant | MODIFIER | *PNPLA3* | Transcript | processed_transcript |
| rs879882 | 6:31139452-31139452 | C | upstream_gene_variant | MODIFIER | *POU5F1* | Transcript | protein_coding |
| rs879882 | 6:31139452-31139452 | G | upstream_gene_variant | MODIFIER | *POU5F1* | Transcript | protein_coding |
| rs879882 | 6:31139452-31139452 | C | downstream_gene_variant | MODIFIER | *PSORS1C3* | Transcript | sense_intronic |
| rs879882 | 6:31139452-31139452 | G | downstream_gene_variant | MODIFIER | *PSORS1C3* | Transcript | sense_intronic |
| rs879882 | 6:31139452-31139452 | C | intron_variant | MODIFIER | *POU5F1* | Transcript | protein_coding |
| rs879882 | 6:31139452-31139452 | G | intron_variant | MODIFIER | *POU5F1* | Transcript | protein_coding |
| rs879882 | 6:31139452-31139452 | C | upstream_gene_variant | MODIFIER | *POU5F1* | Transcript | retained_intron |
| rs879882 | 6:31139452-31139452 | G | upstream_gene_variant | MODIFIER | *POU5F1* | Transcript | retained_intron |
| rs879882 | 6:31139452-31139452 | C | upstream_gene_variant | MODIFIER | *POU5F1* | Transcript | protein_coding |
| rs879882 | 6:31139452-31139452 | G | upstream_gene_variant | MODIFIER | *POU5F1* | Transcript | protein_coding |
| rs879882 | 6:31139452-31139452 | C | upstream_gene_variant | MODIFIER | *POU5F1* | Transcript | protein_coding |
| rs879882 | 6:31139452-31139452 | G | upstream_gene_variant | MODIFIER | *POU5F1* | Transcript | protein_coding |
| rs879882 | 6:31139452-31139452 | C | upstream_gene_variant | MODIFIER | *POU5F1* | Transcript | protein_coding |
| rs879882 | 6:31139452-31139452 | G | upstream_gene_variant | MODIFIER | *POU5F1* | Transcript | protein_coding |
| rs879882 | 6:31139452-31139452 | C | downstream_gene_variant | MODIFIER | *TCF19* | Transcript | protein_coding |
| rs879882 | 6:31139452-31139452 | G | downstream_gene_variant | MODIFIER | *TCF19* | Transcript | protein_coding |
| rs879882 | 6:31139452-31139452 | C | upstream_gene_variant | MODIFIER | *POU5F1* | Transcript | protein_coding |
| rs879882 | 6:31139452-31139452 | G | upstream_gene_variant | MODIFIER | *POU5F1* | Transcript | protein_coding |
| rs879882 | HSCHR6_MHC_COX:31132018-31132018 | T | upstream_gene_variant | MODIFIER | *POU5F1* | Transcript | protein_coding |
| rs879882 | HSCHR6_MHC_COX:31132018-31132018 | G | upstream_gene_variant | MODIFIER | *POU5F1* | Transcript | protein_coding |
| rs879882 | HSCHR6_MHC_COX:31132018-31132018 | T | downstream_gene_variant | MODIFIER | *PSORS1C3* | Transcript | processed_transcript |
| rs879882 | HSCHR6_MHC_COX:31132018-31132018 | G | downstream_gene_variant | MODIFIER | *PSORS1C3* | Transcript | processed_transcript |
| rs879882 | HSCHR6_MHC_COX:31132018-31132018 | T | upstream_gene_variant | MODIFIER | *POU5F1* | Transcript | retained_intron |
| rs879882 | HSCHR6_MHC_COX:31132018-31132018 | G | upstream_gene_variant | MODIFIER | *POU5F1* | Transcript | retained_intron |
| rs879882 | HSCHR6_MHC_COX:31132018-31132018 | T | upstream_gene_variant | MODIFIER | *POU5F1* | Transcript | protein_coding |
| rs879882 | HSCHR6_MHC_COX:31132018-31132018 | G | upstream_gene_variant | MODIFIER | *POU5F1* | Transcript | protein_coding |
| rs879882 | HSCHR6_MHC_COX:31132018-31132018 | T | upstream_gene_variant | MODIFIER | *POU5F1* | Transcript | protein_coding |
| rs879882 | HSCHR6_MHC_COX:31132018-31132018 | G | upstream_gene_variant | MODIFIER | *POU5F1* | Transcript | protein_coding |
| rs879882 | HSCHR6_MHC_DBB:31133196-31133196 | T | upstream_gene_variant | MODIFIER | *POU5F1* | Transcript | protein_coding |
| rs879882 | HSCHR6_MHC_DBB:31133196-31133196 | G | upstream_gene_variant | MODIFIER | *POU5F1* | Transcript | protein_coding |
| rs879882 | HSCHR6_MHC_DBB:31133196-31133196 | T | downstream_gene_variant | MODIFIER | *PSORS1C3* | Transcript | processed_transcript |
| rs879882 | HSCHR6_MHC_DBB:31133196-31133196 | G | downstream_gene_variant | MODIFIER | *PSORS1C3* | Transcript | processed_transcript |
| rs879882 | HSCHR6_MHC_DBB:31133196-31133196 | T | upstream_gene_variant | MODIFIER | *POU5F1* | Transcript | retained_intron |
| rs879882 | HSCHR6_MHC_DBB:31133196-31133196 | G | upstream_gene_variant | MODIFIER | *POU5F1* | Transcript | retained_intron |
| rs879882 | HSCHR6_MHC_DBB:31133196-31133196 | T | upstream_gene_variant | MODIFIER | *POU5F1* | Transcript | protein_coding |
| rs879882 | HSCHR6_MHC_DBB:31133196-31133196 | G | upstream_gene_variant | MODIFIER | *POU5F1* | Transcript | protein_coding |
| rs879882 | HSCHR6_MHC_DBB:31133196-31133196 | T | upstream_gene_variant | MODIFIER | *POU5F1* | Transcript | protein_coding |
| rs879882 | HSCHR6_MHC_DBB:31133196-31133196 | G | upstream_gene_variant | MODIFIER | *POU5F1* | Transcript | protein_coding |
| rs879882 | HSCHR6_MHC_MANN:31184417-31184417 | T | downstream_gene_variant | MODIFIER | *PSORS1C3* | Transcript | processed_transcript |
| rs879882 | HSCHR6_MHC_MANN:31184417-31184417 | G | downstream_gene_variant | MODIFIER | *PSORS1C3* | Transcript | processed_transcript |
| rs879882 | HSCHR6_MHC_MANN:31184417-31184417 | T | upstream_gene_variant | MODIFIER | *POU5F1* | Transcript | protein_coding |
| rs879882 | HSCHR6_MHC_MANN:31184417-31184417 | G | upstream_gene_variant | MODIFIER | *POU5F1* | Transcript | protein_coding |
| rs879882 | HSCHR6_MHC_MANN:31184417-31184417 | T | upstream_gene_variant | MODIFIER | *POU5F1* | Transcript | retained_intron |
| rs879882 | HSCHR6_MHC_MANN:31184417-31184417 | G | upstream_gene_variant | MODIFIER | *POU5F1* | Transcript | retained_intron |
| rs879882 | HSCHR6_MHC_MANN:31184417-31184417 | T | upstream_gene_variant | MODIFIER | *POU5F1* | Transcript | protein_coding |
| rs879882 | HSCHR6_MHC_MANN:31184417-31184417 | G | upstream_gene_variant | MODIFIER | *POU5F1* | Transcript | protein_coding |
| rs879882 | HSCHR6_MHC_MANN:31184417-31184417 | T | upstream_gene_variant | MODIFIER | *POU5F1* | Transcript | protein_coding |
| rs879882 | HSCHR6_MHC_MANN:31184417-31184417 | G | upstream_gene_variant | MODIFIER | *POU5F1* | Transcript | protein_coding |
| rs879882 | HSCHR6_MHC_MANN:31184417-31184417 | T | intron_variant | MODIFIER | *CR847794.1* | Transcript | protein_coding |
| rs879882 | HSCHR6_MHC_MANN:31184417-31184417 | G | intron_variant | MODIFIER | *CR847794.1* | Transcript | protein_coding |
| rs879882 | HSCHR6_MHC_MCF:31217996-31217996 | T | downstream_gene_variant | MODIFIER | *PSORS1C3* | Transcript | processed_transcript |
| rs879882 | HSCHR6_MHC_MCF:31217996-31217996 | G | downstream_gene_variant | MODIFIER | *PSORS1C3* | Transcript | processed_transcript |
| rs879882 | HSCHR6_MHC_MCF:31217996-31217996 | T | upstream_gene_variant | MODIFIER | *POU5F1* | Transcript | protein_coding |
| rs879882 | HSCHR6_MHC_MCF:31217996-31217996 | G | upstream_gene_variant | MODIFIER | *POU5F1* | Transcript | protein_coding |
| rs879882 | HSCHR6_MHC_MCF:31217996-31217996 | T | upstream_gene_variant | MODIFIER | *POU5F1* | Transcript | retained_intron |
| rs879882 | HSCHR6_MHC_MCF:31217996-31217996 | G | upstream_gene_variant | MODIFIER | *POU5F1* | Transcript | retained_intron |
| rs879882 | HSCHR6_MHC_MCF:31217996-31217996 | T | intron_variant | MODIFIER | *CR759815.2* | Transcript | protein_coding |
| rs879882 | HSCHR6_MHC_MCF:31217996-31217996 | G | intron_variant | MODIFIER | *CR759815.2* | Transcript | protein_coding |
| rs879882 | HSCHR6_MHC_MCF:31217996-31217996 | T | upstream_gene_variant | MODIFIER | *POU5F1* | Transcript | protein_coding |
| rs879882 | HSCHR6_MHC_MCF:31217996-31217996 | G | upstream_gene_variant | MODIFIER | *POU5F1* | Transcript | protein_coding |
| rs879882 | HSCHR6_MHC_MCF:31217996-31217996 | T | upstream_gene_variant | MODIFIER | *POU5F1* | Transcript | protein_coding |
| rs879882 | HSCHR6_MHC_MCF:31217996-31217996 | G | upstream_gene_variant | MODIFIER | *POU5F1* | Transcript | protein_coding |
| rs879882 | HSCHR6_MHC_QBL:31131924-31131924 | C | upstream_gene_variant | MODIFIER | *POU5F1* | Transcript | protein_coding |
| rs879882 | HSCHR6_MHC_QBL:31131924-31131924 | G | upstream_gene_variant | MODIFIER | *POU5F1* | Transcript | protein_coding |
| rs879882 | HSCHR6_MHC_QBL:31131924-31131924 | C | downstream_gene_variant | MODIFIER | *PSORS1C3* | Transcript | processed_transcript |
| rs879882 | HSCHR6_MHC_QBL:31131924-31131924 | G | downstream_gene_variant | MODIFIER | *PSORS1C3* | Transcript | processed_transcript |
| rs879882 | HSCHR6_MHC_QBL:31131924-31131924 | C | upstream_gene_variant | MODIFIER | *POU5F1* | Transcript | retained_intron |
| rs879882 | HSCHR6_MHC_QBL:31131924-31131924 | G | upstream_gene_variant | MODIFIER | *POU5F1* | Transcript | retained_intron |
| rs879882 | HSCHR6_MHC_QBL:31131924-31131924 | C | upstream_gene_variant | MODIFIER | *POU5F1* | Transcript | protein_coding |
| rs879882 | HSCHR6_MHC_QBL:31131924-31131924 | G | upstream_gene_variant | MODIFIER | *POU5F1* | Transcript | protein_coding |
| rs879882 | HSCHR6_MHC_QBL:31131924-31131924 | C | upstream_gene_variant | MODIFIER | *POU5F1* | Transcript | protein_coding |
| rs879882 | HSCHR6_MHC_QBL:31131924-31131924 | G | upstream_gene_variant | MODIFIER | *POU5F1* | Transcript | protein_coding |
| rs879882 | HSCHR6_MHC_SSTO:31133546-31133546 | T | downstream_gene_variant | MODIFIER | *PSORS1C3* | Transcript | processed_transcript |
| rs879882 | HSCHR6_MHC_SSTO:31133546-31133546 | G | downstream_gene_variant | MODIFIER | *PSORS1C3* | Transcript | processed_transcript |
| rs879882 | HSCHR6_MHC_SSTO:31133546-31133546 | T | upstream_gene_variant | MODIFIER | *POU5F1* | Transcript | protein_coding |
| rs879882 | HSCHR6_MHC_SSTO:31133546-31133546 | G | upstream_gene_variant | MODIFIER | *POU5F1* | Transcript | protein_coding |
| rs879882 | HSCHR6_MHC_SSTO:31133546-31133546 | T | upstream_gene_variant | MODIFIER | *POU5F1* | Transcript | retained_intron |
| rs879882 | HSCHR6_MHC_SSTO:31133546-31133546 | G | upstream_gene_variant | MODIFIER | *POU5F1* | Transcript | retained_intron |
| rs879882 | HSCHR6_MHC_SSTO:31133546-31133546 | T | upstream_gene_variant | MODIFIER | *POU5F1* | Transcript | protein_coding |
| rs879882 | HSCHR6_MHC_SSTO:31133546-31133546 | G | upstream_gene_variant | MODIFIER | *POU5F1* | Transcript | protein_coding |
| rs879882 | HSCHR6_MHC_SSTO:31133546-31133546 | T | upstream_gene_variant | MODIFIER | *POU5F1* | Transcript | protein_coding |
| rs879882 | HSCHR6_MHC_SSTO:31133546-31133546 | G | upstream_gene_variant | MODIFIER | *POU5F1* | Transcript | protein_coding |

CPASSOC, Cross-Phenotype Association; SNPs, single nucleotide Polymorphisms.

## Table S12. List of SNPs in the 99% credible set identified from fine-mapping analysis for each CPASSOC-identified locus shared between type 2 diabetes mellitus and gallstone disease.

| **Index SNPs** | **Credible-set SNPs** | **CHR** | **POS** | ***P*-CPASSOC** | **PIP** | **CumSum** |
| --- | --- | --- | --- | --- | --- | --- |
| rs10882889 | rs10748694 | 10 | 99056190 | 1.36E-11 | 0.0610 | 0.0610 |
|  | rs10882889 | 10 | 99045890 | 1.36E-11 | 0.0610 | 0.1220 |
|  | rs10786324 | 10 | 99054387 | 1.73E-11 | 0.0480 | 0.1700 |
|  | rs10882883 | 10 | 99013312 | 2.10E-11 | 0.0397 | 0.2097 |
|  | rs10736116 | 10 | 99056921 | 2.14E-11 | 0.0390 | 0.2487 |
|  | rs10882884 | 10 | 99025611 | 2.24E-11 | 0.0373 | 0.2860 |
|  | rs953097 | 10 | 98981914 | 2.43E-11 | 0.0344 | 0.3204 |
|  | rs12221430 | 10 | 99061386 | 2.43E-11 | 0.0344 | 0.3548 |
|  | rs11189037 | 10 | 98974707 | 2.95E-11 | 0.0285 | 0.3832 |
|  | rs10882891 | 10 | 99059645 | 3.13E-11 | 0.0269 | 0.4101 |
|  | rs10882880 | 10 | 99008068 | 3.13E-11 | 0.0269 | 0.4370 |
|  | rs2065861 | 10 | 99004608 | 3.13E-11 | 0.0269 | 0.4639 |
|  | rs2861875 | 10 | 98990139 | 3.28E-11 | 0.0257 | 0.4895 |
|  | rs11189138 | 10 | 99099397 | 3.29E-11 | 0.0256 | 0.5152 |
|  | rs10748691 | 10 | 98983955 | 3.44E-11 | 0.0245 | 0.5397 |
|  | rs4917762 | 10 | 98976471 | 4.36E-11 | 0.0194 | 0.5591 |
|  | rs12357266 | 10 | 98978045 | 4.36E-11 | 0.0194 | 0.5785 |
|  | rs11189038 | 10 | 98974765 | 5.09E-11 | 0.0167 | 0.5953 |
|  | rs3814164 | 10 | 98982749 | 5.13E-11 | 0.0166 | 0.6118 |
|  | rs3740522 | 10 | 99019144 | 5.92E-11 | 0.0144 | 0.6263 |
|  | rs7067835 | 10 | 99074649 | 6.12E-11 | 0.0139 | 0.6402 |
|  | rs10882895 | 10 | 99075300 | 6.42E-11 | 0.0133 | 0.6535 |
|  | rs10786325 | 10 | 99068738 | 6.73E-11 | 0.0127 | 0.6662 |
|  | rs10786319 | 10 | 99008951 | 6.73E-11 | 0.0127 | 0.6789 |
|  | rs7072078 | 10 | 99026509 | 6.81E-11 | 0.0126 | 0.6915 |
|  | rs4919087 | 10 | 99072507 | 7.06E-11 | 0.0121 | 0.7036 |
|  | rs61863767 | 10 | 99084426 | 7.83E-11 | 0.0110 | 0.7146 |
|  | rs945187 | 10 | 99091369 | 7.83E-11 | 0.0110 | 0.7255 |
|  | rs10786333 | 10 | 99090390 | 8.15E-11 | 0.0105 | 0.7361 |
|  | rs11189127 | 10 | 99079023 | 8.21E-11 | 0.0105 | 0.7466 |
|  | rs10786329 | 10 | 99074621 | 8.21E-11 | 0.0105 | 0.7570 |
|  | rs10786330 | 10 | 99074959 | 8.21E-11 | 0.0105 | 0.7675 |
|  | rs10882897 | 10 | 99086917 | 8.54E-11 | 0.0101 | 0.7776 |
|  | rs7080275 | 10 | 99089538 | 8.95E-11 | 0.0096 | 0.7872 |
|  | rs4919090 | 10 | 99088419 | 9.39E-11 | 0.0092 | 0.7964 |
|  | rs7076523 | 10 | 99089296 | 9.39E-11 | 0.0092 | 0.8056 |
|  | rs12220695 | 10 | 98993813 | 9.43E-11 | 0.0091 | 0.8147 |
|  | rs10882881 | 10 | 99010067 | 9.43E-11 | 0.0091 | 0.8238 |
|  | rs10882894 | 10 | 99074100 | 9.43E-11 | 0.0091 | 0.8330 |
|  | rs10786327 | 10 | 99071408 | 9.43E-11 | 0.0091 | 0.8421 |
|  | rs11189120 | 10 | 99069493 | 9.43E-11 | 0.0091 | 0.8513 |
|  | rs11189122 | 10 | 99072595 | 9.88E-11 | 0.0087 | 0.8600 |
|  | rs11189100 | 10 | 99047712 | 1.04E-10 | 0.0083 | 0.8683 |
|  | rs4919089 | 10 | 99087905 | 1.20E-10 | 0.0072 | 0.8755 |
|  | rs10882898 | 10 | 99087330 | 1.20E-10 | 0.0072 | 0.8828 |
|  | rs7078288 | 10 | 99086361 | 1.25E-10 | 0.0069 | 0.8897 |
|  | rs11189132 | 10 | 99086195 | 1.25E-10 | 0.0069 | 0.8966 |
|  | rs10786332 | 10 | 99088181 | 1.25E-10 | 0.0069 | 0.9035 |
|  | rs4919091 | 10 | 99088682 | 1.31E-10 | 0.0066 | 0.9101 |
|  | rs4917766 | 10 | 99097191 | 1.33E-10 | 0.0065 | 0.9167 |
|  | rs12413688 | 10 | 99095544 | 1.38E-10 | 0.0063 | 0.9230 |
|  | rs12571098 | 10 | 99095208 | 1.38E-10 | 0.0063 | 0.9293 |
|  | rs11189137 | 10 | 99095687 | 1.44E-10 | 0.0060 | 0.9353 |
|  | rs7091162 | 10 | 99071242 | 1.71E-10 | 0.0051 | 0.9404 |
|  | rs10882899 | 10 | 99095945 | 1.84E-10 | 0.0048 | 0.9452 |
|  | rs2297987 | 10 | 98989454 | 1.87E-10 | 0.0047 | 0.9498 |
|  | rs10786335 | 10 | 99098702 | 1.93E-10 | 0.0046 | 0.9544 |
|  | rs10786334 | 10 | 99098537 | 2.02E-10 | 0.0044 | 0.9588 |
|  | rs701817 | 10 | 98977329 | 2.58E-10 | 0.0034 | 0.9622 |
|  | rs10786326 | 10 | 99070377 | 2.84E-10 | 0.0031 | 0.9653 |
|  | rs1147600 | 10 | 98981643 | 4.19E-10 | 0.0021 | 0.9674 |
|  | rs793517 | 10 | 98987522 | 4.19E-10 | 0.0021 | 0.9695 |
|  | rs1468069 | 10 | 98988759 | 4.41E-10 | 0.0020 | 0.9716 |
|  | rs701815 | 10 | 98975893 | 4.57E-10 | 0.0020 | 0.9735 |
|  | rs701812 | 10 | 98975413 | 4.77E-10 | 0.0019 | 0.9754 |
|  | rs701819 | 10 | 98986316 | 5.47E-10 | 0.0016 | 0.9770 |
|  | rs701814 | 10 | 98975719 | 5.95E-10 | 0.0015 | 0.9786 |
|  | rs701813 | 10 | 98975632 | 5.95E-10 | 0.0015 | 0.9801 |
|  | rs793516 | 10 | 98987985 | 6.76E-10 | 0.0013 | 0.9814 |
|  | rs11189121 | 10 | 99072036 | 6.79E-10 | 0.0013 | 0.9827 |
|  | rs12769073 | 10 | 99073463 | 7.39E-10 | 0.0012 | 0.9840 |
|  | rs12240590 | 10 | 99072066 | 9.30E-10 | 0.0010 | 0.9849 |
|  | rs701810 | 10 | 98974849 | 9.45E-10 | 0.0010 | 0.9859 |
|  | rs701816 | 10 | 98977250 | 9.45E-10 | 0.0010 | 0.9869 |
|  | rs61861849 | 10 | 99073083 | 9.69E-10 | 0.0009 | 0.9878 |
|  | rs111923823 | 10 | 99073255 | 9.69E-10 | 0.0009 | 0.9887 |
|  | rs1687369 | 10 | 98976923 | 9.86E-10 | 0.0009 | 0.9897 |
|  | rs10736115 | 10 | 99031685 | 1.06E-09 | 0.0009 | 0.9905 |
| rs11075985 | rs9931494 | 16 | 53827179 | 3.35E-83 | 0.0159 | 0.0159 |
|  | rs1558902 | 16 | 53803574 | 3.35E-83 | 0.0159 | 0.0317 |
|  | rs8063057 | 16 | 53812433 | 3.35E-83 | 0.0159 | 0.0476 |
|  | rs62033400 | 16 | 53811788 | 3.35E-83 | 0.0159 | 0.0635 |
|  | rs62033408 | 16 | 53827962 | 3.35E-83 | 0.0159 | 0.0794 |
|  | rs17817497 | 16 | 53815435 | 3.35E-83 | 0.0159 | 0.0952 |
|  | rs7202296 | 16 | 53821690 | 3.35E-83 | 0.0159 | 0.1111 |
|  | rs55872725 | 16 | 53809123 | 3.35E-83 | 0.0159 | 0.1270 |
|  | rs9941349 | 16 | 53825488 | 3.35E-83 | 0.0159 | 0.1429 |
|  | rs10468280 | 16 | 53827479 | 3.35E-83 | 0.0159 | 0.1587 |
|  | rs9940128 | 16 | 53800754 | 3.35E-83 | 0.0159 | 0.1746 |
|  | rs3751814 | 16 | 53818724 | 3.35E-83 | 0.0159 | 0.1905 |
|  | rs62033404 | 16 | 53822239 | 3.35E-83 | 0.0159 | 0.2063 |
|  | rs1121980 | 16 | 53809247 | 3.35E-83 | 0.0159 | 0.2222 |
|  | rs1421086 | 16 | 53801343 | 3.35E-83 | 0.0159 | 0.2381 |
|  | rs9923233 | 16 | 53819198 | 3.35E-83 | 0.0159 | 0.2540 |
|  | rs8050136 | 16 | 53816275 | 3.35E-83 | 0.0159 | 0.2698 |
|  | rs9937354 | 16 | 53799847 | 3.35E-83 | 0.0159 | 0.2857 |
|  | rs17817712 | 16 | 53821125 | 3.35E-83 | 0.0159 | 0.3016 |
|  | rs7206122 | 16 | 53822440 | 3.35E-83 | 0.0159 | 0.3175 |
|  | rs56094641 | 16 | 53806453 | 3.35E-83 | 0.0159 | 0.3333 |
|  | rs62033399 | 16 | 53810943 | 3.35E-83 | 0.0159 | 0.3492 |
|  | rs9939609 | 16 | 53820527 | 3.35E-83 | 0.0159 | 0.3651 |
|  | rs11075991 | 16 | 53819937 | 3.35E-83 | 0.0159 | 0.3810 |
|  | rs9930397 | 16 | 53799985 | 3.35E-83 | 0.0159 | 0.3968 |
|  | rs9935401 | 16 | 53816838 | 3.35E-83 | 0.0159 | 0.4127 |
|  | rs56313538 | 16 | 53818834 | 3.35E-83 | 0.0159 | 0.4286 |
|  | rs3751812 | 16 | 53818460 | 3.35E-83 | 0.0159 | 0.4444 |
|  | rs72803697 | 16 | 53822183 | 3.35E-83 | 0.0159 | 0.4603 |
|  | rs9972653 | 16 | 53814363 | 3.35E-83 | 0.0159 | 0.4762 |
|  | rs62033405 | 16 | 53822387 | 3.35E-83 | 0.0159 | 0.4921 |
|  | rs17817449 | 16 | 53813367 | 3.35E-83 | 0.0159 | 0.5079 |
|  | rs7202116 | 16 | 53821615 | 3.35E-83 | 0.0159 | 0.5238 |
|  | rs7206629 | 16 | 53821413 | 3.35E-83 | 0.0159 | 0.5397 |
|  | rs9926289 | 16 | 53820503 | 3.35E-83 | 0.0159 | 0.5556 |
|  | rs8043757 | 16 | 53813450 | 3.35E-83 | 0.0159 | 0.5714 |
|  | rs7201850 | 16 | 53821862 | 3.35E-83 | 0.0159 | 0.5873 |
|  | rs11075985 | 16 | 53805207 | 3.35E-83 | 0.0159 | 0.6032 |
|  | rs9939973 | 16 | 53800568 | 3.35E-83 | 0.0159 | 0.6190 |
|  | rs11075990 | 16 | 53819893 | 3.35E-83 | 0.0159 | 0.6349 |
|  | rs9923147 | 16 | 53801549 | 3.35E-83 | 0.0159 | 0.6508 |
|  | rs8051591 | 16 | 53816752 | 3.35E-83 | 0.0159 | 0.6667 |
|  | rs9936385 | 16 | 53819169 | 3.35E-83 | 0.0159 | 0.6825 |
|  | rs9923312 | 16 | 53819367 | 3.35E-83 | 0.0159 | 0.6984 |
|  | rs11075989 | 16 | 53819877 | 3.35E-83 | 0.0159 | 0.7143 |
|  | rs7193144 | 16 | 53810686 | 3.35E-83 | 0.0159 | 0.7302 |
|  | rs9923544 | 16 | 53801985 | 3.35E-83 | 0.0159 | 0.7460 |
|  | rs17817964 | 16 | 53828066 | 3.35E-83 | 0.0159 | 0.7619 |
|  | rs9937053 | 16 | 53799507 | 3.35E-83 | 0.0159 | 0.7778 |
|  | rs1558901 | 16 | 53803187 | 3.35E-83 | 0.0159 | 0.7937 |
|  | rs9940278 | 16 | 53800200 | 3.35E-83 | 0.0159 | 0.8095 |
|  | rs9940646 | 16 | 53800629 | 3.35E-83 | 0.0159 | 0.8254 |
|  | rs7185735 | 16 | 53822651 | 3.35E-83 | 0.0159 | 0.8413 |
|  | rs1421085 | 16 | 53800954 | 3.35E-83 | 0.0159 | 0.8571 |
|  | rs11075992 | 16 | 53820066 | 3.35E-83 | 0.0159 | 0.8730 |
|  | rs9928094 | 16 | 53799905 | 3.35E-83 | 0.0159 | 0.8889 |
|  | rs11075988 | 16 | 53819771 | 3.35E-83 | 0.0159 | 0.9048 |
|  | rs62033403 | 16 | 53822237 | 3.35E-83 | 0.0159 | 0.9206 |
|  | rs11642015 | 16 | 53802494 | 3.35E-83 | 0.0159 | 0.9365 |
|  | rs28567725 | 16 | 53826028 | 3.35E-83 | 0.0159 | 0.9524 |
|  | rs66908032 | 16 | 53822142 | 3.35E-83 | 0.0159 | 0.9683 |
|  | rs7206410 | 16 | 53821297 | 3.35E-83 | 0.0159 | 0.9841 |
|  | rs62048402 | 16 | 53803223 | 3.35E-83 | 0.0159 | **1.0000** |
| rs11244061 | rs11244061 | 9 | 136153981 | 5.27E-14 | 0.9957 | 0.9957 |
| rs1169288 | rs1169288 | 12 | 121416650 | 5.75E-30 | 0.8318 | 0.8318 |
|  | rs1169299 | 12 | 121429194 | 3.44E-29 | 0.1409 | 0.9726 |
|  | rs2244608 | 12 | 121416988 | 3.91E-28 | 0.0127 | 0.9853 |
|  | rs2393791 | 12 | 121423956 | 2.16E-27 | 0.0023 | 0.9876 |
|  | rs2393775 | 12 | 121424574 | 2.50E-27 | 0.0020 | 0.9896 |
|  | rs2393776 | 12 | 121424406 | 2.50E-27 | 0.0020 | 0.9916 |
| rs1169307 | rs1169303 | 12 | 121436376 | 3.57E-27 | 0.7370 | 0.7370 |
|  | rs1169302 | 12 | 121432302 | 1.77E-26 | 0.1506 | 0.8876 |
|  | rs2258043 | 12 | 121451425 | 2.47E-26 | 0.1083 | 0.9959 |
| rs1260326 | rs1260326 | 2 | 27730940 | 1.00E-46 | 0.9999 | 0.9999 |
| rs13029250 | rs13029250 | 2 | 43638712 | 2.22E-22 | 1.0000 | **1.0000** |
| rs1800961 | rs1800961 | 20 | 43042364 | 5.69E-58 | 1.0000 | **1.0000** |
| rs2523504 | rs2857605 | 6 | 31524851 | 1.07E-20 | 0.9434 | 0.9434 |
|  | rs2523504 | 6 | 31510858 | 9.66E-19 | 0.0110 | 0.9545 |
|  | rs2239525 | 6 | 31509372 | 1.15E-18 | 0.0093 | 0.9638 |
|  | rs3130055 | 6 | 31497399 | 1.42E-18 | 0.0075 | 0.9713 |
|  | rs2523507 | 6 | 31509355 | 1.42E-18 | 0.0075 | 0.9788 |
|  | rs1055388 | 6 | 31501737 | 1.58E-18 | 0.0068 | 0.9856 |
|  | rs2239526 | 6 | 31509432 | 1.67E-18 | 0.0064 | 0.9921 |
| rs2857609 | rs2857609 | 6 | 31577825 | 1.36E-22 | 0.2848 | 0.2848 |
|  | rs3131377 | 6 | 31639420 | 1.99E-22 | 0.1952 | 0.4800 |
|  | rs3131376 | 6 | 31646683 | 2.08E-22 | 0.1862 | 0.6663 |
|  | rs3115668 | 6 | 31641485 | 2.08E-22 | 0.1862 | 0.8525 |
|  | rs3130068 | 6 | 31590354 | 3.19E-22 | 0.1221 | 0.9746 |
|  | rs3115669 | 6 | 31619024 | 6.64E-21 | 0.0061 | 0.9806 |
|  | rs3117189 | 6 | 32033944 | 1.06E-20 | 0.0038 | 0.9844 |
|  | rs3130287 | 6 | 32050544 | 1.54E-20 | 0.0026 | 0.9871 |
|  | rs3117181 | 6 | 32071017 | 1.55E-20 | 0.0026 | 0.9897 |
|  | rs2857600 | 6 | 31582287 | 1.76E-20 | 0.0023 | 0.9920 |
| rs28929474 | rs28929474 | 14 | 94844947 | 1.32E-41 | 0.9998 | 0.9998 |
| rs3130279 | rs3131377 | 6 | 31639420 | 1.99E-22 | 0.3106 | 0.3106 |
|  | rs3115668 | 6 | 31641485 | 2.08E-22 | 0.2963 | 0.6069 |
|  | rs3131376 | 6 | 31646683 | 2.08E-22 | 0.2963 | 0.9032 |
|  | rs3130279 | 6 | 32112626 | 2.84E-21 | 0.0223 | 0.9256 |
|  | rs9267807 | 6 | 32112955 | 3.11E-21 | 0.0204 | 0.9460 |
|  | rs3115669 | 6 | 31619024 | 6.64E-21 | 0.0097 | 0.9557 |
|  | rs3130342 | 6 | 32080146 | 7.63E-21 | 0.0084 | 0.9641 |
|  | rs3117189 | 6 | 32033944 | 1.06E-20 | 0.0060 | 0.9701 |
|  | rs3134963 | 6 | 32102305 | 1.14E-20 | 0.0057 | 0.9758 |
|  | rs3130287 | 6 | 32050544 | 1.54E-20 | 0.0042 | 0.9800 |
|  | rs3117181 | 6 | 32071017 | 1.55E-20 | 0.0042 | 0.9841 |
|  | rs3130283 | 6 | 32138545 | 2.11E-20 | 0.0031 | 0.9872 |
|  | rs3130285 | 6 | 32026257 | 3.80E-20 | 0.0017 | 0.9889 |
|  | rs9267551 | 6 | 31697957 | 4.09E-20 | 0.0016 | 0.9905 |
| rs35134156 | rs35134156 | 15 | 77315432 | 5.53E-15 | 0.9149 | 0.9149 |
|  | rs12148413 | 15 | 77316133 | 7.98E-14 | 0.0662 | 0.9811 |
|  | rs67501538 | 15 | 77314342 | 6.37E-13 | 0.0086 | 0.9897 |
|  | rs34127110 | 15 | 77313571 | 7.07E-13 | 0.0078 | 0.9975 |
| rs362307 | rs362307 | 4 | 3241845 | 3.47E-14 | 0.9654 | 0.9654 |
|  | rs76034781 | 4 | 3272782 | 1.03E-12 | 0.0345 | 0.9998 |
| rs429358 | rs429358 | 19 | 45411941 | 3.02E-25 | 0.9995 | 0.9995 |
| rs519790 | rs519790 | 11 | 72504141 | 1.31E-12 | 0.2917 | 0.2917 |
|  | rs481206 | 11 | 72497462 | 1.55E-12 | 0.2475 | 0.5392 |
|  | rs663015 | 11 | 72499035 | 1.59E-12 | 0.2410 | 0.7802 |
|  | rs12795307 | 11 | 72506324 | 1.93E-12 | 0.1995 | 0.9797 |
|  | rs4944014 | 11 | 72527180 | 8.11E-11 | 0.0051 | 0.9848 |
|  | rs3862794 | 11 | 72538600 | 1.25E-10 | 0.0034 | 0.9882 |
|  | rs3862796 | 11 | 72705522 | 2.20E-10 | 0.0019 | 0.9901 |
| rs58304657 | rs58304657 | 19 | 46176405 | 3.20E-19 | 0.4771 | 0.4771 |
|  | rs34089191 | 19 | 46176723 | 3.20E-19 | 0.4771 | 0.9543 |
|  | rs55669001 | 19 | 46177235 | 5.29E-18 | 0.0299 | 0.9842 |
|  | rs11671664 | 19 | 46172278 | 2.73E-17 | 0.0059 | 0.9901 |
| rs62052815 | rs62052815 | 16 | 69561826 | 3.51E-16 | 0.0791 | 0.0791 |
|  | rs244418 | 16 | 69622762 | 3.93E-16 | 0.0706 | 0.1497 |
|  | rs1364063 | 16 | 69588572 | 3.94E-16 | 0.0705 | 0.2201 |
|  | rs12923231 | 16 | 69572892 | 4.05E-16 | 0.0686 | 0.2888 |
|  | rs6499240 | 16 | 69686912 | 4.12E-16 | 0.0674 | 0.3562 |
|  | rs889399 | 16 | 69556583 | 4.23E-16 | 0.0656 | 0.4218 |
|  | rs862320 | 16 | 69651866 | 4.41E-16 | 0.0630 | 0.4849 |
|  | rs12933292 | 16 | 69566309 | 4.76E-16 | 0.0585 | 0.5434 |
|  | rs2917677 | 16 | 69750849 | 4.95E-16 | 0.0563 | 0.5997 |
|  | rs4783721 | 16 | 69561156 | 5.24E-16 | 0.0532 | 0.6529 |
|  | rs244415 | 16 | 69666683 | 5.24E-16 | 0.0532 | 0.7061 |
|  | rs4783722 | 16 | 69581912 | 5.59E-16 | 0.0499 | 0.7560 |
|  | rs244420 | 16 | 69657996 | 5.59E-16 | 0.0499 | 0.8060 |
|  | rs62052816 | 16 | 69563406 | 5.88E-16 | 0.0475 | 0.8535 |
|  | rs889398 | 16 | 69556715 | 6.59E-16 | 0.0425 | 0.8960 |
|  | rs35967356 | 16 | 69581354 | 8.51E-16 | 0.0330 | 0.9290 |
|  | rs12599391 | 16 | 69605349 | 8.88E-16 | 0.0316 | 0.9606 |
|  | rs1437134 | 16 | 69730426 | 1.41E-15 | 0.0201 | 0.9807 |
|  | rs7359336 | 16 | 69733460 | 1.59E-15 | 0.0179 | 0.9986 |
| rs72870502 | rs72870502 | 2 | 43920357 | 1.35E-14 | 1.0000 | **1.0000** |
| rs738408 | rs738408 | 22 | 44324730 | 1.25E-16 | 0.4667 | 0.4667 |
|  | rs3747207 | 22 | 44324855 | 2.06E-16 | 0.2847 | 0.7514 |
|  | rs738409 | 22 | 44324727 | 2.61E-16 | 0.2253 | 0.9767 |
|  | rs2294915 | 22 | 44340904 | 2.67E-15 | 0.0227 | 0.9994 |
| rs7461273 | rs2244648 | 8 | 11450422 | 9.03E-13 | 0.0777 | 0.0777 |
|  | rs7461273 | 8 | 11777977 | 1.20E-12 | 0.0586 | 0.1362 |
|  | rs6987692 | 8 | 11777849 | 3.00E-12 | 0.0239 | 0.1602 |
|  | rs34657250 | 8 | 11795373 | 3.31E-12 | 0.0217 | 0.1819 |
|  | rs55896564 | 8 | 11447093 | 3.96E-12 | 0.0182 | 0.2002 |
|  | rs2409798 | 8 | 11435564 | 4.03E-12 | 0.0179 | 0.2181 |
|  | rs2409799 | 8 | 11435927 | 4.03E-12 | 0.0179 | 0.2360 |
|  | rs11250179 | 8 | 11800332 | 4.05E-12 | 0.0178 | 0.2538 |
|  | rs7464553 | 8 | 11777901 | 4.68E-12 | 0.0155 | 0.2693 |
|  | rs7463895 | 8 | 11777884 | 4.91E-12 | 0.0147 | 0.2840 |
|  | rs10435719 | 8 | 11776904 | 5.18E-12 | 0.0140 | 0.2980 |
|  | rs11774631 | 8 | 11780655 | 5.45E-12 | 0.0133 | 0.3113 |
|  | rs4841629 | 8 | 11781223 | 5.45E-12 | 0.0133 | 0.3247 |
|  | rs13268030 | 8 | 11783073 | 5.45E-12 | 0.0133 | 0.3380 |
|  | rs7460153 | 8 | 11781997 | 5.45E-12 | 0.0133 | 0.3513 |
|  | rs11250178 | 8 | 11800234 | 5.70E-12 | 0.0128 | 0.3641 |
|  | rs7014580 | 8 | 11803262 | 5.70E-12 | 0.0128 | 0.3768 |
|  | rs28838382 | 8 | 11783828 | 5.73E-12 | 0.0127 | 0.3895 |
|  | rs28488162 | 8 | 11778994 | 5.73E-12 | 0.0127 | 0.4022 |
|  | rs35890869 | 8 | 11786781 | 5.73E-12 | 0.0127 | 0.4149 |
|  | rs9886639 | 8 | 11784529 | 5.73E-12 | 0.0127 | 0.4276 |
|  | rs11996277 | 8 | 11800031 | 5.75E-12 | 0.0126 | 0.4402 |
|  | rs35778860 | 8 | 11791338 | 6.33E-12 | 0.0115 | 0.4517 |
|  | rs12719915 | 8 | 11786255 | 6.33E-12 | 0.0115 | 0.4632 |
|  | rs34266352 | 8 | 11791462 | 6.65E-12 | 0.0110 | 0.4742 |
|  | rs13256554 | 8 | 11432946 | 6.68E-12 | 0.0109 | 0.4851 |
|  | rs13270267 | 8 | 11444837 | 6.79E-12 | 0.0107 | 0.4958 |
|  | rs9329251 | 8 | 11793606 | 6.99E-12 | 0.0104 | 0.5063 |
|  | rs9692668 | 8 | 11789933 | 6.99E-12 | 0.0104 | 0.5167 |
|  | rs7012446 | 8 | 11780944 | 7.26E-12 | 0.0101 | 0.5268 |
|  | rs7825529 | 8 | 11794444 | 7.35E-12 | 0.0099 | 0.5367 |
|  | rs4367597 | 8 | 11793529 | 7.35E-12 | 0.0099 | 0.5466 |
|  | rs56102998 | 8 | 11796675 | 7.35E-12 | 0.0099 | 0.5566 |
|  | rs35905106 | 8 | 11794745 | 7.35E-12 | 0.0099 | 0.5665 |
|  | rs7821336 | 8 | 11793955 | 7.35E-12 | 0.0099 | 0.5765 |
|  | rs10216773 | 8 | 11793680 | 7.35E-12 | 0.0099 | 0.5864 |
|  | rs7824267 | 8 | 11794279 | 7.35E-12 | 0.0099 | 0.5963 |
|  | rs4841567 | 8 | 11440019 | 7.63E-12 | 0.0096 | 0.6059 |
|  | rs34656282 | 8 | 11780158 | 7.71E-12 | 0.0095 | 0.6154 |
|  | rs13276433 | 8 | 11783036 | 7.71E-12 | 0.0095 | 0.6249 |
|  | rs6999030 | 8 | 11795308 | 7.72E-12 | 0.0095 | 0.6343 |
|  | rs13269417 | 8 | 11791962 | 7.72E-12 | 0.0095 | 0.6438 |
|  | rs7815595 | 8 | 11792351 | 7.72E-12 | 0.0095 | 0.6533 |
|  | rs7820338 | 8 | 11793925 | 7.72E-12 | 0.0095 | 0.6628 |
|  | rs13252854 | 8 | 11792978 | 7.72E-12 | 0.0095 | 0.6722 |
|  | rs13281077 | 8 | 11794814 | 7.72E-12 | 0.0095 | 0.6817 |
|  | rs12719914 | 8 | 11786129 | 8.10E-12 | 0.0090 | 0.6907 |
|  | rs7815572 | 8 | 11792299 | 8.11E-12 | 0.0090 | 0.6997 |
|  | rs4841630 | 8 | 11781567 | 8.50E-12 | 0.0086 | 0.7084 |
|  | rs7816182 | 8 | 11792701 | 8.52E-12 | 0.0086 | 0.7170 |
|  | rs13261205 | 8 | 11791216 | 8.93E-12 | 0.0082 | 0.7252 |
|  | rs34419349 | 8 | 11791557 | 8.93E-12 | 0.0082 | 0.7334 |
|  | rs10089712 | 8 | 11787505 | 9.37E-12 | 0.0078 | 0.7412 |
|  | rs34535807 | 8 | 11791525 | 9.37E-12 | 0.0078 | 0.7490 |
|  | rs57356272 | 8 | 11797180 | 9.41E-12 | 0.0078 | 0.7568 |
|  | rs11250175 | 8 | 11792758 | 9.41E-12 | 0.0078 | 0.7646 |
|  | rs35558344 | 8 | 11434232 | 9.81E-12 | 0.0075 | 0.7721 |
|  | rs9693925 | 8 | 11789963 | 9.84E-12 | 0.0075 | 0.7796 |
|  | rs4841638 | 8 | 11796023 | 9.84E-12 | 0.0075 | 0.7871 |
|  | rs7842810 | 8 | 11794163 | 9.84E-12 | 0.0075 | 0.7945 |
|  | rs10113145 | 8 | 11793295 | 1.03E-11 | 0.0071 | 0.8017 |
|  | rs10113062 | 8 | 11793249 | 1.03E-11 | 0.0071 | 0.8088 |
|  | rs7812563 | 8 | 11794488 | 1.03E-11 | 0.0071 | 0.8159 |
|  | rs4841641 | 8 | 11798227 | 1.04E-11 | 0.0071 | 0.8230 |
|  | rs11250177 | 8 | 11799099 | 1.04E-11 | 0.0071 | 0.8301 |
|  | rs4841639 | 8 | 11796093 | 1.08E-11 | 0.0068 | 0.8368 |
|  | rs7815186 | 8 | 11792062 | 1.08E-11 | 0.0068 | 0.8436 |
|  | rs7815179 | 8 | 11792048 | 1.08E-11 | 0.0068 | 0.8504 |
|  | rs11250176 | 8 | 11792816 | 1.08E-11 | 0.0068 | 0.8572 |
|  | rs7833079 | 8 | 11792129 | 1.08E-11 | 0.0068 | 0.8640 |
|  | rs13252853 | 8 | 11792974 | 1.08E-11 | 0.0068 | 0.8708 |
|  | rs10113042 | 8 | 11793178 | 1.08E-11 | 0.0068 | 0.8776 |
|  | rs4840597 | 8 | 11796068 | 1.14E-11 | 0.0065 | 0.8841 |
|  | rs13279577 | 8 | 11793096 | 1.14E-11 | 0.0065 | 0.8905 |
|  | rs10112958 | 8 | 11793140 | 1.25E-11 | 0.0059 | 0.8964 |
|  | rs35391955 | 8 | 11796728 | 1.25E-11 | 0.0059 | 0.9023 |
|  | rs35181953 | 8 | 11435049 | 1.26E-11 | 0.0059 | 0.9082 |
|  | rs60176945 | 8 | 11796674 | 1.32E-11 | 0.0056 | 0.9138 |
|  | rs10087494 | 8 | 11792734 | 1.32E-11 | 0.0056 | 0.9194 |
|  | rs7464263 | 8 | 11434176 | 1.35E-11 | 0.0055 | 0.9249 |
|  | rs2898290 | 8 | 11433909 | 1.35E-11 | 0.0055 | 0.9304 |
|  | rs2127127 | 8 | 11434792 | 1.49E-11 | 0.0050 | 0.9353 |
|  | rs35496345 | 8 | 11791678 | 1.53E-11 | 0.0048 | 0.9402 |
|  | rs9774603 | 8 | 11777406 | 1.93E-11 | 0.0039 | 0.9440 |
|  | rs10097870 | 8 | 11444516 | 2.20E-11 | 0.0034 | 0.9474 |
|  | rs7459545 | 8 | 11804389 | 2.37E-11 | 0.0032 | 0.9506 |
|  | rs34583868 | 8 | 11791383 | 2.49E-11 | 0.0030 | 0.9536 |
|  | rs7833685 | 8 | 11792535 | 2.73E-11 | 0.0028 | 0.9563 |
|  | rs7459983 | 8 | 11807221 | 3.46E-11 | 0.0022 | 0.9585 |
|  | rs4840599 | 8 | 11805380 | 3.46E-11 | 0.0022 | 0.9607 |
|  | rs6601633 | 8 | 11805173 | 3.46E-11 | 0.0022 | 0.9629 |
|  | rs7006538 | 8 | 11804982 | 3.46E-11 | 0.0022 | 0.9651 |
|  | rs13268217 | 8 | 11782609 | 4.59E-11 | 0.0017 | 0.9667 |
|  | rs13256329 | 8 | 11782214 | 4.59E-11 | 0.0017 | 0.9684 |
|  | rs13264994 | 8 | 11782759 | 4.59E-11 | 0.0017 | 0.9700 |
|  | rs28510449 | 8 | 11809263 | 4.81E-11 | 0.0016 | 0.9716 |
|  | rs36057214 | 8 | 11783690 | 4.82E-11 | 0.0016 | 0.9732 |
|  | rs7011924 | 8 | 11780593 | 5.05E-11 | 0.0015 | 0.9747 |
|  | rs6996342 | 8 | 11784541 | 5.05E-11 | 0.0015 | 0.9762 |
|  | rs12216858 | 8 | 11783118 | 5.05E-11 | 0.0015 | 0.9777 |
|  | rs13255498 | 8 | 11787203 | 5.29E-11 | 0.0014 | 0.9791 |
|  | rs35010200 | 8 | 11785655 | 5.29E-11 | 0.0014 | 0.9806 |
|  | rs2409797 | 8 | 11433780 | 5.69E-11 | 0.0013 | 0.9819 |
|  | rs10108511 | 8 | 11435516 | 5.69E-11 | 0.0013 | 0.9832 |
|  | rs61468577 | 8 | 11784925 | 6.00E-11 | 0.0013 | 0.9845 |
|  | rs10216693 | 8 | 11792902 | 6.09E-11 | 0.0013 | 0.9858 |
|  | rs28641694 | 8 | 11784045 | 6.59E-11 | 0.0012 | 0.9869 |
|  | rs4841634 | 8 | 11781880 | 6.59E-11 | 0.0012 | 0.9881 |
|  | rs4240678 | 8 | 11802426 | 6.69E-11 | 0.0011 | 0.9892 |
|  | rs13275808 | 8 | 11782815 | 6.90E-11 | 0.0011 | 0.9926 |
| rs76747430 | rs76747430 | 22 | 40833842 | 1.19E-12 | 0.8806 | 0.8806 |
|  | rs75817276 | 22 | 40872654 | 1.32E-11 | 0.0837 | 0.9643 |
|  | rs7284704 | 22 | 40845131 | 4.66E-11 | 0.0243 | 0.9886 |
|  | rs56755140 | 22 | 41066598 | 2.14E-10 | 0.0055 | 0.9941 |
| rs879882 | rs879882 | 6 | 31139452 | 3.88E-18 | 0.8599 | 0.8599 |
|  | rs3132520 | 6 | 31140008 | 1.24E-16 | 0.0282 | 0.8881 |
|  | rs2240063 | 6 | 31114745 | 2.60E-16 | 0.0136 | 0.9017 |
|  | rs3130695 | 6 | 31211050 | 3.15E-16 | 0.0112 | 0.9129 |
|  | rs3131012 | 6 | 31115441 | 3.28E-16 | 0.0108 | 0.9237 |
|  | rs3095249 | 6 | 31209245 | 5.35E-16 | 0.0067 | 0.9304 |
|  | rs3132497 | 6 | 31208591 | 5.35E-16 | 0.0067 | 0.9371 |
|  | rs3130408 | 6 | 31213791 | 5.59E-16 | 0.0064 | 0.9434 |
|  | rs3134756 | 6 | 31214873 | 5.59E-16 | 0.0064 | 0.9498 |
|  | rs2240064 | 6 | 31114573 | 6.57E-16 | 0.0054 | 0.9553 |
|  | rs2240059 | 6 | 31120614 | 9.88E-16 | 0.0036 | 0.9589 |
|  | rs3130500 | 6 | 31119976 | 1.04E-15 | 0.0035 | 0.9624 |
|  | rs3130504 | 6 | 31137837 | 1.32E-15 | 0.0027 | 0.9651 |
|  | rs2073723 | 6 | 31130078 | 1.32E-15 | 0.0027 | 0.9678 |
|  | rs3130501 | 6 | 31136453 | 1.32E-15 | 0.0027 | 0.9706 |
|  | rs1065461 | 6 | 31130502 | 1.45E-15 | 0.0025 | 0.9731 |
|  | rs3132523 | 6 | 31136832 | 1.68E-15 | 0.0022 | 0.9752 |
|  | rs3132524 | 6 | 31136714 | 1.77E-15 | 0.0021 | 0.9773 |
|  | rs3130502 | 6 | 31136666 | 1.77E-15 | 0.0021 | 0.9794 |
|  | rs3132528 | 6 | 31131569 | 1.91E-15 | 0.0019 | 0.9813 |
|  | rs3130451 | 6 | 31123242 | 2.04E-15 | 0.0018 | 0.9830 |
|  | rs3130929 | 6 | 31135207 | 2.04E-15 | 0.0018 | 0.9848 |
|  | rs3130928 | 6 | 31136097 | 2.10E-15 | 0.0017 | 0.9865 |
|  | rs3130498 | 6 | 31117619 | 2.15E-15 | 0.0017 | 0.9882 |
|  | rs3130499 | 6 | 31119889 | 2.15E-15 | 0.0017 | 0.9899 |
|  | rs130078 | 6 | 31118565 | 2.25E-15 | 0.0016 | 0.9916 |

CHR, chromosome; CumSum, cumulative sum of posterior inclusion probability; PIP, posterior inclusion probability; POS, position; SNPs, single nucleotide Polymorphisms.

## Table S13. List of SNPs in the 99% credible set identified from fine-mapping analysis for each CPASSOC-identified locus shared between type 2 diabetes mellitus (adjusted for BMI) and gallstone disease.

| **Index SNPs** | **Credible-set SNPs** | **CHR** | **POS** | ***P*-CPASSOC** | **PIP** | **CumSum** |
| --- | --- | --- | --- | --- | --- | --- |
| rs1169288 | rs1169288 | 12 | 121416650 | 5.32E-31 | 0.992 | 0.992 |
| rs1169307 | rs1169303 | 12 | 121436376 | 3.27E-22 | 0.455 | 0.455 |
|  | rs2258043 | 12 | 121451425 | 5.05E-22 | 0.296 | 0.751 |
|  | rs1169302 | 12 | 121432302 | 6.05E-22 | 0.248 | 0.998 |
| rs1260326 | rs1260326 | 2 | 27730940 | 6.81E-40 | 0.998 | 0.998 |
| rs149797 | rs149797 | 5 | 72227620 | 3.52E-11 | 0.078 | 0.078 |
|  | rs198214 | 5 | 72219751 | 3.95E-11 | 0.070 | 0.148 |
|  | rs6880884 | 5 | 72241468 | 4.18E-11 | 0.066 | 0.214 |
|  | rs155626 | 5 | 72211224 | 5.84E-11 | 0.048 | 0.262 |
|  | rs7726959 | 5 | 72276017 | 6.80E-11 | 0.041 | 0.303 |
|  | rs7712921 | 5 | 72276099 | 7.19E-11 | 0.039 | 0.342 |
|  | rs62360696 | 5 | 72256434 | 7.60E-11 | 0.037 | 0.379 |
|  | rs12153586 | 5 | 72380958 | 1.45E-10 | 0.020 | 0.398 |
|  | rs11743222 | 5 | 72377288 | 1.53E-10 | 0.019 | 0.417 |
|  | rs11743168 | 5 | 72377162 | 1.53E-10 | 0.019 | 0.435 |
|  | rs60714547 | 5 | 72379305 | 1.53E-10 | 0.019 | 0.454 |
|  | rs7726559 | 5 | 72376790 | 1.62E-10 | 0.018 | 0.472 |
|  | rs6888510 | 5 | 72372329 | 1.80E-10 | 0.016 | 0.487 |
|  | rs6883874 | 5 | 72372140 | 1.80E-10 | 0.016 | 0.503 |
|  | rs113869457 | 5 | 72246721 | 1.80E-10 | 0.016 | 0.519 |
|  | rs57783755 | 5 | 72369532 | 1.90E-10 | 0.015 | 0.534 |
|  | rs62362211 | 5 | 72372861 | 2.24E-10 | 0.013 | 0.547 |
|  | rs12153061 | 5 | 72381100 | 2.74E-10 | 0.011 | 0.558 |
|  | rs6884621 | 5 | 72376072 | 2.74E-10 | 0.011 | 0.568 |
|  | rs55769858 | 5 | 72280764 | 2.89E-10 | 0.010 | 0.578 |
|  | rs17730967 | 5 | 72380529 | 2.89E-10 | 0.010 | 0.588 |
|  | rs6871266 | 5 | 72325970 | 2.89E-10 | 0.010 | 0.598 |
|  | rs62362165 | 5 | 72320012 | 3.04E-10 | 0.009 | 0.608 |
|  | rs62362163 | 5 | 72310761 | 3.04E-10 | 0.009 | 0.617 |
|  | rs716327 | 5 | 72361632 | 3.05E-10 | 0.009 | 0.626 |
|  | rs10942539 | 5 | 72357086 | 3.05E-10 | 0.009 | 0.636 |
|  | rs10942534 | 5 | 72331910 | 3.05E-10 | 0.009 | 0.645 |
|  | rs62362196 | 5 | 72349190 | 3.05E-10 | 0.009 | 0.655 |
|  | rs62362197 | 5 | 72349264 | 3.05E-10 | 0.009 | 0.664 |
|  | rs6452801 | 5 | 72338727 | 3.05E-10 | 0.009 | 0.674 |
|  | rs7725821 | 5 | 72338981 | 3.05E-10 | 0.009 | 0.683 |
|  | rs3911450 | 5 | 72326444 | 3.05E-10 | 0.009 | 0.693 |
|  | rs11738261 | 5 | 72354766 | 3.05E-10 | 0.009 | 0.702 |
|  | rs56754821 | 5 | 72337851 | 3.05E-10 | 0.009 | 0.712 |
|  | rs6452798 | 5 | 72338469 | 3.05E-10 | 0.009 | 0.721 |
|  | rs62362195 | 5 | 72348030 | 3.05E-10 | 0.009 | 0.731 |
|  | rs6880813 | 5 | 72362929 | 3.05E-10 | 0.009 | 0.740 |
|  | rs62362160 | 5 | 72296385 | 3.21E-10 | 0.009 | 0.749 |
|  | rs58820200 | 5 | 72364917 | 3.22E-10 | 0.009 | 0.758 |
|  | rs62360750 | 5 | 72273850 | 3.39E-10 | 0.009 | 0.767 |
|  | rs10515154 | 5 | 72329075 | 3.39E-10 | 0.009 | 0.775 |
|  | rs12186995 | 5 | 72335327 | 3.39E-10 | 0.009 | 0.784 |
|  | rs62362203 | 5 | 72357741 | 3.57E-10 | 0.008 | 0.792 |
|  | rs7730663 | 5 | 72360801 | 3.57E-10 | 0.008 | 0.800 |
|  | rs57149385 | 5 | 72356181 | 3.57E-10 | 0.008 | 0.808 |
|  | rs1156398 | 5 | 72362150 | 3.57E-10 | 0.008 | 0.816 |
|  | rs62362190 | 5 | 72341518 | 3.57E-10 | 0.008 | 0.824 |
|  | rs7706969 | 5 | 72345763 | 3.57E-10 | 0.008 | 0.832 |
|  | rs62362198 | 5 | 72349477 | 3.57E-10 | 0.008 | 0.841 |
|  | rs6875819 | 5 | 72358789 | 3.57E-10 | 0.008 | 0.849 |
|  | rs6861844 | 5 | 72375780 | 3.57E-10 | 0.008 | 0.857 |
|  | rs7349774 | 5 | 72351180 | 3.57E-10 | 0.008 | 0.865 |
|  | rs2277017 | 5 | 72378015 | 3.57E-10 | 0.008 | 0.873 |
|  | rs6452804 | 5 | 72378863 | 3.57E-10 | 0.008 | 0.881 |
|  | rs7712838 | 5 | 72347223 | 3.57E-10 | 0.008 | 0.889 |
|  | rs62362193 | 5 | 72345509 | 3.59E-10 | 0.008 | 0.897 |
|  | rs6890953 | 5 | 72343856 | 3.77E-10 | 0.008 | 0.905 |
|  | rs6892578 | 5 | 72325334 | 3.77E-10 | 0.008 | 0.913 |
|  | rs16902297 | 5 | 72262482 | 3.77E-10 | 0.008 | 0.920 |
|  | rs1568712 | 5 | 72365856 | 3.98E-10 | 0.007 | 0.928 |
|  | rs155428 | 5 | 72109700 | 4.09E-10 | 0.007 | 0.935 |
|  | rs155625 | 5 | 72206862 | 5.47E-10 | 0.005 | 0.940 |
|  | rs12189525 | 5 | 72389272 | 6.08E-10 | 0.005 | 0.945 |
|  | rs6895786 | 5 | 72391011 | 6.71E-10 | 0.004 | 0.949 |
|  | rs11747525 | 5 | 72392597 | 6.71E-10 | 0.004 | 0.954 |
|  | rs62362239 | 5 | 72388732 | 7.08E-10 | 0.004 | 0.958 |
|  | rs6892973 | 5 | 72385914 | 7.08E-10 | 0.004 | 0.962 |
|  | rs12188789 | 5 | 72389197 | 7.47E-10 | 0.004 | 0.966 |
|  | rs10063568 | 5 | 72102924 | 8.44E-10 | 0.004 | 0.970 |
|  | rs6452814 | 5 | 72395915 | 1.20E-09 | 0.002 | 0.972 |
|  | rs10223082 | 5 | 72399063 | 1.25E-09 | 0.002 | 0.975 |
|  | rs7727222 | 5 | 72394095 | 1.26E-09 | 0.002 | 0.977 |
|  | rs6452816 | 5 | 72398657 | 1.39E-09 | 0.002 | 0.979 |
|  | rs7709974 | 5 | 72394735 | 1.46E-09 | 0.002 | 0.981 |
|  | rs6452813 | 5 | 72394785 | 1.46E-09 | 0.002 | 0.983 |
|  | rs7713398 | 5 | 72252475 | 3.33E-09 | 0.001 | 0.984 |
|  | rs258887 | 5 | 72277724 | 3.40E-09 | 0.001 | 0.985 |
|  | rs7734148 | 5 | 72160974 | 3.47E-09 | 0.001 | 0.986 |
|  | rs266422 | 5 | 72105250 | 3.65E-09 | 0.001 | 0.987 |
|  | rs1912004 | 5 | 72130646 | 3.67E-09 | 0.001 | 0.988 |
|  | rs16901618 | 5 | 72125995 | 3.87E-09 | 0.001 | 0.988 |
|  | rs6871112 | 5 | 72125370 | 3.87E-09 | 0.001 | 0.989 |
|  | rs4704044 | 5 | 72180647 | 4.82E-09 | 0.001 | 0.990 |
|  | rs2545646 | 5 | 72255808 | 4.87E-09 | 0.001 | 0.990 |
| rs1800961 | rs1800961 | 20 | 43042364 | 1.44E-59 | 1.000 | **1.000** |
| rs2239525 | rs2857605 | 6 | 31524851 | 9.12E-17 | 0.627 | 0.627 |
|  | rs2239525 | 6 | 31509372 | 7.54E-16 | 0.078 | 0.706 |
|  | rs2523504 | 6 | 31510858 | 8.15E-16 | 0.072 | 0.778 |
|  | rs2523507 | 6 | 31509355 | 9.64E-16 | 0.061 | 0.839 |
|  | rs2239526 | 6 | 31509432 | 1.16E-15 | 0.051 | 0.891 |
|  | rs3130055 | 6 | 31497399 | 2.02E-15 | 0.030 | 0.920 |
|  | rs1055388 | 6 | 31501737 | 2.28E-15 | 0.026 | 0.946 |
|  | rs933208 | 6 | 31506648 | 2.28E-15 | 0.026 | 0.973 |
|  | rs2075580 | 6 | 31503975 | 3.09E-15 | 0.020 | 0.992 |
| rs244418 | rs244418 | 16 | 69622762 | 2.94E-12 | 0.089 | 0.089 |
|  | rs244420 | 16 | 69657996 | 3.27E-12 | 0.081 | 0.170 |
|  | rs862320 | 16 | 69651866 | 3.33E-12 | 0.079 | 0.249 |
|  | rs62052815 | 16 | 69561826 | 3.65E-12 | 0.072 | 0.322 |
|  | rs244415 | 16 | 69666683 | 4.02E-12 | 0.066 | 0.388 |
|  | rs1437134 | 16 | 69730426 | 4.31E-12 | 0.062 | 0.449 |
|  | rs1364063 | 16 | 69588572 | 4.64E-12 | 0.057 | 0.506 |
|  | rs12599391 | 16 | 69605349 | 5.39E-12 | 0.049 | 0.556 |
|  | rs4783721 | 16 | 69561156 | 5.64E-12 | 0.047 | 0.603 |
|  | rs889399 | 16 | 69556583 | 6.02E-12 | 0.044 | 0.647 |
|  | rs62052816 | 16 | 69563406 | 6.38E-12 | 0.042 | 0.689 |
|  | rs4783722 | 16 | 69581912 | 6.74E-12 | 0.040 | 0.729 |
|  | rs12933292 | 16 | 69566309 | 6.81E-12 | 0.039 | 0.768 |
|  | rs889398 | 16 | 69556715 | 7.22E-12 | 0.037 | 0.805 |
|  | rs12923231 | 16 | 69572892 | 7.40E-12 | 0.036 | 0.841 |
|  | rs7359336 | 16 | 69733460 | 7.77E-12 | 0.035 | 0.876 |
|  | rs2917677 | 16 | 69750849 | 7.77E-12 | 0.035 | 0.910 |
|  | rs35967356 | 16 | 69581354 | 8.07E-12 | 0.033 | 0.944 |
|  | rs6499240 | 16 | 69686912 | 8.33E-12 | 0.032 | 0.976 |
|  | rs7193038 | 16 | 69926134 | 2.89E-10 | 0.001 | 0.977 |
|  | rs6499244 | 16 | 69735271 | 3.05E-10 | 0.001 | 0.978 |
|  | rs7196842 | 16 | 69923333 | 3.58E-10 | 0.001 | 0.979 |
|  | rs3790078 | 16 | 69906182 | 3.79E-10 | 0.001 | 0.980 |
|  | rs3790076 | 16 | 69907445 | 3.93E-10 | 0.001 | 0.980 |
|  | rs3790075 | 16 | 69907811 | 4.25E-10 | 0.001 | 0.981 |
|  | rs6499270 | 16 | 69917740 | 4.40E-10 | 0.001 | 0.982 |
|  | rs6499268 | 16 | 69906529 | 4.40E-10 | 0.001 | 0.982 |
|  | rs8060478 | 16 | 69902870 | 4.66E-10 | 0.001 | 0.983 |
|  | rs12926791 | 16 | 69849546 | 4.80E-10 | 0.001 | 0.984 |
|  | rs7190013 | 16 | 69908248 | 4.93E-10 | 0.001 | 0.984 |
|  | rs8044876 | 16 | 69916104 | 5.04E-10 | 0.001 | 0.985 |
|  | rs2362641 | 16 | 69915761 | 5.21E-10 | 0.001 | 0.985 |
|  | rs1500337 | 16 | 69901884 | 5.33E-10 | 0.001 | 0.986 |
|  | rs3790080 | 16 | 69902139 | 5.33E-10 | 0.001 | 0.986 |
|  | rs68161338 | 16 | 69823778 | 5.56E-10 | 0.001 | 0.987 |
|  | rs12921407 | 16 | 69860933 | 5.56E-10 | 0.001 | 0.987 |
|  | rs8047682 | 16 | 69913446 | 5.84E-10 | 0.001 | 0.988 |
|  | rs4985448 | 16 | 69899395 | 5.97E-10 | 0.000 | 0.988 |
|  | rs10852461 | 16 | 69928846 | 5.97E-10 | 0.000 | 0.989 |
|  | rs1566453 | 16 | 69899787 | 6.17E-10 | 0.000 | 0.989 |
|  | rs4296254 | 16 | 69881268 | 6.22E-10 | 0.000 | 0.990 |
|  | rs6499263 | 16 | 69890149 | 6.32E-10 | 0.000 | 0.990 |
| rs2857609 | rs2857609 | 6 | 31577825 | 5.78E-17 | 0.276 | 0.276 |
|  | rs3131377 | 6 | 31639420 | 8.72E-17 | 0.184 | 0.460 |
|  | rs3115668 | 6 | 31641485 | 9.18E-17 | 0.175 | 0.634 |
|  | rs3131376 | 6 | 31646683 | 9.18E-17 | 0.175 | 0.809 |
|  | rs3130068 | 6 | 31590354 | 1.06E-16 | 0.152 | 0.961 |
|  | rs3117189 | 6 | 32033944 | 2.80E-15 | 0.006 | 0.967 |
|  | rs3130287 | 6 | 32050544 | 3.04E-15 | 0.006 | 0.972 |
|  | rs3130285 | 6 | 32026257 | 3.22E-15 | 0.005 | 0.978 |
|  | rs3117181 | 6 | 32071017 | 3.73E-15 | 0.005 | 0.982 |
|  | rs9267658 | 6 | 31845985 | 4.01E-15 | 0.004 | 0.986 |
|  | rs3096695 | 6 | 32069806 | 7.70E-15 | 0.002 | 0.989 |
|  | rs3134954 | 6 | 32071893 | 8.95E-15 | 0.002 | 0.990 |
| rs3130279 | rs3131377 | 6 | 31639420 | 8.72E-17 | 0.279 | 0.279 |
|  | rs3115668 | 6 | 31641485 | 9.18E-17 | 0.265 | 0.543 |
|  | rs3131376 | 6 | 31646683 | 9.18E-17 | 0.265 | 0.808 |
|  | rs3130279 | 6 | 32112626 | 6.04E-16 | 0.041 | 0.850 |
|  | rs9267807 | 6 | 32112955 | 6.65E-16 | 0.038 | 0.887 |
|  | rs3130283 | 6 | 32138545 | 2.10E-15 | 0.012 | 0.899 |
|  | rs3117189 | 6 | 32033944 | 2.80E-15 | 0.009 | 0.909 |
|  | rs3134603 | 6 | 32126002 | 2.89E-15 | 0.009 | 0.917 |
|  | rs3130287 | 6 | 32050544 | 3.04E-15 | 0.008 | 0.926 |
|  | rs3130342 | 6 | 32080146 | 3.08E-15 | 0.008 | 0.934 |
|  | rs3130285 | 6 | 32026257 | 3.22E-15 | 0.008 | 0.942 |
|  | rs3117181 | 6 | 32071017 | 3.73E-15 | 0.007 | 0.949 |
|  | rs9267658 | 6 | 31845985 | 4.01E-15 | 0.006 | 0.955 |
|  | rs3134963 | 6 | 32102305 | 4.71E-15 | 0.005 | 0.961 |
|  | rs3134943 | 6 | 32147761 | 5.15E-15 | 0.005 | 0.966 |
|  | rs3134604 | 6 | 32122386 | 6.65E-15 | 0.004 | 0.970 |
|  | rs3096695 | 6 | 32069806 | 7.70E-15 | 0.003 | 0.973 |
|  | rs3134954 | 6 | 32071893 | 8.95E-15 | 0.003 | 0.976 |
|  | rs3130346 | 6 | 32131510 | 9.58E-15 | 0.003 | 0.979 |
|  | rs3134953 | 6 | 32109165 | 1.12E-14 | 0.002 | 0.981 |
|  | rs3131283 | 6 | 32119898 | 1.14E-14 | 0.002 | 0.983 |
|  | rs3130682 | 6 | 31884823 | 1.39E-14 | 0.002 | 0.985 |
|  | rs3115669 | 6 | 31619024 | 1.56E-14 | 0.002 | 0.987 |
|  | rs1800684 | 6 | 32151994 | 1.97E-14 | 0.001 | 0.988 |
|  | rs3130683 | 6 | 31888367 | 2.40E-14 | 0.001 | 0.989 |
|  | rs9267576 | 6 | 31812038 | 2.94E-14 | 0.001 | 0.990 |
| rs519790 | rs519790 | 11 | 72504141 | 6.60E-12 | 0.320 | 0.320 |
|  | rs12795307 | 11 | 72506324 | 8.35E-12 | 0.254 | 0.574 |
|  | rs663015 | 11 | 72499035 | 8.35E-12 | 0.254 | 0.827 |
|  | rs481206 | 11 | 72497462 | 2.11E-11 | 0.102 | 0.930 |
|  | rs4944014 | 11 | 72527180 | 1.05E-10 | 0.021 | 0.951 |
|  | rs3862794 | 11 | 72538600 | 1.83E-10 | 0.012 | 0.963 |
|  | rs11822039 | 11 | 72638704 | 4.42E-10 | 0.005 | 0.968 |
|  | rs3862796 | 11 | 72705522 | 4.83E-10 | 0.005 | 0.973 |
|  | rs4944016 | 11 | 72597438 | 6.48E-10 | 0.004 | 0.977 |
|  | rs10898889 | 11 | 72605382 | 7.22E-10 | 0.003 | 0.980 |
|  | rs12799445 | 11 | 72592506 | 7.91E-10 | 0.003 | 0.983 |
|  | rs7946861 | 11 | 72557986 | 1.01E-09 | 0.002 | 0.985 |
|  | rs10793048 | 11 | 72580398 | 1.12E-09 | 0.002 | 0.988 |
|  | rs12805658 | 11 | 72555300 | 1.37E-09 | 0.002 | 0.989 |
|  | rs4400839 | 11 | 72675665 | 1.67E-09 | 0.001 | 0.991 |
| rs56094641 | rs56094641 | 16 | 53806453 | 1.88E-16 | 0.102 | 0.102 |
|  | rs62048402 | 16 | 53803223 | 2.76E-16 | 0.070 | 0.172 |
|  | rs11075985 | 16 | 53805207 | 2.85E-16 | 0.068 | 0.241 |
|  | rs1421085 | 16 | 53800954 | 2.92E-16 | 0.066 | 0.307 |
|  | rs11642015 | 16 | 53802494 | 2.97E-16 | 0.065 | 0.372 |
|  | rs55872725 | 16 | 53809123 | 3.20E-16 | 0.061 | 0.433 |
|  | rs1558901 | 16 | 53803187 | 3.48E-16 | 0.056 | 0.489 |
|  | rs9928094 | 16 | 53799905 | 3.75E-16 | 0.052 | 0.540 |
|  | rs1558902 | 16 | 53803574 | 3.98E-16 | 0.049 | 0.589 |
|  | rs9940646 | 16 | 53800629 | 4.66E-16 | 0.042 | 0.631 |
|  | rs9923544 | 16 | 53801985 | 4.74E-16 | 0.041 | 0.672 |
|  | rs9937354 | 16 | 53799847 | 4.74E-16 | 0.041 | 0.714 |
|  | rs9930397 | 16 | 53799985 | 5.47E-16 | 0.036 | 0.749 |
|  | rs1421086 | 16 | 53801343 | 5.47E-16 | 0.036 | 0.785 |
|  | rs9940278 | 16 | 53800200 | 5.88E-16 | 0.033 | 0.818 |
|  | rs9923147 | 16 | 53801549 | 5.88E-16 | 0.033 | 0.852 |
|  | rs9939973 | 16 | 53800568 | 5.88E-16 | 0.033 | 0.885 |
|  | rs9937053 | 16 | 53799507 | 6.32E-16 | 0.031 | 0.916 |
|  | rs9940128 | 16 | 53800754 | 6.32E-16 | 0.031 | 0.947 |
|  | rs1121980 | 16 | 53809247 | 1.10E-15 | 0.018 | 0.965 |
|  | rs10468280 | 16 | 53827479 | 6.84E-15 | 0.003 | 0.968 |
|  | rs62033408 | 16 | 53827962 | 7.33E-15 | 0.003 | 0.971 |
|  | rs17817964 | 16 | 53828066 | 8.44E-15 | 0.002 | 0.973 |
|  | rs9931494 | 16 | 53827179 | 1.14E-14 | 0.002 | 0.975 |
|  | rs28567725 | 16 | 53826028 | 1.32E-14 | 0.002 | 0.976 |
|  | rs9941349 | 16 | 53825488 | 1.73E-14 | 0.001 | 0.977 |
|  | rs17817712 | 16 | 53821125 | 1.76E-14 | 0.001 | 0.979 |
|  | rs62033405 | 16 | 53822387 | 2.01E-14 | 0.001 | 0.980 |
|  | rs3751812 | 16 | 53818460 | 2.31E-14 | 0.001 | 0.981 |
|  | rs17817497 | 16 | 53815435 | 2.31E-14 | 0.001 | 0.981 |
|  | rs62033400 | 16 | 53811788 | 2.65E-14 | 0.001 | 0.982 |
|  | rs7201850 | 16 | 53821862 | 3.15E-14 | 0.001 | 0.983 |
|  | rs7202116 | 16 | 53821615 | 3.17E-14 | 0.001 | 0.984 |
|  | rs56313538 | 16 | 53818834 | 3.17E-14 | 0.001 | 0.984 |
|  | rs11075990 | 16 | 53819893 | 3.17E-14 | 0.001 | 0.985 |
|  | rs9936385 | 16 | 53819169 | 3.39E-14 | 0.001 | 0.986 |
|  | rs62033403 | 16 | 53822237 | 3.39E-14 | 0.001 | 0.986 |
|  | rs11075992 | 16 | 53820066 | 3.39E-14 | 0.001 | 0.987 |
|  | rs7206410 | 16 | 53821297 | 3.39E-14 | 0.001 | 0.987 |
|  | rs7185735 | 16 | 53822651 | 3.39E-14 | 0.001 | 0.988 |
|  | rs11075991 | 16 | 53819937 | 3.39E-14 | 0.001 | 0.989 |
|  | rs62033404 | 16 | 53822239 | 3.39E-14 | 0.001 | 0.989 |
|  | rs11075988 | 16 | 53819771 | 3.61E-14 | 0.001 | 0.990 |
|  | rs11075989 | 16 | 53819877 | 3.62E-14 | 0.001 | 0.990 |
| rs58304657 | rs58304657 | 19 | 46176405 | 2.50E-26 | 0.350 | 0.350 |
|  | rs34089191 | 19 | 46176723 | 2.50E-26 | 0.350 | 0.700 |
|  | rs55669001 | 19 | 46177235 | 2.93E-26 | 0.299 | 0.999 |
| rs736820 | rs736820 | 20 | 43034016 | 1.25E-11 | 0.746 | 0.746 |
|  | rs8114057 | 20 | 43036452 | 3.77E-11 | 0.254 | **1.000** |
| rs738408 | rs738408 | 22 | 44324730 | 1.00E-17 | 0.479 | 0.479 |
|  | rs738409 | 22 | 44324727 | 1.90E-17 | 0.254 | 0.733 |
|  | rs3747207 | 22 | 44324855 | 1.91E-17 | 0.253 | 0.985 |
|  | rs2294915 | 22 | 44340904 | 4.88E-16 | 0.010 | 0.996 |
| rs879882 | rs879882 | 6 | 31139452 | 1.22E-15 | 0.937 | 0.937 |
|  | rs3132520 | 6 | 31140008 | 6.52E-14 | 0.019 | 0.956 |
|  | rs3130695 | 6 | 31211050 | 1.48E-13 | 0.008 | 0.964 |
|  | rs3132497 | 6 | 31208591 | 2.12E-13 | 0.006 | 0.970 |
|  | rs3095249 | 6 | 31209245 | 2.12E-13 | 0.006 | 0.976 |
|  | rs3130408 | 6 | 31213791 | 2.24E-13 | 0.006 | 0.981 |
|  | rs3134756 | 6 | 31214873 | 2.24E-13 | 0.006 | 0.987 |
|  | rs2240063 | 6 | 31114745 | 7.27E-13 | 0.002 | 0.989 |
|  | rs3131012 | 6 | 31115441 | 9.39E-13 | 0.001 | 0.990 |

CHR, chromosome; CumSum, cumulative sum of posterior inclusion probability; PIP, posterior inclusion probability; POS, position; SNPs, single nucleotide Polymorphisms.

## Table S14. Results of colocalization analysis for each pleiotropic locus identified from CPASSOC between type 2 diabetes mellitus and gallstone disease‡.

| **SNPs** | **A1** | **A2** | **BETA** | |  | ***P*_value** | | | **No. of SNPs** | **PP H_0_** | **PP H_1_** | **PP H_2_** | **PP H_3_** | **PP H_4_** |
| --- | --- | --- | --- | --- | --- | --- | --- | --- | --- | --- | --- | --- | --- | --- |
|  |  |  | **T2DM** | **GSD** |  | **T2DM** | **GSD** | **CPASSOC** |  |  |  |  |  |  |
| rs58304657 | C | G | -0.0724 | 0.069 |  | 7.92E-09 | 1.40E-11 | 3.20E-19 | 2737 | 1.78E-29 | 2.68E-16 | 6.63E-14 | **1.000** | 0.000 |
| rs13029250 | T | G | 0.0731 | -0.032 |  | 9.00E-22 | 4.50E-07 | 2.22E-22 | 3125 | 0.00E+00 | 0.00E+00 | 1.75E-27 | **1.000** | 0.000 |
| rs72870502 | T | C | 0.0662 | -0.044 |  | 1.40E-10 | 2.50E-07 | 1.35E-14 | 3109 | 0.00E+00 | 0.00E+00 | 1.75E-27 | **1.000** | 0.000 |
| rs1169288 | C | A | -0.0808 | -0.049 |  | 5.84E-23 | 7.30E-13 | 5.75E-30 | 3032 | 1.94E-33 | 9.15E-21 | 2.12E-13 | **1.000** | 0.000 |
| rs1169307 | C | T | -0.0572 | -0.041 |  | 1.63E-13 | 4.50E-10 | 3.19E-20 | 3014 | 1.94E-33 | 9.15E-21 | 2.12E-13 | **1.000** | 0.000 |
| rs7461273 | G | C | -0.0383 | -0.035 |  | 6.92E-07 | 9.60E-08 | 1.20E-12 | 2766 | 1.22E-12 | 3.47E-09 | 3.50E-04 | **1.000** | 0.000 |
| rs35134156 | G | A | 0.0352 | -0.041 |  | 4.05E-06 | 4.10E-10 | 5.53E-15 | 1649 | 1.66E-25 | 1.86E-02 | 8.73E-24 | 0.978 | 0.003 |
| rs519790 | G | C | 0.0375 | -0.037 |  | 4.05E-06 | 3.80E-08 | 1.31E-12 | 1978 | 4.53E-31 | 4.36E-02 | 9.92E-30 | 0.956 | 0.000 |
| rs3130279 | G | A | 0.0577 | -0.071 |  | 4.81E-07 | 1.90E-15 | 2.84E-21 | 4519 | 2.55E-16 | 4.17E-02 | 5.83E-15 | 0.956 | 0.002 |
| rs76747430 | G | A | -0.0611 | 0.04 |  | 3.40E-09 | 2.30E-06 | 1.19E-12 | 1499 | 1.14E-07 | 2.91E-07 | 2.78E-01 | 0.708 | 0.014 |
| rs10882889 | G | A | -0.0418 | 0.03 |  | 5.92E-08 | 4.30E-06 | 1.36E-11 | 2269 | 2.45E-05 | 7.00E-04 | 7.58E-03 | 0.216 | 0.776 |
| rs429358 | C | T | -0.0556 | 0.08 |  | 1.02E-07 | 1.80E-18 | 3.02E-25 | 2596 | 9.81E-16 | 1.20E-04 | 9.96E-13 | 0.121 | 0.879 |
| rs362307 | T | C | 0.0647 | 0.074 |  | 6.17E-06 | 1.10E-09 | 3.47E-14 | 2880 | 4.30E-06 | 4.54E-03 | 1.01E-04 | 0.106 | 0.889 |
| rs62052815 | T | C | -0.042 | -0.04 |  | 5.63E-08 | 6.50E-10 | 3.51E-16 | 1995 | 1.23E-08 | 7.78E-04 | 1.55E-06 | 0.097 | 0.902 |
| rs11075985 | A | C | 0.0354 | 0.12 |  | 3.92E-06 | 1.00E-74 | 3.35E-83 | 2722 | 6.75E-75 | 2.49E-02 | 1.73E-74 | 0.063 | 0.912 |
| rs2857609 | G | A | 0.055 | -0.073 |  | 9.15E-07 | 9.30E-17 | 1.36E-22 | 6859 | 7.32E-14 | 1.51E-03 | 1.21E-12 | 0.024 | 0.975 |
| rs2523504 | C | T | 0.0406 | -0.054 |  | 6.09E-06 | 6.10E-14 | 9.66E-19 | 7282 | 7.52E-14 | 1.52E-03 | 8.42E-13 | 0.016 | 0.982 |
| rs879882 | C | T | 0.0373 | -0.048 |  | 4.14E-06 | 4.00E-13 | 3.88E-18 | 8585 | 1.16E-13 | 1.45E-03 | 1.09E-12 | 0.013 | 0.986 |
| rs738408 | T | C | -0.0483 | 0.049 |  | 1.75E-07 | 1.80E-10 | 1.25E-16 | 3505 | 2.49E-08 | 3.15E-04 | 8.83E-07 | 0.010 | 0.990 |
| rs11244061 | T | C | 0.0621 | 0.057 |  | 1.60E-07 | 1.90E-08 | 5.27E-14 | 3150 | 3.93E-06 | 2.31E-04 | 6.22E-05 | 0.003 | 0.997 |
| rs28929474 | T | C | 0.3262 | -0.11 |  | 1.11E-39 | 5.90E-06 | 1.32E-41 | 3863 | 2.35E-35 | 1.44E-35 | 5.80E-03 | 0.003 | 0.992 |
| rs1260326 | C | T | 0.079 | -0.067 |  | 1.22E-23 | 1.30E-24 | 1.00E-46 | 1508 | 1.46E-37 | 5.80E-20 | 2.78E-21 | 0.000 | **1.000** |
| rs1800961 | T | C | 0.2893 | 0.16 |  | 4.48E-50 | 3.20E-20 | 5.69E-58 | 2439 | 1.42E-60 | 1.50E-46 | 9.44E-18 | 0.000 | **1.000** |

CPASSOC, Cross-Phenotype Association; GSD, gallstone disease; PP, posterior probability; SNPs, single nucleotide Polymorphisms; T2DM, type 2 diabetes mellitus.

## Table S15. Results of colocalization analysis for each pleiotropic locus identified from CPASSOC between type 2 diabetes mellitus (adjusted for BMI) and gallstone disease‡.

| **SNPs** | **A1** | **A2** | **BETA** | |  | ***P*_value** | | | **No. of SNPs** | **PP H_0_** | **PP H_1_** | **PP H_2_** | **PP H_3_** | **PP H_4_** |
| --- | --- | --- | --- | --- | --- | --- | --- | --- | --- | --- | --- | --- | --- | --- |
|  |  |  | **T2DM** | **GSD** |  | **T2DM** | **GSD** | **CPASSOC** |  |  |  |  |  |  |
| rs58304657 | C | G | -0.0724 | 0.11 |  | 7.92E-09 | 7.50E-19 | 2.50E-26 | 2737 | 1.94E-30 | 2.68E-16 | 7.24E-15 | **1.000** | 0.000 |
| rs1169288 | C | A | -0.0808 | -0.055 |  | 5.84E-23 | 6.80E-12 | 5.32E-31 | 3032 | 1.09E-29 | 9.15E-21 | 1.19E-09 | **1.000** | 0.000 |
| rs1169307 | C | T | -0.0572 | -0.042 |  | 1.63E-13 | 3.90E-08 | 1.61E-18 | 3014 | 1.09E-29 | 9.15E-21 | 1.19E-09 | **1.000** | 0.000 |
| rs3130279 | G | A | 0.0577 | -0.072 |  | 4.81E-07 | 1.30E-11 | 6.04E-16 | 4518 | 1.01E-14 | 4.18E-02 | 2.32E-13 | 0.958 | 0.001 |
| rs519790 | G | C | 0.0375 | -0.042 |  | 4.05E-06 | 1.30E-07 | 6.60E-12 | 1978 | 2.16E-34 | 4.36E-02 | 4.74E-33 | 0.956 | 0.000 |
| rs149797* | T | C | -0.0427 | 0.036 |  | 2.20E-07 | 5.70E-06 | 3.52E-11 | 2159 | 8.66E-05 | 2.09E-03 | 2.29E-02 | 0.553 | 0.422 |
| rs2857609 | G | A | 0.055 | -0.07 |  | 9.15E-07 | 1.10E-11 | 5.78E-17 | 6859 | 5.65E-10 | 2.53E-02 | 9.34E-09 | 0.418 | 0.557 |
| rs2239525 | A | G | 0.0411 | 0.059 |  | 4.81E-06 | 3.90E-12 | 7.54E-16 | 7290 | 6.61E-10 | 2.96E-02 | 7.41E-09 | 0.331 | 0.640 |
| rs244418 | A | G | -0.0377 | -0.04 |  | 1.04E-06 | 1.60E-07 | 2.94E-12 | 1959 | 2.92E-05 | 1.29E-03 | 3.68E-03 | 0.161 | 0.834 |
| rs56094641 | G | A | 0.0353 | -0.054 |  | 4.73E-06 | 1.30E-12 | 1.88E-16 | 2722 | 1.58E-09 | 2.51E-02 | 4.04E-09 | 0.063 | 0.912 |
| rs879882 | C | T | 0.0373 | -0.053 |  | 4.14E-06 | 1.50E-11 | 1.22E-15 | 8585 | 1.36E-09 | 4.54E-03 | 1.28E-08 | 0.042 | 0.954 |
| rs738408 | T | C | -0.0483 | 0.062 |  | 1.75E-07 | 3.00E-12 | 1.00E-17 | 3505 | 6.78E-10 | 3.12E-04 | 2.41E-08 | 0.010 | 0.990 |
| rs1260326 | C | T | 0.079 | -0.067 |  | 1.22E-23 | 7.40E-19 | 6.81E-40 | 1508 | 1.95E-31 | 1.81E-19 | 3.72E-15 | 0.002 | 0.998 |
| rs1800961 | T | C | 0.2893 | 0.18 |  | 4.48E-50 | 9.20E-18 | 1.44E-59 | 2439 | 2.39E-57 | 1.99E-46 | 1.59E-14 | 0.000 | 1.000 |
| rs736820 | A | G | -0.0433 | -0.035 |  | 4.41E-08 | 9.70E-06 | 1.25E-11 | 2473 | 2.39E-57 | 1.99E-46 | 1.59E-14 | 0.000 | 1.000 |

CPASSOC, Cross-Phenotype Association; GSD, gallstone disease; PP, posterior probability; SNPs, single nucleotide Polymorphisms; T2DM, type 2 diabetes mellitus; *, a novel SNP.

## Table S16. Shared transcriptome-wide association study significant genes between type 2 diabetes mellitus (adjusted for BMI) and gallstone disease.

| **Tissue** | **Gene** | **CHR** | **GSD** | |  | **T2DM** | |
| --- | --- | --- | --- | --- | --- | --- | --- |
|  |  |  | **BEST.GWAS.ID** | ***P* _Bonferroni_** |  | **BEST.GWAS.ID** | ***P* _Bonferroni_** |
| Adipose_Subcutaneous | *IFT172* | 2 | rs1260326 | 2.15E-02 |  | rs1260326 | 1.92E-04 |
| Adrenal_Gland | *GTF3C2* | 2 | rs1260326 | 2.15E-02 |  | rs1260326 | 1.92E-04 |
| Artery_Tibial | *NRBP1* | 2 | rs1260326 | 1.33E-02 |  | rs1260326 | 5.37E-05 |
| Brain_Caudate_basal_ganglia | *SNX17* | 2 | rs1260326 | 2.15E-02 |  | rs1260326 | 1.92E-04 |
| Brain_Frontal_Cortex_BA9 | *THADA* | 2 | rs4299376 | 4.56E-08 |  | rs10203174 | 1.84E-12 |
| Brain_Nucleus_accumbens_basal_ganglia | *KRTCAP3* | 2 | rs1260326 | 2.44E-04 |  | rs1260326 | 7.07E-04 |
| Brain_Spinal_cord_cervical_c-1 | *LINC01460* | 2 | rs1260326 | 3.82E-07 |  | rs1260326 | 6.23E-04 |
| Cells_Cultured_fibroblasts | *GTF3C2* | 2 | rs1260326 | 2.15E-02 |  | rs1260326 | 1.92E-04 |
| Cells_Cultured_fibroblasts | *NRBP1* | 2 | rs1260326 | 1.73E-02 |  | rs1260326 | 1.72E-04 |
| Cells_Cultured_fibroblasts | *SNX17* | 2 | rs1260326 | 1.17E-02 |  | rs1260326 | 5.17E-04 |
| Cells_EBV-transformed_lymphocytes | *NRBP1* | 2 | rs1260326 | 4.77E-02 |  | rs1260326 | 3.33E-02 |
| Colon_Sigmoid | *GTF3C2* | 2 | rs1260326 | 1.60E-02 |  | rs1260326 | 1.84E-04 |
| Esophagus_Gastroesophageal_Junction | *NRBP1* | 2 | rs1260326 | 9.93E-03 |  | rs1260326 | 8.65E-03 |
| Heart_Atrial_Appendage | *NRBP1* | 2 | rs1260326 | 4.33E-02 |  | rs1260326 | 7.96E-04 |
| Liver | *P2RX4* | 12 | rs2393791 | 4.62E-02 |  | rs1169302 | 1.32E-03 |
| Muscle_Skeletal | *KRTCAP3* | 2 | rs1260326 | 3.66E-02 |  | rs1260326 | 3.25E-02 |
| Muscle_Skeletal | *PPM1G* | 2 | rs1260326 | 4.13E-02 |  | rs1260326 | 7.96E-05 |
| Nerve_Tibial | *DMWD* | 19 | rs34255979 | 6.03E-05 |  | rs11671664 | 2.59E-03 |
| Nerve_Tibial | *IFT172* | 2 | rs1260326 | 1.75E-02 |  | rs1260326 | 1.75E-04 |
| Nerve_Tibial | *THADA* | 2 | rs13029250 | 1.14E-04 |  | rs10203174 | 1.65E-07 |
| Pituitary | *THADA* | 2 | rs13029250 | 1.13E-02 |  | rs10203174 | 1.99E-04 |
| Skin_Not_Sun_Exposed_Suprapubic | *NRBP1* | 2 | rs1260326 | 1.64E-02 |  | rs1260326 | 1.58E-04 |
| Skin_Not_Sun_Exposed_Suprapubic | *RBKS* | 2 | rs1260326 | 1.61E-07 |  | rs1260326 | 1.62E-03 |
| Skin_Sun_Exposed_Lower_leg | *NRBP1* | 2 | rs1260326 | 1.75E-02 |  | rs1260326 | 1.75E-04 |
| Testis | *OASL* | 12 | rs2393791 | 1.77E-05 |  | rs1169302 | 5.00E-05 |
| Whole_Blood | *SPPL3* | 12 | rs2393791 | 8.38E-08 |  | rs1169302 | 3.22E-05 |

CHR, chromosome; GSD, gallstone disease; T2DM, type 2 diabetes mellitus

## Figure S1. The participant selection flow chart.


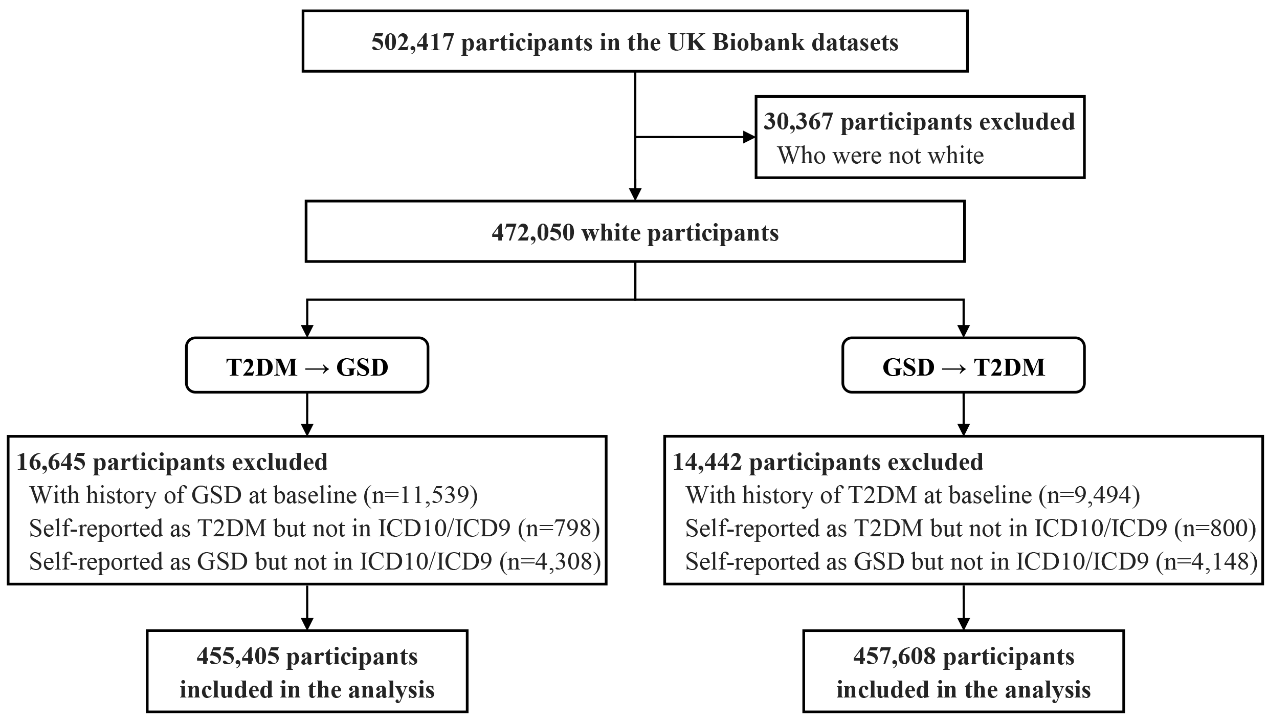


## Figure S2. Undated meta-analysis of relationship between the type 2 diabetes mellitus and gallstone disease risk.


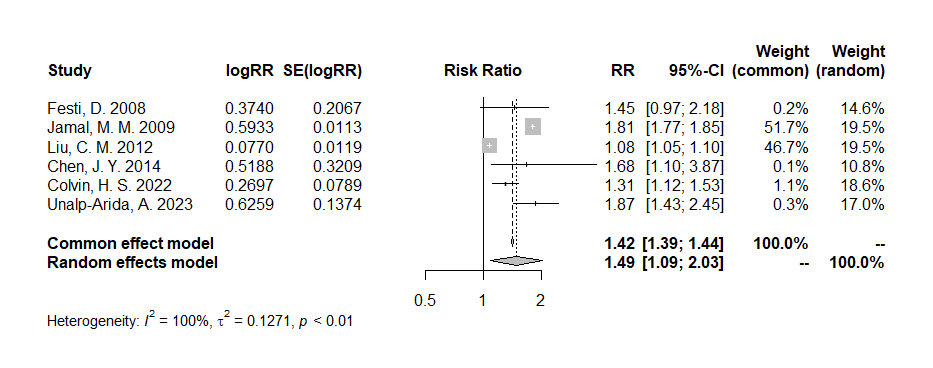

Supplement: Supplementary file 1 [file DataSheet_1.docx]
